# Synthesis and antitumor activity of novel *N*-substituted carbazole imidazolium salt derivatives

**DOI:** 10.1038/srep13101

**Published:** 2015-08-19

**Authors:** Lan-Xiang Liu, Xue-Quan Wang, Bei Zhou, Li-Juan Yang, Yan Li, Hong-Bin Zhang, Xiao-Dong Yang

**Affiliations:** 1 Key Laboratory of Medicinal Chemistry for Natural Resource, Ministry of Education, Yunnan University, Kunming, 650091, P.R. China; 2 State Key Laboratory for Phytochemistry and Plant Resources in West China, Kunming Institute of Botany, Chinese Academy of Science, Kunming, 650204, P.R. China; 3 Key Laboratory of Ethnic Medicine Resource Chemistry, State Ethnic Affairs Commission & Ministry of Education, Yunnan Minzu University, Kunming, 650500, P.R. China; 4 Research Institute of Resources Insects, Chinese Academy of Forestry, Kunming, 650224, P.R. China

## Abstract

A series of novel *N*-substituted carbazole imidazolium salt derivatives has been prepared and investigated for their cytotoxic activity against five human tumor cell lines by MTS assay. The results indicated that the existence of 5,6-dimethyl-benzimidazole ring, substitution of the imidazolyl-3-position with a 2-bromobenzyl or naphthylacyl group, as well as alkyl chain length between carbazole and imidazole ring were important for the antitumor activity. Compound 61, bearing a 2-bromobenzyl substituent at position-3 of the 5,6-dimethyl-benzimidazole, showed powerful inhibitory activities and was more selective to HL-60, SMMC-7721, MCF-7 and SW480 cell lines with IC_50_ values 0.51–2.48 μM. Mechanism of action studies revealed that this new compound could remarkably induce cell cycle arrest and apoptosis in SMMC-7721 cells. This work provides alternative novel way for future drug development based on carbazole and imidazolium salt scaffolds.

Carbazole and its derivatives are an important type of nitrogen-containing aromatic heterocyclic compounds with biological activity. Many natural products and drug molecules with the carbazole framework exhibit a broad range of biological and pharmacological activities^1^,^2^,^3^,^4^,^5^,^6^,^7^,^8^. In particular, carbazole derivatives show significant antitumor activity^9^,^10^,^11^. For example, glybomine B and C showed significant antitumor-promoting activity, which was confirmed by the inhibiting effect of these alkaloids in conjunction with the tumor promoter 12-*O*-tetradecanoylphorbol-13-acetate (TPA)^12^, while heptaphylline and 7-methoxyheptaphylline displayed strong cytotoxicity against NCI-H187 and KB cell lines (Fig. 1)^13^.

Recently, considerable attention has also been focused on imidazolium salts because of their remarkable array of biological activities, especially antitumor activity^14^,^15^,^16^,^17^. As exemplified in Fig. 1, natural compounds Lepidiline A and B, isolated from *Lepidium meyenii*, exhibited potent cytotoxic activity against a series of human cancer cell lines^18^. Meanwhile, the synthesis and potential cytotoxic activity of a series of new imidazolium salt derivatives, such as NMIB (Fig. 1), were reported in our previous literatures^19^,^20^,^21^,^22^,^23^. The antitumor mechanisms underlying arresting cell cycle progression and triggering tumour cell death by apoptosis have been validated for imidazolium salt derivatives^22^,^23^.

During the past 10 years, a pharmacophore hybrid approach for exploration of novel and highly bioactive compounds has been an effective and commonly used trend in the drug discovery field^24^,^25^,^26^,^27^,^28^. To validate synergistic integration of the anticancer activity of carbazole derivatives and the potent cytotoxic activity of imidazolium salts, we were interested in synthesizing a series of hybridizing compounds of carbazole with imidazole moieties. To the best of our knowledge, no reports concerning antitumor activity of carbazole imidazolium salt derivatives have been found in the literature.

In this paper, a series of novel *N*-substituted carbazole imidazolium salt derivatives were prepared. The purpose of this study was to investigate the antitumor activity of carbazole-based imidazole hybrids, with the final goal of developing potent antitumor agents.

## Results and Discussion

### Chemistry

**Figure 2:**
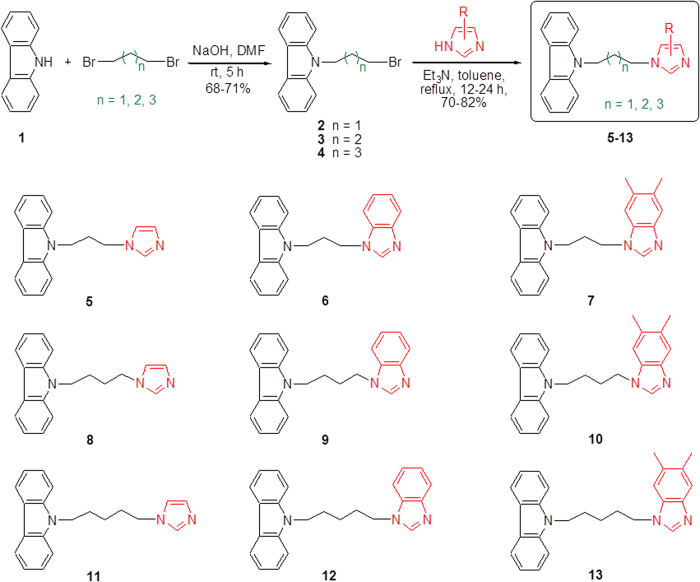
Synthesis of *N*-substituted carbazole–imidazole hybrids 5–13.

To prepare the *N*-substituted carbazole–imidazole hybrids (**5**–**13**), we used commercially available imidazole derivatives that were alkylated with *N*-alkyl bromide substituted carbazole, which was synthesized from readily available starting material carbazole **1** as depicted in Fig. 2. Straight chain alkyl groups (propyl, butyl and pentyl) were selected as linkers in the target compounds. Firstly, carbazole **1** reacted with dibromo alkane (1,3-dibromopropane, 1,4-dibromobutane or 1,5-dibromopentane) in the presence of sodium hydroxide to form the respective *N*-alkylbromide substituted carbazole **2**–**4** with 68–71% yield^29^. Next, bromide carbazole **2**–**4** was transformed to the corresponding nine *N*-substituted carbazole–imidazole hybrids **5**–**13** with imidazole or substituted benzimidazole (benzimidazole or 5,6-dimethyl-benzimidazole) by refluxing under acetone in 70–82% yields.

**Figure 3:**
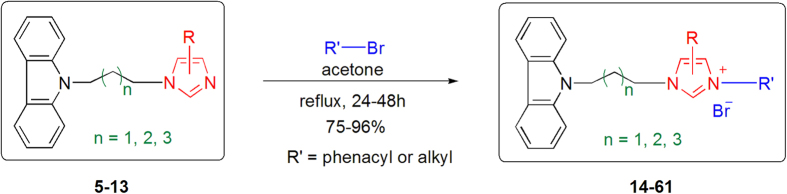
Synthesis of *N*-substituted carbazole imidazolium salt derivatives 14–61.

Finally, forty-eight *N*-substituted carbazole imidazolium salt derivatives **14**–**61** were synthesized with excellent yields by reaction of *N*-substituted carbazole–imidazole hybrids **5**–**13** with the corresponding alkyl and phenacyl bromides in refluxing acetone with 75–96% yields (Fig. 3). The structures and yields of imidazolium salt derivatives are listed in Table 1.

**Figure 4:**
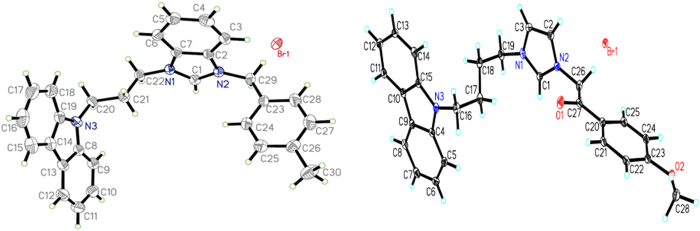
X-ray crystal structures of imidazolium salts 24 and 30.

In order to confirm the chemical structures of the *N*-substituted carbazole imidazolium salt derivatives, compounds **24** and **30** were selected as the model compounds and determined by means of single-crystal X-ray diffraction analysis (the Cambridge Crystallographic Data Centre (CCDC) 1058661 and 1058662 contain the supplementary crystallographic data for compound **24** and **30**. These data can be obtained free of charge from the Cambridge Crystallographic Data Center via www.ccdc.cam.ac.uk/ data_request/cif). The molecular structures are shown in Fig. 4.

### Biological evaluation and structure-activity relationship analysis

The cytotoxic potential of all newly synthesized imidazole and imidazolium salt derivatives toward five human tumor cell lines, HL-60 (myeloid leukaemia), SMMC-7721 (liver cancer), A549 (lung cancer), MCF-7 (breast cancer) and SW480 (colon cancer), were screened *in vitro* using MTS assay^30^. DDP (Cisplatin), as well as carbazole (**1**) and imidazole, were chosen as positive controls. The screening results are summarized in Table 2.

As shown in Table 2, carbazole (1) and imidazole, as controls, lacked activity against all tumor cell lines investigated at the concentration of 40 μM. However, fifty-seven designed compounds (**5**–**61**) exhibited broad inhibitory effects against five tested cell lines. Obviously, the structures of carbazole-based imidazole derivatives and imidazolium salt derivatives have made a significant impact on their antitumor activity. *N*-substituted carbazole–imidazole hybrids **5**–**13** exhibited no inhibitory or very weak activities against five tested cell lines. In contrast, *N*-substituted carbazole–imidazolium salts **14**–**61** displayed moderate to good cytotoxic potential. This could be understandable because of the changes of molecular structure, charge distribution and water solubility^31^.

For the alkyl chain between carbazole and imidazole ring, the inhibitory activities of imidazolium salt derivatives against five tumor cell lines strengthened with the increase of alkyl chain length (n = 3 > 2 > 1, propyl in **14**–**28**, butyl in **29**–**46** and pentyl in **47**–**61**). Firstly, compounds **14**–**28** with propyl group showed relatively weak activities against five cell lines. Among them, compound **22**, bearing naphthylacyl substituent at position-3 of the benzimidazole, displayed higher cytotoxic activities with IC_50_ values of 2.69–5.65 μM. Secondly, imidazolium salts **29**–**46** with butyl group displayed medium cytotoxic activities with IC_50_ values of 0.49–19.98 μM. Finally, compounds **47**–**61** with pentyl group exhibited strong cytotoxic activities with IC_50_ values below 4.50 μM and more active than DDP (except compounds **47** and **50**).

For the imidazole ring (imidazole, benzimidazole or 5,6-dimethyl-benzimidazole), imidazolium salt derivatives **14**–**19**, **29**–**34** and **47**–**49** with imidazole ring showed relatively low inhibitory activities against five cell lines. Most this kind compounds exhibited weak cytotoxic activities with IC_50_ values above 10.00 μM. Only compounds **47**–**49**, with pentyl group between carbazole and imidazole ring, showed higher inhibitory activities with IC_50_ values of 0.55–8.69 μM. In comparison, imidazolium salt derivatives **20**–**25**, **35**–**40** and **50**–**55** with benzimidazole ring exhibited higher inhibitory activities with IC_50_ values of 0.56–25.87 μM. Among them, there were one half of compounds (9/18) with IC_50_ values below 5.00 μM. Notably, imidazolium salt derivatives **26**–**28**, **41**–**46** and **56**–**61** with 5,6-dimethyl-benzimidazole ring displayed strong inhibitory activities. Most this kind compounds showed powerful inhibitory activities with IC_50_ values below 5.00 μM and were significantly more active than DDP. Among them, compounds **61** and **62**, bearing a 4-methylbenzyl or 2-bromobenzyl substituent at position-3 of the 5,6-dimethyl-benzimidazole, exhibited remarkable inhibitory activities with IC_50_ values of 0.51–3.12 μM against five test cell lines.

For the substituents of imidazolium salts, a phenacyl substituent at position-3 of imidazole ring, such as compounds **14**, **20**, **32**, **38** and **50**, decreased the inhibitory activities against five tumor cell lines, while a 4-bromophenacyl substituent, such as compounds **17**, **23**, **31**, **37** and **53**, could slightly improve the inhibitory activities. In contrast, a 4-methoxyphenacyl substituent in compounds **21**, **27**, **30**, **42**, **51** and **57**, or a 4-methylbenzyl substituent in compounds **24**, **28**, **33**, **45**, **57** and **60** have positive effects on the inhibitory activities against tumor cell lines. Interestingly, compared with above substituents, a naphthylacyl substituent at position-3 of imidazole ring, such as compounds **22**, **26**, **35**, **41**, **48**, **52** and **58**, or a 2-bromobenzyl substituent in compounds **34**, **40**, **46**, **55** and **61** could led to substantial improvement of the antitumor activity. It can be seen that most of these kinds of derivatives displayed strong cytotoxic activities and were much more active than DDP. Especially, compound **61**, bearing a 2-bromobenzyl substituent at position-3 of the 5,6-dimethyl-benzimidazole, showed excellent inhibitory activities and was more selective to HL-60, SMMC-7721, MCF-7 and SW480 cell lines with IC_50_ values 0.51–2.48 μM.

**Figure 5:**
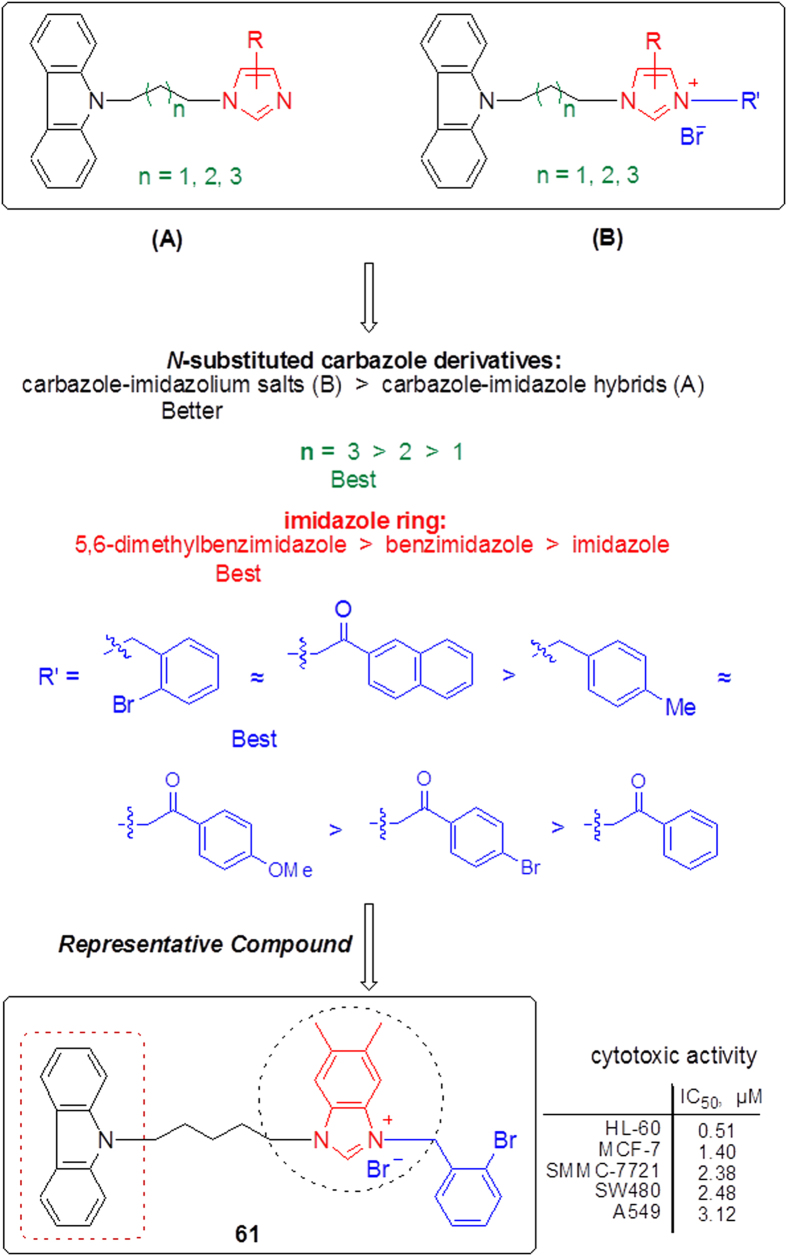
Structure-activity relationship of *N*-substituted carbazole imidazolium salt derivatives.

The results indicated that the existence of 5,6-dimethyl-benzimidazole ring and substitution of the imidazolyl-3-position with a 2-bromobenzyl or naphthylacyl group were important for the antitumor activity. Moreover, the increase of alkyl chain length (n = 3 > 2 > 1) also led to enhance of the inhibitory activity. Overall, the structure-activity relationship (SAR) results of *N*-substituted carbazole imidazolium salt derivatives have been depicted in Fig. 5.

### Apoptosis and arrest of the SMMC-7721 cells induced by selected derivative

**Figure 6:**
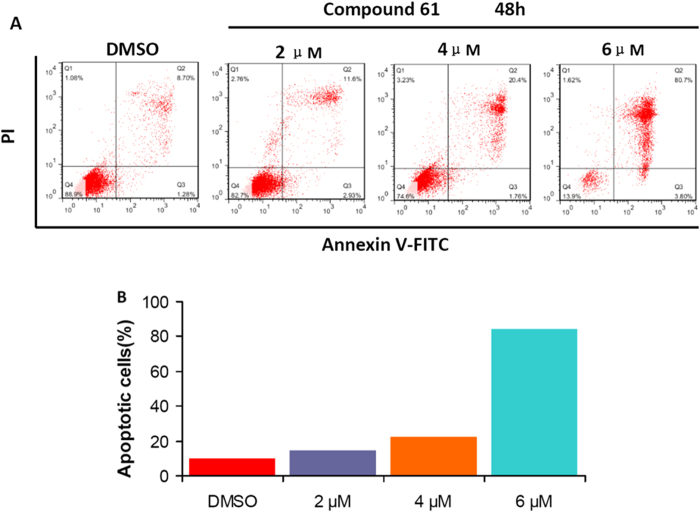
Compound 61 induce significant apoptosis of SMMC-7721 cells. (**A**) Cells were treated with 2, 4 and 6 μM compound **61** for 48 h. Treatment with **61** increased the early apoptotic (Annexin V+/PI−, lower right quadrant) and late apoptotic (Annexin V+/PI+, upper right quadrant) cells. (**B**) The quantification of cell apoptosis. Data represents the mean of three independent experiments.

We then explored the mechanisms of action of these new *N*-substituted carbazole imidazolium salt derivatives. Initially, compound **61** was examined for apoptosis-induction ability. Apoptosis in SMMC-7721 cells was induced by treatment with compound **61** in a dose-dependent manner for 48 h. Apoptotic cell number increased to 14.83%, 22.26% and 84.5% when the cells were treated with compound **61** at 2, 4 and 6 μM, respectively, which were statistically different from the control (9.98%) (Fig. 6). These results showed that *N*-substituted carbazole imidazolium salt **61** can remarkably induce apoptosis of the SMMC-7721 cells.

**Figure 7:**
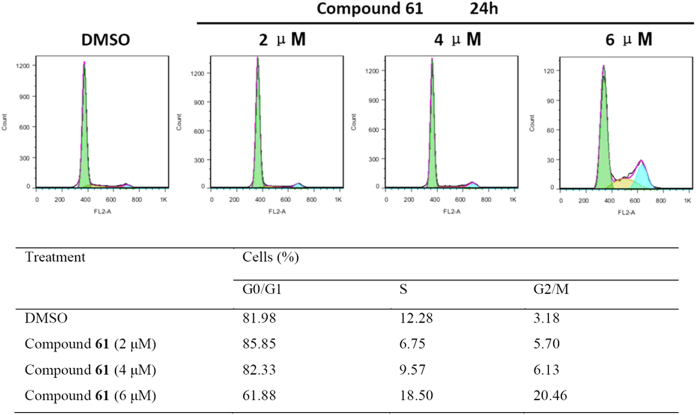
Compound 61 induces G2/M phase arrest in SMMC-7721 cells. (**A**) Cells were treated with 2, 4 and 6 μM of compound **61** for 24 h. Cell cycle was determined by PI staining and cell cytometry. (**B**) The percentages of cells in different phases were quantified. At least three independent experiments were performed and data of one representative experiment is shown.

To further examine how new imidazolium salts suppressed the growth of SMMC-7721 cells, the effect of compound **61** on cell cycle distribution was investigated and the results of a typical experiment are shown in Fig. 7. SMMC-7721 cells were treated with compound **61** for 24 h, resulting in an obvious increase of the percentage of cells in G2/M phase when compared with the control. Compound **61** treatment caused 20.46% cells in G2/M phase as compared to control showing 3.18%. Inversely, G1 phase cell population was decreased to 61.88% as compared to control having 81.98%, while the proportion of S phase cells showed no significant change. These results suggested the role of cell cycle arrest in compound **61**-induced growth inhibition of SMMC-7721 cells. This result is significant because disruption or malfunction of cell cycle control within the G2/M phase has been recognized as one of the most important biochemical phenomenon for tumor progression and tumorigenesis^32^.

In summary, a series of novel *N*-substituted carbazole imidazolium salt derivatives has been prepared in the present study and characterized by ^1^H-NMR, ^13^C-NMR, HRMS, IR, and single-crystal X-ray diffraction. All derivatives were evaluated *in vitro* against five human tumor cell lines for their cytotoxicity profile. The results indicated that the existence of 5,6-dimethyl-benzimidazole ring, substitution of the imidazolyl-3-position with a 2-bromobenzyl or naphthylacyl group, as well as alkyl chain length between carbazole and imidazole ring were important for the antitumor activity. Imidazolium salts **51**, **52**, **54**, **55, 58**, **60** and **61** were found to be the most potent compounds. Notably, compound **61**, bearing a 2-bromobenzyl substituent at position-3 of the 5,6-dimethyl-benzimidazole, showed powerful inhibitory activities and was more selective to HL-60, SMMC-7721, MCF-7 and SW480 cell lines with IC_50_ values 0.51–2.48 μM. Mechanism of action studies revealed that this new compound could remarkably induce cell cycle arrest and apoptosis in SMMC-7721 cells. This work provides alternative novel way for future drug development based on carbazole and imidazolium salt scaffolds. Further studies on the mechanism and structural modifications of these *N*-substituted carbazole imidazolium salt derivatives are underway in our laboratories.

## Methods

### General procedures

Melting points were obtained on a XT-4 melting-point apparatus and were uncorrected. Proton nuclear magnetic resonance (^1^H-NMR) spectra were recorded on a Bruker Avance 300/400 spectrometer at 300/400 MHz. Carbon-13 nuclear magnetic resonance (^13^C-NMR) was recorded on Bruker Avance 300/400 spectrometer at 75/100 MHz. Chemical shifts are reported as δ values in parts per million (ppm) relative to tetramethylsilane (TMS) for all recorded NMR spectra. Low-resolution Mass spectra were recorded on a VG Auto Spec-3000 magnetic sector MS spectrometer. High Resolution Mass spectra were taken on AB QSTAR Pulsar mass spectrometer. X-Ray data was determined using a Bruker APEX JASCO P-1020 polarimeter. Silica gel (200–300 mesh) for column chromatography and silica GF_254_ for TLC were produced by Qingdao Marine Chemical Company (China). All air- or moisture-sensitive reactions were conducted under an argon atmosphere. Starting materials and reagents used in reactions were obtained commercially from Acros, Aldrich, Fluka and were used without purification, unless otherwise indicated.

### Synthesis of compounds 2–4

To a mixture of carbazole **1** (1.5 g, 9 mmol) and NaOH (520 mg, 13 mmol) in DMF (30 mL) at 0 °C was added alkyl dibromide (27 mmol). The reaction mixture was stirred at room temperature for 5 h. Reaction progress was monitored by TLC, then diluted with water (50 mL), and extracted with ether (20 mL×3). The combined organic layers were washed with brine (20 mL), dried over anhydrous Na_2_SO_4_ and concentrated. The residue was purified by column chromatography (silica gel, petroleum ether 60–90 °C: EtOAc = 5:1) to afford **2**–**4** in 68–72% yield as white powder.

#### 9-(3-Bromopropyl*)*-9H-carbazole (**2**)

Yield 68%. White powder, m.p. 148–150 °C. ^1^H NMR (300 MHz, CDCl_3_) δ: 8.06 (2H, d, J = 9.0 Hz), 7.47–7.44 (4H, m), 7.43–7.41 (2H, m), 4.42 (2H, t, J = 6.0 Hz), 3.31 (2H, t, J = 6.0 Hz), 2.40–2.32 (2H, m). ^13^C NMR (75 MHz, CDCl_3_): δ 140.04, 125.90, 123.02, 120.51, 119.20, 108.72, 41.03, 32.04, 30.91.

#### 9-(4-Bromobutyl)-9H-carbazole (**3**)

Yield 68%. White powder, m.p. 104–106 °C. ^1^H NMR (300 MHz, CDCl_3_): δ 8.08 (2H, d, 004A = 6.0 Hz), 7.46 (2H, t, J = 6.0 Hz), 7.38–7.36 (2H, m), 7.19 (2H, t, J = 3.0 Hz), 4.26 (2H, t, J = 5.4 Hz), 3.30 (2H, t, J = 5.4 Hz), 1.90–1.77 (4H, m), 1.53–1.43 (2H, m). ^13^C NMR (75 MHz, CDCl_3_): δ140.39, 125.71, 122.91, 120.44, 118.90, 108.61, 42.82, 33.36, 32.50, 28.21, 25.93.

#### 9-(5-Bromopentyl)-9H-carbazole (**4**)

Yield 72%. White powder, m.p. 51–53 °C. ^1^H NMR (300 MHz, CDCl_3_) δ: 8.08 (2H, d, J = 8.7 Hz), 7.47–7.41 (2H, m), 7.35 (2H, d, J = 8.1 Hz), 7.24–7.19 (2H, m), 4.26 (2H, t, J = 6.9 Hz), 3.30 (2H, t, J = 6.9 Hz), 1.90–1.77 (4H, m), 1.53–1.43 (2H, m). ^13^C NMR (75 MHz, CDCl_3_): δ 140.38, 125.71, 122.91, 120.44, 118.90, 108.61, 42.83, 33.37, 32.50, 28.21, 25.93.

### Synthesis of compounds 5–13

A mixture of compound **2**–**4** (2 mmol) and imidazole or substituted imidazole (6 mmol) and Et_3_N (3 mmol) was stirred in tuloene (20 ml) at reflux for 12–24 h (monitored by TLC). After cooling to room temperature, the solvent was concentrated, and the residue was diluted with EtOAc (20 mL). The organic layer was washed with water (20 mL) and brine (20 mL), dried over anhydrous Na_2_SO_4_ and concentrated. The residue was purified by column chromatography (silica gel, petroleum ether 60–90 °C: EtOAc = 3:1) to afford **5–13** in 68–72% yield as powder or oil.

#### 9-(3-(1H-Imidazol-1-yl)propyl)-9H-carbazole (**5**)

Yield 68%. Yellow powder, m.p. 101–103 °C. IR *ν*_max_ (cm^−1^): 3425, 3106, 3050, 2947, 2872, 1595, 1487, 1453, 1338, 1227, 1163, 1074, 907, 822, 752, 663. ^1^H NMR (300 MHz, CDCl_3_) δ: 8.05 (2H, d, J = 7.8 Hz), 7.41 (2H, t, J = 7.8 Hz), 7.34 (1H, s), 7.23–7.15 (4H, m), 7.06 (1H, s), 6.79 (1H, s), 4.16 (2H, d, J = 6.9 Hz), 3.79 (2H, d, J = 6.9 Hz), 2.29–2.22 (2H, m). ^13^C NMR (75 MHz, CDCl_3_) δ: 140.07, 137.10, 129.77, 125.96, 123.04, 120.54, 119.36, 118.60, 108.36, 44.33, 39.66, 29.83. HRMS (ESI-TOF) m/z Calcd for C_18_H_18_N_3_ [M+1]^+^ 276.1501, found 276.1497.

#### 9-(3-(1H-Benzo[d]imidazol-1-yl)propyl)-9H-carbazole (**6**)

Yield 70%. White powder, m.p. 45–47 °C. IR *ν*_max_ (cm^−1^): 3401, 3051, 2932, 2876, 1599, 1490, 1453, 1376, 1332, 1254, 1215, 1163, 1063, 1008, 930, 889, 747, 624.^1^H NMR (300 MHz, CDCl_3_) δ: 8.08 (2H, d, J = 6.0 Hz), 7.81 (1H, d, J = 9.0 Hz), 7.75 (1H, s), 7.41 (2H, t, J = 7.5 Hz), 7.35–7.17 (6H, m), 7.12 (1H, d, J = 6.0 Hz), 4.26 (2H, t, J = 7.5 Hz), 4.04 (2H, t, J = 7.5 Hz), 2.47–2.40 (2H, m). ^13^C NMR (75 MHz, CDCl_3_) δ: 189.98, 155.93, 155.31, 136.70, 134.15, 131.33, 131.20, 130.42, 128.07, 126.60, 124.47, 124.09, 123.35, 123.16, 121.28, 120.84, 119.90, 116.38, 116.08, 112.19, 111.81, 58.72, 55.46, 20.85. HRMS (ESI-TOF) m/z Calcd for C_22_H_20_N_3_ [M+1]^+^ 326.1657, found 326.1649.

#### 9-(3-(5,6-Dimethyl-1H-benzo[d]imidazol-1-yl)propyl)-9H-carbazole (**7**)

Yield 72%. White powder, m.p. 195–197 °C. IR *ν*_max_ (cm^−1^): 3425, 3052, 3096, 1595, 1485, 1456, 1333, 1257, 1215, 1164, 1058, 1006, 845, 755, 618. ^1^H NMR (300 MHz, CDCl_3_) δ: 8.12–8.07 (2H, m), 7.69 (1H, s), 7.60 (1H, s), 7.46–7.40 (2H, m), 7.27–7.22 (4H, m), 6.84 (1H, s), 4.32 (2H, t, J = 6.6 Hz), 4.04 (2H, t, J = 7.2 Hz), 2.54–2.34 (2H, m), 2.35 (3H, s), 2.29 (3H, s). ^13^C NMR (75 MHz, CDCl_3_) δ: 142.64, 141.98, 140.11, 132.24, 132.06, 131.18, 125.95, 123.10, 120.59, 120.52, 119.35, 109.73, 108.45, 42.39, 39.94, 28.76, 20.52, 20.25. HRMS (ESI-TOF) m/z Calcd for C_24_H_24_N_3_ [M+1]^+^ 353.1970, found 354.1961.

#### 9-(4-(1H-imidazol-1-yl)butyl)-9H-carbazole (**8**)

Yield 70%. White powder, m.p. 267–270 °C. IR *ν*_max_ (cm^−1^): 3111, 3054, 2932, 2868, 1594, 1499, 1452, 1327, 1228, 1156, 1069, 1027, 911, 849, 747, 622. ^1^H NMR (300 MHz, DMSO) δ: 8.05 (2H, d, J = 7.8 Hz), 7.42 (2H, t, J = 8.1 Hz), 7.39 (1H, s), 7.24–7.15 (4H, m), 7.06 (1H, s), 6.79 (1H, s), 4.16 (2H, t, J = 6.9 Hz), 3.80 (2H, t, J = 6.9 Hz), 2.29–2.22 (2H, m). ^13^C NMR (75 MHz, DMSO) δ: 140.07, 137.10, 129.77, 125.97, 123.04, 120.54, 119.36, 118.59, 108.36, 44.33, 39.67, 29.83. HRMS (ESI-TOF) m/z Calcd for C_19_H_20_N_3_ [M+1]^+^ 290.1657, found 290.1652.

#### 9-(4-(1H-benzo[d]imidazol-1-yl)butyl)-9H-carbazole(**9**)

Yield 72%. White powder, m.p. 110–112 °C. IR *ν*_max_ (cm^−1^): 3414, 3050, 2934, 2867, 1598, 1489, 1453, 1372, 1334, 1239, 1159, 1067, 1007, 930, 860, 740, 626. ^1^H NMR (300 MHz, CDCl3) δ: 8.09 (2H, d, J = 7.8 Hz), 7.79 (1H, t, J = 5.7 Hz), 7.65 (1H, s), 7.44 (2H, t, J = 7.5 Hz), 7.32–7.29 (2H, m), 7.26–7.21 (5H, m), 4.26–4.24 (2H, m), 3.96–3.94 (2H, m), 1.88–1.86 (4H, m). ^13^C NMR (75 MHz, DMSO) δ: 143.87, 142.79, 140.22, 133.63, 125.85, 122.93, 122.13, 120.54, 119.15, 109.49, 108.51, 44.64, 42.35, 27.74, 26.28. HRMS (ESI-TOF) m/z Calcd for C_23_H_22_N_3_ [M+1]^+^ 340.1814, found 340.1810.

#### 9-(4-(5,6-dimethyl-1H-benzo[d]imidazol-1-yl)butyl)-9H-carbazole (**10**)

Yield 72%. White powder, m.p. 112–114 °C. IR *ν*_max_ (cm^−1^): 3425, 3051, 2944, 2864, 1594, 1489, 1454, 1335, 1216, 1161, 1061, 1008, 933, 844, 751, 618. ^1^H NMR (300 MHz, CDCl_3_) δ: 8.08 (2H, d, J = 7.5 Hz), 7.54 (2H, d, J = 6.3 Hz), 7.43 (2H, t, J = 8.1 Hz), 7.29 (2H, d, J = 8.1 Hz), 7.22 (2H, t, J = 7.5 Hz), 6.97 (1H, s), 4.25–4.22 (2H, m), 3.90–3.88 (2H, m), 2.34 (6H, s), 1.86–1.84 (4H, m). ^13^C NMR (75 MHz, CDCl_3_) δ: 142.53, 142.08, 140.24, 132.16, 132.07, 131.00, 125.83, 122.92, 120.50, 120.44, 119.11, 109.67, 108.53, 44.58, 42.35, 27.68, 26.27, 20.61, 20.26. HRMS (ESI-TOF) m/z Calcd for C_25_H_26_N_3_ [M+1]^+^ 368.2127, found 368.2118.

#### 9-(5-(1H-imidazol-1-yl)pentyl)-9H-carbazole (**11**)

Yield 70%. Yellow oil. IR *ν*_max_ (cm^−1^): 3419, 3049, 2933, 2859, 1595, 1486, 1456, 1328, 1229, 1154, 1077, 909, 818, 726, 664. ^1^H NMR (300 MHz, DMSO) δ: 8.07 (2H, d, J = 7.8 Hz), 7.46–7.41 (2H, m), 7.33–7.30 (3H, m), 7.24–7.19 (2H, m), 7.01 (1H, s), 6.74 (1H, s), 4.22 (2H, t, J = 6.9 Hz), 3.73 (2H, t, J = 6.9 Hz), 1.87–1.77 (2H, m), 1.71–1.61 (2H, m), 1.33–1.25 (2H, m). ^13^C NMR (75 MHz, DMSO) δ: 140.31, 136.97, 129.47, 125.73, 122.87, 120.45, 118.96, 118.73, 108.55, 46.69, 42.67, 30.92, 28.46, 24.36. HRMS (ESI-TOF) m/z Calcd for C_20_H_22_N_3_ [M+1]^+^ 304.1814, found 304.1810.

#### 9-(5-(1H-benzo[d]imidazol-1-yl)pentyl)-9H-carbazole (**12**)

Yield 72%. Yellow powder, m.p. 149–151 °C. IR *ν*_max_ (cm^−1^): 3436, 3051, 2933, 2862, 1923, 1807, 1738, 1598, 1487, 1451, 1368, 1331, 1243, 1203, 1155, 1119, 1074, 1009, 929, 880, 839, 744, 619. ^1^H NMR (300 MHz, CDCl_3_) δ: 8.08 (2H, d, J = 7.8 Hz), 7.81–7.78 (1H, m), 7.72 (1H, s), 7.46–7.41 (2H, m), 7.32–7.19 (7H, m), 4.21(2H, t, J = 6.9 Hz), 3.98 (2H, t, J = 6.9 Hz), 1.87–1.75 (4H, m), 1.35–1.30 (2H, m). ^13^C NMR (75 MHz, CDCl_3_): δ143.90, 142.82, 140.31, 133.75, 125.74, 122.89, 122.09, 120.46, 118.97, 109.57, 108.54, 44.77, 42.66, 29.78, 28.53, 24.71. HRMS (ESI-TOF) m/z Calcd for C_24_H_24_N_3_ [M+1]^+^ 354.1970, found 354.1957.

#### 9-(5-(5,6-dimethyl-1H-benzo[d]imidazol-1-yl)pentyl)-9H-carbazole (**13**)

Yield 72%. White powder, m.p. 103–105 °C. IR *ν*_max_ (cm^−1^): 3415, 3053, 2928, 2859, 1594, 1490, 1455, 1490, 1455, 1334, 1273, 1216, 1156, 1067, 1008, 841, 752, 618. ^1^H NMR (300 MHz, CDCl_3_) δ: 8.07 (2H, d, J = 7.8 Hz), 7.62 (1H, s), 7.56 (1H, s), 7.46–7.41 (2H, m), 7.31–7.29 (2H, m), 7.21 (2H, t, J = 7.5 Hz), 7.04 (1H, s), 4.19 (2H, d, J = 6.9 Hz), 3.93 (2H, d, J = 6.9 Hz), 2.36 (6H, s), 1.85–1.73 (4H, m), 1.36–1.31 (2H, m). ^13^C NMR (75 MHz, CDCl_3_) δ: 142.45, 142.09, 140.31, 132.26, 132.08, 131.01, 125.74, 122.86, 120.45, 120.36, 118.95, 109.77, 108.55, 44.74, 42.68, 29.74, 28.57, 24.66, 20.65, 20.29. HRMS (ESI-TOF) m/z Calcd for C_26_H_28_N_3_ [M+1]^+^ 382.2283, found 382.2777.

### Synthesis of compounds 14–61

A mixture of substituted imidazole **5–13** (0.25 mmol) and phenacyl or alkyl (0.75 mmol) was stirred in acetone (10 ml) at reflux 24–48 h (10 ml). An insoluble substance was formed. After completion of the reaction as indicated by TLC, the precipitate was filtered and washed with acetone (3 × 10 ml), then dried to afford imidazolium salts **14–61** in 75–96% yields.

#### 1-(3-(9H-carbazol-9-yl)propyl)-3-(2-oxo-2-phenylethyl)-1H-imidazol-3-iumbromide (**14**)

Yield 94%. Yellow powder, m.p. 124–126 °C. IR *ν*_max_ (cm^−1^): 3395, 3134, 3065, 2963, 1697, 1595, 1454, 1338, 1229, 1166, 991, 754, 684. ^1^H NMR (300 MHz, MeOH) δ: 8.79 (1H, s), 8.05 (2H, d, J = 9.0 Hz), 7.98 (2H, d, J = 9.0 Hz), 7.65 (1H, d, J = 9.0 Hz), 7.55–7.50 (4H, m), 7.48–7.41 (4H, m), 7.19 (2H, d, J = 9.0 Hz), 5.76 (2H, s), 4.43 (2H, t, J = 6.0 Hz), 4.25 (2H, t, J = 6.0 Hz), 2.44–2.35 (2H, m). ^13^C NMR (75 MHz, MeOH) δ: 191.90, 141.41, 138.43, 135.81, 130.22, 129.41, 127.23, 125.33, 124.91, 122.93, 121.42, 120.48, 110.12, 56.71, 56.52, 40.76, 30.03. HRMS (ESI-TOF) m/z Calcd for C_26_H_24_N_3_O [M-Br]^+^ 394.1914, found 394.1910.

#### 1-(3-(9H-carbazol-9-yl)propyl)-3-(2-(4-methoxyphenyl)-2-oxoethyl)-1H-imidazol-3-ium bromide (**15**)

Yield 95%. White powder, m.p. 112–114 °C. IR *ν*_max_ (cm^−1^): 3415, 3141, 3054, 2965, 2839, 1684, 1600, 1454, 1335, 1240, 1167, 1025, 835, 755. ^1^H NMR (300 MHz, MeOH) δ: 8.74 (1H, s), 8.00 (2H, d, J = 6.0 Hz), 7.86 (2H, d, J = 9.0 Hz), 7.46–7.40 (4H, m), 7.36 (2H, s), 7.16 (2H, t, J = 7.5 Hz), 6.93 (2H, t, J = 9.0 Hz), 5.62 (2H, s), 4.36 (2H, t, J = 6.0 Hz), 4.19 (2H, t, J = 9.0 Hz), 3.36 (3H, s), 2.32 (2H, m). ^13^C NMR (75 MHz, MeOH) δ: 190.21, 166.20, 141.30, 138.44, 131.92, 127.62, 127.21, 125.33, 124.11, 122.81, 121.52, 120.51, 115.42, 110.22, 56.41, 56.10, 40.72, 30.02. HRMS (ESI-TOF) m/z Calcd for C_27_H_26_N_3_O_2_ [M-Br]^+^ 424.2020, found 424.2018.

#### 1-(3-(9H-carbazol-9-yl)propyl)-3-(2-(naphthalen-2-yl)-2-oxoethyl)-1H-imidazol-3-ium bromide (**16**)

Yield 94%. White powder, m.p. 136–137 °C. IR *ν*_max_ (cm^−1^): 3392, 3145, 3047, 2966, 1687, 1630, 1560, 1456, 1336, 1224, 1166, 1034, 935, 818, 753, 620. ^1^H NMR (300 MHz, MeOH) δ: 8.87 (1H, s), 8.67 (1H, s), 8.07 (3H, d, J = 6.0 Hz ), 8.00–7.89 (3H, m), 7.67–7.62 (2H, m), 7.59–7.23 (6H, m), 7.20 (2H, t, J = 6.0 Hz), 5.95 (2H, s), 4.49 (2H, t, J = 6.0 Hz), 4.31 (2H, t, J = 6.0 Hz), 2.47 (2H, m). ^13^C NMR (75 MHz, MeOH) δ: 191.85, 141.44, 138.65, 137.62, 133.91, 132.35, 131.82, 130.93, 130.49, 130.04, 128.97, 128.40, 127.12, 125.41, 124.25, 122.91, 120.39, 109.99, 56.50, 40.75, 29.91. HRMS (ESI-TOF) m/z Calcd for C_30_H_26_N_3_O [M-Br]^+^ 444.2070, found 444.2072.

#### 1-(3-(9H-carbazol-9-yl)propyl)-3-(2-(4-bromophenyl)-2-oxoethyl)-1H-imidazol-3-ium bromide (**17**)

Yield 95%. White powder, m.p. 105–107 °C. IR *ν*_max_ (cm^−1^): 3407, 3129, 3053, 2959, 1697, 1582, 1454, 1335, 1228, 1164, 1068, 994, 820, 755, 621. ^1^H NMR (300 MHz, CDCl_3_) δ: 8.78 (1H, s), 8.03 (2H, d, J = 6.0 Hz), 7.85 (2H, d, J = 6.0 Hz), 7.65 (2H, d, J = 6.0 Hz), 7.49–7.40 (6H, m), 7.18 (2H, t, J = 6.0 Hz), 5.72 (2H, s), 4.43 (2H, t, J = 6.0 Hz), 4,26 (2H, t, J = 6.0 Hz), 2.42–2.40 (2H, m). ^13^C NMR (75 MHz, CDCl_3_) δ: 191.11, 141.32, 138.41, 133.82, 133.52, 131.11, 130.72, 127.21, 125.33, 124.22, 122.93, 121.40, 120.51, 110.09, 56.40, 49.11, 40.72, 30.03. HRMS (ESI-TOF) m/z Calcd for C_26_H_23_BrN_3_O [M-Br]^+^ 472.1024, found 472.1022.

#### 1-(3-(9H-carbazol-9-yl)propyl)-3-(4-bromobenzyl)-1H-imidazol-3-ium bromide (**18**)

Yield 80%. White powder, m.p. 64–66 °C. IR *ν*_max_ (cm^−1^): 3411, 3137, 3049, 2949, 1595, 1486, 1453, 1333, 1154, 1067, 1011, 809, 753, 613. ^1^H NMR (300 MHz, CDCl_3_) δ: 9.95 (1H, s), 7.96 (2H, d, J = 6.0 Hz), 7.38–7.29 (6H, m), 7.23 (2H, d, J = 6.0 Hz), 7.15 (2H, m), 7.05 (1H, s), 6.98 (1H, s), 5.27 (2H, s), 4.42 (2H, t, J = 6.0 Hz), 4.28 (2H, t, J = 6.0 Hz), 2.43–2.45 (2H, m). ^13^C NMR (75 MHz, CDCl_3_) δ: 139.80, 136.11, 132.42, 131.81, 130.74, 126.23, 123.72, 122.73, 121.93, 121.63, 120.31, 119.42, 108.92, 52.31, 47.92, 40.03, 29.03. HRMS (ESI-TOF) m/z Calcd for C_25_H_23_BrN_3_ [M-Br]^+^ 444.1070, found 444.1065.

#### 1-(3-(9H-carbazol-9-yl)propyl)-3-(4-methylbenzyl)-1H-imidazol-3-ium bromide (**19**)

Yield 85%. Yellow oil. IR *ν*_max_ (cm^−1^): 3403, 3129, 3051, 2964, 1599, 1562, 1454, 1334, 1230, 1155, 1054, 832, 755, 626. ^1^H NMR (300 MHz, CDCl_3_) δ: 9.96 (1H, s), 7.98 (2H, d, J = 9.0 Hz), 7.44–7.36 (4H, m), 7.19–7.14 (4H, m), 7.08–7.04 (3H, m), 6.92 (1H, s), 5.19 (2H, s), 4.48–4.46 (2H, m), 4.36–4.34 (2H, m), 2.50–2.48 (2H, m), 2.25 (3H, s). ^13^C NMR (75 MHz, CDCl_3_) δ: 139.81, 139.51, 136.12, 130.03, 129.52, 128.92, 126.11, 122.73, 121.81, 121.30, 119.32, 109.00, 53.02, 47.92, 40.02, 29.10, 21.11. HRMS (ESI-TOF) m/z Calcd for C_26_H_26_N_3_ [M-Br]^+^ 380.2127, found 380.2121.

#### 1-(3-(9H-carbazol-9-yl)propyl)-3-(2-oxo-2-phenylethyl)-1H-benzo[d]imidazol-3-ium bromide (**20**)

Yield 95%. White powder, m.p. 120–122 °C. IR *ν*_max_ (cm^−1^): 3425, 3049, 2965, 1695, 1600, 1565, 1482, 1453, 1339, 1229, 1049, 987, 754, 684. ^1^H NMR (300 MHz, DMSO) δ: 9.74 (1H, s), 8.16–8.11 (5H, m), 8.07–8.04 (1H, m), 7.19 (1H, t, J = 7.2 Hz), 7.69–7.64 (6H, m), 7.44 (2H, t, J = 7.5 Hz), 7.20 (2H, t, J = 7.5 Hz), 6.36 (2H, s), 4.76 (2H, t, J = 7.2 Hz), 4.61 (2H, t, J = 6.9 Hz), 2.47–2.44 (2H, m). ^13^C NMR (75 MHz, DMSO) δ: 191.68, 143.79, 140.21, 135.08, 134.15, 132.38, 131.11, 129.54, 128.90, 127.26, 127.03, 126.25, 122.65, 120.80, 119.45, 114.43, 114.04, 109.70, 53.68, 54.20, 28.58. HRMS (ESI-TOF) m/z Calcd for C_30_H_26_N_3_O [M-Br]^+^ 444.2076, found 444.2072.

#### 1-(3-(9H-carbazol-9-yl)propyl)-3-(2-(4-methoxyphenyl)-2-oxoethyl)-1H-benzo[d]imidazol-3-ium bromide (**21**)

Yield 95%. Yellow powder, m.p. 144–146 °C. IR *ν*_max_ (cm^−1^): 3431, 3378, 3035, 2933, 2847, 1683, 1600, 1565, 1454, 1331, 1239, 1175, 1024, 983, 837, 755. ^1^H NMR (300 MHz, DMSO) δ: 9.81 (1H, s), 8.16–8.05 (6H, m), 7.69–7.67 (4H, m), 7.44 (2H, t, J = 7.2 Hz), 7.23–7.16 (4H, m), 6.34 (2H, s), 4.80–4.78 (2H, m), 4.63–4.62 (2H, m), 3.89 (3H, s), 2.49–2.47 (2H, m). ^13^C NMR (75 MHz, DMSO) δ: 197.37, 164.18, 143.40, 139.73, 131.91, 130.85, 130.64, 126.70, 126.50, 125.72, 122.16, 120.39, 118.93, 114.30, 113.90, 113.56, 109.20, 55.78, 52.76, 44.67, 39.47, 28.12. HRMS (ESI-TOF) m/z Calcd for C_31_H_28_N_3_O_2_ [M-Br]^+^ 474.2182, found 474.2174.

#### 1-(3-(9H-carbazol-9-yl)propyl)-3-(2-(naphthalen-2-yl)-2-oxoethyl)-1H-benzo[d]imidazol-3-ium  bromide (**22**)

Yield 95%. Yellow powder, m.p. 161–163 °C. IR *ν*_max_ (cm^−1^): 3429, 3129, 3048, 2952, 1688, 1625, 1564, 1454, 1339, 1221, 1187, 1008, 938, 862, 821, 753. ^1^H NMR (300 MHz, DMSO) δ: 9.87 (1H, s), 8.96 (1H, s), 8.24 (1H, d, J = 7.5 Hz), 8.17–8.12 (5H, m), 8.08–8.06 (2H, m), 7.77–7.68 (6H, m), 7.45 (2H, d, J = 7.5 Hz), 7.21 (2H, d, J = 7.5 Hz), 6.55 (2H, s), 4.81 (2H, t, J = 6.9 Hz), 4.64 (2H, t, J = 6.9 Hz), 2.49–2.48 (2H, m). ^13^C NMR (75 MHz, DMSO) δ: 191.11, 143.42, 139.74, 135.55, 131.95, 130.97, 130.68, 129.70, 129.36, 128.68, 127.85, 127.37, 126.77, 126.54, 125.75, 123.29, 122.17, 120.32, 118.95, 113.97, 113.60, 109.21, 53.23, 44.71, 39.47, 28.14. HRMS (ESI-TOF) m/z Calcd for C_34_H_28_N_3_O [M-Br]^+^ 494.2232, found 494.2227.

#### 1-(3-(9H-carbazol-9-yl)propyl)-3-(4-bromobenzyl)-1H-benzo[d]imidazol-3-ium bromide (**23**)

Yield 95%. Yellow powder, m.p. 222–224 °C. IR *ν*_max_ (cm^−1^): 3425, 3031, 2953, 1600, 1563, 1485, 1453, 1335, 1225, 1069, 806, 750. ^1^H NMR (300 MHz, DMSO) δ: 9.96 (1H, s), 8.15 (2H, d, J = 7.8 Hz), 8.11–8.08 (1H, m), 7.92–7.89 (1H, m), 7.70 (2H, d, J = 8.1 Hz), 7.65–7.57 (4H, m), 7.49–7.23 (4H, m), 7.21 (2H, t, J = 7.5 Hz), 5.71 (2H, s), 4.66–4.64 (4H, m), 2.49–2.47 (2H, m). ^13^C NMR (75 MHz, DMSO) δ: 142.25, 139.77, 133.28, 131.76, 131.24, 130.57, 126.60, 126.50, 125.70, 122.14, 121.99, 120.29, 118.91, 113.72, 109.29, 49.13, 44.78, 39.51, 28.02. HRMS (ESI-TOF) m/z Calcd for C_29_H_25_ BrN_3_ [M-Br]^+^ 494.1232, found 494.1226.

#### 1-(3-(9H-carbazol-9-yl)propyl)-3-(4-methylbenzyl)-1H-benzo[d]imidazol-3-ium bromide (**24**)

Yield 85%. White powder, m.p. 201–203 °C. IR *ν*_max_ (cm^−1^): 3411, 3117, 3025, 2956, 1606, 1564, 1453, 1336, 1224, 1143, 1028, 931, 752. ^1^H NMR (300 MHz, DMSO) δ: 9.87 (1H, s), 8.15 (2H, d, J = 7.8 Hz), 8.05 (1H, d, J = 4.8 Hz), 7.89 (1H, t, J = 5.0 Hz), 7.68–7.61 (4H, m), 7.47–7.37 (4H, m), 7.23–7.17 (4H, m), 5.63 (2H, s), 4.64–4.62 (4H, m), 2.49–2.48 (2H, m), 2.64 (3H, s). ^13^C NMR (75 MHz, DMSO) δ: 142.30, 139.76, 138.09, 131.24, 130.82, 129.41, 128.26, 126.54, 125.70, 122.15, 120.31, 118.93, 113.63, 109.21, 49.68, 44.73, 39.49, 27.99, 20.65. HRMS (ESI-TOF) m/z Calcd for C_30_H_28_N_3_ [M-Br]^+^ 430.2283, found 430.2278.

#### 1-(3-(9H-carbazol-9-yl)propyl)-3-(2-bromobenzyl)-1H-benzo[d]imidazol-3-ium bromide (**25**)

Yield 75%. Yellow powder, m.p. 119–121 °C. IR *ν*_max_ (cm^−1^): 3129, 3045, 2937, 1707, 1600, 1562, 1452, 1336, 1223, 1147, 1027, 753, 609. ^1^H NMR (300 MHz, CDCl_3_) δ: 9.91 (1H, s), 8.24 (1H, s), 8.15–8.12 (3H, m), 7.87–7.85 (1H, m), 7.72–7.69 (3H, m), 7.64–7.62 (2H, m), 7.46–7.33 (5H, m), 7.20 (2H, t, J = 7.5 Hz), 5.78 (2H, s), 4.75–4.65 (4H, m), 2.48–2.47 (2H, m). ^13^C NMR (75 MHz, CDCl_3_) δ: 142.96, 139.74, 133.17, 132.54, 131.09, 130.94, 130.64, 128.36, 126.81, 126.60, 125.70, 122.96, 122.13, 120.28, 118.91, 113.90, 113.62, 109.30, 50.33, 44.86, 39.77, 28.14. HRMS (ESI-TOF) m/z Calcd for C_29_H_25_BrN_3_ [M-Br]^+^ 494.1232, found 494.1228.

#### 1-(3-(9H-carbazol-9-yl)propyl)-5,6-dimethyl-3-(2-(naphthalen-2-yl)-2-oxoethyl)-1H-benzo[d] imidazol-3-ium bromide (**26**)

Yield 95%. White powder, m.p. 159–161 °C. IR *ν*_max_ (cm^−1^): 3129, 3045, 2960, 1688, 1625, 1564, 1454, 1335, 1220, 1128, 1013, 935, 829, 754, 683. ^1^H NMR (300 MHz, DMSO) δ: 9.70 (1H, s), 8.96 (1H, s), 8.24 (1H, d, J = 7.92 Hz), 8.17–8.13 (3H, m), 8.08–8.06 (2H, m), 7.90 (1H, s), 7.78–7.69 (5H, m), 7.47 (2H, t, J = 7.4 Hz), 7.22 (2H, t, J = 7.4 Hz), 6.47 (2H, s), 4.71 (2H, t, J = 7.1 Hz), 4.64 (2H, t, J = 6.8 Hz), 2.48–2.46 (2H, m), 2.36–2.35 (6H, m). ^13^C NMR (75 MHz, DMSO) δ: 191.63, 142.64, 140.24, 136.93, 136.03, 132.50, 131.49, 131.42, 130.91, 130.19, 129.84, 129.56, 129.17, 128.35, 127.86, 126.24, 123.79, 122.67, 120.82, 119.44, 113.88, 113.45, 109.74, 53.60, 45.04, 28.67, 20.43. HRMS (ESI-TOF) m/z Calcd for C_36_H_32_N_3_O [M-Br]^+^ 522.2540, found 522.2541.

#### 1-(3-(9H-carbazol-9-yl)propyl)-3-(2-(4-methoxyphenyl)-2-oxoethyl)-5,6-dimethyl-1H-benzo[d] imidazol-3-ium bromide (**27**)

Yield 94%. White powder, m.p. 176–179 °C. IR *ν*_max_ (cm^−1^): 3128, 3015, 2966, 1682, 1601, 1566, 1454, 1337, 1240, 1181, 1020, 956, 840, 752, 690, 603. ^1^H NMR (400 MHz, DMSO) δ: 9.67 (1H, s), 8.16–8.09 (4H, m), 7.82 (1H, s), 7.77 (1H, s), 7.68 (2H, d, J = 8.2 Hz), 7.45 (2H, t, J = 7.4 Hz), 7.23–7.17 (4H, m), 6.26 (2H, s), 4.69 (2H, t, J = 7.2 Hz), 4.62 (2H, t, J = 6.8 Hz), 3.90 (3H, s), 2.48–2.46 (2H, m), 2.36–2.35 (6H, m). ^13^C NMR (100 MHz, DMSO) δ: 189.91, 164.66, 142.60, 140.22, 136.86, 136.65, 131.35, 130.88, 129.52, 127.02, 126.21, 122.64, 120.80, 119.42, 114.78, 113.80, 113.42, 109.74, 56.28, 53.15, 44.99, 39.99, 28.65, 20.41. HRMS (ESI-TOF) m/z Calcd for C_33_H_32_N_3_O_2_ [M-Br]^+^ 502.2489, found 502.2492.

#### 1-(3-(9H-carbazol-9-yl)propyl)-5,6-dimethyl-3-(4-methylbenzyl)-1H-benzo[d]imidazol-3-ium  bromide (**28**)

Yield 85%. White powder, m.p. 169–171 °C. IR *ν*_max_ (cm^−1^): 3124, 3023, 2961, 1600, 1563, 1453, 1336, 1221, 1126, 1014, 845, 755, 673. ^1^H NMR (400 MHz, DMSO) δ: 9.73 (1H, s), 8.15–8.13 (2H, m), 7.69–7.67 (4H, m), 7.46–7.44 (2H, m), 7.35–7.33 (2H, m), 7.20–7.17 (4H, m), 5.55 (2H, s), 4.62–4.55 (4H, m), 2.35–2.30 (6H, m), 2.26–2.25 (2H, m), 2.08 (3H, s). ^13^C NMR (100 MHz, DMSO) δ: 141.51, 140.24, 138.50, 136.69, 131.54, 130.07, 129.90, 129.68, 128.61, 126.19, 122.62, 120.80, 119.42, 113.55, 109.75, 49.92, 45.09, 39.99, 31.16, 28.48, 21.15, 20.43. HRMS (ESI-TOF) m/z Calcd for C_32_H_32_N_3_ [M-Br]^+^ 458.2596, found 458.2591.

#### 1-(4-(9H-fluoren-9-yl)butyl)-3-(2-(naphthalen-2-yl)-2-oxoethyl)-1H-imidazol-3-ium bromide (**29**)

Yield 95%. White powder, m.p. 107–109 °C. IR *ν*_max_ (cm^−1^): 3051, 2943, 2859, 1692, 1625, 1593, 1564, 1455, 1335, 1226, 1166, 1029, 939, 861, 753, 626. ^1^H NMR (400 MHz, DMSO) δ: 9.29 (1H, s), 8.86 (1H, s), 8.22–8.11 (4H, m), 8.7–8.02 (2H, m), 7.92 (1H, s), 7.83 (1H, s), 7.75–7.65 (4H, m), 7.47 (2H, t, J = 7.4 Hz), 7.21 (2H, t, J = 7.4 Hz), 6.27 (2H, s), 4.48 (2H, t, J = 6.7 Hz), 4.37 (2H, t, J = 6.7 Hz), 1.95–1.92 (2H, m), 1.82–1.79 (2H, m). ^13^C NMR (100 MHz, DMSO) δ: 191.74, 140.38, 137.82, 135.99, 132.51, 131.43, 131.06, 130.17, 129.81, 129.25, 128.35, 127.86, 126.23, 124.71, 123.65, 122.56, 122.51, 120.80, 119.26, 109.78, 56.02, 49.13, 42.10, 27.68, 25.69. HRMS (ESI-TOF) m/z Calcd for C_31_H_28_N_3_O [M-Br]^+^ 457.2227, found 457.2226.

#### 1-(4-(9H-carbazol-9-yl)butyl)-3-(2-(4-methoxyphenyl)-2-oxoethyl)-1H-imidazol-3-ium bromide (**30**)

Yield 96%. White powder, m.p. 90–92 °C. IR *ν*_max_ (cm^−1^): 3054, 2937, 2835, 1688, 1599, 1564, 1499, 1455, 1340, 1240, 1169, 1026, 935, 835, 757, 627. ^1^H NMR (400 MHz, DMSO) δ: 9.22 (1H, s), 8.16 (2H, d, J = 7.7 Hz), 8.03 (2H, d, J = 8.6 Hz), 7.88 (1H, s), 7.76 (1H, s), 7.65 (2H, d, J = 8.2 Hz), 7.47 (2H, t, J = 7.4 Hz), 7.21(2H, t, J = 7.4 Hz), 7.15 (2H, d, J = 8.6 Hz), 6.05 (2H, s), 4.48 (2H, t, J = 6.7 Hz), 4.33 (2H, t, J = 6.7 Hz), 3.41 (3H, s), 1.93–1.90 (2H, m), 1.80–1.77 (2H, m). ^13^C NMR (100 MHz, DMSO) δ: 190.03, 164.56, 140.37, 137.77, 131.07, 126.94, 126.21, 124.67, 122.54, 122.39, 120.79, 119.24, 114.82, 109.77, 56.26, 55.56, 49.08, 42.08, 27.67, 25.67. HRMS (ESI-TOF) m/z Calcd for C_28_H_28_N_3_O_2_ [M-Br]^+^ 438.2176, found 438.2177.

#### 1-(4-(9H-carbazol-9-yl)butyl)-3-(2-(4-bromophenyl)-2-oxoethyl)-1H-imidazol-3-ium bromide (**31**)

Yield 95%. White powder, m.p. 153–155 °C. IR *ν*_max_ (cm^−1^): 3141, 3049, 2933, 2851, 1698, 1582, 1455, 1389, 1230, 1166, 1069, 993, 823, 755, 622. ^1^H NMR (400 MHz, DMSO) δ: 9.25–9.22 (1H, m), 8.16 (2H, d, J = 7.7 Hz), 7.98 (2H, d, J = 7.9 Hz), 7.89–7.84 (3H, m), 7.77 (1H, s), 7.65 (2H, d, J = 8.2 Hz), 7.46 (2H, t, J = 7.5 Hz), 7.20 (2H, t, J = 7.4 Hz), 6.10 (2H, s), 4.47 (2H, t, J = 6.7 Hz), 4.34 (2H, t, J = 6.7 Hz), 1.92–1.90 (2H, m), 1.80–1.77 (2H, m). ^13^C NMR (100 MHz, DMSO) δ: 191.21, 140.36, 137.73, 133.18, 132.66, 130.60, 129.11, 126.20, 124.62, 122.54, 122.48, 120.79, 119.24, 109.76, 55.93, 49.11, 42.09, 27.67, 25.67. HRMS (ESI-TOF) m/z Calcd for C_27_H_25_BrN_3_O [M-Br]^+^ 486.1181, found 486.1176.

#### 1-(4-(9H-carbazol-9-yl)butyl)-3-(2-oxo-2-phenylethyl)-1H-imidazol-3-ium bromide (**32**)

Yield 94%. White powder, m.p.96–97 °C. IR *ν*_max_ (cm^−1^): 3133, 3054, 2942, 2859, 1903, 1696, 1593, 1565, 1453, 1337, 1231, 1165, 1119, 990, 818, 756, 686. ^1^H NMR (400 MHz, DMSO) δ: 9.21 (1H, s), 8.16 (2H, d, J = 7.7 Hz), 8.05 (2H, d, J = 7.6 Hz), 7.88 (1H, s), 7.78–7.74 (2H, m), 7.66–7.61 (4H, m), 7.46 (2H, t, J = 7.5 Hz), 7.21 (2H, t, J = 7.4 Hz), 6.11 (2H, s), 4.48 (2H, t, J = 6.7 Hz), 4.34 (2H, t, J = 6.7 Hz), 1.93–1.90 (2H, m), 1.80–1.77 (2H, m). ^13^C NMR (100 MHz, DMSO) δ: 191.81, 140.37, 137.75, 134.99, 134.12, 129.57, 128.63, 126.21, 124.66, 122.54, 122.46, 120.80, 119.24, 109.76, 55.94, 49.10, 42.08, 27.67, 25.67. HRMS (ESI-TOF) m/z Calcd for C_27_H_26_N_3_O [M-Br]^+^ 408.2076, found 408.2072.

#### 1-(4-(9H-carbazol-9-yl)butyl)-3-(4-methylbenzyl)-1H-imidazol-3-ium bromide (**33**)

Yield 85%. Yellow powder, m.p. 174–176 °C. IR *ν*_max_ (cm^−1^): 3133, 2948, 2864, 1598, 1558, 1453, 1334, 1231, 1153, 1027, 826, 754, 622. ^1^H NMR (400 MHz, DMSO) δ: 9.42 (1H, s), 8.15 (2H, d, J = 7.7 Hz), 7.81 (2H, d, J = 7.9 Hz), 7.62 (2H, d, J = 8.2 Hz), 7.44 (2H, t, J = 7.4 Hz), 7.29 (2H, d, J = 7.8 Hz), 7.22–7.14 (4H, m), 5.39 (2H, s), 4.44 (2H, t, J = 6.7 Hz), 4.23 (2H, t, J = 6.7 Hz), 2.27 (3H, s), 1.90–1.86 (2H, m), 1.74–1.72 (2H, m). ^13^C NMR (100 MHz, DMSO) δ: 140.33, 138.60, 136.47, 132.44, 129.95, 128.76, 126.18, 123.14, 123.00, 122.53, 120.79, 119.22, 109.71, 52.10, 49.09, 42.06, 27.43, 25.66, 21.18. HRMS (ESI-TOF) m/z Calcd for C_27_H_28_N_3_ [M-Br]^+^ 394.2278, found 394.2274.

#### 1-(4-(9H-carbazol-9-yl)butyl)-3-(2-bromobenzyl)-1H-imidazol-3-ium bromide (**34**)

Yield 80%. Yellow powder, m.p.157–159 °C. IR *ν*_max_ (cm^−1^): 3099, 2955, 2851, 1594, 1559, 1453, 1336, 1226, 1160, 1057, 880, 739, 648. ^1^H NMR (400 MHz, DMSO) δ: 9.36 (1H, s), 8.15 (2H, d, J = 7.7 Hz), 7.87 (1H, s), 7.77 (1H, s), 7.68 (1H, d, J = 7.8 Hz), 7.62 (2H, d, J = 8.2 Hz), 7.46–7.36 (5H, m), 7.20 (2H, t, J = 7.4 Hz), 5.51 (2H, s), 4.46 (2H, t, J = 6.7 Hz), 4.27 (2H, t, J = 6.7 Hz), 1.90–1.87 (2H, m), 1.74–1.70 (2H, m). ^13^C NMR (100 MHz, DMSO) δ: 140.33, 137.17, 133.96, 133.61, 131.52, 131.39, 128.96, 126.17, 123.62, 123.31, 123.25, 122.53, 120.79, 119.22, 109.72, 52.70, 49.14, 42.06, 27.56, 25.65. HRMS (ESI-TOF) m/z Calcd for C_26_H_26_BrN_3_ [M-Br]^+^ 458.1232, found 458.1226.

#### 1-(4-(9H-carbazol-9-yl)butyl)-3-(2-(naphthalen-2-yl)-2-oxoethyl)-1H-benzo[d]imidazol-3-ium  bromide (**35**)

Yield 95%. White powder, m.p. 239–241 °C. IR *ν*_max_ (cm^−1^): 3109, 3020, 2947, 2884, 1795, 1682, 1624, 1557, 1455, 1338, 1255, 1124, 1078, 933, 818, 753, 678. ^1^H NMR (400 MHz, DMSO) δ: 9.81 (1H, s), 8.94 (1H, s), 8.24 (1H, d, J = 7.5 Hz), 8.16–8.04 (7H, m), 7.76–7.70 (4H, m), 7.65 (2H, t, J = 7.6 Hz), 7.45 (2H, t, J = 6.9 Hz), 7.20 (2H, t, J = 6.9 Hz), 6.55 (2H, s), 4.68–4.66 (2H, m), 4.51–4.49 (2H, m), 2.05–2.03 (2H, m), 1.92–1.90 (2H, m). ^13^C NMR (100 MHz, DMSO) δ: 191.62, 143.84, 140.37, 136.05, 132.49, 131.44, 131.13, 130.20, 129.88, 129.19, 128.38, 127.90, 127.29, 127.08, 126.18, 123.81, 122.53, 120.78, 119.23, 114.50, 114.22, 109.79, 53.73, 47.11, 42.13, 26.76, 25.93. HRMS (ESI-TOF) m/z Calcd for C_35_H_30_N_3_O [M-Br]^+^ 508.2383, found 508.2385.

#### 1-(4-(9H-carbazol-9-yl)butyl)-3-(2-(4-methoxyphenyl)-2-oxoethyl)-1H-benzo[d]imidazol-3-ium  bromide (**36**)

Yield 95%. White powder, m.p. 182–184 °C. IR *ν*_max_ (cm^−1^): 3028, 2930, 2839, 1796, 1678, 1600, 1560, 1453, 1334, 1238, 1175, 1025, 983, 832, 754. ^1^H NMR (300 MHz, DMSO) δ: 9.93 (1H, s), 8.16–8.13 (3H, m), 8.11–8.09 (3H, m), 7.66–7.64 (4H, m), 7.44 (2H, t, J = 7.5 Hz), 7.21–7.16 (4H, m), 6.44 (2H, s), 4.66–4.63 (2H, m), 4.50–4.46 (2H, m), 3.89 (3H, s), 2.04–2.01 (2H, m), 1.92–1.90 (2H, m). ^13^C NMR (75 MHz, DMSO) δ: 189.94, 164.66, 143.76, 140.37, 132.43, 131.43, 131.07, 127.18, 127.01, 126.19, 122.53, 120.77, 119.23, 114.78, 114.48, 114.18, 109.83, 56.31, 53.46, 47.06, 42.15, 26.73, 25.89. HRMS (ESI-TOF) m/z Calcd for C_32_H_30_N_3_O_2_ [M-Br]^+^ 488.2338, found 488.2332.

#### 1-(4-(9H-carbazol-9-yl)butyl)-3-(2-(4-bromophenyl)-2-oxoethyl)-1H-benzo[d]imidazol-3-ium  bromide (**37**)

Yield 95%. White powder, m.p. 237–239 °C. IR *ν*_max_ (cm^−1^): 3021, 2931, 2876, 1795, 1688, 1580, 1552, 1452, 1386, 1221, 1169, 1070, 984, 822, 753, 618. ^1^H NMR (300 MHz, DMSO) δ: 9.80 (1H, s), 8.16–8.05 (6H, m), 7.89 (2H, d, J = 8.1 Hz), 7.69–7.64 (4H, m), 7.44 (2H, t, J = 7.5 Hz), 7.19 (2H, t, J = 7.3 Hz), 6.43 (2H, s), 4.67–4.64 (2H, m), 4.50–4.47 (2H, m), 2.03–2.01 (2H, m), 1.90–1.88 (2H, m). ^13^C NMR (75 MHz, DMSO): δ 191.12, 143.71, 140.36, 133.24, 132.61, 132.41, 131.08, 130.89, 129.20, 127.24, 127.06, 126.17, 122.52, 120.77, 119.22, 114.54, 114.20, 109.80, 53.76, 47.09, 42.13, 26.75, 25.91. HRMS (ESI-TOF) m/z Calcd for C_31_H_27_BrN_3_O [M-Br]^+^ 536.1338, found 536.1330.

#### 1-(4-(9H-carbazol-9-yl)butyl)-3-(2-oxo-2-phenylethyl)-1H-benzo[d]imidazol-3-ium bromide (**38**)

Yield 95%. White powder, m.p. 179–181 °C. IR *ν*_max_ (cm^−1^): 3024, 2936, 1795, 1692, 1596, 1563, 1452, 1337, 1229, 1180, 1074, 930, 823, 754, 615. ^1^H NMR (300 MHz, DMSO) δ: 9.86 (1H, s), 8.16–8.11 (6H, m), 7.79 (1H, t, J = 7.2 Hz), 7.68–7.64 (6H, m), 7.45 (2H, t, J = 7.4 Hz), 7.20 (2H, t, J = 7.4 Hz), 6.47 (2H, s), 4.66 (2H, t, J = 6.7 Hz), 4.49 (2H, t, J = 6.7 Hz), 2.04–2.01 (2H, m), 1.91–1.90 (2H, m). ^13^C NMR (75 MHz, DMSO) δ: 191.74, 143.74, 140.37, 135.06, 134.19, 132.43, 131.09, 129.53, 128.95, 127.23, 127.04, 126.18, 122.52, 120.77, 119.22, 114.53, 114.20, 109.82, 53.81, 47.08, 42.14, 26.74, 25.90. HRMS (ESI-TOF) m/z Calcd for C_31_H_28_N_3_O [M-Br]^+^ 458.2232, found 458.2230.

#### 1-(4-(9H-carbazol-9-yl)butyl)-3-(4-methylbenzyl)-1H-benzo[d]imidazol-3-ium bromide (**39**)

Yield 95%. White powder, m.p. 196–198 °C. IR *ν*_max_ (cm^−1^): 3113, 3023, 1815, 1599, 1559, 1453, 1376, 1216, 1180, 1024, 754, 610. ^1^H NMR (400 MHz, DMSO) δ: 10.12 (1H, s), 8.14 (2H, d, J = 7.6 Hz), 8.05 (1H, t, J = 3.2 Hz), 7.96 (1H, t, J = 5.2 Hz), 7.66–7.60 (4H, m), 7.45–7.39 (4H, m), 7.19 (2H, t, J = 7.4 Hz), 7.13 (2H, t, J = 7.6 Hz), 5.73 (2H, s), 4.57–4.54 (2H, m), 4.49–4.46 (2H, m), 2.25 (3H, m), 2.02–2.01 (2H, m), 1.87–1.85 (2H, m). ^13^C NMR (100 MHz, DMSO) δ: 142.71, 140.34, 138.55, 131.69, 131.46, 131.20, 129.92, 128.96, 127.06, 126.15, 122.52, 120.77, 119.20, 114.41, 114.30, 109.79, 50.10, 47.03, 42.16, 26.57, 25.96, 21.17. HRMS (ESI-TOF) m/z Calcd for C_31_H_30_N_3_ [M-Br]^+^ 444.2440, found 444.2427.

#### 1-(4-(9H-carbazol-9-yl)butyl)-3-(2-bromobenzyl)-1H-benzo[d]imidazol-3-ium bromide (**40**)

Yield 95%. Yellow powder, m.p.100–102 °C. IR *ν*_max_ (cm^−1^): 3117, 3043, 2942, 1600, 1563, 1453, 1335, 1226, 1024, 753, 665, 615. ^1^H NMR (300 MHz, DMSO) δ: 9.93 (1H, s), 8.14 (2H, d, J = 7.7 Hz), 8.09 (1H, d, J = 8.1 Hz), 7.88 (1H, t, J = 7.9 Hz), 7.69 (1H, d, J = 7.7 Hz), 7.65–7.61 (4H, m), 7.44–7.35 (5H, m), 7.19 (2H, t, J = 7.4 Hz), 5.81 (2H, s), 4.59 (2H, t, J = 6.7 Hz), 4.47 (2H, t, J = 6.7 Hz), 2.02–1.98 (2H, m), 1.87–1.84 (2H, m). ^13^C NMR (100 MHz, DMSO) δ: 143.42, 140.34, 133.76, 133.00, 131.57, 131.49, 131.37, 128.89, 127.33, 127.18, 126.15, 123.59, 122.51, 120.77, 119.21, 114.45, 114.26, 109.77, 50.93, 47.08, 42.13, 26.76, 25.93. HRMS (ESI-TOF) m/z Calcd for C_30_H_27_BrN_3_ [M-Br]^+^ 508.1383, found 508.1382.

#### 1-(4-(9H-carbazol-9-yl)butyl)-5,6-dimethyl-3-(2-(naphthalen-2-yl)-2-oxoethyl)-1H-benzo[d]imidazol-3-ium bromide (**41**)

Yield 95%. White powder, m.p.249–251 °C. IR *ν*_max_ (cm^−1^): 3024, 2949, 1808, 1685, 1625, 1593, 1562, 1454, 1336, 1217, 1186, 1011, 933, 858, 753, 617. ^1^H NMR (300 MHz, DMSO) δ: 9.58 (1H, s), 8.86 (1H, s), 8.15 (1H, d, J = 7.9 Hz), 8.05 (3H, d, J = 8.0 Hz), 7.97 (2H, t, J = 10.0 Hz), 7.81 (1H, s), 7.76 (1H, s), 7.69–7.61 (2H, m), 7.55 (2H, d, J = 8.2 Hz), 7.36 (2H, t, J = 7.5 Hz), 7.11 (2H, t, J = 7.4 Hz), 6.40 (2H, s), 4.51 (2H, t, J = 6.3 Hz), 4.40 (2H, t, J = 6.6 Hz), 2.31 (3H, s), 2.27 (3H, s), 1.94–1.91 (2H, m), 1.82–1.80 (2H, m). ^13^C NMR (75 MHz, DMSO) δ: 191.63, 142.51, 140.35, 136.99, 136.78, 136.03, 132.50, 131.48, 131.42, 130.96, 130.19, 129.85, 129.52, 129.16, 128.37, 127.88, 126.16, 123.80, 122.51, 120.75, 119.21, 113.91, 113.56, 109.78, 53.62, 46.98, 42.09, 26.66, 25.83, 20.45. HRMS (ESI-TOF) m/z Calcd for C_37_H_34_N_3_O [M-Br]^+^ 536.2696, found 536.2697.

#### 1-(4-(9H-carbazol-9-yl)butyl)-3-(2-(4-methoxyphenyl)-2-oxoethyl)-5,6-dimethyl-1H-benzo[d]imidazol-3-ium bromide (**42**)

Yield 96%. White powder, m.p. 156–158 °C. IR *ν*_max_ (cm^−1^): 3051, 3015, 2936, 1683, 1599, 1565, 1454, 1336, 1239, 1176, 1016, 955, 838, 755, 601. ^1^H NMR (300 MHz, DMSO) δ: 9.62 (1H, s), 8.13 (2H, d, J = 7.7 Hz), 8.09 (2H, d, J = 8.5 Hz), 7.83–7,82 (2H, m), 7.63 (2H, d, J = 8.2 Hz), 7.43 (2H, t, J = 7.4 Hz), 7.21–7.17 (4H, m), 6.28 (2H, s), 4.57 (2H, t, J = 6.0 Hz), 4.47 (2H, t, J = 6.6 Hz), 3.90 (3H, s), 2.38 (3H, s), 2.34 (3H, s), 1.99–1.97 (2H, m), 1.88–1.87 (2H, m). ^13^C NMR (75 MHz, DMSO) δ: 189.91, 164.66, 142.49, 140.34, 136.92, 136.72, 131.35, 130.92, 129.48, 127.01, 126.14, 122.50, 120.73, 119.19, 114.77, 113.83, 113.52, 109.77, 56.29, 53.16, 46.92, 42.07, 26.65, 25.80, 20.42. HRMS (ESI-TOF) m/z Calcd for C_34_H_34_N_3_O_2_ [M-Br]^+^ 516.2646, found 516.2648.

#### 1-(4-(9H-carbazol-9-yl)butyl)-3-(2-(4-bromophenyl)-2-oxoethyl)-5,6-dimethyl-1H-benzo[d]imidazol-3-ium bromide (**43**)

Yield 94%. White powder, m.p. 230–232 °C. IR *ν*_max_ (cm^−1^): 3015, 2934, 1694, 1582, 1454, 1336, 1229, 1180, 1071, 957, 819, 753, 611. ^1^H NMR (300 MHz, DMSO) δ: 8.03–7.95 (4H, m), 7.78–7.64 (3H, m), 7.44–7.37 (8H, m), 7.32–7.24 (2H, m), 7.09–7.07 (2H, m), 6.01 (2H, s), 3.81 (3H, s), 2.52 (3H, s). ^13^C NMR (75 MHz, DMSO) δ: 191.18,142.48, 140.40, 137.03, 136.84, 133.31, 132.67, 130.92, 129.55, 129.23, 126.21, 122.57, 120.81, 119.27, 114.00, 113.63, 109.84, 53.65, 47.03, 46.13, 42.14, 26.72, 25.88, 20.53. HRMS (ESI-TOF) m/z Calcd for C_33_H_31_ BrN_3_ [M-Br]^+^ 564.1645, found 564.1638.

#### 1-(4-(9H-carbazol-9-yl)butyl)-5,6-dimethyl-3-(2-oxo-2-phenylethyl)-1H-benzo[d]imidazol-3-ium bromide (**44**)

Yield 90%. White powder, m.p. 152–153 °C. IR *ν*_max_ (cm^−1^): 3121, 3043, 2936, 1694, 1599, 1564, 1453, 1337, 1230, 1187, 1001, 955, 848, 755, 612. ^1^H NMR (300 MHz, DMSO) δ: 9.63 (1H, s), 8.13 (4H, t, J = 7.2 Hz), 7.85 (2H, d, J = 4.9 Hz), 7.80–7.78 (1H, m), 7.68–7.62 (4H, m), 7.44 (2H, t, J = 7.6 Hz), 7.19 (2H, t, J = 7.4 Hz), 6.36 (2H, s), 4.59 (2H, t, J = 6.6 Hz), 4.48 (2H, t, J = 6.8 Hz), 2.39 (3H, s), 2.35 (3H, s), 2.01–1.98 (2H, m), 1.89–1.87 (2H, m). ^13^C NMR (75 MHz, DMSO) δ: 191.71, 142.43, 140.34, 136.95, 136.75, 135.04, 134.18, 130.91, 129.51, 128.90, 126.14, 122.50, 120.74, 119.19, 113.91, 113.54, 109.77, 53.60, 46.95, 42.08, 26.66, 25.81, 20.45. HRMS (ESI-TOF) m/z Calcd for C_33_H_32_N_3_O [M-Br]^+^ 486.2545, found 486.2535.

#### 1-(4-(9H-carbazol-9-yl)butyl)-3-(2-bromobenzyl)-5,6-dimethyl-1H-benzo[d]imidazol-3-ium bromide (**45**)

Yield 85%. Yellow powder, m.p. 129–131 °C. IR *ν*_max_ (cm^−1^): 3137, 3047, 2939, 1600, 1562, 1453, 1337, 1228, 1184, 1021, 947, 844, 755, 608. ^1^H NMR (300 MHz, DMSO) δ: 9.69 (1H, s), 8.12 (2H, d, J = 7.7 Hz), 7.82 (1H, s), 7.70 (1H, d, J = 7.5 Hz), 7.66 (1H, s), 7.60 (2H, d, J = 8.2 Hz), 7.43–7.37 (4H, m), 7.34–7.32 (1H, m), 7.18 (2H, t, J = 7.5 Hz), 5.71 (2H, s), 4.50 (2H, t, J = 6.6 Hz), 4.45 (2H, t, J = 6.8 Hz), 2.36 (3H, s), 2.34 (3H, s), 1.98–1.95 (2H, m), 1.84–1.82 (2H, m). ^13^C NMR (75 MHz, DMSO) δ: 142.03, 140.31, 137.06, 136.99, 133.72, 133.20, 131.38, 130.97, 130.00, 129.91, 128.88, 126.11, 123.44, 122.49, 120.73, 119.17, 113.80, 113.62, 109.73, 50.71, 46.96, 42.06, 26.64, 25.82, 20.49, 20.45. HRMS (ESI-TOF) m/z Calcd for C_32_H_32_BrN_3_ [M-Br]^+^ 536.1701, found 536.1698.

#### 1-(4-(9H-carbazol-9-yl)butyl)-5,6-dimethyl-3-(4-methylbenzyl)-1H-benzo[d]imidazol-3-ium bromide (**46**)

Yield 86%. White powder, m.p. 129–131 °C. IR *ν*_max_ (cm^−1^): 3128, 3041, 2936, 1599, 1559, 1454, 1337, 1228, 1183, 1012, 944, 840, 755, 609. ^1^H NMR (300 MHz, CDCl_3_) δ: 9.87 (1H, s), 8.13 (2H, d, J = 7.7 Hz), 7.76 (1H, s), 7.73 (1H, s), 7.61 (2H, d, J = 8.2 Hz), 7.42 (2H, t, J = 7.5 Hz), 7.35–7.32 (2H, m), 7.18 (2H, t, J = 7.5 Hz), 7.12 (2H, d, J = 7.7 Hz), 5.62 (2H, s), 4.50–4.44 (4H, m), 2.34 (6H, s), 2.25 (3H, s), 2.00–1.97 (2H, m), 1.84–1.81 (2H, m). ^13^C NMR (75 MHz, CDCl_3_) δ: 141.34, 140.29, 138.47, 136.84, 132.90, 131.62, 130.01, 129.91, 129.70, 128.54, 126.12, 122.48, 120.73, 119.18, 113.72, 113.65, 109.72, 52.32, 49.86, 46.91, 40.17, 26.46, 25.84, 21.15, 20.47, 20.43. HRMS (ESI-TOF) m/z Calcd for C_34_H_34_N_3_ [M-Br]^+^ 472.2747, found 472.2742.

#### 1-(5-(9H-carbazol-9-yl)pentyl)-3-(2-(4-methoxyphenyl)-2-oxoethyl)-1H-imidazol-3-ium  bromide (**47**)

Yield 95%. Yellow oil. IR *ν*_max_ (cm^−1^): 3137, 3054, 2936, 1685, 1599, 1454, 1335, 1241, 1167, 1023, 983, 835, 755, 628. ^1^H NMR (300 MHz, MeOH) δ: 8.80 (1H, s), 8.02 (2H, d, J = 7.8 Hz), 7.95 (2H, d, J = 8.7 Hz), 7.45 (1H, s), 7.42–7.35 (5H, m), 7.17–7.12 (2H, 3), 6.98 (2H, d, J = 8.7 Hz), 5.75 (2H, s), 4.21 (2H, t, J = 13.5 Hz), 3.92 (2H, t, J = 7.2 Hz), 3.77 (3H, s), 1.78–1.70 (2H, m), 1.67–1.59 (2H, m), 1.19–1.18 (2H, m). ^13^C NMR (75 MHz, MeOH) δ: 190.06, 166.10, 141.57, 138.29, 131.75, 127.64, 126.85, 125.22, 123.85, 122.83, 121.22, 119.95, 115.30, 110.13, 56.27, 56.03, 50.46, 43.29, 30.53, 29.18, 24.58. HRMS (ESI-TOF) m/z Calcd for C_29_H_30_N_3_O_2_ [M-Br]^+^ 452.2333, found 452.2327.

#### 1-(5-(9H-carbazol-9-yl)pentyl)-3-(2-(naphthalen-2-yl)-2-oxoethyl)-1H-imidazol-3-ium  bromide (**48**)

Yield 95%. White powder, m.p. 116–118 °C. IR *ν*_max_ (cm^−1^): 3137, 3051, 2939, 1693, 1625, 1564, 1455, 1335, 1223, 1166, 98, 822, 753, 628. ^1^H NMR (300 MHz, DMSO) δ: 9.26 (1H, s), 8.87 (1H, s), 8.21–8.06 (6H, m), 7.88–7.81 (2H, m), 7.72–7.62 (4H, m), 7.47–7.45 (2H, m), 7.21–7.20 (2H, m), 6.26 (2H, s), 4.42–4.26 (4H, m), 1.86–1.84 (4H, m), 1.36–1.34 (2H, m). ^13^C NMR (75 MHz, DMSO) δ: 191.76, 140.42, 137.76, 136.01, 132.52, 131.45, 131.06, 130.17, 129.82, 129.27, 128.36, 127.87, 126.17, 124.60, 123.66, 122.52, 120.76, 119.16, 109.75, 55.97, 49.22, 42.51, 29.75, 28.36, 23.58. HRMS (ESI-TOF) m/z Calcd for C_32_H_30_N_3_O [M-Br]^+^ 472.2383, found 472.2386.

#### 1-(5-(9H-carbazol-9-yl)pentyl)-3-(4-methylbenzyl)-1H-imidazol-3-ium bromide (**49**)

Yield 80%. Yellow oil. IR *ν*_max_ (cm^−1^): 3129, 3048, 2937, 1600, 1557, 1454, 1334, 1227, 1154, 1026, 831, 755, 627. ^1^H NMR (300 MHz, MeOH) δ: 8.89 (1H, s), 8.01 (2H, d, J = 7.8 Hz), 7.38–7.37 (5H, m), 7.27 (1H, s), 7.23–7.21 (2H, m), 7.17–7.11 (4H, m), 5.20 (2H, s), 4.20 (2H, t, J = 6.6 Hz), 3.87 (2H, t, J = 7.2 Hz), 2.27 (3H, s), 1.75–1.70 (2H, m), 1.64–1.59 (2H, m), 1.16–1.11 (2H, m). ^13^C NMR (75 MHz, MeOH) δ: 141.71, 140.50, 136.74, 132.15, 131.01, 129.79, 126.94, 123.97, 123.78, 123.51, 121.29, 120.06, 110.23, 53.87, 50.58, 43.33, 30.62, 29.29, 24.73, 21.35. HRMS (ESI-TOF) m/z Calcd for C_28_H_30_N_3_ [M-Br]^+^ 408.2434, found 408.2436.

#### 1-(5-(9H-carbazol-9-yl)pentyl)-3-(2-oxo-2-phenylethyl)-1H-benzo[d]imidazol-3-ium bromide (**50**)

Yield 90%. White powder, m.p. 225–227 °C. IR *ν*_max_ (cm^−1^): 3129, 3027, 2937, 1695, 1598, 1565, 1452, 1336, 1222, 1115, 987, 753, 690. ^1^H NMR (300 MHz, DMSO) δ: 9.89 (1H, s), 8.18–8.06 (6H, m), 7.80 (1H, t, J = 5.4 Hz), 7.70–7.66 (4H, m), 7.60 (2H, d, J = 6.2 Hz), 7.43 (2H, t, J = 5.6 Hz), 7.19 (2H, t, J = 5.6 Hz), 6.51 (2H, s), 4.56 (2H, t, J = 5.1 Hz), 4.40 (2H, t, J = 4.8 Hz), 1.95 (2H, t, J = 6.0 Hz), 1.86 (2H, d, J = 6.0 Hz), 1.45–1.43 (2H, m). ^13^C NMR (75 MHz, DMSO) δ: 191.78, 143.71, 140.41, 135.08, 134.22, 132.41, 131.13, 129.54, 128.96, 127.21, 127.02, 126.15, 122.51, 120.74, 119.14, 114.48, 114.21, 109.72, 53.80, 47.12, 42.56, 28.96, 28.46, 23.90. HRMS (ESI-TOF) m/z Calcd for C_32_H_30_N_3_O [M-Br]^+^ 472.2383, found 472.2383.

#### 1-(5-(9H-carbazol-9-yl)pentyl)-3-(2-(4-methoxyphenyl)-2-oxoethyl)-1H-benzo[d]imidazol-3-ium bromide (**51**)

Yield 94%. White powder, m.p. 131–133 °C. IR *ν*_max_ (cm^−1^): 3137, 3011, 2936, 2323, 1684, 1600, 1566, 1454, 1336, 1238, 1174, 1022, 984, 836, 755. ^1^H NMR (300 MHz, DMSO) δ: 9.86 (1H, s), 8.15–8.13 (4H, m), 8.08–8.04 (2H, m), 7.67–7.65 (2H, m), 7.60–7.58 (2H, m), 7.45–7.41 (2H, m), 7.20–7.21 (4H, m), 6.42 (2H, s), 4.45 (2H, t, J = 6.0 Hz), 4.39 (2H, d, J = 6.0 Hz), 3.90 (3H, s), 1.96–1.91 (2H, m), 1.86–1.81 (2H, m), 1.44–1.42 (2H, m). ^13^C NMR (75 MHz, DMSO) δ: 189.94, 164.70, 143.74, 140.40, 132.41, 131.11, 127.19, 127.02, 126.15, 122.50, 120.73, 119.14, 114.81, 114.40, 114.17, 109.70, 56.31, 53.34, 47.10, 42.55, 28.94, 28.44, 23.89. HRMS (ESI-TOF) m/z Calcd for C_33_H_32_N_3_O_2_ [M-Br]^+^ 502.2489, found 502.2489.

#### 1-(5-(9H-carbazol-9-yl)pentyl)-3-(2-(4-methoxyphenyl)-2-oxoethyl)-1H-benzo[d]imidazol-3-ium bromide (**52**)

Yield 90%. White powder, m.p. 120–122 °C. IR *ν*_max_ (cm^−1^): 3145, 3039, 2936, 2855, 1688, 1617, 1564, 1454, 1336, 1220, 1185, 997, 936, 821, 753. ^1^H NMR (300 MHz, DMSO) δ: 9.87 (1H, s), 8.99 (1H, s), 8.25 (1H, d, J = 7.8 Hz), 8.15 (4H, d, J = 6.9 Hz), 8.09 (3H, d, J = 7.7 Hz), 7.78–7.71 (2H, m), 7.70–7.67 (2H, m), 7.60 (2H, d, J = 8.2 Hz), 7.44 (2H, t, J = 7.4 Hz), 7.19 (2H, t, J = 7.4 Hz), 6.60 (2H, s), 4.58 (2H, t, J = 6.9 Hz), 4.42 (2H, t, J = 6.6 Hz), 1.98 (2H, t, J = 6.7 Hz), 1.87 (2H, t, J = 7.0 Hz), 1.46–1.45 (2H, m). ^13^C NMR (75 MHz, DMSO) δ: 191.67, 143.79, 140.42, 136.07, 132.52, 132.46, 131.51, 131.18, 130.21, 129.88, 129.20, 128.38, 127.90, 127.25, 127.06, 126.15, 123.82, 122.51, 120.75, 119.14, 114.46, 114.25, 109.71, 53.76, 47.16, 42.57, 28.98, 28.47, 23.93. HRMS (ESI-TOF) m/z Calcd for C_36_H_32_N_3_O [M-Br]^+^ 522.2540, found 522.2538.

#### 1-(5-(9H-carbazol-9-yl)pentyl)-3-(2-(4-bromophenyl)-2-oxoethyl)-1H-benzo[d]imidazol-3-ium  bromide (**53**)

Yield 94%. White powder, m.p. 187–189 °C. IR *ν*_max_ (cm^−1^): 3011, 2962, 2925, 1694, 1583, 1452, 1387, 1335, 1225, 1200, 1070, 985, 820, 750, 624. ^1^H NMR (400 MHz, DMSO) δ: 9.83 (1H, s), 8.15 (2H, d, J = 7.7 Hz), 8.10 (4H, d, J = 6.5 Hz), 7.91 (2H, d, J = 8.3 Hz), 7.69–7.66 (2H, m), 7.60 (2H, d, J = 8.2 Hz), 7.44 (2H, t, J = 7.4 Hz), 7.19 (2H, t, J = 7.4 Hz), 6.47 (2H, s), 4.57 (2H, t, J = 6.9 Hz), 4.41 (2H, t, J = 6.6 Hz), 1.98 (2H, t, J = 6.7 Hz), 1.87 (2H, t, J = 7.0 Hz), 1.46–1.45 (2H, m). ^13^C NMR (100 MHz, DMSO) δ: 191.16, 143.67, 140.41, 133.27, 132.63, 132.38, 131.13, 130.91, 129.22, 127.22, 127.04, 126.14, 122.50, 120.74, 119.13, 114.48, 114.22, 109.71, 53.75, 47.14, 42.56, 28.97, 28.47, 23.91. HRMS (ESI-TOF) m/z Calcd for C_32_H_29_BrN_3_O [M-Br]^+^ 550.1489, found 550.1484.

#### 1-(5-(9H-carbazol-9-yl)pentyl)-3-(4-methylbenzyl)-1H-benzo[d]imidazol-3-ium bromide (**54**)

Yield 90%. White powder, m.p. 193–195 °C. IR *ν*_max_ (cm^−1^): 3117, 3020, 2933, 2864, 2323, 1600, 1557, 1453, 1376, 1335, 1218, 1180, 1023, 844, 756.^1^H NMR (400 MHz, DMSO) δ: 10.08 (1H, s), 8.15 (2H, d, J = 7.7 Hz), 8.02–8.00 (1H, m), 7.98–7.96 (1H, m), 7.64–7.62 (2H, m), 7.60–7.57 (2H, m), 7.44–7.41 (4H, m), 7.21–7.17 (4H, m), 5.74 (2H, s), 4.46 (2H, t, J = 7.2 Hz), 4.40 (2H, t, J = 6.8 Hz), 2.28 (3H, s), 1.99–1.92 (2H, m), 1.88–1.81 (2H, m), 1.45–1.37 (2H, m). ^13^C NMR (100 MHz, DMSO) δ: 142.68, 140.40, 138.60, 131.74, 131.46, 131.19, 129.95, 128.82, 127.04, 126.12, 122.49, 120.73, 119.13, 114.35, 114.31, 109.69, 50.13, 47.09, 42.53, 28.89, 28.51, 23.99, 21.18. HRMS (ESI-TOF) m/z Calcd for C_32_H_32_N_3_ [M-Br]^+^ 458.2591, found 458.2592.

#### 1-(5-(9H-carbazol-9-yl)pentyl)-3-(2-bromobenzyl)-1H-benzo[d]imidazol-3-ium bromide (**55**)

Yield 90%. White powder, m.p. 171–173 °C. IR *ν*_max_ (cm^−1^): 3121, 3043, 3015, 2936, 2864, 2323, 1600, 1562, 1453, 1376, 1335, 1222, 1024, 754, 614. ^1^H NMR (400 MHz, MeOH) δ: 9.97 (1H, s), 8.15 (2H, d, J = 8.0 Hz), 8.08–8.06 (1H, m), 7.93–7.91 (1H, m), 7.75 (1H, d, J = 8.0 Hz), 7.67–7.65 (2H, m), 7.58 (2H, d, J = 8.0 Hz), 7.46–7.37 (5H, m), 7.19 (2H, t, J = 8.0 Hz), 6.60 (2H, s), 4.51 (2H, t, J = 6.9 Hz), 4.40 (2H, t, J = 6.6 Hz), 1.95 (2H, t, J = 6.7 Hz), 1.85 (2H, t, J = 7.0 Hz), 1.42–1.41 (2H, m). ^13^C NMR (100 MHz, MeOH) δ: 143.37, 140.39, 133.77, 133.03, 131.61, 131.51, 131.36, 128.92, 127.31, 127.18, 126.12, 123.61, 122.49, 120.74, 119.12, 114.47, 114.22, 109.69, 50.90, 47.14, 42.54, 29.01, 28.51 23.92. HRMS (ESI-TOF) m/z Calcd for C_31_H_29_BrN_3_ [M-Br]^+^ 522.1545, found 522.1542.

#### 1-(5-(9H-carbazol-9-yl)pentyl)-5,6-dimethyl-3-(2-oxo-2-phenylethyl)-1H-benzo[d]imidazol-3-ium bromide (**56**)

Yield 90%. White powder, m.p. 261–263 °C. IR *ν*_max_ (cm^−1^): 3127, 3031, 2944, 1696, 1597, 1565, 1482, 1452, 1336, 1222, 1151, 999, 958, 848, 755, 613. ^1^H NMR (400 MHz, DMSO) δ: 9.66 (1H, s), 8.15 (4H, t, J = 5.2 Hz), 7.87 (2H, d, J = 4.0 Hz), 7.80 (1H, t, J = 7.4 Hz), 7.68 (2H, t, J = 7.6 Hz), 7.59 (2H, d, J = 8.2 Hz), 7.42 (2H, t, J = 7.2 Hz), 7.19 (2H, t, J = 7.6 Hz), 6.39 (2H, s), 4.50 (2H, t, J = 7.6 Hz), 4.40 (2H, t, J = 7.6 Hz), 2.40 (3H, s), 2.36 (3H, s), 1.96–1.92 (2H, m), 1.87–1.83 (2H, m), 1.42–1.40 (2H, m). ^13^C NMR (100 MHz, DMSO) δ: 191.76, 142.41, 140.40, 136.93, 136.77, 135.07, 134.22, 130.90, 129.61, 129.54, 128.92, 126.11, 122.49, 120.72, 119.11, 113.87, 113.63, 109.69, 53.59, 46.98, 42.56, 28.94, 28.46, 23.86, 20.44. HRMS (ESI-TOF) m/z Calcd for C_34_H_34_N_3_O [M-Br]^+^ 500.2696, found 500.2691.

#### 1-(5-(9H-carbazol-9-yl)pentyl)-3-(2-(4-methoxyphenyl)-2-oxoethyl)-5,6-dimethyl-1H-benzo[d] imidazol-3-ium bromide (**57**)

Yield 95%. White powder, m.p. 228–230 °C. IR *ν*_max_ (cm^−1^): 3125, 2938, 1684, 1600, 1566, 1454, 1334, 1240, 1177, 1017, 958, 840, 754, 600. ^1^H NMR (400 MHz, DMSO) δ: 9.70 (1H, s), 8.14 (4H, d, J = 7.9 Hz), 7.86 (2H, s), 7.59 (2H, d, J = 8.2 Hz), 7.42 (2H, t, J = 7.4 Hz), 7.21–7.17 (4H, m), 6.35 (2H, s), 4.49 (2H, d, J = 6.7 Hz), 4.39 (2H, d, J = 6.4 Hz), 3.91 (3H, s), 2.40 (3H, s), 2.36 (3H, s), 1.96–1.92 (2H, m), 1.87–1.83 (2H, m), 1.41–1.39 (2H, m). ^13^C NMR (100 MHz, DMSO) δ: 189.97, 164.68, 142.45, 140.39, 136.88, 136.72, 131.39, 130.91, 129.59, 127.05, 126.11, 122.49, 120.71, 119.10, 114.79, 113.82, 113.60, 109.68, 56.31, 53.21, 46.95, 42.56, 28.92, 28.46, 23.84, 20.42. HRMS (ESI-TOF) m/z Calcd for C_35_H_36_N_3_O_2_ [M-Br]^+^ 530.2802, found 530.2795.

#### 1-(5-(9H-carbazol-9-yl)pentyl)-5,6-dimethyl-3-(2-(naphthalen-2-yl)-2-oxoethyl)-1H-benzo[d]imidazol-3-ium bromide (**58**)

Yield 96%. White powder, m.p. 205–207 °C. IR *ν*_max_ (cm^−1^): 3035, 2933, 1686, 1625, 1564, 1454, 1335, 1221, 1188, 1010, 933, 827, 752, 602. ^1^H NMR (400 MHz, DMSO) δ: 9.55 (1H, s), 8.87 (1H, s), 8.16 (1H, d, J = 7.9 Hz), 8.09–8.06 (3H, m), 8.02–7.99 (2H, m), 7.81 (2H, d, J = 8.5 Hz), 7.71–7.62 (2H, m), 7.52 (2H, d, J = 8.0 Hz), 7.35 (2H, t, J = 8.0 Hz), 7.11 (2H, t, J = 8.0 Hz), 6.40 (2H, s), 4.44 (2H, d, J = 7.2 Hz), 4.34 (2H, d, J = 6.8 Hz), 2.33 (3H, s), 2.30 (3H, s), 1.91–1.83 (2H, m), 1.81–1.76 (2H, m), 1.38–1.31 (2H, m). ^13^C NMR (100 MHz, DMSO) δ: 191.65, 142.50, 140.41, 136.99, 136.81, 136.06, 132.51, 131.51, 131.39, 130.95, 130.19, 129.88, 129.66, 129.20, 128.39, 127.91, 126.12, 123.81, 122.50, 120.74, 119.13, 113.85, 113.67, 109.68, 53.55, 47.02, 42.57, 28.95, 28.47, 23.89, 20.45. HRMS (ESI-TOF) m/z Calcd for C_38_H_36_N_3_O [M-Br]^+^ 550.2853, found 550.2848.

#### 1-(5-(9H-carbazol-9-yl)pentyl)-3-(2-(4-bromophenyl)-2-oxoethyl)-5,6-dimethyl-1H-benzo[d]imidazol-3-ium bromide (**59**)

Yield 96%. Yellow powder, m.p. 196–198 °C. IR *ν*_max_ (cm^−1^): 3015, 2926, 1694, 1582, 1452, 1386, 1335, 1227, 1069, 1009, 958, 821, 748, 612. ^1^H NMR (400 MHz, DMSO) δ: 9.63 (1H, s), 8.14 (2H, d, J = 7.7 Hz), 8.07 (2H, d, J = 8.3 Hz), 7.92–7.86 (4H, m), 7.58 (2H, d, J = 8.2 Hz), 7.43 (2H, t, J = 7.4 Hz), 7.19 (2H, t, J = 7.5 Hz), 6.37 (2H, s), 4.49 (2H, t, J = 7.0 Hz), 4.40 (2H, t, J = 6.8 Hz), 2.40 (3H, s), 2.36 (3H, s), 1.96–1.92 (2H, m), 1.87–1.83 (2H, m), 1.42–1.40 (2H, m). ^13^C NMR (100 MHz, DMSO) δ: 191.16, 142.36, 140.40, 136.94, 136.97, 133.27, 132.62, 130.87, 129.60, 129.20, 126.11, 122.49, 120.72, 119.11, 113.89, 113.63, 109.68, 53.57, 47.00, 42.56, 28.94, 28.46, 23.86, 23.86, 20.44. HRMS (ESI-TOF) m/z Calcd for C_34_H_33_BrN_3_O [M-Br]^+^ 578.1802, found 578.1806.

#### 1-(5-(9H-carbazol-9-yl)pentyl)-5,6-dimethyl-3-(4-methylbenzyl)-1H-benzo[d]imidazol-3-ium  bromide (**60**)

Yield 90%. White powder, m.p. 123–125 °C. IR *ν*_max_ (cm^−1^): 3117, 2937, 1598, 1558, 1474, 1453, 1337, 1224, 1125, 1013, 954, 846, 754, 718, 607. ^1^H NMR (400 MHz, DMSO) δ: 9.70 (1H, s), 8.13 (2H, d, J = 7.7 Hz), 7.89 (2H, d, J = 8.4 Hz), 7.57 (2H, d, J = 8.2 Hz), 7.43–7.40 (4H, m), 7.21–7.17 (4H, m), 5.69 (2H, s), 4.41–4.37 (4H, m), 2.35–2.33 (6H, m), 2.27 (3H, s), 1.97–1.90 (2H, m), 1.87–1.80 (2H, m), 1.42–1.35 (2H, m). ^13^C NMR (100 MHz, DMSO) δ: 141.31, 140.38, 138.51, 136.84, 136.80, 131.68, 130.14, 129.93, 129.69, 128.67, 126.07, 122.47, 120.69, 119.09, 113.71, 109.66, 49.90, 46.95, 42.52, 31.17, 28.84, 28.50. 23.92, 21.17, 20.48, 20.40. HRMS (ESI-TOF) m/z Calcd for C_34_H_36_BrN_3_ [M-Br]^+^ 486.2909, found 486.2902.

#### *1-(5-(9H-carbazol-9-yl)pentyl)-3-(2-bromobenzyl)-5,6-dimethyl-1H-benzo[d]imidazol-3-ium bromide (**61***)

Yield 90%. White powder, m.p. 126–127 °C. IR *ν*_max_ (cm^−1^): 3125, 3051, 2938, 2876, 2353, 1599, 1561, 1453, 1335, 1218, 1155, 1029, 953, 845, 752, 611. ^1^H NMR (400 MHz, DMSO) δ: 9.75 (1H, s), 8.12 (2H, d, J = 7.5 Hz), 7.86 (1H, s), 7.74 (1H, d, J = 7.5 Hz), 7.70 (1H, s), 7.55 (2H, t, J = 8.0 Hz), 7.42–7.36 (5H, m), 7.18 (2H, t, J = 7.2 Hz), 5.76 (2H, s), 4.43–4.41 (2H, m), 4.38–4.37 (2H, m), 2.37 (3H, s), 2.35 (3H, s), 1.93–1.90 (2H, m), 1.83–1.80 (2H, m), 1.38–1.35 (2H, m). ^13^C NMR (100 MHz, MeOH) δ: 141.98, 140.37, 137.04, 133.73, 133.22, 131.41, 130.99, 130.04, 130.00, 128.92, 126.08, 123.47, 122.47, 120.71, 119.09, 113.89, 113.59, 109.66, 50.70, 47.01, 42.53, 28.96, 28.51, 23.86, 20.50, 20.42. HRMS (ESI-TOF) m/z Calcd for C_33_H_33_BrN_3_ [M-Br]^+^ 550.1852, found 550.1854.

### MTS assay

Cytotoxicity was determined by performing MTS assay. Briefly, 100 ml of cells suspension were seeded in 96-well cell culture plates and allowed to adhere overnight. The cells were treated with drugs for 48 hours, and then 20 ml of CellTiter 96^®^ AQ_ueous_ One Solution Reagent (Promega, Madison, USA) was added and the cells were further incubated at 37 °C for 1–2 h. Cell viability was measured by reading the absorbance at a wavelength of 490 nm. Concentrations of 50% inhibition of growth (IC50) were calculated on the basis of the relative survival curve.

### Cell apoptosis assay

To analyze the cells for apoptosis, cells were plated and allowed to adhere overnight. Cells were treated with drugs indicated for 48 hours and then analyzed for apoptosis using Annexin-V-FITC/Propidium iodide staining. Cells were trypsinized, pelleted, washed in PBS, and resuspended in 1×binding buffer containing Annexin-V-FITC and propidium iodide (BD Pharmingen) according to the manufacturer’s instructions. The samples were analyzed for the apoptosis using a FACSCalibur flow cytometer (BD Biosciences, Franklin Lakes, NJ).

### Cell cycle analysis

To analyze the DNA content by flow cytometry, cells were collected and washed twice with PBS. Cells were fixed with 70% ethanol overnight. Fixed cells were washed with PBS, and then stained with a 50 μg/ml propidium iodide (PI) solution containing 50 μg/ml RNase A for 30 min at room temperature. Fluorescence intensity was analyzed by FACSCalibur flow cytometer (BD Biosciences, San Jose, CA, USA). The percentages of the cells distributed in different phases of the cell cycle were determined using ModFIT LT 2.0.

## Additional Information

**How to cite this article**: Liu, L.-X. *et al*. Synthesis and antitumor activity of novel *N*-substituted carbazole imidazolium salt derivatives. *Sci. Rep*. **5**, 13101; doi: 10.1038/srep13101 (2015).

## Supplementary Material

Supplementary Information

## Figures and Tables

**Figure 1:**
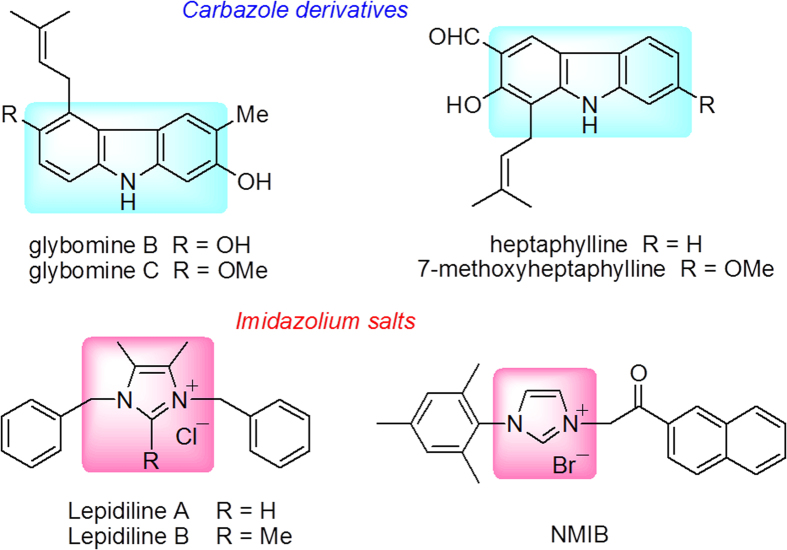
Representative structures of carbazole derivatives and imidazolium salts.

**Table 1:** Structures and yields of compounds 5–61.

Entry	Compound	n	Imidazole ring	R’	Molecular formula	m.p. (°C)	Yields (%)
1	**5**	1	imidazole	—	C_18_H_17_N_3_	101–103	68
2	**6**	1	benzimidazole	—	C_22_H_19_N_3_	45–47	70
3	**7**	1	5,6-dimethyl-benzimidazole	—	C_24_H_23_N_3_	195–197	72
4	**8**	2	imidazole	—	C_19_H_19_N_3_	267–269	70
5	**9**	2	benzimidazole	—	C_23_H_21_N_3_	110–112	72
6	**10**	2	5,6-dimethyl-benzimidazole	—	C_25_H_25_N_3_	112–114	72
7	**11**	3	imidazole	—	C_20_H_21_N_3_	oil	70
8	**12**	3	benzimidazole	—	C_24_H_23_N_3_	149–151	70
9	**13**	3	5,6-dimethyl-benzimidazole	—	C_26_H_27_N_3_	103–105	72
10	**14**	1	imidazole	phenacyl	C_26_H_24_BrN_3_O	124–126	95
11	**15**	1	imidazole	4-methoxyphenacyl	C_27_H_26_BrN_3_O_2_	112–114	95
12	**16**	1	imidazole	naphthylacyl	C_30_H_26_BrN_3_O	136–138	94
13	**17**	1	imidazole	4-bromophenacyl	C_26_H_23_Br_2_N_3_O	105–107	95
14	**18**	1	imidazole	4-bromobenzyl	C_25_H_23_Br_2_N_3_	64–66	80
15	**19**	1	imidazole	4-methylbenzyl	C_26_H_26_BrN_3_	oil	85
16	**20**	1	benzimidazole	phenacyl	C_30_H_26_BrN_3_O	120–122	95
17	**21**	1	benzimidazole	4-methoxyphenacyl	C_31_H_28_BrN_3_O_2_	144–146	95
18	**22**	1	benzimidazole	naphthylacyl	C_34_H_28_BrN_3_O	161–163	95
19	**23**	1	benzimidazole	4-bromobenzyl	C_29_H_25_Br_2_N_3_	222–224	85
20	**24**	1	benzimidazole	4-methylbenzyl	C_30_H_28_BrN_3_	201–203	85
21	**25**	1	benzimidazole	2-bromobenzyl	C_29_H_25_Br_2_N_3_	119–121	75
22	**26**	1	5,6-dimethyl-benzimidazole	naphthylacyl	C_36_H_32_BrN_3_O	159–161	95
23	**27**	1	5,6-dimethyl-benzimidazole	4-methoxyphenacyl	C_33_H_32_BrN_3_O_2_	176–178	94
24	**28**	1	5,6-dimethyl-benzimidazole	4-methylbenzyl	C_32_H_32_BrN_3_	169–171	85
25	**29**	2	imidazole	naphthylacyl	C_32_H_29_BrN_3_O	107–109	95
26	**30**	2	imidazole	4-methoxyphenacyl	C_28_H_28_BrN_3_O_2_	90–92	96
27	**31**	2	imidazole	4-bromophenacyl	C_27_H_25_Br_2_N_3_O	153–155	95
28	**32**	2	imidazole	phenacyl	C_24_H_26_BrN_3_O	96–98	94
29	**33**	2	imidazole	4-methylbenzyl	C_27_H_28_BrN_3_	174–176	85
30	**34**	2	imidazole	2-bromobenzyl	C_26_H_25_Br_2_N_3_	157–159	80
31	**35**	2	benzimidazole	naphthylacyl	C_35_H_30_BrN_3_O	239–241	95
32	**36**	2	benzimidazole	4-methoxyphenacyl	C_32_H_30_BrN_3_O_2_	182–184	96
33	**37**	2	benzimidazole	4-bromophenacyl	C_31_H_27_Br_2_N_3_O	237–239	95
34	**38**	2	benzimidazole	phenacyl	C_31_H_28_BrN_3_O	179–181	95
35	**39**	2	benzimidazole	4-methylbenzyl	C_31_H_30_BrN_3_	196–198	95
36	**40**	2	benzimidazole	2-bromobenzyl	C_30_H_27_Br_2_N_3_	100–102	90
37	**41**	2	5,6-dimethyl-benzimidazole	naphthylacyl	C_37_H_34_BrN_3_O	249–251	95
38	**42**	2	5,6-dimethyl-benzimidazole	4-methoxyphenacyl	C_34_H_34_BrN_3_O_2_	156–158	96
39	**43**	2	5,6-dimethyl-benzimidazole	4-bromophenacyl	C_33_H_31_Br_2_N_3_O	230–232	94
40	**44**	2	5,6-dimethyl-benzimidazole	phenacyl	C_33_H_32_BrN_3_O	152–154	90
41	**45**	2	5,6-dimethyl-benzimidazole	2-bromobenzyl	C_32_H_31_Br_2_N_3_	129–131	85
42	**46**	2	5,6-dimethyl-benzimidazole	4-methylbenzyl	C_33_H_34_BrN_3_	129–131	86
43	**47**	3	imidazole	4-methoxyphenacyl	C_29_H_30_BrN_3_O_2_	oil	90
44	**48**	3	imidazole	naphthylacyl	C_32_H_30_BrN_3_O	116–118	95
45	**49**	3	imidazole	4-methylbenzyl	C_28_H_30_BrN_3_	oil	80
46	**50**	3	benzimidazole	phenacyl	C_32_H_30_BrN_3_O	225–227	90
47	**51**	3	benzimidazole	4-methoxyphenacyl	C_33_H_32_BrN_3_O_2_	131–133	94
48	**52**	3	benzimidazole	naphthylacyl	C_36_H_32_BrN_3_O	120–122	90
49	**53**	3	benzimidazole	4-bromophenacyl	C_32_H_29_Br_2_N_3_ O	187–189	94
50	**54**	3	benzimidazole	4-methylbenzyl	C_32_H_32_BrN_3_	193–195	90
51	**55**	3	benzimidazole	2-bromobenzyl	C_31_H_29_Br_2_N_3_	171–173	90
52	**56**	3	5,6-dimethyl-benzimidazole	phenacyl	C_34_H_34_BrN_3_O	261–263	90
53	**57**	3	5,6-dimethyl-benzimidazole	4-methoxyphenacyl	C_35_H_36_BrN_3_O_2_	228–230	95
54	**58**	3	5,6-dimethyl-benzimidazole	naphthylacyl	C_38_H_36_BrN_3_O	205–207	96
55	**59**	3	5,6-dimethyl-benzimidazole	4-bromophenacyl	C_34_H_33_Br_2_N_3_O	196–198	90
56	**60**	3	5,6-dimethyl-benzimidazole	4-methylbenzyl	C_34_H_36_BrN_3_	123–125	90
57	**61**	3	5,6-dimethyl-benzimidazole	2-bromobenzyl	C_33_H_33_Br_2_N_3_	126–128	90

**Table 2:** Cytotoxic activities of compounds 5–61 *in vitro* against five tumor cell lines^b^ (IC_50_, μM^a^).

Entry	Compound	HL-60	SMMC-7721	A549	MCF-7	SW480
1	**1**	>40	>40	>40	>40	>40
2	imidazole	>40	>40	>40	>40	>40
3	**5**	>40	27.31	>40	>40	>40
4	**6**	7.91	21.59	25.96	13.99	25.84
5	**7**	ND^c^	ND	ND	ND	ND
6	**8**	21.89	>40	37.38	>40	>40
7	**9**	3.11	3.21	12.36	5.06	18.25
8	**10**	20.58	20.31	19.36	17.80	20.01
9	**11**	14.43	>40	31.86	20.74	>40
10	**12**	14.11	>40	37.97	27.65	>40
11	**13**	14.71	14.10	17.64	18.79	17.47
12	**14**	6.23	24.62	>40	12.39	>40
13	**15**	2.44	13.83	25.11	8.78	19.61
14	**16**	2.79	6.99	15.44	4.60	9.53
15	**17**	3.38	11.89	19.62	8.74	12.49
16	**18**	3.09	13.48	24.78	8.25	12.20
17	**19**	2.15	13.65	19.82	6.90	14.98
18	**20**	3.22	15.79	25.87	13.99	15.00
19	**21**	2.28	11.58	15.57	5.92	12.26
20	**22**	2.80	3.27	5.65	2.69	3.28
21	**23**	2.95	15.67	18.19	3.88	9.57
22	**24**	1.17	10.24	12.66	3.85	5.22
23	**25**	1.94	8.54	12.24	3.78	7.41
24	**26**	1.74	3.19	3.89	2.66	3.32
25	**27**	1.99	6.59	11.11	2.46	3.38
26	**28**	9.93	4.89	9.14	10.10	13.67
27	**29**	ND	ND	ND	ND	ND
28	**30**	1.34	8.41	11.07	2.54	11.74
29	**31**	2.42	10.22	15.70	3.95	14.16
30	**32**	2.98	11.69	19.04	19.98	16.39
31	**33**	0.84	5.74	3.92	2.24	9.56
32	**34**	0.49	3.04	2.92	1.95	4.33
33	**35**	2.37	3.53	2.80	2.41	3.33
34	**36**	0.56	2.78	5.16	2.39	3.37
35	**37**	2.30	3.56	3.74	2.54	2.80
36	**38**	0.98	6.32	12.94	2.98	3.84
37	**39**	2.60	3.57	3.15	2.32	3.59
38	**40**	0.71	3.66	3.58	2.14	3.08
39	**41**	3.34	2.41	3.16	1.65	2.50
40	**42**	3.71	2.34	3.60	1.78	2.31
41	**43**	1.80	3.71	4.40	3.35	3.38
42	**44**	0.56	3.74	6.32	2.88	2.97
43	**45**	0.54	2.78	2.83	4.49	5.62
44	**46**	0.70	3.30	3.10	4.10	6.58
45	**47**	0.68	6.34	4.83	3.04	8.69
46	**48**	0.87	2.93	2.99	2.59	4.50
47	**49**	0.55	3.05	2.29	1.91	4.45
48	**50**	2.67	5.41	14.03	3.13	3.83
49	**51**	0.66	2.16	2.80	1.60	2.43
50	**52**	1.36	2.58	3.02	2.25	3.40
51	**53**	2.19	2.88	3.89	3.88	3.39
52	**54**	0.57	2.55	2.65	2.82	3.19
53	**55**	0.64	2.16	3.00	2.39	2.54
54	**56**	1.25	3.31	4.19	3.21	3.48
55	**57**	0.94	2.83	3.39	2.50	3.58
56	**58**	0.76	2.21	2.98	1.94	3.23
57	**59**	2.60	2.71	3.74	3.32	3.64
58	**60**	0.56	2.00	2.84	2.10	2.88
59	**61**	0.51	2.38	3.12	1.40	2.48
60	DDP	1.32	6.24	11.83	15.17	12.95

^a^Cytotoxicity as IC_50_ for each cell line, is the concentration of compound which reduced by 50% the optical density of treated cells with respect to untreated cells using the MTT assay.

^b^Data represent the mean values of three independent determinations.

^c^ND: not determined.
