# Supplementary material for: Synthesis and antitumor activity of novel N-substituted carbazole imidazolium salt derivatives
Source: Sci Rep. 2015 Aug 19;5:13101. doi: 10.1038/srep13101 (PMC4642527; doi:10.1038/srep13101)
Supplement: Supplementary Information [file srep13101-s1.pdf]

Supplementary Information

Synthesis and antitumor activity of novel *N*-substituted carbazole imidazolium salt derivatives

Lan-Xiang Liu[1,4], Xue-Quan Wang[1], Bei Zhou[1], Li-Juan Yang[3], Yan Li[2], Hong-Bin Zhang[1], Xiao-Dong Yang[1]

[1]Key Laboratory of Medicinal Chemistry for Natural Resource, Ministry of Education, Yunnan University, Kunming, 650091, P.R. China, [2]State Key Laboratory for Phytochemistry and Plant Resources in West China, Kunming Institute of Botany, Chinese Academy of Science, Kunming, 650204, P.R. China, [3]Key Laboratory of Ethnic Medicine Resource Chemistry, State Ethnic Affairs Commission & Ministry of Education, Yunnan Minzu University, Kunming, 650500, P.R. China, [4]Research Institute of Resources Insects, Chinese Academy of Forestry, Kunming, 650224, P.R. China.

Correspondence and requests for materials should be addressed to X.-D. Y. (xdyang@ynu.edu.cn), H.-B. Z. (zhanghbyd@gmail.com), or Y. L. (liyanb@mail.kib.ac.cn).

TABLE OF CONTENTS

1. General Experimental... .... S2
2. Experimental Procedures and Analytical Data... S3-S42
3. X-ray crystal structure of compounds **24** and **30**..S43-S70
4. Biological Assay Procedures and Results...S71-S74
5. ^{1}H-NMR and ^{13}C-NMR Spectral of New Compounds............................... S75-S188

1. General Experimental

Melting points were obtained on a XT-4 melting-point apparatus and were uncorrected. Proton nuclear magnetic resonance (^{1}H-NMR) spectra were recorded on a Bruker Avance 300/400 spectrometer at 300/400 MHz. Carbon-13 nuclear magnetic resonance (^{13}C-NMR) was recorded on Bruker Avance 300/400 spectrometer at 75/100 MHz. Chemical shifts are reported as δ values in parts per million (ppm) relative to tetramethylsilane (TMS) for all recorded NMR spectra. Low-resolution Mass spectra were recorded on a VG Auto Spec-3000 magnetic sector MS spectrometer. High Resolution Mass spectra were taken on AB QSTAR Pulsar mass spectrometer. X-Ray data was determined using a Bruker APEX JASCO P-1020 polarimeter.

Silica gel (200–300 mesh) for column chromatography and silica GF_{254} for TLC were produced by Qingdao Marine Chemical Company (China). All air- or moisture-sensitive reactions were conducted under an argon atmosphere. Starting materials and reagents used in reactions were obtained commercially from Acros, Aldrich, Fluka and were used without purification, unless otherwise indicated.

2. Experimental Procedures and Analytical Data

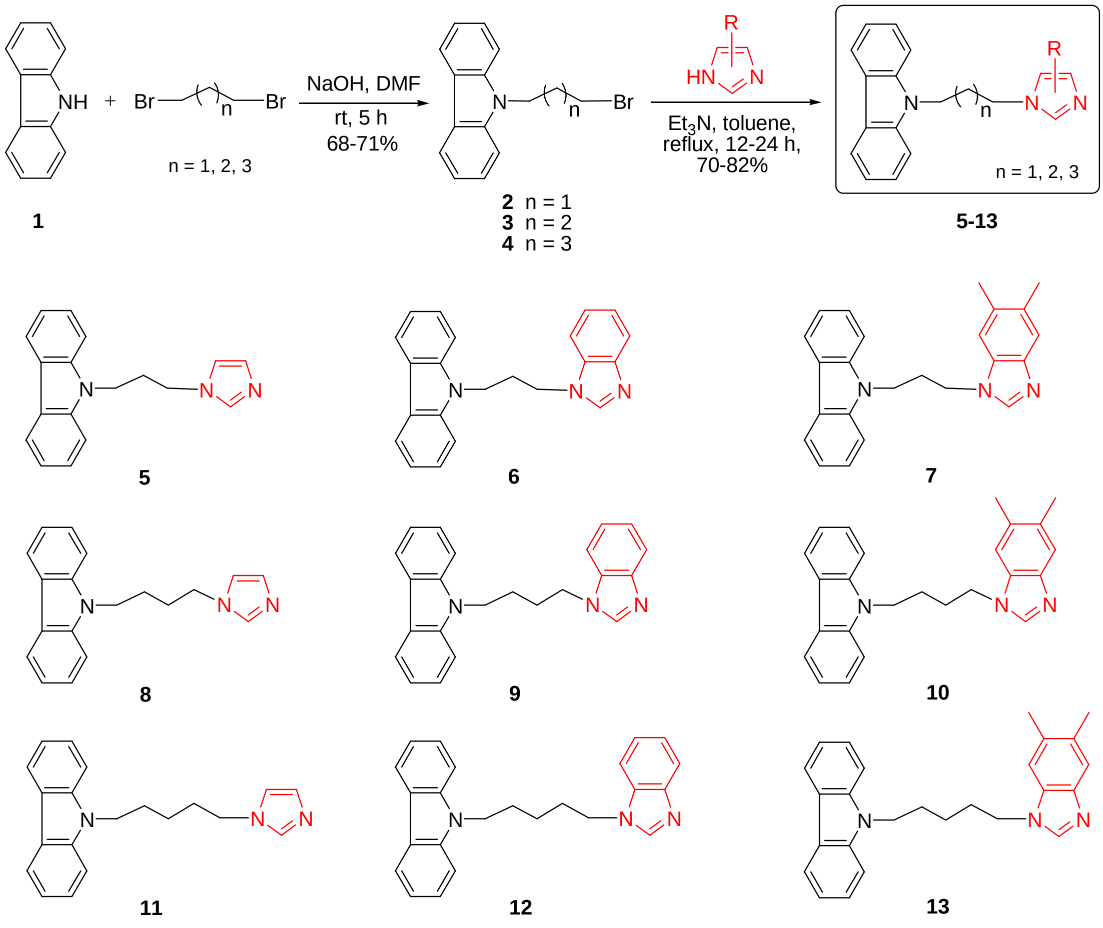

Synthesis of *N*-substituted carbazole–imidazole hybrids **5–13**

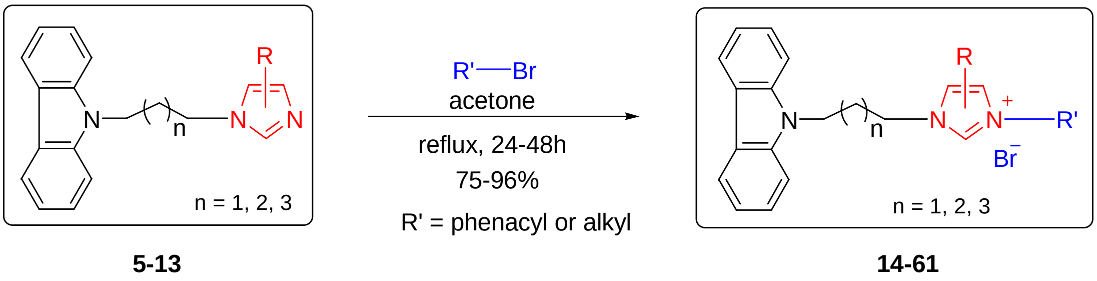

Synthesis of *N*-substituted carbazole imidazolium salt derivatives **14–61** from **5–13**

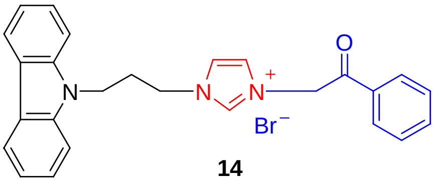

14

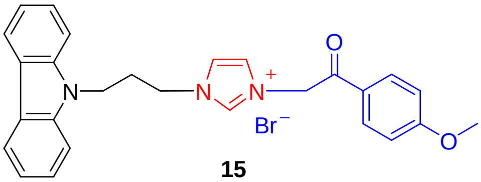

15

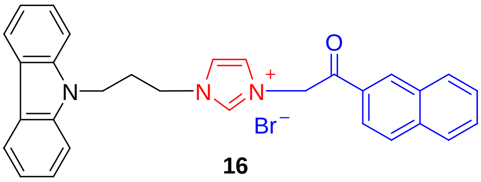

16

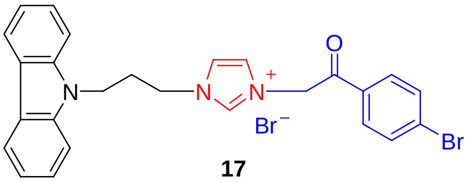

17

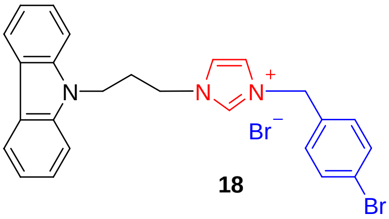

18

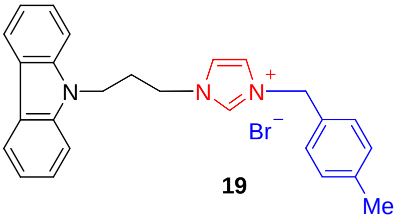

19

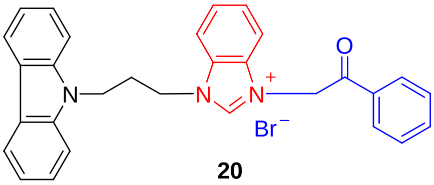

20

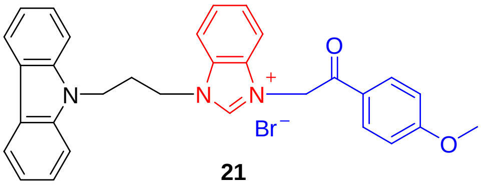

21

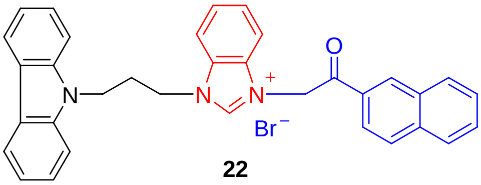

22

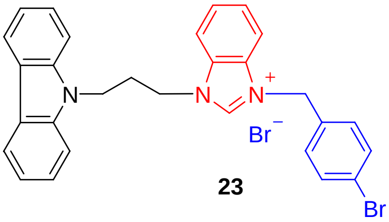

23

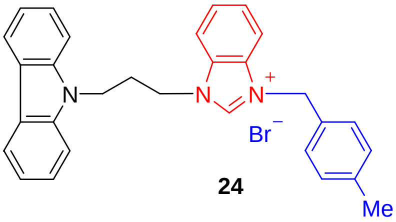

24

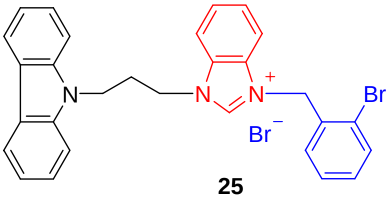

25

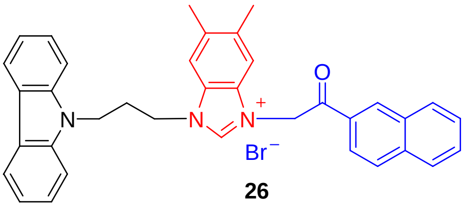

26

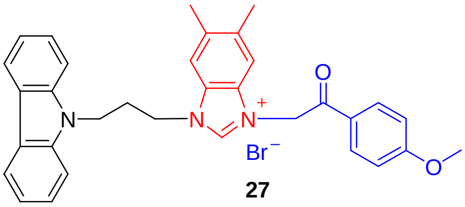

27

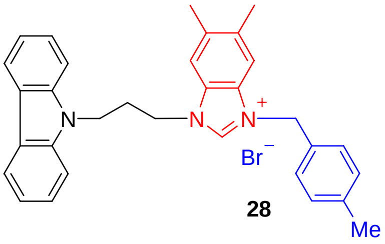

28

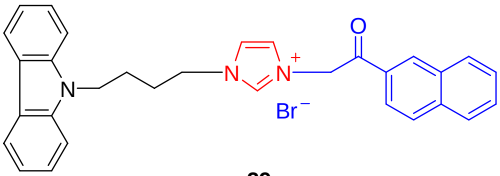

29

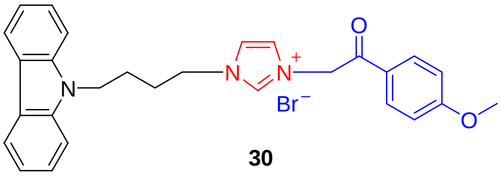

30

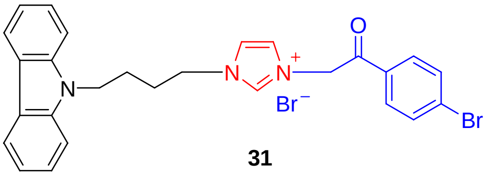

31

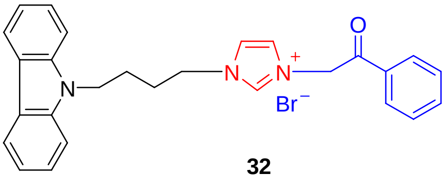

32

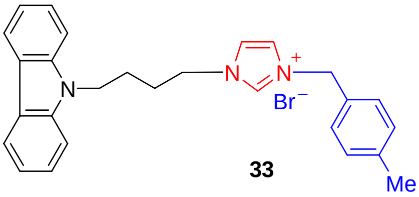

33

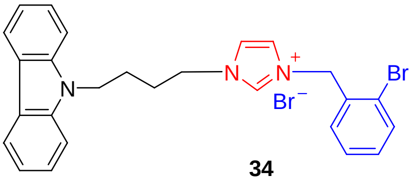

34

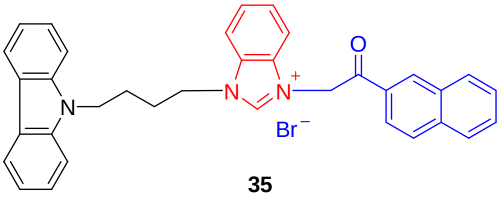

35

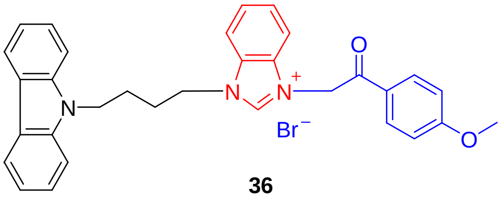

36

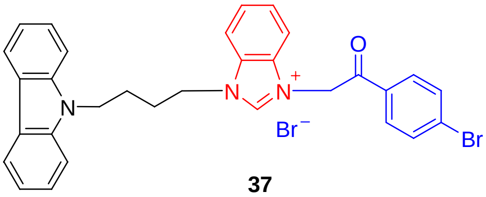

37

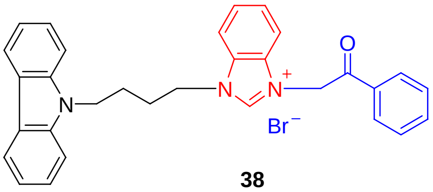

38

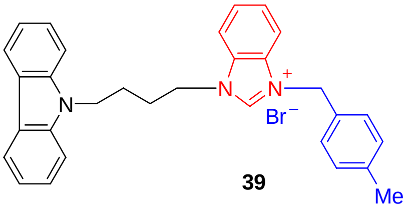

39

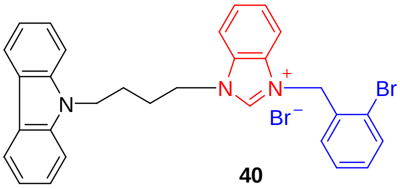

40

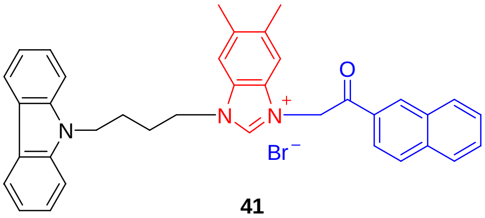

41

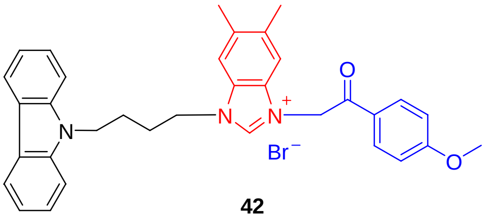

42

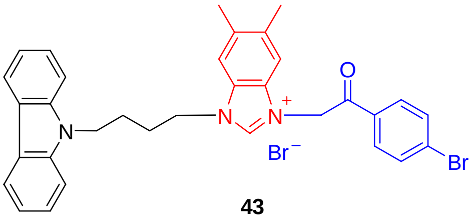

43

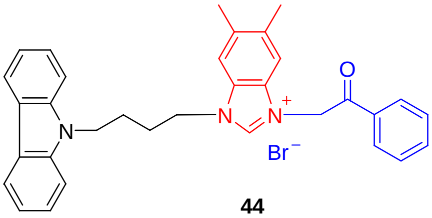

44

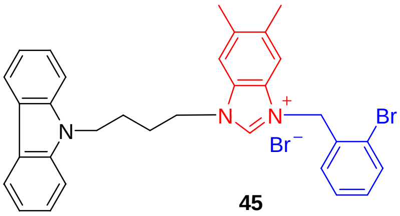

45

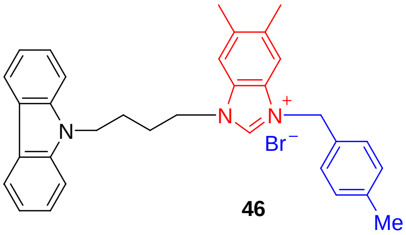

46

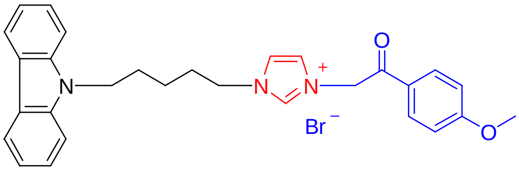

47

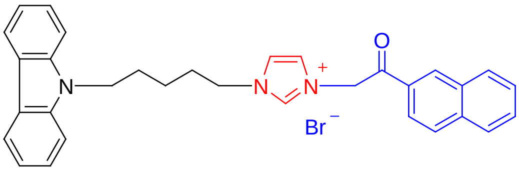

48

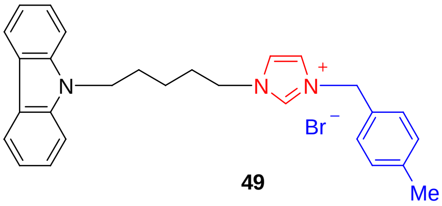

49

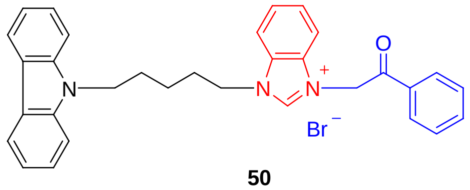

50

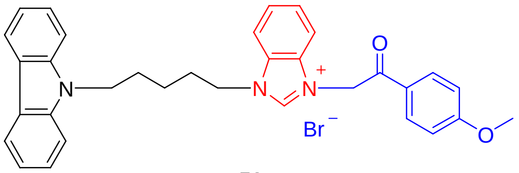

51

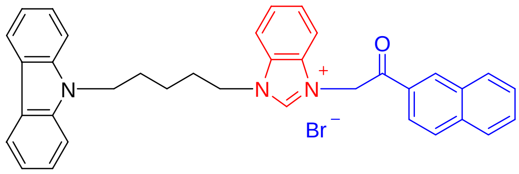

52

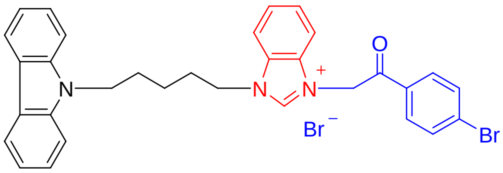

53

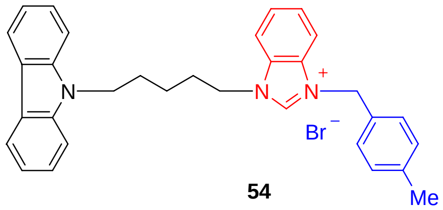

54

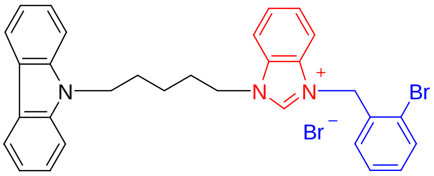

55

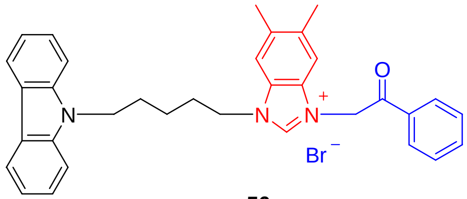

56

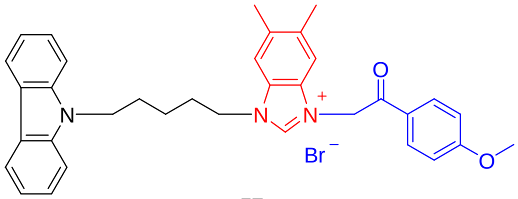

57

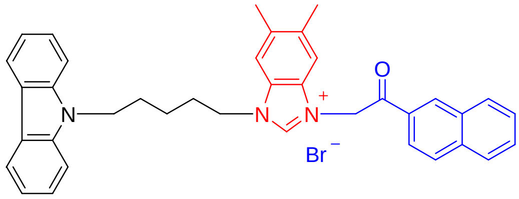

58

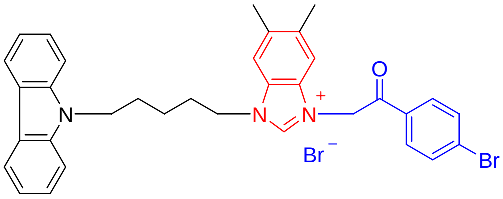

59

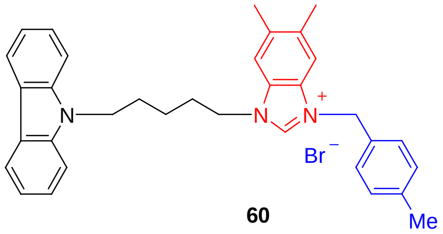

60

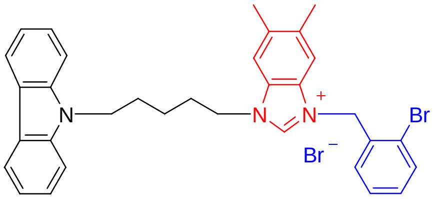

61

2.1 Synthesis of compounds 2-4

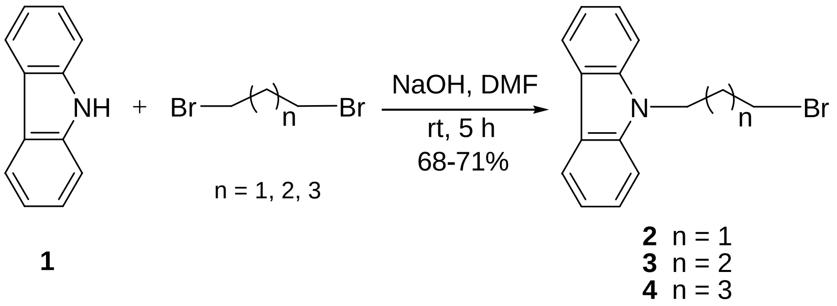

To a mixture of carbazole **1** (1.5 g, 9 mmol) and NaOH (520 mg, 13 mmol) in DMF (30 mL) at 0 °C was added alkyl dibromide (27 mmol). The reaction mixture was stirred at room temperature for 5 h. Reaction progress was monitored by TLC, then diluted with water (50 mL), and extracted with ether (20 mL×3). The combined organic layers were washed with brine (20 mL), dried over anhydrous Na_2SO_4 and concentrated. The residue was purified by column chromatography (silica gel, petroleum ether 60–90 °C : EtOAc = 15:1) to afford **2-4** in 68-72% yield as white powder.

(Wei, Z. L., Petukhov, P. A., Bizik, F., Teixeira, J. C., Mercola, M., Volpe, E. A., Glazer, R. I., Willson, T. M., Kozikowski, A. P. *J. Am. Chem. Soc.* 2004, 126, 16714-16715)

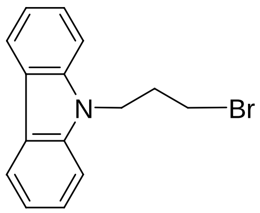

9-(3-Bromopropyl)-9*H*-carbazole (**2**). Yield 68%. White powder, m.p. 148-150 °C. ^{1}H NMR (300 MHz, $CDCl_3$) δ: 8.06 (2H, d, J = 9.0 Hz), 7.47-7.44 (4H, m), 7.43-7.41 (2H, m), 4.42 (2H, t, J = 6.0 Hz), 3.31 (2H, t, J = 6.0 Hz), 2.40-2.32 (2H, m).^{13}C NMR (75 MHz, $CDCl_3$): δ 140.04, 125.90, 123.02, 120.51, 119.20, 108.72, 41.03, 32.04, 30.91.

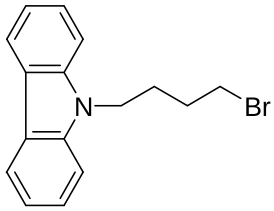

9-(4-Bromobutyl)-9*H*-carbazole (**3**). Yield 68%. White powder, m.p. 104-106 °C. ^{1}H NMR (300 MHz, $CDCl_3$): δ 8.08 (2H, d, J = 6.0 Hz), 7.46 (2H, t, J = 6.0 Hz), 7.38-7.36 (2H, m), 7.19 (2H, t , J = 3.0 Hz), 4.26 (2H, t, J = 5.4 Hz), 3.30 (2H, t, J = 5.4 Hz), 1.90-1.77 (4H, m), 1.53-1.43 (2H, m). ^{13}C NMR (75 MHz, $CDCl_3$): δ140.39, 125.71, 122.91, 120.44, 118.90, 108.61, 42.82, 33.36, 32.50, 28.21, 25.93.

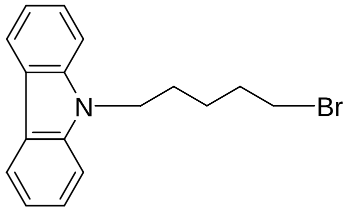

9-(5-Bromopentyl)-9*H*-carbazole (**4**). Yield 72%. White powder, m.p. 51-53 °C. ^{1}H NMR (300 MHz, $CDCl_3$) δ: 8.08 (2H, d, J = 8.7 Hz), 7.47-7.41 (2H, m), 7.35 (2H, d, J = 8.1 Hz), 7.24-7.19 (2H, m), 4.26 (2H, t, J = 6.9 Hz), 3.30 (2H, t, J = 6.9 Hz), 1.90-1.77 (4H, m), 1.53-1.43 (2H, m). ^{13}C NMR (75 MHz, $CDCl_3$): δ 140.38, 125.71, 122.91, 120.44, 118.90, 108.61, 42.83, 33.37, 32.50, 28.21, 25.93.

2.2 Synthesis of compounds 5-13

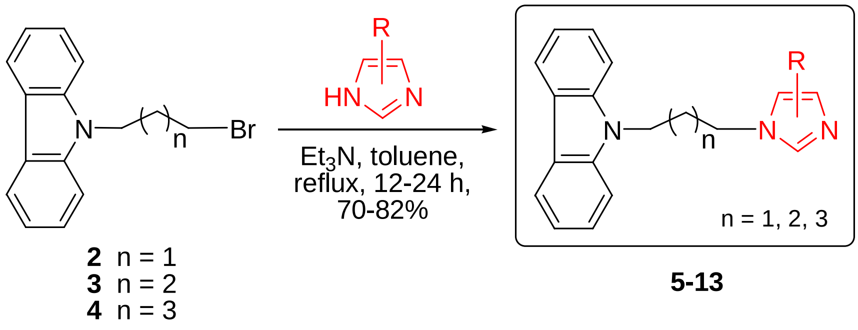

A mixture of compound **2**, **3** or **4** (2 mmol) and imidazole or substituted imidazole (6 mmol) and Et_3N (3 mmol) was stirred in tuloene (20 ml) at reflux for 12–24 h (monitored by TLC). After cooling to room temperature, the solvent was concentrated, and the residue was diluted with EtOAc (20 mL). The organic layer was washed with water (20 mL) and brine (20 mL), dried over anhydrous Na_2SO_4 and concentrated. The residue was purified by column chromatography (silica gel, petroleum ether 60–90 ^oC : EtOAc = 3:1) to afford **5-13** in 68-72% yield as powder or oil.

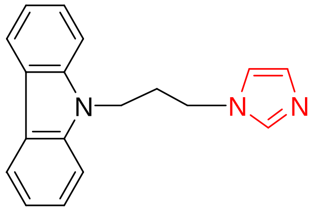

9-(3-(1*H*-Imidazol-1-yl)propyl)-9*H*-carbazole (**5**). Yield 68%. Yellow powder, m.p. 101-103 ^oC. IR ν_{max} (cm^{-1}): 3425, 3106, 3050, 2947, 2872, 1595, 1487, 1453, 1338, 1227, 1163, 1074, 907, 822, 752, 663. ^1H NMR (300 MHz, $CDCl_3$) δ: 8.05 (2H, d, J = 7.8 Hz), 7.41 (2H, t, J = 7.8 Hz), 7.34 (1H, s), 7.23-7.15 (4H, m), 7.06 (1H, s), 6.79 (1H, s), 4.16 (2H, d, J = 6.9 Hz), 3.79 (2H, d, J = 6.9 Hz), 2.29-2.22 (2H, m). ^{13}C NMR (75 MHz, $CDCl_3$) δ: 140.07, 137.10, 129.77, 125.96, 123.04, 120.54, 119.36, 118.60, 108.36, 44.33, 39.66, 29.83. HRMS (ESI-TOF) m/z Calcd for $C_{18}H_{18}N_3$ $[M+1]^+$ 276.1501, found 276.1497.

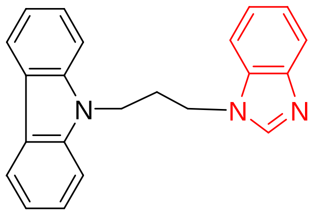

9-(3-(1*H*-Benzo[*d*]imidazol-1-yl)propyl)-9*H*-carbazole (**6**). Yield 70%. White powder, m.p. 45-47 °C. IR ν_{max} (cm^{-1}): 3401, 3051, 2932, 2876, 1599, 1490, 1453, 1376, 1332, 1254, 1215, 1163, 1063, 1008, 930, 889, 747, 624. ^1H NMR (300 MHz, $CDCl_3$) δ: 8.08 (2H, d, J = 6.0 Hz), 7.81 (1H, d, J = 9.0 Hz), 7.75 (1H, s), 7.41 (2H, t, J = 7.5 Hz), 7.35-7.17 (6H, m), 7.12 (1H, d, J = 6.0 Hz), 4.26 (2H, t, J = 7.5Hz), 4.04 (2H, t, J = 7.5Hz), 2.47-2.40 (2H, m). ^{13}C NMR (75 MHz, $CDCl_3$) δ: 189.98, 155.93, 155.31, 136.70, 134.15, 131.33, 131.20, 130.42, 128.07, 126.60, 124.47, 124.09, 123.35, 123.16, 121.28, 120.84, 119.90, 116.38, 116.08, 112.19, 111.81, 58.72, 55.46, 20.85. HRMS (ESI-TOF) m/z Calcd for $C_{22}H_{20}N_3$ $[M+1]^+$ 326.1657, found 326.1649.

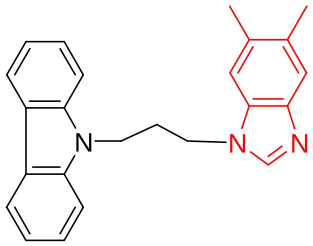

9-(3-(5,6-Dimethyl-1*H*-benzo[*d*]imidazol-1-yl)propyl)-9*H*-carbazole (**7**). Yield 72%. White powder, m.p. 195-197 °C. IR ν_{max} (cm^{-1}): 3425, 3052, 3096, 1595, 1485, 1456, 1333, 1257, 1215, 1164, 1058, 1006, 845, 755, 618. ^1H NMR (300 MHz, $CDCl_3$) δ: 8.12-8.07 (2H, m), 7.69 (1H, s), 7.60 (1H, s), 7.46-7.40 (2H, m), 7.27-7.22 (4H, m), 6.84 (1H, s), 4.32 (2H, t, J = 6.6 Hz), 4.04 (2H, t, J = 7.2 Hz), 2.54-2.34 (2H, m), 2.35 (3H, s), 2.29 (3H, s). ^{13}C NMR (75 MHz, $CDCl_3$) δ: 142.64, 141.98, 140.11, 132.24, 132.06, 131.18, 125.95, 123.10, 120.59, 120.52, 119.35, 109.73, 108.45, 42.39, 39.94, 28.76, 20.52, 20.25. HRMS (ESI-TOF) m/z Calcd for $C_{24}H_{24}N_3$ $[M+1]^+$ 353.1970, found 354.1961.

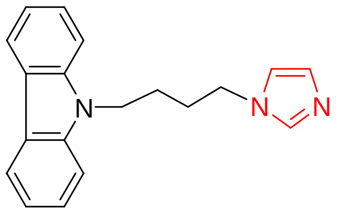

9-(4-(1*H*-imidazol-1-yl)butyl)-9*H*-carbazole (**8**). Yield 70%. White powder, m.p. 267-270 °C. IR ν_{max} (cm^{-1}): 3111, 3054, 2932, 2868, 1594, 1499, 1452, 1327, 1228, 1156, 1069, 1027, 911, 849, 747, 622. ^{1}H NMR (300 MHz, DMSO) δ: 8.05 (2H, d, J = 7.8 Hz), 7.42 (2H, t, J = 8.1 Hz), 7.39 (1H, s), 7.24-7.15 (4H, m), 7.06 (1H, s), 6.79 (1H, s), 4.16 (2H, t, J = 6.9 Hz), 3.80 (2H, t, J = 6.9 Hz), 2.29-2.22 (2H, m). ^{13}C NMR (75 MHz, DMSO) δ: 140.07, 137.10, 129.77, 125.97, 123.04, 120.54, 119.36, 118.59, 108.36, 44.33, 39.67, 29.83. HRMS (ESI-TOF) m/z Calcd for $C_{19}H_{20}N_3$ $[M+1]^+$ 290.1657, found 290.1652.

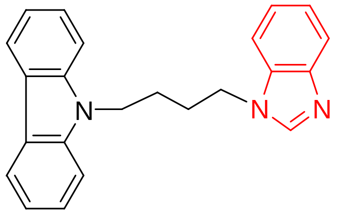

9-(4-(1*H*-benzo[*d*]imidazol-1-yl)butyl)-9*H*-carbazole(**9**). Yield 72%. White powder, m.p. 110-112 °C. IR ν_{max} (cm^{-1}): 3414, 3050, 2934, 2867, 1598, 1489, 1453, 1372, 1334, 1239, 1159, 1067, 1007, 930, 860, 740, 626. ^{1}H NMR (300 MHz, CDCl3) δ: 8.09 (2H, d, J = 7.8 Hz), 7.79 (1H, t, J = 5.7 Hz), 7.65 (1H, s), 7.44 (2H, t, J = 7.5 Hz), 7.32-7.29 (2H, m), 7.26-7.21 (5H, m), 4.26-4.24 (2H, m), 3.96-3.94 (2H, m), 1.88-1.86 (4H, m). ^{13}C NMR (75 MHz, DMSO) δ: 143.87, 142.79, 140.22, 133.63, 125.85, 122.93, 122.13, 120.54, 119.15, 109.49, 108.51, 44.64, 42.35, 27.74, 26.28. HRMS (ESI-TOF) m/z Calcd for $C_{23}H_{22}N_3$ $[M+1]^+$ 340.1814, found 340.1810.

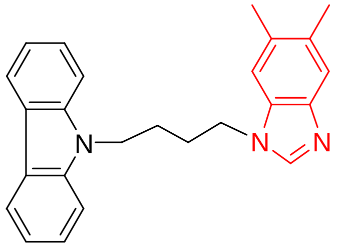

9-(4-(5,6-dimethyl-1*H*-benzo[*d*]imidazol-1-yl)butyl)-9*H*-carbazole (**10**). Yield 72%. White powder, m.p. 112-114 °C. IR ν_{max} (cm^{-1}): 3425, 3051, 2944, 2864, 1594, 1489,

1454, 1335, 1216, 1161, 1061, 1008, 933, 844, 751, 618. ^{1}H NMR (300 MHz, $CDCl_3$) δ: 8.08 (2H, d, J = 7.5 Hz), 7.54 (2H, d, J = 6.3 Hz), 7.43 (2H, t, J = 8.1 Hz), 7.29 (2H, d, J = 8.1 Hz), 7.22 (2H, t, J = 7.5 Hz), 6.97 (1H, s), 4.25-4.22 (2H, m), 3.90-3.88 (2H, m), 2.34 (6H, s), 1.86-1.84 (4H, m). ^{13}C NMR (75 MHz, $CDCl_3$) δ: 142.53, 142.08, 140.24, 132.16, 132.07, 131.00, 125.83, 122.92, 120.50, 120.44, 119.11, 109.67, 108.53, 44.58, 42.35, 27.68, 26.27, 20.61, 20.26. HRMS (ESI-TOF) m/z Calcd for $C_{25}H_{26}N_3$ $[M+1]^+$ 368.2127, found 368.2118.

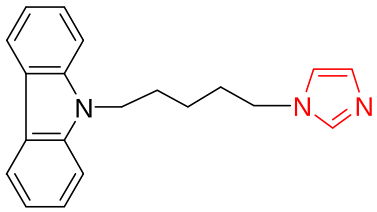

9-(5-(1H-imidazol-1-yl)pentyl)-9H-carbazole (**11**). Yield 70%. Yellow oil. IR ν_{max} (cm^{-1}): 3419, 3049, 2933, 2859, 1595, 1486, 1456, 1328, 1229, 1154, 1077, 909, 818, 726, 664. ^{1}H NMR (300 MHz, DMSO) δ: 8.07 (2H, d, J = 7.8 Hz), 7.46-7.41 (2H, m), 7.33-7.30 (3H, m), 7.24-7.19 (2H, m), 7.01 (1H, s), 6.74 (1H, s), 4.22 (2H, t, J = 6.9 Hz) , 3.73 (2H, t, J = 6.9 Hz), 1.87-1.77 (2H, m), 1.71-1.61 (2H, m), 1.33-1.25 (2H, m). ^{13}C NMR (75MHz, DMSO) δ: 140.31, 136.97, 129.47, 125.73, 122.87, 120.45, 118.96, 118.73, 108.55, 46.69, 42.67, 30.92, 28.46, 24.36. HRMS (ESI-TOF) m/z Calcd for $C_{20}H_{22}N_3$ $[M+1]^+$ 304.1814, found 304.1810.

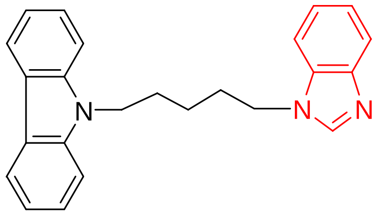

9-(5-(1*H*-benzo[*d*]imidazol-1-yl)pentyl)-9*H*-carbazole (**12**). Yield 72%. Yellow powder, m.p. 149-151 °C. IR ν_{max} (cm^{-1}): 3436, 3051, 2933, 2862, 1923, 1807, 1738, 1598, 1487, 1451, 1368, 1331, 1243, 1203, 1155, 1119, 1074, 1009, 929, 880, 839,

744, 619. ^{1}H NMR (300 MHz, $CDCl_3$) δ: 8.08 (2H, d, J = 7.8 Hz), 7.81-7.78 (1H, m), 7.72 (1H, s), 7.46-7.41 (2H, m), 7.32-7.19 (7H, m), 4.21(2H, t, J = 6.9 Hz), 3.98 (2H, t, J = 6.9 Hz), 1.87-1.75 (4H, m), 1.35-1.30 (2H, m). ^{13}C NMR (75 MHz, $CDCl_3$): δ143.90, 142.82, 140.31, 133.75, 125.74, 122.89, 122.09, 120.46, 118.97, 109.57, 108.54, 44.77, 42.66, 29.78, 28.53, 24.71. HRMS (ESI-TOF) m/z Calcd for $C_{24}H_{24}N_3$ $[M+1]^+$ 354.1970, found 354.1957.

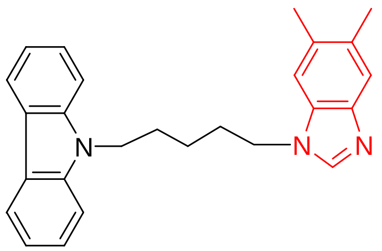

9-(5-(5,6-dimethyl-1*H*-benzo[*d*]imidazol-1-yl)pentyl)-9*H*-carbazole (**13**). Yield 72%. White powder, m.p. 103-105 °C. IR ν_{max} (cm^{-1}): 3415, 3053, 2928, 2859, 1594, 1490, 1455, 1490, 1455, 1334, 1273, 1216, 1156, 1067, 1008, 841, 752, 618. ^{1}H NMR (300 MHz, $CDCl_3$) δ: 8.07 (2H, d, J = 7.8 Hz), 7.62 (1H, s), 7.56 (1H, s), 7.46-7.41 (2H, m), 7.31-7.29 (2H, m), 7.21 (2H, t, J = 7.5 Hz), 7.04 (1H, s), 4.19 (2H, d, J =6.9 Hz), 3.93 (2H, d, J =6.9 Hz), 2.36 (6H, s), 1.85-1.73 (4H, m), 1.36-1.31 (2H, m). ^{13}C NMR (75 MHz, $CDCl_3$) δ: 142.45, 142.09, 140.31, 132.26, 132.08, 131.01, 125.74, 122.86, 120.45, 120.36, 118.95, 109.77, 108.55, 44.74, 42.68, 29.74, 28.57, 24.66, 20.65, 20.29. HRMS (ESI-TOF) m/z Calcd for $C_{26}H_{28}N_3$ $[M+1]^+$ 382.2283, found 382.2777.

2.3 Synthesis of compounds 14-61

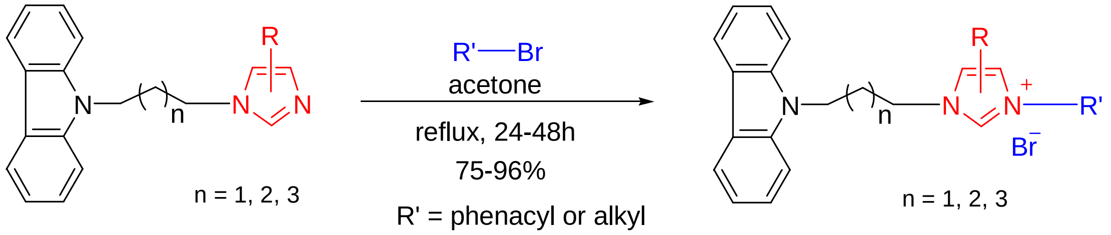

5-13 **14-61**

A mixture of substituted imidazole **5-13** (0.25 mmol) and phenacyl or alkyl (0.75 mmol) was stirred in acetone (10 ml) at reflux 24-48 h (10 ml). An insoluble substance was formed. After completion of the reaction as indicated by TLC, the precipitate was filtered and washed with acetone (3 × 10 ml), then dried to afford imidazolium salts **14-61** in 75–96% yields.

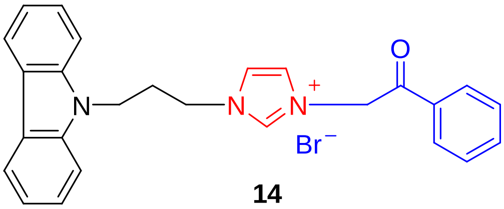

14

1-(3-(9*H*-carbazol-9-yl)propyl)-3-(2-oxo-2-phenylethyl)-1*H*-imidazol-3-iumbromide (**14**). Yield 94%. Yellow powder, m.p. 124-126 ^{o}C. IR ν_{max} (cm^{-1}): 3395, 3134, 3065, 2963, 1697, 1595, 1454, 1338, 1229, 1166, 991, 754, 684. ^{1}H NMR (300 MHz, MeOH) δ: 8.79 (1H, s), 8.05 (2H, d, J = 9.0 Hz), 7.98 (2H, d, J = 9.0Hz), 7.65 (1H, d, J = 9.0 Hz), 7.55-7.50 (4H, m), 7.48-7.41 (4H, m), 7.19 (2H, d, J = 9.0 Hz), 5.76 (2H, s), 4.43 (2H, t, J = 6.0 Hz), 4.25 (2H, t, J =6.0 Hz), 2.44-2.35 (2H, m). ^{13}C NMR (75 MHz, MeOH) δ: 191.90, 141.41, 138.43, 135.81, 130.22, 129.41, 127.23, 125.33, 124.91, 122.93, 121.42, 120.48, 110.12, 56.71, 56.52, 40.76, 30.03. HRMS (ESI-TOF) m/z Calcd for $C_{26}H_{24}N_3O$ [M-Br]$^+$ 394.1914, found 394.1910.

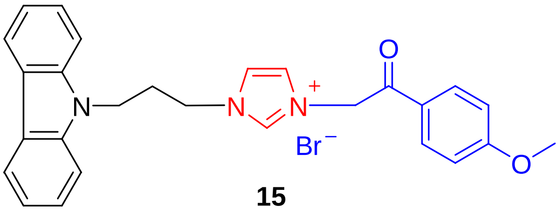

15

1-(3-(9*H*-carbazol-9-yl)propyl)-3-(2-(4-methoxyphenyl)-2-oxoethyl)-1*H*-imidazol-3-ium bromide (**15**). Yield 95%. White powder, m.p. 112-114 ^{o}C. IR ν_{max} (cm^{-1}): 3415, 3141, 3054, 2965, 2839, 1684, 1600, 1454, 1335, 1240, 1167, 1025, 835, 755. ^{1}H

NMR (300 MHz, MeOH) δ: 8.74 (1H, s), 8.00 (2H, d, J = 6.0 Hz), 7.86 (2H, d, J = 9.0 Hz), 7.46-7.40 (4H, m), 7.36 (2H, s), 7.16 (2H, t, J = 7.5 Hz), 6.93 (2H, t, J = 9.0 Hz), 5.62 (2H, s), 4.36 (2H, t, J = 6.0 Hz), 4.19 (2H, t, J = 9.0 Hz), 3.36 (3H, s), 2.32 (2H, m). ^{13}C NMR (75 MHz, MeOH) δ: 190.21, 166.20, 141.30, 138.44, 131.92, 127.62, 127.21, 125.33, 124.11, 122.81, 121.52, 120.51, 115.42, 110.22, 56.41, 56.10, 40.72, 30.02. HRMS (ESI-TOF) m/z Calcd for $C_{27}H_{26}N_3O_2$ $[M-Br]^+$ 424.2020, found 424.2018.

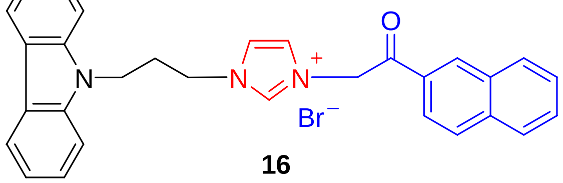

16

1-(3-(9*H*-carbazol-9-yl)propyl)-3-(2-(naphthalen-2-yl)-2-oxoethyl)-1*H*-imidazol-3-ium bromide (**16**). Yield 94%. White powder, m.p. 136-137 °C. IR ν_{max} (cm^{-1}): 3392, 3145, 3047, 2966, 1687, 1630, 1560, 1456, 1336, 1224, 1166, 1034, 935, 818, 753, 620. ^{1}H NMR (300 MHz, MeOH) δ: 8.87 (1H, s), 8.67 (1H, s), 8.07 (3H, d, J = 6.0 Hz), 8.00-7.89 (3H, m), 7.67-7.62 (2H, m), 7.59-7.23 (6H, m), 7.20 (2H, t, J = 6.0 Hz), 5.95 (2H, s), 4.49 (2H, t, J = 6.0 Hz), 4.31 (2H, t, J = 6.0 Hz), 2.47 (2H, m). ^{13}C NMR (75 MHz, MeOH) δ: 191.85, 141.44, 138.65, 137.62, 133.91, 132.35, 131.82, 130.93, 130.49, 130.04, 128.97, 128.40, 127.12, 125.41, 124.25, 122.91, 120.39, 109.99, 56.50, 40.75, 29.91. HRMS (ESI-TOF) m/z Calcd for $C_{30}H_{26}N_3O$ $[M-Br]^+$ 444.2070, found 444.2072.

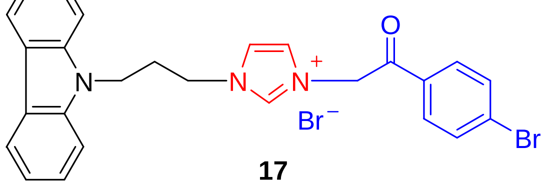

17

1-(3-(9*H*-carbazol-9-yl)propyl)-3-(2-(4-bromophenyl)-2-oxoethyl)-1*H*-imidazol-3-ium bromide (**17**). Yield 95%. White powder, m.p. 105-107°C. IR ν_{max} (cm^{-1}): 3407, 3129, 3053, 2959, 1697, 1582, 1454, 1335, 1228, 1164, 1068, 994, 820, 755, 621. ^{1}H NMR (300 MHz, $CDCl_3$) δ: 8.78 (1H, s), 8.03 (2H, d, J = 6.0 Hz), 7.85 (2H, d, J = 6.0 Hz), 7.65 (2H, d, J = 6.0 Hz), 7.49-7.40 (6H, m), 7.18 (2H, t, J = 6.0 Hz), 5.72 (2H, s), 4.43 (2H, t, J = 6.0 Hz), 4,26 (2H, t, J = 6.0 Hz), 2.42-2.40 (2H, m). ^{13}C NMR (75 MHz, $CDCl_3$) δ: 191.11, 141.32, 138.41, 133.82, 133.52, 131.11, 130.72, 127.21, 125.33, 124.22, 122.93, 121.40, 120.51, 110.09, 56.40, 49.11, 40.72, 30.03. HRMS (ESI-TOF) m/z Calcd for $C_{26}H_{23}BrN_3O$ $[M-Br]^+$ 472.1024, found 472.1022.

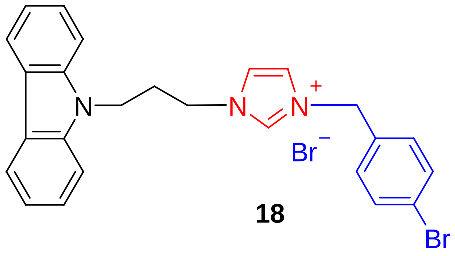

18

1-(3-(9H-carbazol-9-yl)propyl)-3-(4-bromobenzyl)-1H-imidazol-3-ium bromide (**18**). Yield 80%. White powder, m.p. 64-66°C. IR ν_{max} (cm^{-1}): 3411, 3137, 3049, 2949, 1595, 1486, 1453, 1333, 1154, 1067, 1011, 809, 753, 613. ^{1}H NMR (300 MHz, $CDCl_3$) δ: 9.95 (1H, s), 7.96 (2H, d, J = 6.0 Hz), 7.38-7.29 (6H, m), 7.23 (2H, d, J = 6.0 Hz), 7.15 (2H, m), 7.05 (1H, s), 6.98 (1H, s), 5.27 (2H, s), 4.42 (2H, t, J =6.0 Hz), 4.28 (2H, t, J =6.0 Hz), 2.43-2.45 (2H, m). ^{13}C NMR (75 MHz, $CDCl_3$) δ: 139.80, 136.11, 132.42, 131.81, 130.74, 126.23, 123.72, 122.73, 121.93, 121.63, 120.31, 119.42, 108.92, 52.31, 47.92, 40.03, 29.03. HRMS (ESI-TOF) m/z Calcd for $C_{25}H_{23}BrN_3$ $[M-Br]^+$ 444.1070, found 444.1065.

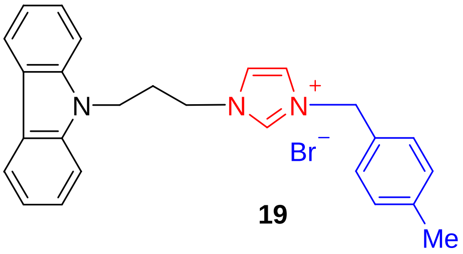

19

1-(3-(9*H*-carbazol-9-yl)propyl)-3-(4-methylbenzyl)-1*H*-imidazol-3-ium bromide (**19**). Yield 85%. Yellow oil. IR ν_{max} (cm^{-1}): 3403, 3129, 3051, 2964, 1599, 1562, 1454, 1334, 1230, 1155, 1054, 832, 755, 626. ^{1}H NMR (300 MHz, $CDCl_3$) δ: 9.96 (1H, s), 7.98 (2H, d, J = 9.0 Hz), 7.44-7.36 (4H, m), 7.19-7.14 (4H, m), 7.08-7.04 (3H, m), 6.92 (1H, s), 5.19 (2H, s), 4.48-4.46 (2H,m), 4.36-4.34 (2H, m), 2.50-2.48 (2H, m), 2.25 (3H, s). ^{13}C NMR (75 MHz, $CDCl_3$) δ: 139.81, 139.51, 136.12, 130.03, 129.52, 128.92, 126.11, 122.73, 121.81, 121.30, 119.32, 109.00, 53.02, 47.92, 40.02, 29.10, 21.11.HRMS (ESI-TOF) m/z Calcd for $C_{26}H_{26}N_3$ $[M-Br]^+$ 380.2127, found 380.2121.

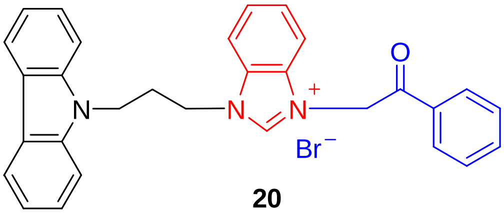

20

1-(3-(9*H*-carbazol-9-yl)propyl)-3-(2-oxo-2-phenylethyl)-1*H*-benzo[*d*]imidazol-3-ium bromide (**20**). Yield 95%. White powder, m.p. 120-122 °C. IR ν_{max} (cm^{-1}): 3425, 3049, 2965, 1695, 1600, 1565, 1482, 1453, 1339, 1229, 1049, 987, 754, 684. ^{1}H NMR (300 MHz, DMSO) δ: 9.74 (1H, s), 8.16-8.11 (5H, m), 8.07-8.04 (1H, m), 7.19 (1H, t, J = 7.2 Hz), 7.69-7.64 (6H, m), 7.44 (2H, t, J = 7.5 Hz), 7.20 (2H, t, J = 7.5 Hz), 6.36 (2H, s), 4.76 (2H, t, J = 7.2Hz), 4.61 (2H, t, J = 6.9Hz), 2.47-2.44 (2H, m). ^{13}C NMR (75 MHz, DMSO) δ: 191.68, 143.79, 140.21, 135.08, 134.15, 132.38, 131.11, 129.54, 128.90, 127.26, 127.03, 126.25, 122.65, 120.80, 119.45, 114.43, 114.04, 109.70, 53.68, 54.20, 28.58. HRMS (ESI-TOF) m/z Calcd for $C_{30}H_{26}N_3O$ $[M-Br]^+$ 444.2076, found 444.2072.

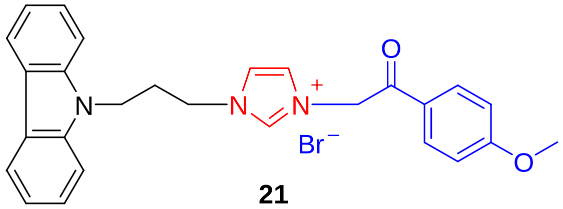

21

1-(3-(9*H*-carbazol-9-yl)propyl)-3-(2-(4-methoxyphenyl)-2-oxoethyl)-1*H*-benzo[*d*]imidazol-3-ium bromide (**21**). Yield 95%. Yellow powder, m.p. 144-146 °C. IR ν_{max} (cm^{-1}): 3431, 3378, 3035, 2933, 2847, 1683, 1600, 1565, 1454, 1331, 1239, 1175, 1024, 983, 837, 755. ^{1}H NMR (300 MHz, DMSO) δ: 9.81 (1H, s), 8.16-8.05 (6H, m), 7.69-7.67 (4H, m), 7.44 (2H, t, J = 7.2 Hz), 7.23-7.16 (4H, m), 6.34 (2H, s), 4.80-4.78 (2H, m), 4.63-4.62 (2H, m), 3.89 (3H, s), 2.49-2.47 (2H, m). ^{13}C NMR (75 MHz, DMSO) δ: 197.37, 164.18, 143.40, 139.73, 131.91, 130.85, 130.64, 126.70, 126.50, 125.72, 122.16, 120.39, 118.93, 114.30, 113.90, 113.56, 109.20, 55.78, 52.76, 44.67, 39.47, 28.12. HRMS (ESI-TOF) m/z Calcd for $C_{31}H_{28}N_3O_2$ [M-Br]$^+$ 474.2182, found 474.2174.

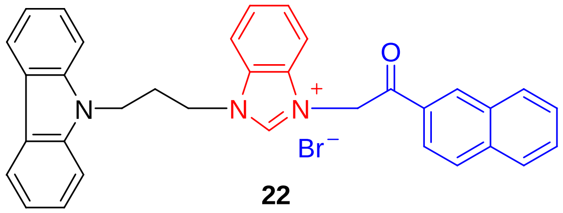

22

1-(3-(9*H*-carbazol-9-yl)propyl)-3-(2-(naphthalen-2-yl)-2-oxoethyl)-1*H*-benzo[*d*]imidazol-3-ium bromide (**22**). Yield 95%. Yellow powder, m.p. 161-163 °C. IR ν_{max} (cm^{-1}): 3429, 3129, 3048, 2952, 1688, 1625, 1564, 1454, 1339, 1221, 1187, 1008, 938, 862, 821, 753. ^{1}H NMR (300 MHz, DMSO) δ: 9.87 (1H, s), 8.96 (1H, s), 8.24 (1H, d, J = 7.5 Hz), 8.17-8.12 (5H, m), 8.08-8.06 (2H, m), 7.77-7.68 (6H, m), 7.45 (2H, d, J = 7.5 Hz), 7.21 (2H, d, J = 7.5 Hz), 6.55 (2H, s), 4.81 (2H, t, J = 6.9 Hz), 4.64 (2H, t, J = 6.9 Hz), 2.49-2.48 (2H, m). ^{13}C NMR (75 MHz, DMSO) δ: 191.11, 143.42, 139.74, 135.55, 131.95, 130.97, 130.68, 129.70, 129.36, 128.68, 127.85, 127.37, 126.77,

126.54, 125.75, 123.29, 122.17, 120.32, 118.95, 113.97, 113.60, 109.21, 53.23, 44.71, 39.47, 28.14. HRMS (ESI-TOF) m/z Calcd for $C_{34}H_{28}N_3O$ $[M\text{-}Br]^+$ 494.2232, found 494.2227.

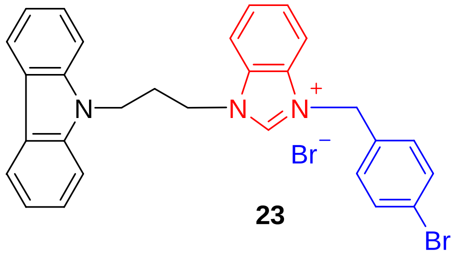

23

1-(3-(9*H*-carbazol-9-yl)propyl)-3-(4-bromobenzyl)-1*H*-benzo[*d*]imidazol-3-ium bromide (**23**). Yield 95%. Yellow powder, m.p. 222-224 °C. IR ν_{max} (cm^{-1}): 3425, 3031, 2953, 1600, 1563, 1485, 1453, 1335, 1225, 1069, 806, 750. ^1H NMR (300 MHz, DMSO) δ: 9.96 (1H, s), 8.15 (2H, d, J = 7.8 Hz), 8.11-8.08 (1H, m), 7.92-7.89 (1H, m), 7.70 (2H, d, J = 8.1 Hz), 7.65-7.57 (4H, m), 7.49-7.23 (4H, m), 7.21 (2H, t, J = 7.5 Hz), 5.71 (2H, s), 4.66-4.64 (4H, m), 2.49-2.47 (2H, m). ^{13}C NMR (75 MHz, DMSO) δ: 142.25, 139.77, 133.28, 131.76, 131.24, 130.57, 126.60, 126.50, 125.70, 122.14, 121.99, 120.29, 118.91, 113.72, 109.29, 49.13, 44.78, 39.51, 28.02. HRMS (ESI-TOF) m/z Calcd for $C_{29}H_{25}$ BrN_3 $[M\text{-}Br]^+$ 494.1232, found 494.1226.

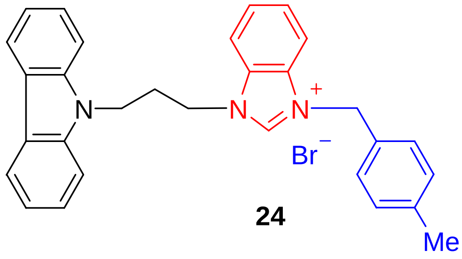

24

1-(3-(9H-carbazol-9-yl)propyl)-3-(4-methylbenzyl)-1H-benzo[d]imidazol-3-ium bromide (**24**). Yield 85%. White powder, m.p. 201-203 °C. IR ν_{max} (cm^{-1}): 3411, 3117, 3025, 2956, 1606, 1564, 1453, 1336, 1224, 1143, 1028, 931, 752. ^1H NMR (300 MHz, DMSO) δ: 9.87 (1H, s), 8.15 (2H, d, J = 7.8 Hz), 8.05 (1H, d, J = 4.8 Hz), 7.89 (1H, t,

J = 5.0 Hz), 7.68-7.61 (4H, m), 7.47-7.37 (4H, m), 7.23-7.17 (4H, m), 5.63 (2H, s), 4.64-4.62 (4H, m), 2.49-2.48 (2H, m), 2.64 (3H, s). ^{13}C NMR (75 MHz, DMSO) δ: 142.30, 139.76, 138.09, 131.24, 130.82, 129.41, 128.26, 126.54, 125.70, 122.15, 120.31, 118.93, 113.63, 109.21, 49.68, 44.73, 39.49, 27.99, 20.65. HRMS (ESI-TOF) m/z Calcd for $C_{30}H_{28}N_3$ $[M-Br]^+$ 430.2283, found 430.2278.

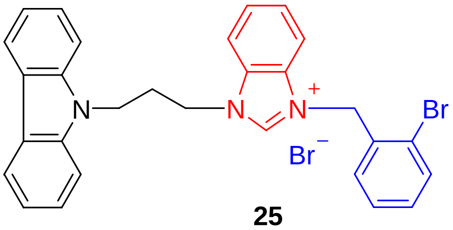

25

1-(3-(9H-carbazol-9-yl)propyl)-3-(2-bromobenzyl)-1H-benzo[d]imidazol-3-ium bromide (**25**). Yield 75%. Yellow powder, m.p. 119-121 °C. IR ν_{max} (cm^{-1}): 3129, 3045, 2937, 1707, 1600, 1562, 1452, 1336, 1223, 1147, 1027, 753, 609. ^{1}H NMR (300 MHz, $CDCl_3$) δ: 9.91 (1H, s), 8.24 (1H, s), 8.15-8.12 (3H, m), 7.87-7.85 (1H, m), 7.72-7.69 (3H, m), 7.64-7.62 (2H, m), 7.46-7.33 (5H, m), 7.20 (2H, t, J = 7.5 Hz), 5.78 (2H, s), 4.75-4.65 (4H, m), 2.48-2.47 (2H, m). ^{13}C NMR (75 MHz, $CDCl_3$) δ: 142.96, 139.74, 133.17, 132.54, 131.09, 130.94, 130.64, 128.36, 126.81, 126.60, 125.70, 122.96, 122.13, 120.28, 118.91, 113.90, 113.62, 109.30, 50.33, 44.86, 39.77, 28.14. HRMS (ESI-TOF) m/z Calcd for $C_{29}H_{25}BrN_3$ $[M-Br]^+$ 494.1232, found 494.1228.

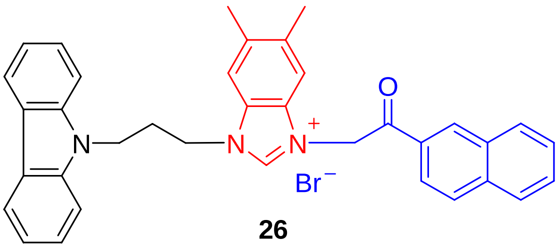

26

1-(3-(9*H*-carbazol-9-yl)propyl)-5,6-dimethyl-3-(2-(naphthalen-2-yl)-2-oxoethyl)-1*H*-benzo[*d*]imidazol-3-ium bromide (**26**). Yield 95%. White powder, m.p. 159-161 °C. IR ν_{max} (cm^{-1}): 3129, 3045, 2960, 1688, 1625, 1564, 1454, 1335, 1220, 1128, 1013,

935, 829, 754, 683. ^{1}H NMR (300 MHz, DMSO) δ: 9.70 (1H, s), 8.96 (1H, s), 8.24 (1H, d, J = 7.92 Hz), 8.17-8.13 (3H, m), 8.08-8.06 (2H, m), 7.90 (1H, s), 7.78-7.69 (5H, m), 7.47 (2H, t, J = 7.4 Hz), 7.22 (2H, t, J = 7.4 Hz), 6.47 (2H, s), 4.71 (2H, t, J = 7.1 Hz), 4.64 (2H, t, J = 6.8 Hz), 2.48-2.46 (2H, m), 2.36-2.35 (6H, m). ^{13}C NMR (75 MHz, DMSO) δ: 191.63, 142.64, 140.24, 136.93, 136.03, 132.50, 131.49, 131.42, 130.91, 130.19, 129.84, 129.56, 129.17, 128.35, 127.86, 126.24, 123.79, 122.67, 120.82, 119.44, 113.88, 113.45, 109.74, 53.60, 45.04, 28.67, 20.43. HRMS (ESI-TOF) m/z Calcd for $C_{36}H_{32}N_3O$ [M-Br]$^+$ 522.2540, found 522.2541.

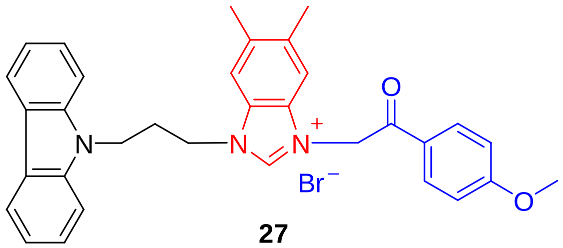

27

1-(3-(9*H*-carbazol-9-yl)propyl)-3-(2-(4-methoxyphenyl)-2-oxoethyl)-5,6-dimethyl-1*H*-benzo[*d*]imidazol-3-ium bromide (**27**). Yield 94%. White powder, m.p. 176-179 °C. IR ν_{max} (cm^{-1}): 3128, 3015, 2966, 1682, 1601, 1566, 1454, 1337, 1240, 1181, 1020, 956, 840, 752, 690, 603. ^{1}H NMR (400 MHz, DMSO) δ: 9.67 (1H, s), 8.16-8.09 (4H, m), 7.82 (1H, s), 7.77 (1H, s), 7.68 (2H, d, J = 8.2Hz), 7.45 (2H, t, J = 7.4 Hz), 7.23-7.17 (4H, m), 6.26 (2H, s), 4.69 (2H, t, J = 7.2 Hz), 4.62 (2H, t, J = 6.8 Hz), 3.90 (3H, s), 2.48-2.46 (2H, m), 2.36-2.35 (6H, m). ^{13}C NMR (100 MHz, DMSO) δ: 189.91, 164.66, 142.60, 140.22, 136.86, 136.65, 131.35, 130.88, 129.52, 127.02, 126.21, 122.64, 120.80, 119.42, 114.78, 113.80, 113.42, 109.74, 56.28, 53.15, 44.99, 39.99, 28.65, 20.41. HRMS (ESI-TOF) m/z Calcd for $C_{33}H_{32}N_3O_2$ [M-Br]$^+$ 502.2489, found 502.2492.

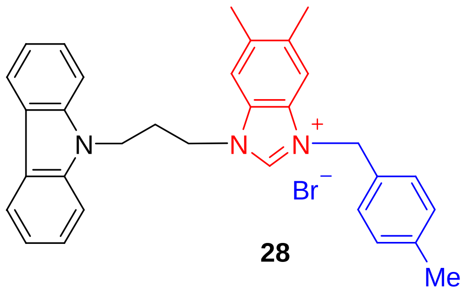

28

1-(3-(9*H*-carbazol-9-yl)propyl)-5,6-dimethyl-3-(4-methylbenzyl)-1*H*-benzo[*d*]imidazol-3-ium bromide (**28**). Yield 85%. White powder, m.p. 169-171 °C. IR ν_{max} (cm^{-1}): 3124, 3023, 2961, 1600, 1563, 1453, 1336, 1221, 1126, 1014, 845, 755, 673. ^{1}H NMR (400 MHz, DMSO) δ: 9.73 (1H, s), 8.15-8.13 (2H, m), 7.69-7.67 (4H, m), 7.46-7.44 (2H, m), 7.35-7.33 (2H, m), 7.20-7.17 (4H, m), 5.55 (2H, s), 4.62-4.55 (4H, m), 2.35-2.30 (6H, m), 2.26-2.25 (2H, m), 2.08 (3H, s). ^{13}C NMR (100 MHz, DMSO) δ: 141.51, 140.24, 138.50, 136.69, 131.54, 130.07, 129.90, 129.68, 128.61, 126.19, 122.62, 120.80, 119.42, 113.55, 109.75, 49.92, 45.09, 39.99, 31.16, 28.48, 21.15, 20.43. HRMS (ESI-TOF) m/z Calcd for $C_{32}H_{32}N_3$ [M-Br]$^+$ 458.2596, found 458.2591.

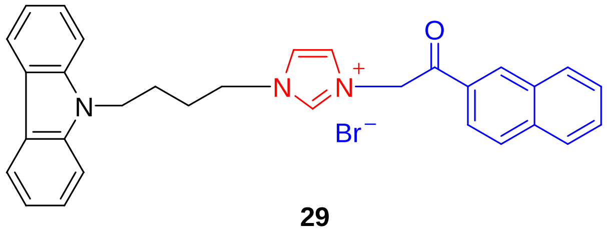

29

1-(4-(9*H*-fluoren-9-yl)butyl)-3-(2-(naphthalen-2-yl)-2-oxoethyl)-1*H*-imidazol-3-ium bromide (**29**). Yield 95%. White powder, m.p. 107-109 °C. IR ν_{max} (cm^{-1}): 3051, 2943, 2859, 1692, 1625, 1593, 1564, 1455, 1335, 1226, 1166, 1029, 939, 861, 753, 626. ^{1}H NMR (400 MHz, DMSO) δ: 9.29 (1H, s), 8.86 (1H, s), 8.22-8.11 (4H, m), 8.7-8.02 (2H, m), 7.92 (1H, s), 7.83 (1H, s), 7.75-7.65 (4H, m), 7.47 (2H, t, J = 7.4 Hz), 7.21 (2H, t, J = 7.4 Hz), 6.27 (2H, s), 4.48 (2H, t, J = 6.7 Hz), 4.37 (2H, t, J = 6.7 Hz), 1.95-1.92 (2H, m), 1.82-1.79 (2H, m). ^{13}C NMR (100 MHz, DMSO) δ: 191.74, 140.38, 137.82, 135.99, 132.51, 131.43, 131.06, 130.17, 129.81, 129.25, 128.35, 127.86, 126.23, 124.71, 123.65, 122.56, 122.51, 120.80, 119.26, 109.78, 56.02, 49.13,

42.10, 27.68, 25.69. HRMS (ESI-TOF) m/z Calcd for $C_{31}H_{28}N_3O$ $[M-Br]^+$ 457.2227, found 457.2226.

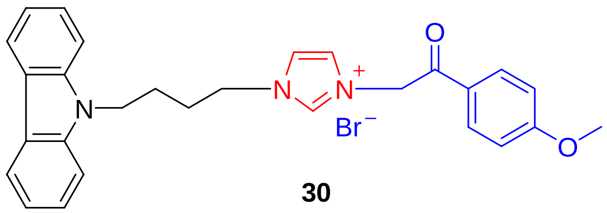

30

1-(4-(9*H*-carbazol-9-yl)butyl)-3-(2-(4-methoxyphenyl)-2-oxoethyl)-1*H*-imidazol-3-ium bromide (**30**). Yield 96%. White powder, m.p. 90-92 °C. IR ν_{max} (cm^{-1}): 3054, 2937, 2835, 1688, 1599, 1564, 1499, 1455, 1340, 1240, 1169, 1026, 935, 835, 757, 627. ^1H NMR (400 MHz, DMSO) δ: 9.22 (1H, s), 8.16 (2H, d, J = 7.7 Hz), 8.03 (2H, d, J = 8.6 Hz), 7.88 (1H, s), 7.76 (1H, s), 7.65 (2H, d, J = 8.2 Hz), 7.47 (2H, t, J = 7.4 Hz), 7.21(2H, t, J = 7.4 Hz), 7.15 (2H, d, J = 8.6 Hz), 6.05 (2H, s), 4.48 (2H, t, J = 6.7 Hz), 4.33 (2H, t, J = 6.7 Hz), 3.41 (3H, s), 1.93-1.90 (2H, m), 1.80-1.77 (2H, m). ^{13}C NMR (100 MHz, DMSO) δ: 190.03, 164.56, 140.37, 137.77, 131.07, 126.94, 126.21, 124.67, 122.54, 122.39, 120.79, 119.24, 114.82, 109.77, 56.26, 55.56, 49.08, 42.08, 27.67, 25.67. HRMS (ESI-TOF) m/z Calcd for $C_{28}H_{28}N_3O_2$ $[M-Br]^+$ 438.2176, found 438.2177.

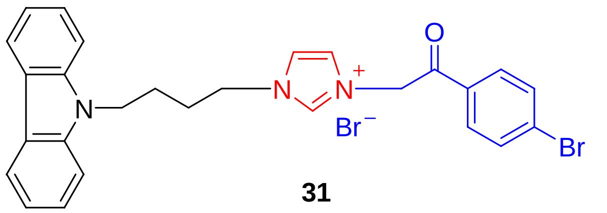

31

1-(4-(9*H*-carbazol-9-yl)butyl)-3-(2-(4-bromophenyl)-2-oxoethyl)-1*H*-imidazol-3-ium bromide (**31**). Yield 95%. White powder, m.p. 153-155 °C. IR ν_{max} (cm^{-1}): 3141, 3049, 2933, 2851, 1698, 1582, 1455, 1389, 1230, 1166, 1069, 993, 823, 755, 622. ^1H NMR (400 MHz, DMSO) δ: 9.25-9.22 (1H, m), 8.16 (2H, d, J = 7.7 Hz), 7.98 (2H, d, J = 7.9 Hz), 7.89-7.84 (3H, m), 7.77 (1H, s), 7.65 (2H, d, J = 8.2 Hz), 7.46 (2H, t, J = 7.5

Hz), 7.20 (2H, t, J = 7.4 Hz), 6.10 (2H, s), 4.47 (2H, t, J = 6.7 Hz), 4.34 (2H, t, J = 6.7 Hz), 1.92-1.90 (2H, m), 1.80-1.77 (2H, m). ^{13}C NMR (100 MHz, DMSO) δ: 191.21, 140.36, 137.73, 133.18, 132.66, 130.60, 129.11, 126.20, 124.62, 122.54, 122.48, 120.79, 119.24, 109.76, 55.93, 49.11, 42.09, 27.67, 25.67. HRMS (ESI-TOF) m/z Calcd for $C_{27}H_{25}BrN_3O$ $[M-Br]^+$ 486.1181, found 486.1176.

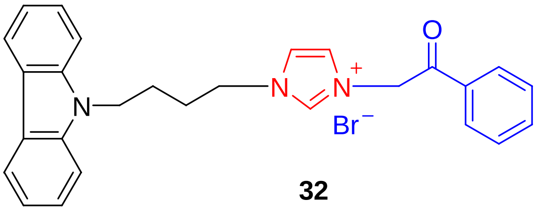

32

1-(4-(9H-carbazol-9-yl)butyl)-3-(2-oxo-2-phenylethyl)-1H-imidazol-3-ium bromide (**32**). Yield 94%. White powder, m.p.96-97 °C. IR ν_{max} (cm^{-1}): 3133, 3054, 2942, 2859, 1903, 1696, 1593, 1565, 1453, 1337, 1231, 1165, 1119, 990, 818, 756, 686. ^{1}H NMR (400 MHz, DMSO) δ: 9.21 (1H, s), 8.16 (2H, d, J = 7.7 Hz), 8.05 (2H, d, J = 7.6 Hz), 7.88 (1H, s), 7.78-7.74 (2H, m), 7.66-7.61 (4H, m), 7.46 (2H, t, J = 7.5 Hz), 7.21 (2H, t, J = 7.4 Hz), 6.11 (2H, s), 4.48 (2H, t, J = 6.7 Hz), 4.34 (2H, t, J = 6.7 Hz), 1.93-1.90 (2H, m), 1.80-1.77 (2H, m). ^{13}C NMR (100 MHz, DMSO) δ: 191.81, 140.37, 137.75, 134.99, 134.12, 129.57, 128.63, 126.21, 124.66, 122.54, 122.46, 120.80, 119.24, 109.76, 55.94, 49.10, 42.08, 27.67, 25.67. HRMS (ESI-TOF) m/z Calcd for $C_{27}H_{26}N_3O$ $[M-Br]^+$ 408.2076, found 408.2072.

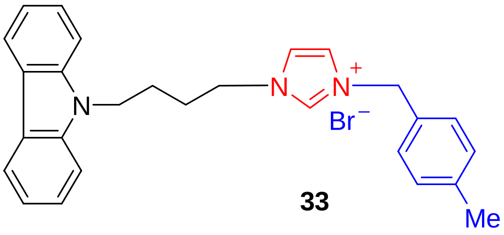

33

1-(4-(9*H*-carbazol-9-yl)butyl)-3-(4-methylbenzyl)-1*H*-imidazol-3-ium bromide (**33**). Yield 85%. Yellow powder, m.p. 174-176 °C. IR ν_{max} (cm^{-1}): 3133, 2948, 2864, 1598, 1558, 1453, 1334, 1231, 1153, 1027, 826, 754, 622. ^{1}H NMR (400 MHz, DMSO) δ:

9.42 (1H, s), 8.15 (2H, d, J = 7.7 Hz), 7.81 (2H, d, J = 7.9 Hz), 7.62 (2H, d, J = 8.2 Hz), 7.44 (2H, t, J = 7.4 Hz), 7.29 (2H, d, J = 7.8 Hz), 7.22-7.14 (4H, m), 5.39 (2H, s), 4.44 (2H, t, J = 6.7 Hz), 4.23 (2H, t, J = 6.7 Hz), 2.27 (3H, s), 1.90-1.86 (2H, m), 1.74-1.72 (2H, m). ^{13}C NMR (100 MHz, DMSO) δ: 140.33, 138.60, 136.47, 132.44, 129.95, 128.76, 126.18, 123.14, 123.00, 122.53, 120.79, 119.22, 109.71, 52.10, 49.09, 42.06, 27.43, 25.66, 21.18. HRMS (ESI-TOF) m/z Calcd for $C_{27}H_{28}N_3$ $[M\text{-}Br]^+$ 394.2278, found 394.2274.

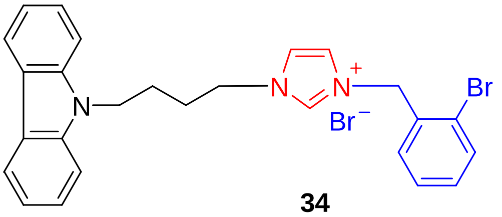

34

1-(4-(9*H*-carbazol-9-yl)butyl)-3-(2-bromobenzyl)-1*H*-imidazol-3-ium bromide (**34**). Yield 80%. Yellow powder, m.p.157-159 °C. IR ν_{max} (cm^{-1}): 3099, 2955, 2851, 1594, 1559, 1453, 1336, 1226, 1160, 1057, 880, 739, 648. ^{1}H NMR (400 MHz, DMSO) δ: 9.36 (1H, s), 8.15 (2H, d, J = 7.7 Hz), 7.87 (1H, s), 7.77 (1H, s), 7.68 (1H, d, J = 7.8 Hz), 7.62 (2H, d, J = 8.2 Hz), 7.46-7.36 (5H, m), 7.20 (2H, t, J = 7.4 Hz), 5.51 (2H, s), 4.46 (2H, t, J = 6.7 Hz), 4.27 (2H, t, J = 6.7 Hz), 1.90-1.87 (2H, m), 1.74-1.70 (2H, m). ^{13}C NMR (100 MHz, DMSO) δ: 140.33, 137.17, 133.96, 133.61, 131.52, 131.39, 128.96, 126.17, 123.62, 123.31, 123.25, 122.53, 120.79, 119.22, 109.72, 52.70, 49.14, 42.06, 27.56, 25.65. HRMS (ESI-TOF) m/z Calcd for $C_{26}H_{26}BrN_3$ $[M\text{-}Br]^+$ 458.1232, found 458.1226.

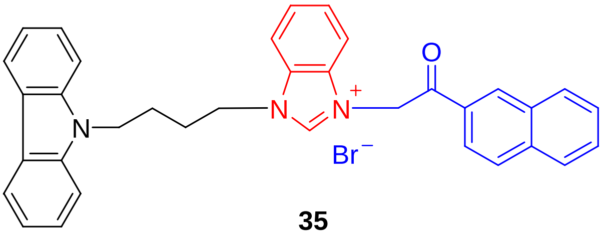

35

1-(4-(9*H*-carbazol-9-yl)butyl)-3-(2-(naphthalen-2-yl)-2-oxoethyl)-1*H*-benzo[*d*]imidazol-3-ium bromide (**35**). Yield 95%. White powder, m.p. 239-241 °C. IR ν_{max} (cm^{-1}): 3109, 3020, 2947, 2884, 1795, 1682, 1624, 1557, 1455, 1338, 1255, 1124, 1078, 933, 818, 753, 678. ^{1}H NMR (400 MHz, DMSO) δ: 9.81 (1H, s), 8.94 (1H, s), 8.24 (1H, d, J = 7.5 Hz), 8.16-8.04 (7H, m), 7.76-7.70 (4H, m), 7.65 (2H, t, J = 7.6 Hz), 7.45 (2H, t, J = 6.9 Hz), 7.20 (2H, t, J = 6.9 Hz), 6.55 (2H, s), 4.68-4.66 (2H, m), 4.51-4.49 (2H, m), 2.05-2.03 (2H, m), 1.92-1.90 (2H, m). ^{13}C NMR (100 MHz, DMSO) δ: 191.62, 143.84, 140.37, 136.05, 132.49, 131.44, 131.13, 130.20, 129.88, 129.19, 128.38, 127.90, 127.29, 127.08, 126.18, 123.81, 122.53, 120.78, 119.23, 114.50, 114.22, 109.79, 53.73, 47.11, 42.13, 26.76, 25.93. HRMS (ESI-TOF) m/z Calcd for $C_{35}H_{30}N_3O$ $[M-Br]^+$ 508.2383, found 508.2385.

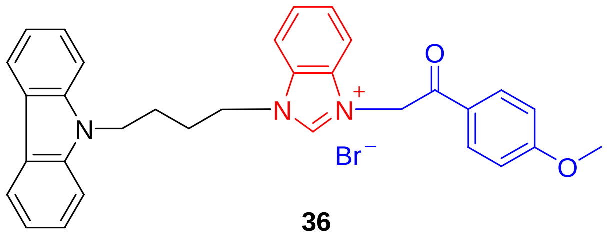

36

1-(4-(9*H*-carbazol-9-yl)butyl)-3-(2-(4-methoxyphenyl)-2-oxoethyl)-1*H*-benzo[*d*]imidazol-3-ium bromide (**36**). Yield 95%. White powder, m.p. 182-184 °C. IR ν_{max} (cm^{-1}): 3028, 2930, 2839, 1796, 1678, 1600, 1560, 1453, 1334, 1238, 1175, 1025, 983, 832, 754. ^{1}H NMR (300 MHz, DMSO) δ: 9.93 (1H, s), 8.16-8.13 (3H, m), 8.11-8.09 (3H, m), 7.66-7.64 (4H, m), 7.44 (2H, t, J = 7.5 Hz), 7.21-7.16 (4H, m), 6.44 (2H, s), 4.66-4.63 (2H, m), 4.50-4.46 (2H, m), 3.89 (3H, s), 2.04-2.01 (2H, m), 1.92-1.90 (2H, m). ^{13}C NMR (75 MHz, DMSO) δ: 189.94, 164.66, 143.76, 140.37, 132.43, 131.43, 131.07, 127.18, 127.01, 126.19, 122.53, 120.77, 119.23, 114.78, 114.48, 114.18, 109.83, 56.31, 53.46, 47.06, 42.15, 26.73, 25.89. HRMS (ESI-TOF) m/z Calcd for $C_{32}H_{30}N_3O_2$ $[M-Br]^+$ 488.2338, found 488.2332.

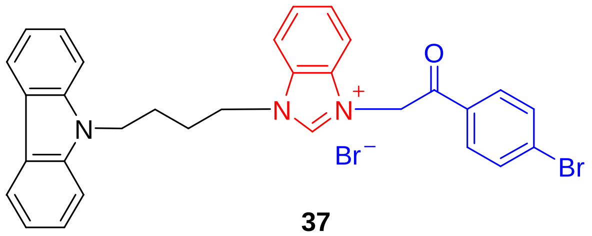

37

1-(4-(9*H*-carbazol-9-yl)butyl)-3-(2-(4-bromophenyl)-2-oxoethyl)-1*H*-benzo[*d*]imidazol-3-ium bromide (**37**). Yield 95%. White powder, m.p. 237-239 °C. IR ν_{max} (cm^{-1}): 3021, 2931, 2876, 1795, 1688, 1580, 1552, 1452, 1386, 1221, 1169, 1070, 984, 822, 753, 618. ^{1}H NMR (300 MHz, DMSO) δ: 9.80 (1H, s), 8.16-8.05 (6H, m), 7.89 (2H, d, J = 8.1 Hz), 7.69-7.64 (4H, m), 7.44 (2H, t, J = 7.5 Hz), 7.19 (2H, t, J = 7.3 Hz), 6.43 (2H, s), 4.67-4.64 (2H, m), 4.50-4.47 (2H, m), 2.03-2.01 (2H, m), 1.90-1.88 (2H, m). ^{13}C NMR (75 MHz, DMSO): δ 191.12, 143.71, 140.36, 133.24, 132.61, 132.41, 131.08, 130.89, 129.20, 127.24, 127.06, 126.17, 122.52, 120.77, 119.22, 114.54, 114.20, 109.80, 53.76, 47.09, 42.13, 26.75, 25.91. HRMS (ESI-TOF) m/z Calcd for $C_{31}H_{27}BrN_3O$ $[M-Br]^+$ 536.1338, found 536.1330.

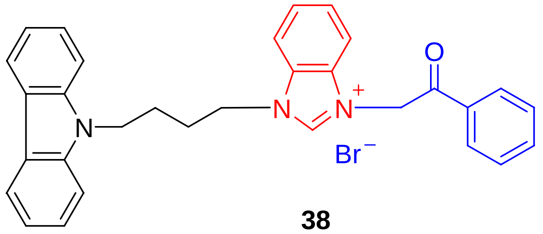

38

1-(4-(9*H*-carbazol-9-yl)butyl)-3-(2-oxo-2-phenylethyl)-1*H*-benzo[*d*]imidazol-3-ium bromide (**38**). Yield 95%. White powder, m.p. 179-181 °C. IR ν_{max} (cm^{-1}): 3024, 2936, 1795, 1692, 1596, 1563, 1452, 1337, 1229, 1180, 1074, 930, 823, 754, 615. ^{1}H NMR (300 MHz, DMSO) δ: 9.86 (1H, s), 8.16-8.11 (6H, m), 7.79 (1H, t, J = 7.2 Hz), 7.68-7.64 (6H, m), 7.45 (2H, t, J = 7.4 Hz), 7.20 (2H, t, J = 7.4 Hz), 6.47 (2H, s), 4.66 (2H, t, J = 6.7 Hz), 4.49 (2H, t, J = 6.7 Hz), 2.04-2.01 (2H, m), 1.91-1.90 (2H, m). ^{13}C NMR (75 MHz, DMSO) δ: 191.74, 143.74, 140.37, 135.06, 134.19, 132.43, 131.09, 129.53, 128.95, 127.23, 127.04, 126.18, 122.52, 120.77, 119.22, 114.53, 114.20,

109.82, 53.81, 47.08, 42.14, 26.74, 25.90. HRMS (ESI-TOF) m/z Calcd for $C_{31}H_{28}N_3O$ [M-Br]$^+$ 458.2232, found 458.2230.

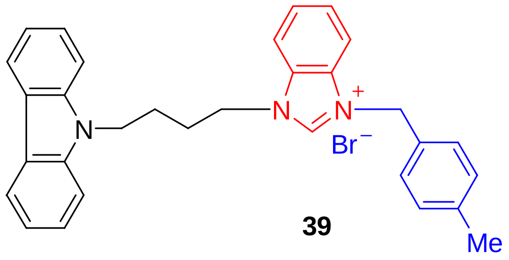

39

1-(4-(9*H*-carbazol-9-yl)butyl)-3-(4-methylbenzyl)-1*H*-benzo[*d*]imidazol-3-ium bromide (**39**). Yield 95%. White powder, m.p. 196-198 °C. IR ν_{max} (cm^{-1}): 3113, 3023, 1815, 1599, 1559, 1453, 1376, 1216, 1180, 1024, 754, 610. ^{1}H NMR (400 MHz, DMSO) δ: 10.12 (1H, s), 8.14 (2H, d, J = 7.6 Hz), 8.05 (1H, t, J = 3.2 Hz), 7.96 (1H, t, J = 5.2 Hz), 7.66-7.60 (4H, m), 7.45-7.39 (4H, m), 7.19 (2H, t, J = 7.4 Hz), 7.13 (2H, t, J = 7.6 Hz), 5.73 (2H, s), 4.57-4.54 (2H, m), 4.49-4.46 (2H, m), 2.25 (3H, m), 2.02-2.01 (2H, m), 1.87-1.85 (2H, m). ^{13}C NMR (100 MHz, DMSO) δ: 142.71, 140.34, 138.55, 131.69, 131.46, 131.20, 129.92, 128.96, 127.06, 126.15, 122.52, 120.77, 119.20, 114.41, 114.30, 109.79, 50.10, 47.03, 42.16, 26.57, 25.96, 21.17. HRMS (ESI-TOF) m/z Calcd for $C_{31}H_{30}N_3$ [M-Br]$^+$ 444.2440, found 444.2427.

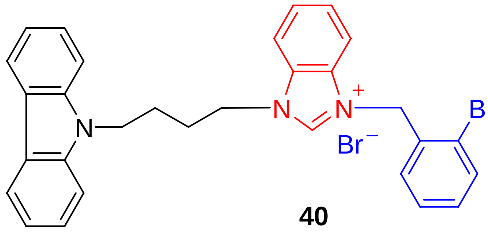

40

1-(4-(9*H*-carbazol-9-yl)butyl)-3-(2-bromobenzyl)-1*H*-benzo[*d*]imidazol-3-ium bromide (4**0**). Yield 95%. Yellow powder, m.p.100-102 °C. IR ν_{max} (cm^{-1}): 3117, 3043, 2942, 1600, 1563, 1453, 1335, 1226, 1024, 753, 665, 615. ^{1}H NMR (300 MHz, DMSO) δ: 9.93 (1H, s), 8.14 (2H, d, J = 7.7 Hz), 8.09 (1H, d, J = 8.1 Hz), 7.88 (1H, t, J = 7.9 Hz), 7.69 (1H, d, J = 7.7 Hz), 7.65-7.61 (4H, m), 7.44-7.35 (5H, m), 7.19 (2H,

t, J = 7.4 Hz), 5.81 (2H, s), 4.59 (2H, t, J = 6.7 Hz), 4.47 (2H, t, J = 6.7 Hz), 2.02-1.98 (2H, m), 1.87-1.84 (2H, m). ^{13}C NMR (100 MHz, DMSO) δ: 143.42, 140.34, 133.76, 133.00, 131.57, 131.49, 131.37, 128.89, 127.33, 127.18, 126.15, 123.59, 122.51, 120.77, 119.21, 114.45, 114.26, 109.77, 50.93, 47.08, 42.13, 26.76, 25.93. HRMS (ESI-TOF) m/z Calcd for $C_{30}H_{27}BrN_3$ [M-Br]$^+$ 508.1383, found 508.1382.

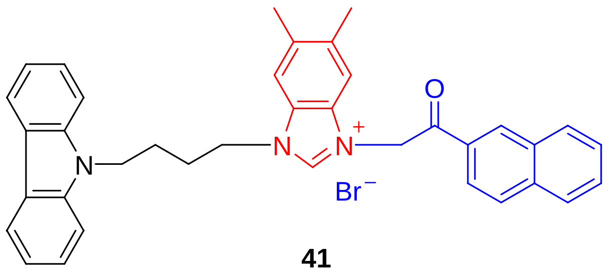

41

1-(4-(9*H*-carbazol-9-yl)butyl)-5,6-dimethyl-3-(2-(naphthalen-2-yl)-2-oxoethyl)-1*H*-benzo[*d*]imidazol-3-ium bromide (**41**). Yield 95%. White powder, m.p.249-251 °C. IR ν_{max} (cm^{-1}): 3024, 2949, 1808, 1685, 1625, 1593, 1562, 1454, 1336, 1217, 1186, 1011, 933, 858, 753, 617. ^{1}H NMR (300 MHz, DMSO) δ: 9.58 (1H, s), 8.86 (1H, s), 8.15 (1H, d, J = 7.9 Hz), 8.05 (3H, d, J = 8.0 Hz), 7.97 (2H, t, J = 10.0 Hz), 7.81 (1H, s), 7.76 (1H, s), 7.69-7.61 (2H, m), 7.55 (2H, d, J = 8.2 Hz), 7.36 (2H, t, J = 7.5 Hz), 7.11 (2H, t, J = 7.4 Hz), 6.40 (2H, s), 4.51 (2H, t, J = 6.3 Hz), 4.40 (2H, t, J = 6.6 Hz), 2.31 (3H, s), 2.27 (3H, s), 1.94-1.91 (2H, m), 1.82-1.80 (2H, m). ^{13}C NMR (75 MHz, DMSO) δ: 191.63, 142.51, 140.35, 136.99, 136.78, 136.03, 132.50, 131.48, 131.42, 130.96, 130.19, 129.85, 129.52, 129.16, 128.37, 127.88, 126.16, 123.80, 122.51, 120.75, 119.21, 113.91, 113.56, 109.78, 53.62, 46.98, 42.09, 26.66, 25.83, 20.45. HRMS (ESI-TOF) m/z Calcd for $C_{37}H_{34}N_3O$ [M-Br]$^+$ 536.2696, found 536.2697.

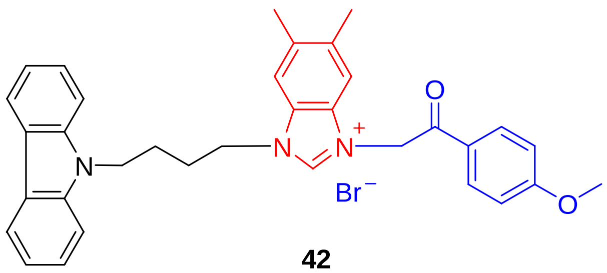

42

1-(4-(9*H*-carbazol-9-yl)butyl)-3-(2-(4-methoxyphenyl)-2-oxoethyl)-5,6-dimethyl-1*H*-benzo[*d*]imidazol-3-ium bromide (**42**). Yield 96%. White powder, m.p. 156-158 °C. IR ν_{max} (cm^{-1}): 3051, 3015, 2936, 1683, 1599, 1565, 1454, 1336, 1239, 1176, 1016, 955, 838, 755, 601. ^{1}H NMR (300 MHz, DMSO) δ: 9.62 (1H, s), 8.13 (2H, d, J = 7.7 Hz), 8.09 (2H, d, J = 8.5 Hz), 7.83-7,82 (2H, m), 7.63 (2H, d, J = 8.2 Hz), 7.43 (2H, t, J = 7.4 Hz), 7.21-7.17 (4H, m), 6.28 (2H, s), 4.57 (2H, t, J = 6.0 Hz), 4.47 (2H, t, J = 6.6 Hz), 3.90 (3H, s), 2.38 (3H, s), 2.34 (3H, s), 1.99-1.97 (2H, m), 1.88-1.87 (2H, m). ^{13}C NMR (75 MHz, DMSO) δ: 189.91, 164.66, 142.49, 140.34, 136.92, 136.72, 131.35, 130.92, 129.48, 127.01, 126.14, 122.50, 120.73, 119.19, 114.77, 113.83, 113.52, 109.77, 56.29, 53.16, 46.92, 42.07, 26.65, 25.80, 20.42. HRMS (ESI-TOF) m/z Calcd for $C_{34}H_{34}N_3O_2$ $[M-Br]^+$ 516.2646, found 516.2648.

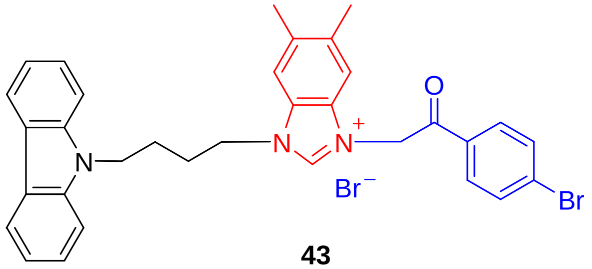

43

1-(4-(9*H*-carbazol-9-yl)butyl)-3-(2-(4-bromophenyl)-2-oxoethyl)-5,6-dimethyl-1*H*-benzo[*d*]imidazol-3-ium bromide (**43**). Yield 94%. White powder, m.p. 230-232 °C. IR ν_{max} (cm^{-1}): 3015, 2934, 1694, 1582, 1454, 1336, 1229, 1180, 1071, 957, 819, 753, 611. ^{1}H NMR (300 MHz, DMSO) δ: 8.03-7.95 (4H, m), 7.78-7.64 (3H, m), 7.44-7.37 (8H, m), 7.32-7.24 (2H, m), 7.09-7.07 (2H, m), 6.01 (2H, s), 3.81 (3H, s), 2.52 (3H, s). ^{13}C NMR (75 MHz, DMSO) δ: 191.18,142.48, 140.40, 137.03, 136.84, 133.31, 132.67, 130.92, 129.55, 129.23, 126.21, 122.57, 120.81, 119.27, 114.00, 113.63, 109.84, 53.65, 47.03, 46.13, 42.14, 26.72, 25.88, 20.53. HRMS (ESI-TOF) m/z Calcd for $C_{33}H_{31}$ BrN_3 $[M-Br]^+$ 564.1645, found 564.1638.

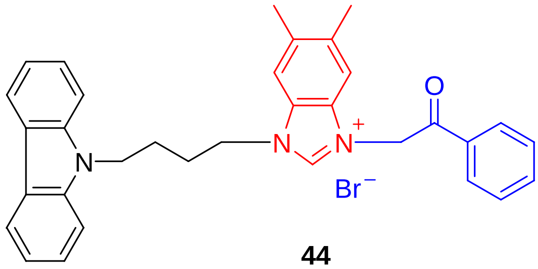

44

1-(4-(9*H*-carbazol-9-yl)butyl)-5,6-dimethyl-3-(2-oxo-2-phenylethyl)-1*H*-benzo[*d*]imidazol-3-ium bromide (**44**). Yield 90%. White powder, m.p. 152-153 °C. IR ν_{max} (cm^{-1}): 3121, 3043, 2936, 1694, 1599, 1564, 1453, 1337, 1230, 1187, 1001, 955, 848, 755, 612. ^{1}H NMR (300 MHz, DMSO) δ: 9.63 (1H, s), 8.13 (4H, t, J = 7.2 Hz), 7.85 (2H, d, J = 4.9 Hz), 7.80-7.78 (1H, m), 7.68-7.62 (4H, m), 7.44 (2H, t, J = 7.6 Hz), 7.19 (2H, t, J = 7.4 Hz), 6.36 (2H, s), 4.59 (2H, t, J = 6.6 Hz), 4.48 (2H, t, J = 6.8 Hz), 2.39 (3H, s), 2.35 (3H, s), 2.01-1.98 (2H, m), 1.89-1.87 (2H, m). ^{13}C NMR (75 MHz, DMSO) δ: 191.71, 142.43, 140.34, 136.95, 136.75, 135.04, 134.18, 130.91, 129.51, 128.90, 126.14, 122.50, 120.74, 119.19, 113.91, 113.54, 109.77, 53.60, 46.95, 42.08, 26.66, 25.81, 20.45. HRMS (ESI-TOF) m/z Calcd for $C_{33}H_{32}N_3O$ $[M-Br]^+$ 486.2545, found 486.2535.

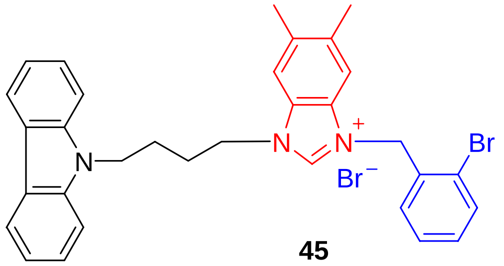

45

1-(4-(9*H*-carbazol-9-yl)butyl)-3-(2-bromobenzyl)-5,6-dimethyl-1*H*-benzo[*d*]imidazol-3-ium bromide (**45**). Yield 85%. Yellow powder, m.p. 129-131 °C. IR ν_{max} (cm^{-1}): 3137, 3047, 2939, 1600, 1562, 1453, 1337, 1228, 1184, 1021, 947, 844, 755, 608. ^{1}H NMR (300 MHz, DMSO) δ: 9.69 (1H, s), 8.12 (2H, d, J = 7.7 Hz), 7.82 (1H, s), 7.70 (1H, d, J = 7.5 Hz), 7.66 (1H, s), 7.60 (2H, d, J = 8.2 Hz), 7.43-7.37 (4H, m), 7.34-7.32 (1H, m), 7.18 (2H, t, J = 7.5 Hz), 5.71 (2H, s), 4.50 (2H, t, J = 6.6 Hz), 4.45

(2H, t, J = 6.8 Hz), 2.36 (3H, s), 2.34 (3H, s), 1.98-1.95 (2H, m), 1.84-1.82 (2H, m). ^{13}C NMR (75 MHz, DMSO) δ: 142.03, 140.31, 137.06, 136.99, 133.72, 133.20, 131.38, 130.97, 130.00, 129.91, 128.88, 126.11, 123.44, 122.49, 120.73, 119.17, 113.80, 113.62, 109.73, 50.71, 46.96, 42.06, 26.64, 25.82, 20.49, 20.45. HRMS (ESI-TOF) m/z Calcd for $C_{32}H_{32}BrN_3$ $[M-Br]^+$ 536.1701, found 536.1698.

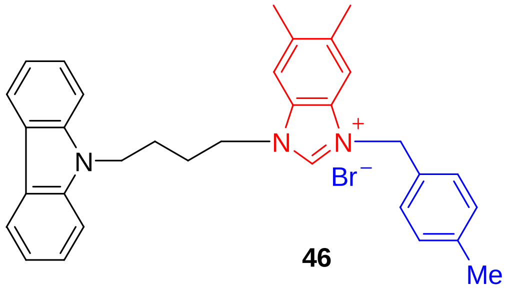

46

1-(4-(9*H*-carbazol-9-yl)butyl)-5,6-dimethyl-3-(4-methylbenzyl)-1*H*-benzo[*d*]imidazol-3-ium bromide (**46**). Yield 86%. White powder, m.p. 129-131 °C. IR ν_{max} (cm^{-1}): 3128, 3041, 2936, 1599, 1559, 1454, 1337, 1228, 1183, 1012, 944, 840, 755, 609. ^{1}H NMR (400 MHz, $CDCl_3$) δ: 9.87 (1H, s), 8.13 (2H, d, J = 7.7 Hz), 7.76 (1H, s), 7.73 (1H, s), 7.61 (2H, d, J = 8.2 Hz), 7.42 (2H, t, J = 7.5 Hz), 7.35-7.32 (2H, m), 7.18 (2H, t, J = 7.5 Hz), 7.12 (2H, d, J = 7.7 Hz), 5.62 (2H, s), 4.50-4.44 (4H, m), 2.34 (6H, s), 2.25 (3H, s), 2.00-1.97 (2H, m), 1.84-1.81 (2H, m). ^{13}C NMR (100 MHz, $CDCl_3$) δ: 141.34, 140.29, 138.47, 136.84, 132.90, 131.62, 130.01, 129.91, 129.70, 128.54, 126.12, 122.48, 120.73, 119.18, 113.72, 113.65, 109.72, 52.32, 49.86, 46.91, 40.17, 26.46, 25.84, 21.15, 20.47, 20.43. HRMS (ESI-TOF) m/z Calcd for $C_{34}H_{34}N_3$ $[M-Br]^+$ 472.2747, found 472.2742.

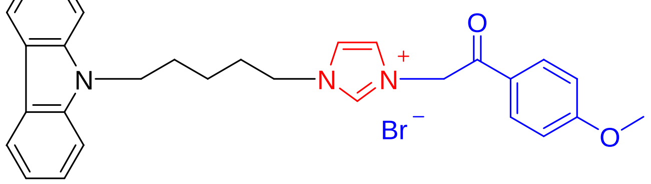

47

1-(5-(9*H*-carbazol-9-yl)pentyl)-3-(2-(4-methoxyphenyl)-2-oxoethyl)-1*H*-imidazol-3-ium bromide (**47**). Yield 95%. Yellow oil. IR ν_{max} (cm^{-1}): 3137, 3054, 2936, 1685, 1599, 1454, 1335, 1241, 1167, 1023, 983, 835, 755, 628. ^{1}H NMR (300 MHz, MeOH) δ: 8.80 (1H, s), 8.02 (2H, d, J = 7.8Hz), 7.95 (2H, d, J = 8.7 Hz), 7.45 (1H, s), 7.42-7.35 (5H, m), 7.17-7.12 (2H, 3), 6.98 (2H, d, J = 8.7 Hz), 5.75 (2H, s), 4.21 (2H, t, J = 13.5 Hz) , 3.92 (2H, t, J = 7.2 Hz), 3.77 (3H, s), 1.78-1.70 (2H, m), 1.67-1.59 (2H, m), 1.19-1.18 (2H, m). ^{13}C NMR (75 MHz, MeOH) δ: 190.06, 166.10, 141.57, 138.29, 131.75, 127.64, 126.85, 125.22, 123.85, 122.83, 121.22, 119.95, 115.30, 110.13, 56.27, 56.03, 50.46, 43.29, 30.53, 29.18, 24.58. HRMS (ESI-TOF) m/z Calcd for $C_{29}H_{30}N_3O_2$ $[M-Br]^+$ 452.2333, found 452.2327.

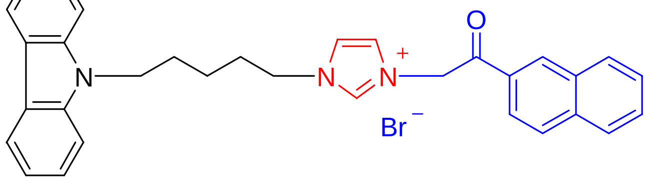

48

1-(5-(9*H*-carbazol-9-yl)pentyl)-3-(2-(naphthalen-2-yl)-2-oxoethyl)-1*H*-imidazol-3-ium bromide (**48**). Yield 95%. White powder, m.p. 116-118 °C. IR ν_{max} (cm^{-1}): 3137, 3051, 2939, 1693, 1625, 1564, 1455, 1335, 1223, 1166, 98, 822, 753, 628. ^{1}H NMR (300 MHz, DMSO) δ: 9.26 (1H, s), 8.87 (1H, s), 8.21-8.06 (6H, m), 7.88-7.81 (2H, m), 7.72-7.62 (4H, m), 7.47-7.45 (2H, m), 7.21-7.20 (2H, m), 6.26 (2H, s), 4.42-4.26 (4H, m), 1.86-1.84 (4H, m), 1.36-1.34 (2H, m). ^{13}C NMR (75 MHz, DMSO) δ: 191.76, 140.42, 137.76, 136.01, 132.52, 131.45, 131.06, 130.17, 129.82, 129.27, 128.36, 127.87, 126.17, 124.60, 123.66, 122.52, 120.76, 119.16, 109.75, 55.97, 49.22, 42.51, 29.75, 28.36, 23.58. HRMS (ESI-TOF) m/z Calcd for $C_{32}H_{30}N_3O$ $[M-Br]^+$ 472.2383, found 472.2386.

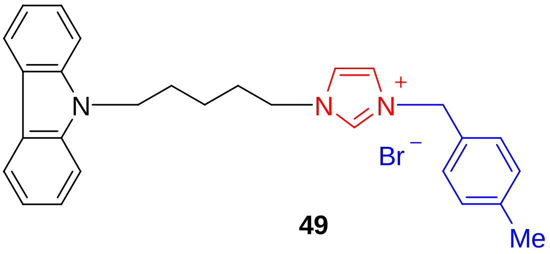

49

1-(5-(9*H*-carbazol-9-yl)pentyl)-3-(4-methylbenzyl)-1*H*-imidazol-3-ium bromide (**49**). Yield 80%. Yellow oil. IR ν_{max} (cm^{-1}): 3129, 3048, 2937, 1600, 1557, 1454, 1334, 1227, 1154, 1026, 831, 755, 627. ^{1}H NMR (300 MHz, MeOH) δ: 8.89 (1H, s), 8.01 (2H, d, J = 7.8 Hz), 7.38-7.37 (5H, m), 7.27 (1H, s), 7.23-7.21 (2H, m), 7.17-7.11 (4H, m), 5.20 (2H, s), 4.20 (2H, t, J = 6.6 Hz) , 3.87 (2H, t, J = 7.2 Hz), 2.27 (3H, s), 1.75-1.70 (2H, m), 1.64-1.59 (2H, m), 1.16-1.11 (2H, m). ^{13}C NMR (75 MHz, MeOH) δ: 141.71, 140.50, 136.74, 132.15, 131.01, 129.79, 126.94, 123.97, 123.78, 123.51, 121.29, 120.06, 110.23, 53.87, 50.58, 43.33, 30.62, 29.29, 24.73, 21.35. HRMS (ESI-TOF) m/z Calcd for $C_{28}H_{30}N_3$ [M-Br]$^+$ 408.2434, found 408.2436.

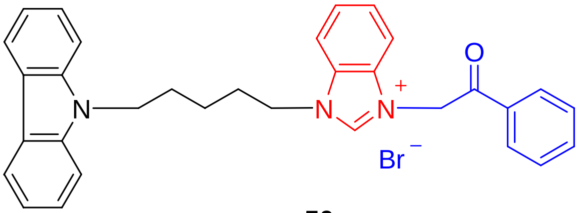

50

1-(5-(9*H*-carbazol-9-yl)pentyl)-3-(2-oxo-2-phenylethyl)-1*H*-benzo[*d*]imidazol-3-ium bromide (**50**). Yield 90%. White powder, m.p. 225-227 °C. IR ν_{max} (cm^{-1}): 3129, 3027, 2937, 1695, 1598, 1565, 1452, 1336, 1222, 1115, 987, 753, 690. ^{1}H NMR (300 MHz, DMSO) δ: 9.89 (1H, s), 8.18-8.06 (6H, m), 7.80 (1H, t, J = 5.4 Hz), 7.70-7.66 (4H, m), 7.60 (2H, d, J = 6.2 Hz), 7.43 (2H, t, J = 5.6 Hz), 7.19 (2H, t, J = 5.6 Hz), 6.51 (2H, s), 4.56 (2H, t, J = 5.1 Hz), 4.40 (2H, t, J = 4.8 Hz), 1.95 (2H, t, J = 6.0 Hz), 1.86 (2H, d, J = 6.0 Hz), 1.45-1.43 (2H, m).^{13}C NMR (75 MHz, DMSO) δ: 191.78, 143.71, 140.41, 135.08, 134.22, 132.41, 131.13, 129.54, 128.96, 127.21, 127.02, 126.15, 122.51,

120.74, 119.14, 114.48, 114.21, 109.72, 53.80, 47.12, 42.56, 28.96, 28.46, 23.90. HRMS (ESI-TOF) m/z Calcd for $C_{32}H_{30}N_3O$ $[M-Br]^+$ 472.2383, found 472.2383.

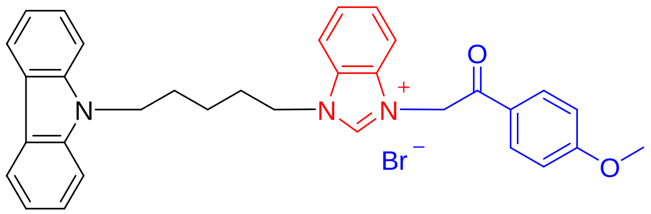

51

1-(5-(9*H*-carbazol-9-yl)pentyl)-3-(2-(4-methoxyphenyl)-2-oxoethyl)-1*H*-benzo[*d*]imidazol-3-ium bromide (**51**). Yield 94%. White powder, m.p. 131-133 °C. IR ν_{max} (cm^{-1}): 3137, 3011, 2936, 2323, 1684, 1600, 1566, 1454, 1336, 1238, 1174, 1022, 984, 836, 755. ^{1}H NMR (300 MHz, DMSO) δ: 9.86 (1H,s), 8.15-8.13 (4H, m), 8.08-8.04 (2H, m), 7.67-7.65 (2H, m), 7.60-7.58 (2H, m), 7.45-7.41 (2H, m), 7.20-7.21 (4H, m), 6.42 (2H, s), 4.45 (2H, t, J = 6.0 Hz), 4.39 (2H, d, J = 6.0 Hz), 3.90 (3H, s), 1.96-1.91 (2H, m), 1.86-1.81 (2H, m), 1.44-1.42 (2H, m). ^{13}C NMR (75 MHz, DMSO) δ: 189.94, 164.70, 143.74, 140.40, 132.41, 131.11, 127.19, 127.02, 126.15, 122.50, 120.73, 119.14, 114.81, 114.40, 114.17, 109.70, 56.31, 53.34, 47.10, 42.55, 28.94, 28.44, 23.89. HRMS (ESI-TOF) m/z Calcd for $C_{33}H_{32}N_3O_2$ $[M-Br]^+$ 502.2489, found 502.2489.

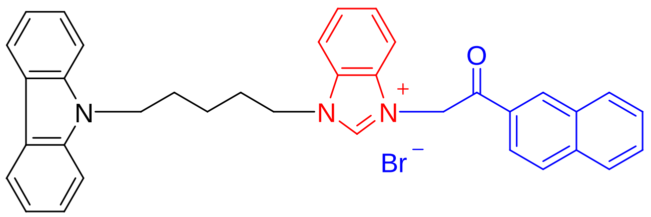

52

1-(5-(9*H*-carbazol-9-yl)pentyl)-3-(2-(4-methoxyphenyl)-2-oxoethyl)-1*H*-benzo[*d*]imidazol-3-ium bromide (**52**). Yield 90%. White powder, m.p. 120-122 °C. IR ν_{max} (cm^{-1}): 3145, 3039, 2936, 2855, 1688, 1617, 1564, 1454, 1336, 1220, 1185, 997, 936, 821,

753. ^{1}H NMR (300 MHz, DMSO) δ: 9.87 (1H, s), 8.99 (1H, s), 8.25 (1H, d, J = 7.8 Hz), 8.15 (4H, d, J = 6.9 Hz), 8.09 (3H, d, J = 7.7 Hz), 7.78-7.71 (2H, m), 7.70-7.67 (2H, m), 7.60 (2H, d, J = 8.2 Hz), 7.44 (2H, t, J = 7.4 Hz), 7.19 (2H, t, J = 7.4 Hz), 6.60 (2H, s), 4.58 (2H, t, J = 6.9 Hz), 4.42 (2H, t, J = 6.6 Hz), 1.98 (2H, t, J = 6.7 Hz), 1.87 (2H, t, J = 7.0 Hz), 1.46-1.45 (2H, m). ^{13}C NMR (75 MHz, DMSO) δ: 191.67, 143.79, 140.42, 136.07, 132.52, 132.46, 131.51, 131.18, 130.21, 129.88, 129.20, 128.38, 127.90, 127.25, 127.06, 126.15, 123.82, 122.51, 120.75, 119.14, 114.46, 114.25, 109.71, 53.76, 47.16, 42.57, 28.98, 28.47, 23.93. HRMS (ESI-TOF) m/z Calcd for $C_{36}H_{32}N_3O$ [M-Br]$^+$ 522.2540, found 522.2538.

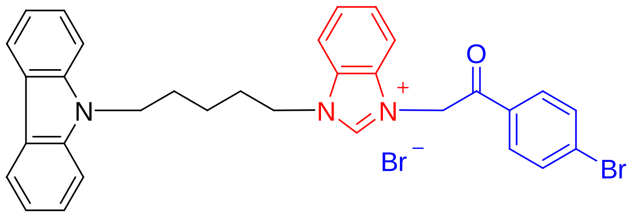

53

1-(5-(9*H*-carbazol-9-yl)pentyl)-3-(2-(4-bromophenyl)-2-oxoethyl)-1*H*-benzo[*d*]imidazol-3-ium bromide (**53**). Yield 94%. White powder, m.p. 187-189 °C. IR ν_{max} (cm^{-1}): 3011, 2962, 2925, 1694, 1583, 1452, 1387, 1335, 1225, 1200, 1070, 985, 820, 750, 624. ^{1}H NMR (400 MHz, DMSO) δ: 9.83 (1H, s), 8.15 (2H, d, J = 7.7 Hz), 8.10 (4H, d, J = 6.5 Hz), 7.91 (2H, d, J = 8.3 Hz), 7.69-7.66 (2H, m), 7.60 (2H, d, J = 8.2 Hz), 7.44 (2H, t, J = 7.4 Hz), 7.19 (2H, t, J = 7.4 Hz), 6.47 (2H, s), 4.57 (2H, t, J = 6.9 Hz), 4.41 (2H, t, J = 6.6 Hz), 1.98 (2H, t, J = 6.7 Hz), 1.87 (2H, t, J = 7.0 Hz), 1.46-1.45 (2H, m). ^{13}C NMR (100 MHz, DMSO) δ: 191.16, 143.67, 140.41, 133.27, 132.63, 132.38, 131.13, 130.91, 129.22, 127.22, 127.04, 126.14, 122.50, 120.74, 119.13, 114.48, 114.22, 109.71, 53.75, 47.14, 42.56, 28.97, 28.47, 23.91. HRMS (ESI-TOF) m/z Calcd for $C_{32}H_{29}BrN_3O$ [M-Br]$^+$ 550.1489, found 550.1484.

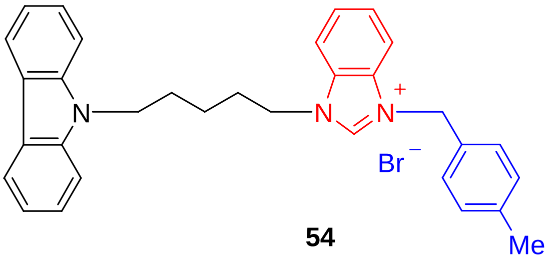

54

1-(5-(9*H*-carbazol-9-yl)pentyl)-3-(4-methylbenzyl)-1*H*-benzo[*d*]imidazol-3-ium bromide (**54**). Yield 90%. White powder, m.p. 193-195 °C. IR ν_{max} (cm^{-1}): 3117, 3020, 2933, 2864, 2323, 1600, 1557, 1453, 1376, 1335, 1218, 1180, 1023, 844, 756.^{1}H NMR (400 MHz, DMSO) δ: 10.08 (1H, s), 8.15 (2H, d, J = 7.7 Hz), 8.02-8.00 (1H,m), 7.98-7.96 (1H, m), 7.64-7.62 (2H, m), 7.60-7.57 (2H, m), 7.44-7.41 (4H, m), 7.21-7.17 (4H, m), 5.74 (2H, s), 4.46 (2H, t, J = 7.2 Hz), 4.40 (2H, t, J = 6.8 Hz), 2.28 (3H, s), 1.99-1.92 (2H, m), 1.88-1.81 (2H, m), 1.45-1.37 (2H, m). ^{13}C NMR (100 MHz, DMSO) δ: 142.68, 140.40, 138.60, 131.74, 131.46, 131.19, 129.95, 128.82, 127.04, 126.12, 122.49, 120.73, 119.13, 114.35, 114.31, 109.69, 50.13, 47.09, 42.53, 28.89, 28.51, 23.99, 21.18. HRMS (ESI-TOF) m/z Calcd for $C_{32}H_{32}N_3$ [M-Br]$^+$ 458.2591, found 458.2592.

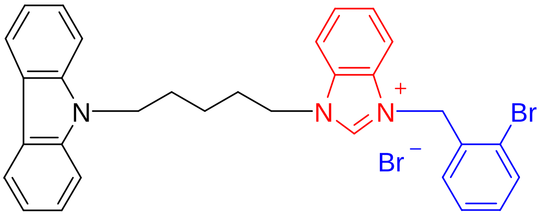

55

1-(5-(9*H*-carbazol-9-yl)pentyl)-3-(2-bromobenzyl)-1*H*-benzo[*d*]imidazol-3-ium bromide (**55**). Yield 90%. White powder, m.p. 171-173 °C. IR ν_{max} (cm^{-1}): 3121, 3043, 3015, 2936, 2864, 2323, 1600, 1562, 1453, 1376, 1335, 1222, 1024, 754, 614. ^{1}H NMR (400 MHz, MeOH) δ: 9.97 (1H, s), 8.15 (2H, d, J = 8.0 Hz), 8.08-8.06 (1H, m), 7.93-7.91 (1H, m), 7.75 (1H, d, J = 8.0 Hz), 7.67-7.65 (2H, m), 7.58 (2H, d, J = 8.0 Hz), 7.46-7.37 (5H, m), 7.19 (2H, t, J = 8.0 Hz), 6.60 (2H, s), 4.51 (2H, t, J = 6.9 Hz),

4.40 (2H, t, J = 6.6 Hz), 1.95 (2H, t, J = 6.7 Hz), 1.85 (2H, t, J = 7.0 Hz), 1.42-1.41 (2H, m)。 ^{13}C NMR (100 MHz, MeOH) δ: 143.37, 140.39, 133.77, 133.03, 131.61, 131.51, 131.36, 128.92, 127.31, 127.18, 126.12, 123.61, 122.49, 120.74, 119.12, 114.47, 114.22, 109.69, 50.90, 47.14, 42.54, 29.01, 28.51 23.92. HRMS (ESI-TOF) m/z Calcd for $C_{31}H_{29}BrN_3$ $[M-Br]^+$ 522.1545, found 522.1542.

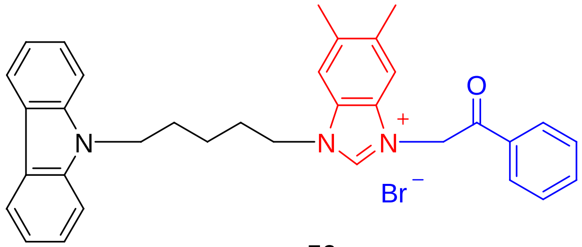

56

1-(5-(9*H*-carbazol-9-yl)pentyl)-5,6-dimethyl-3-(2-oxo-2-phenylethyl)-1*H*-benzo[*d*]imidazol-3-ium bromide (**56**). Yield 90%. White powder, m.p. 261-263 °C. IR ν_{max} (cm^{-1}): 3127, 3031, 2944, 1696, 1597, 1565, 1482, 1452, 1336, 1222, 1151, 999, 958, 848, 755, 613. ^{1}H NMR (400 MHz, DMSO) δ: 9.66 (1H, s), 8.15 (4H, t, J = 5.2 Hz), 7.87 (2H, d, J = 4.0 Hz), 7.80 (1H, t, J = 7.4 Hz), 7.68 (2H, t, J = 7.6 Hz), 7.59 (2H, d, J = 8.2 Hz), 7.42 (2H, t, J = 7.2 Hz), 7.19 (2H, t, J = 7.6 Hz), 6.39 (2H, s), 4.50 (2H, t, J = 7.6 Hz), 4.40 (2H, t, J = 7.6 Hz), 2.40 (3H, s), 2.36 (3H, s), 1.96-1.92 (2H, m), 1.87-1.83 (2H, m), 1.42-1.40 (2H, m). ^{13}C NMR (100 MHz, DMSO) δ: 191.76, 142.41, 140.40, 136.93, 136.77, 135.07, 134.22, 130.90, 129.61, 129.54, 128.92, 126.11, 122.49, 120.72, 119.11, 113.87, 113.63, 109.69, 53.59, 46.98, 42.56, 28.94, 28.46, 23.86, 20.44. HRMS (ESI-TOF) m/z Calcd for $C_{34}H_{34}N_3O$ $[M-Br]^+$ 500.2696, found 500.2691.

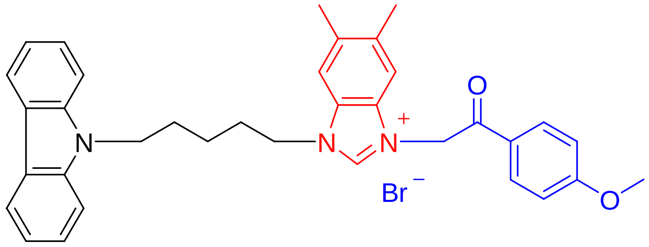

57

1-(5-(9*H*-carbazol-9-yl)pentyl)-3-(2-(4-methoxyphenyl)-2-oxoethyl)-5,6-dimethyl-1*H*-benzo[*d*]imidazol-3-ium bromide (**57**). Yield 95%. White powder, m.p. 228-230 °C. IR ν_{max} (cm^{-1}): 3125, 2938, 1684, 1600, 1566, 1454, 1334, 1240, 1177, 1017, 958, 840, 754, 600. ^{1}H NMR (400 MHz, DMSO) δ: 9.70 (1H, s), 8.14 (4H, d, J = 7.9 Hz), 7.86 (2H, s), 7.59 (2H, d, J = 8.2 Hz), 7.42 (2H, t, J = 7.4 Hz), 7.21-7.17 (4H, m), 6.35 (2H, s), 4.49 (2H, d, J =6.7 Hz), 4.39 (2H, d, J =6.4 Hz), 3.91 (3H, s), 2.40 (3H, s), 2.36 (3H, s), 1.96-1.92 (2H, m), 1.87-1.83 (2H, m), 1.41-1.39 (2H, m). ^{13}C NMR (100 MHz, DMSO) δ: 189.97, 164.68, 142.45, 140.39, 136.88, 136.72, 131.39, 130.91, 129.59, 127.05, 126.11, 122.49, 120.71, 119.10, 114.79, 113.82, 113.60, 109.68, 56.31, 53.21, 46.95, 42.56, 28.92, 28.46, 23.84, 20.42. HRMS (ESI-TOF) m/z Calcd for $C_{35}H_{36}N_3O_2$ [M-Br]$^+$ 530.2802, found 530.2795.

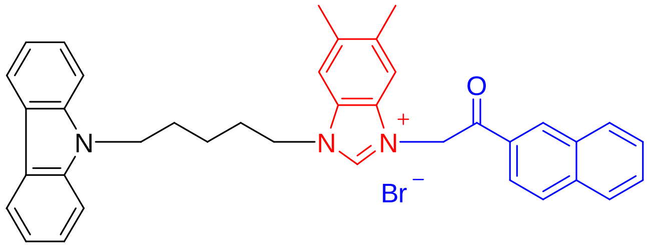

58

1-(5-(9*H*-carbazol-9-yl)pentyl)-5,6-dimethyl-3-(2-(naphthalen-2-yl)-2-oxoethyl)-1*H*-benzo[*d*]imidazol-3-ium bromide (**58**). Yield 96%. White powder, m.p. 205-207 °C. IR ν_{max} (cm^{-1}): 3035, 2933, 1686, 1625, 1564, 1454, 1335, 1221, 1188, 1010, 933, 827, 752, 602. ^{1}H NMR (400 MHz, DMSO) δ: 9.55 (1H, s), 8.87 (1H, s), 8.16 (1H, d, J = 7.9 Hz), 8.09-8.06 (3H, m), 8.02-7.99 (2H, m), 7.81 (2H, d, J = 8.5 Hz), 7.71-7.62 (2H, m), 7.52 (2H, d, J = 8.0 Hz), 7.35 (2H, t, J = 8.0 Hz), 7.11 (2H, t, J = 8.0 Hz), 6.40 (2H, s), 4.44 (2H, d, J = 7.2 Hz), 4.34 (2H, d, J =6.8 Hz), 2.33 (3H, s), 2.30 (3H, s), 1.91-1.83 (2H, m), 1.81-1.76 (2H, m), 1.38-1.31 (2H, m). ^{13}C NMR (100 MHz, DMSO) δ: 191.65, 142.50, 140.41, 136.99, 136.81, 136.06, 132.51, 131.51, 131.39, 130.95, 130.19, 129.88, 129.66, 129.20, 128.39, 127.91, 126.12, 123.81, 122.50, 120.74, 119.13, 113.85, 113.67, 109.68, 53.55, 47.02, 42.57, 28.95, 28.47, 23.89,

20.45. HRMS (ESI-TOF) m/z Calcd for $C_{38}H_{36}N_3O$ $[M-Br]^+$ 550.2853, found 550.2848.

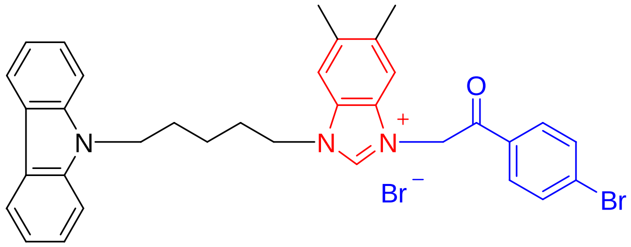

59

1-(5-(9H-carbazol-9-yl)pentyl)-3-(2-(4-bromophenyl)-2-oxoethyl)-5,6-dimethyl-1H-benzo[d]imidazol-3-ium bromide (**59**). Yield 96%. Yellow powder, m.p. 196-198 °C. IR ν_{max} (cm^{-1}): 3015, 2926, 1694, 1582, 1452, 1386, 1335, 1227, 1069, 1009, 958, 821, 748, 612. ^1H NMR (400 MHz, DMSO) δ: 9.63 (1H, s), 8.14 (2H, d, J = 7.7 Hz), 8.07 (2H, d, J = 8.3 Hz), 7.92-7.86 (4H, m), 7.58 (2H, d, J = 8.2 Hz), 7.43 (2H, t, J = 7.4 Hz), 7.19 (2H, t, J = 7.5 Hz), 6.37 (2H, s), 4.49 (2H, t, J = 7.0 Hz), 4.40 (2H, t, J = 6.8 Hz), 2.40 (3H, s), 2.36 (3H, s), 1.96-1.92 (2H, m), 1.87-1.83 (2H, m), 1.42-1.40 (2H, m). ^{13}C NMR (100 MHz, DMSO) δ: 191.16, 142.36, 140.40, 136.94, 136.97, 133.27, 132.62, 130.87, 129.60, 129.20, 126.11, 122.49, 120.72, 119.11, 113.89, 113.63, 109.68, 53.57, 47.00, 42.56, 28.94, 28.46, 23.86, 23.86, 20.44. HRMS (ESI-TOF) m/z Calcd for $C_{34}H_{33}BrN_3O$ $[M-Br]^+$ 578.1802, found 578.1806.

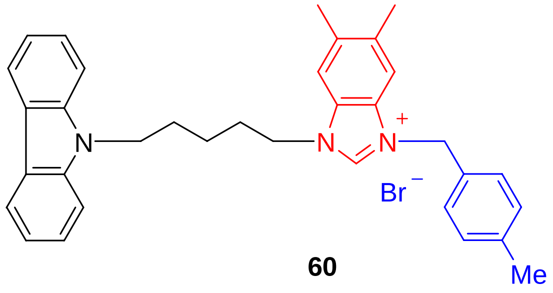

60

1-(5-(9*H*-carbazol-9-yl)pentyl)-5,6-dimethyl-3-(4-methylbenzyl)-1*H*-benzo[*d*]imidazol-3-ium bromide (**60**). Yield 90%. White powder, m.p. 123-125 °C. IR ν_{max} (cm^{-1}): 3117, 2937, 1598, 1558, 1474, 1453, 1337, 1224, 1125, 1013, 954, 846, 754, 718, 607.

^{1}H NMR (400 MHz, DMSO) δ: 9.70 (1H, s), 8.13 (2H, d, J = 7.7 Hz), 7.89 (2H, d, J = 8.4 Hz), 7.57 (2H, d, J = 8.2 Hz), 7.43-7.40 (4H, m), 7.21-7.17 (4H, m), 5.69 (2H, s), 4.41-4.37 (4H, m), 2.35-2.33 (6H, m), 2.27 (3H, s), 1.97-1.90 (2H, m), 1.87-1.80 (2H, m), 1.42-1.35 (2H, m). ^{13}C NMR (100 MHz, DMSO) δ: 141.31, 140.38, 138.51, 136.84, 136.80, 131.68, 130.14, 129.93, 129.69, 128.67, 126.07, 122.47, 120.69, 119.09, 113.71, 109.66, 49.90, 46.95, 42.52, 31.17, 28.84, 28.50. 23.92, 21.17, 20.48, 20.40. HRMS (ESI-TOF) m/z Calcd for $C_{34}H_{36}BrN_3$ $[M-Br]^+$ 486.2909, found 486.2902.

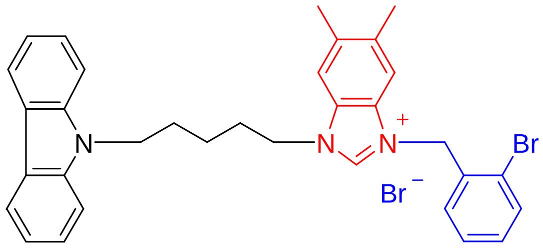

61

1-(5-(9*H*-carbazol-9-yl)pentyl)-3-(2-bromobenzyl)-5,6-dimethyl-1*H*-benzo[*d*]imidazol-3-ium bromide (**61**). Yield 90%. White powder, m.p. 126-127 °C. IR ν_{max} (cm^{-1}): 3125, 3051, 2938, 2876, 2353, 1599, 1561, 1453, 1335, 1218, 1155, 1029, 953, 845, 752, 611. ^{1}H NMR (400 MHz, DMSO) δ: 9.75 (1H, s), 8.12 (2H, d, J = 7.5 Hz), 7.86 (1H, s), 7.74 (1H, d, J = 7.5 Hz), 7.70 (1H, s), 7.55 (2H, t, J = 8.0 Hz), 7.42-7.36 (5H, m), 7.18 (2H, t, J = 7.2 Hz), 5.76 (2H, s), 4.43-4.41 (2H,m), 4.38-4.37 (2H, m), 2.37 (3H, s), 2.35 (3H, s), 1.93-1.90 (2H, m), 1.83-1.80 (2H, m), 1.38-1.35 (2H, m). ^{13}C NMR (100 MHz, MeOH) δ: 141.98, 140.37, 137.04, 133.73, 133.22, 131.41, 130.99, 130.04, 130.00, 128.92, 126.08, 123.47, 122.47, 120.71, 119.09, 113.89, 113.59, 109.66, 50.70, 47.01, 42.53, 28.96, 28.51, 23.86, 20.50, 20.42. HRMS (ESI-TOF) m/z Calcd for $C_{33}H_{33}BrN_3$ $[M-Br]^+$ 550.1852, found 550.1854.

3. X-ray crystal structure of compound 24 and 30

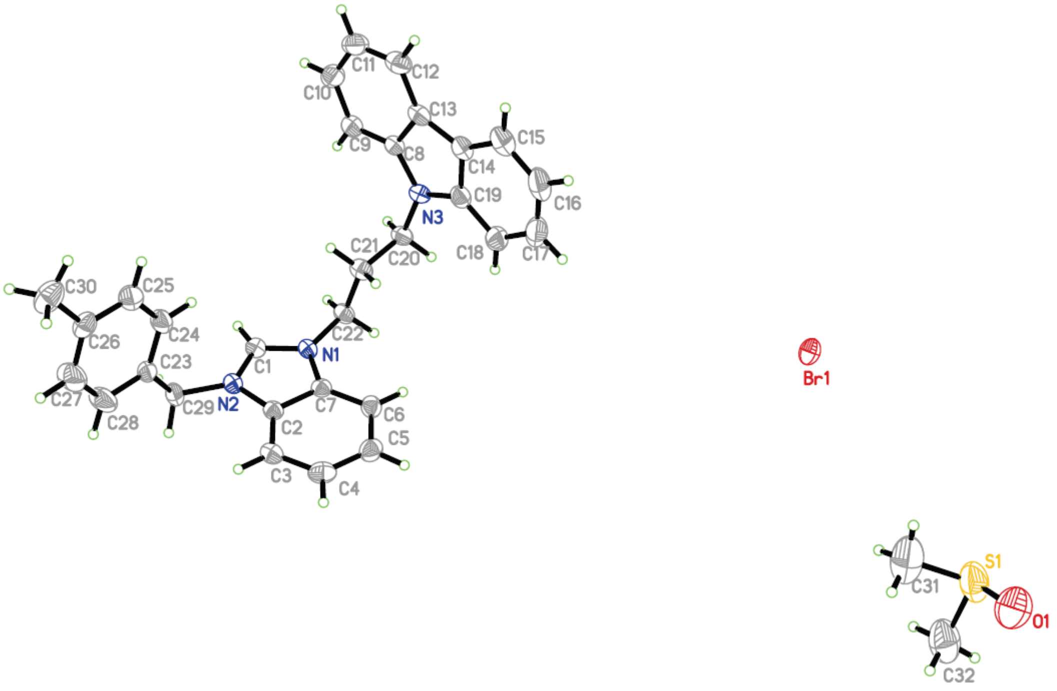

X-ray crystal structure of compound **24**.

data_**24**

_audit_creation_method SHELXL-97
_chemical_formula_sum 'C32 H34 Br N3 O S'
_chemical_formula_weight 588.59

loop_ _atom_type_symbol
 _atom_type_description
 _atom_type_scat_dispersion_real
 _atom_type_scat_dispersion_imag
 _atom_type_scat_source
 'C' 'C' 0.0033 0.0016
 'International Tables Vol C Tables 4.2.6.8 and 6.1.1.4'
 'H' 'H' 0.0000 0.0000
 'International Tables Vol C Tables 4.2.6.8 and 6.1.1.4'
 'N' 'N' 0.0061 0.0033
 'International Tables Vol C Tables 4.2.6.8 and 6.1.1.4'
 'Br' 'Br' -0.2901 2.4595
 'International Tables Vol C Tables 4.2.6.8 and 6.1.1.4'
 'O' 'O' 0.0106 0.0060

'International Tables Vol C Tables 4.2.6.8 and 6.1.1.4'
'S' 'S' 0.1246 0.1234
'International Tables Vol C Tables 4.2.6.8 and 6.1.1.4'

_symmetry_cell_setting Monoclinic
_symmetry_space_group_name_H-M P2(1)/c

loop_
_symmetry_equiv_pos_as_xyz
'x, y, z'
'-x, y+1/2, -z+1/2'
'-x, -y, -z'
'x, -y-1/2, z-1/2'

_cell_length_a 14.9093(17)
_cell_length_b 12.5673(14)
_cell_length_c 17.2574(19)
_cell_angle_alpha 90.00
_cell_angle_beta 112.6450(10)
_cell_angle_gamma 90.00
_cell_volume 2984.2(6)
_cell_formula_units_Z 4
_cell_measurement_temperature 293(2)
_cell_measurement_reflns_used 3014
_cell_measurement_theta_min 2.19
_cell_measurement_theta_max 20.84

_exptl_crystal_description BLOCK
_exptl_crystal_colour COLOURLESS
_exptl_crystal_size_max 0.30
_exptl_crystal_size_mid 0.24
_exptl_crystal_size_min 0.19
_exptl_crystal_density_meas 0
_exptl_crystal_density_diffrn 1.310
_exptl_crystal_density_method 'not measured'
_exptl_crystal_F_000 1224
_exptl_absorpt_coefficient_mu 1.476
_exptl_absorpt_correction_type multi-scan
_exptl_absorpt_correction_T_min 0.6535
_exptl_absorpt_correction_T_max 1.0000
_exptl_absorpt_process_details sadabs
_exptl_special_details
;
?

;

```
_diffrn_ambient_temperature            293(2)
_diffrn_radiation_wavelength           0.71073
_diffrn_radiation_type                 MoK\a
_diffrn_radiation_source               'fine-focus sealed tube'
_diffrn_radiation_monochromator        graphite
_diffrn_measurement_device_type        'CCD area detector'
_diffrn_measurement_method             'phi and omega scans'
_diffrn_detector_area_resol_mean       ?
_diffrn_standards_number               ?
_diffrn_standards_interval_count       ?
_diffrn_standards_interval_time        ?
_diffrn_standards_decay_%              ?
_diffrn_reflns_number                  20211
_diffrn_reflns_av_R_equivalents        0.0510
_diffrn_reflns_av_sigmaI/netI          0.0712
_diffrn_reflns_limit_h_min             -19
_diffrn_reflns_limit_h_max             19
_diffrn_reflns_limit_k_min             -16
_diffrn_reflns_limit_k_max             16
_diffrn_reflns_limit_l_min             -22
_diffrn_reflns_limit_l_max             22
_diffrn_reflns_theta_min               1.48
_diffrn_reflns_theta_max               28.18
_reflns_number_total                   6991
_reflns_number_gt                      3633
_reflns_threshold_expression           >2sigma(I)

_computing_data_collection             'Bruker apex II'
_computing_cell_refinement             'Bruker apex II'
_computing_data_reduction              'Bruker SAINT'
_computing_structure_solution          'SHELXS-97 (Sheldrick, 1990)'
_computing_structure_refinement        'SHELXL-97 (Sheldrick, 1997)'
_computing_molecular_graphics          'Bruker SHELXTL'
_computing_publication_material        'Bruker SHELXTL'

_refine_special_details
;
 Refinement of F^2^ against ALL reflections.  The weighted R-factor wR and
 goodness of fit S are based on F^2^, conventional R-factors R are based
 on F, with F set to zero for negative F^2^. The threshold expression of
 F^2^ > 2sigma(F^2^) is used only for calculating R-factors(gt) etc. and is
 not relevant to the choice of reflections for refinement.  R-factors based
```

on F^2^ are statistically about twice as large as those based on F, and R-
factors based on ALL data will be even larger.
;

```
_refine_ls_structure_factor_coef    Fsqd
_refine_ls_matrix_type              full
_refine_ls_weighting_scheme         calc
_refine_ls_weighting_details    'calc w=1/[\s^2^(Fo^2^)+(0.0599P)^2^+0.0000P] where
P=(Fo^2^+2Fc^2^)/3'
_atom_sites_solution_primary        direct
_atom_sites_solution_secondary      difmap
_atom_sites_solution_hydrogens      geom
_refine_ls_hydrogen_treatment       mixed
_refine_ls_extinction_method        SHELXL
_refine_ls_extinction_coef          0.0008(4)
_refine_ls_extinction_expression    'Fc^*^=kFc[1+0.001xFc^2^\l^3^/sin(2\q)]^-1/4^'
_refine_ls_number_reflns            6991
_refine_ls_number_parameters        347
_refine_ls_number_restraints        0
_refine_ls_R_factor_all             0.1177
_refine_ls_R_factor_gt              0.0483
_refine_ls_wR_factor_ref            0.1332
_refine_ls_wR_factor_gt             0.1094
_refine_ls_goodness_of_fit_ref      0.989
_refine_ls_restrained_S_all         0.989
_refine_ls_shift/su_max             0.000
_refine_ls_shift/su_mean            0.000

loop_
  _atom_site_label
  _atom_site_type_symbol
  _atom_site_fract_x
  _atom_site_fract_y
  _atom_site_fract_z
  _atom_site_U_iso_or_equiv
  _atom_site_adp_type
  _atom_site_occupancy
  _atom_site_symmetry_multiplicity
  _atom_site_calc_flag
  _atom_site_refinement_flags
  _atom_site_disorder_assembly
  _atom_site_disorder_group
N1 N 0.36400(16) 0.36220(17) 0.97016(15) 0.0397(6) Uani 1 1 d . . .
N2 N 0.36906(17) 0.46462(18) 1.07396(15) 0.0426(6) Uani 1 1 d . . .
```

N3 N 0.13467(18) 0.16308(19) 0.73394(16) 0.0496(6) Uani 1 1 d . . .
O1 O 0.3157(3) 1.0448(3) -0.0066(2) 0.1625(16) Uani 1 1 d . . .
S1 S 0.35490(14) 0.94847(11) 0.04261(9) 0.1324(6) Uani 1 1 d . . .
Br1 Br 0.45209(3) 0.22244(3) 0.24280(2) 0.06402(17) Uani 1 1 d . . .
C1 C 0.3629(2) 0.3648(2) 1.04649(19) 0.0440(7) Uani 1 1 d . . .
H1 H 0.3585 0.3052 1.0767 0.053 Uiso 1 1 calc R . .
C2 C 0.37482(19) 0.5312(2) 1.01198(18) 0.0401(7) Uani 1 1 d . . .
C3 C 0.3841(2) 0.6408(2) 1.0093(2) 0.0500(8) Uani 1 1 d . . .
H3 H 0.3853 0.6845 1.0532 0.060 Uiso 1 1 calc R . .
C4 C 0.3913(2) 0.6809(3) 0.9380(2) 0.0623(9) Uani 1 1 d . . .
H4 H 0.3965 0.7541 0.9332 0.075 Uiso 1 1 calc R . .
C5 C 0.3910(2) 0.6162(3) 0.8726(2) 0.0579(9) Uani 1 1 d . . .
H5 H 0.3981 0.6472 0.8263 0.069 Uiso 1 1 calc R . .
C6 C 0.3806(2) 0.5069(2) 0.87430(19) 0.0490(8) Uani 1 1 d . . .
H6 H 0.3794 0.4633 0.8303 0.059 Uiso 1 1 calc R . .
C7 C 0.37196(19) 0.4665(2) 0.94600(18) 0.0403(7) Uani 1 1 d . . .
C8 C 0.0580(2) 0.1149(2) 0.74663(19) 0.0466(8) Uani 1 1 d . . .
C9 C 0.0595(2) 0.0521(2) 0.8130(2) 0.0553(8) Uani 1 1 d . . .
H9 H 0.1177 0.0357 0.8571 0.066 Uiso 1 1 calc R . .
C10 C -0.0285(3) 0.0143(3) 0.8119(2) 0.0658(10) Uani 1 1 d . . .
H10 H -0.0292 -0.0280 0.8559 0.079 Uiso 1 1 calc R . .
C11 C -0.1151(3) 0.0386(3) 0.7462(3) 0.0723(11) Uani 1 1 d . . .
H11 H -0.1732 0.0125 0.7469 0.087 Uiso 1 1 calc R . .
C12 C -0.1167(3) 0.1002(3) 0.6806(2) 0.0650(10) Uani 1 1 d . . .
H12 H -0.1754 0.1156 0.6369 0.078 Uiso 1 1 calc R . .
C13 C -0.0297(2) 0.1403(2) 0.6790(2) 0.0522(8) Uani 1 1 d . . .
C14 C -0.0042(3) 0.2082(2) 0.6237(2) 0.0549(8) Uani 1 1 d . . .
C15 C -0.0570(3) 0.2583(3) 0.5481(2) 0.0758(11) Uani 1 1 d . . .
H15 H -0.1242 0.2509 0.5229 0.091 Uiso 1 1 calc R . .
C16 C -0.0071(4) 0.3194(3) 0.5112(2) 0.0859(13) Uani 1 1 d . . .
H16 H -0.0418 0.3541 0.4609 0.103 Uiso 1 1 calc R . .
C17 C 0.0921(4) 0.3304(3) 0.5468(3) 0.0831(13) Uani 1 1 d . . .
H17 H 0.1230 0.3714 0.5196 0.100 Uiso 1 1 calc R . .
C18 C 0.1472(3) 0.2821(3) 0.6220(2) 0.0676(10) Uani 1 1 d . . .
H18 H 0.2143 0.2901 0.6463 0.081 Uiso 1 1 calc R . .
C19 C 0.0975(3) 0.2206(2) 0.6598(2) 0.0534(8) Uani 1 1 d . . .
C20 C 0.2336(2) 0.1662(2) 0.79423(19) 0.0506(8) Uani 1 1 d . . .
H20A H 0.2773 0.1745 0.7651 0.061 Uiso 1 1 calc R . .
H20B H 0.2491 0.0995 0.8249 0.061 Uiso 1 1 calc R . .
C21 C 0.2484(2) 0.2576(2) 0.8555(2) 0.0510(8) Uani 1 1 d . . .
H21A H 0.2299 0.3237 0.8243 0.061 Uiso 1 1 calc R . .
H21B H 0.2060 0.2476 0.8857 0.061 Uiso 1 1 calc R . .
C22 C 0.3518(2) 0.2666(2) 0.91770(19) 0.0461(7) Uani 1 1 d . . .
H22A H 0.3687 0.2038 0.9532 0.055 Uiso 1 1 calc R . .

H22B H 0.3952 0.2705 0.8879 0.055 Uiso 1 1 calc R . .
C23 C 0.2767(2) 0.5441(2) 1.15218(19) 0.0447(7) Uani 1 1 d . . .
C24 C 0.1889(2) 0.5038(3) 1.1004(2) 0.0591(9) Uani 1 1 d . . .
H24 H 0.1864 0.4494 1.0631 0.071 Uiso 1 1 calc R . .
C25 C 0.1033(3) 0.5436(3) 1.1031(2) 0.0676(10) Uani 1 1 d . . .
H25 H 0.0444 0.5144 1.0678 0.081 Uiso 1 1 calc R . .
C26 C 0.1028(3) 0.6246(3) 1.1562(2) 0.0580(9) Uani 1 1 d . . .
C27 C 0.1909(3) 0.6643(3) 1.2062(3) 0.0896(13) Uani 1 1 d . . .
H27 H 0.1934 0.7195 1.2429 0.108 Uiso 1 1 calc R . .
C28 C 0.2767(3) 0.6256(3) 1.2046(3) 0.0815(12) Uani 1 1 d . . .
H28 H 0.3354 0.6554 1.2397 0.098 Uiso 1 1 calc R . .
C29 C 0.3718(2) 0.4974(2) 1.15652(18) 0.0501(8) Uani 1 1 d . . .
H29A H 0.4229 0.5498 1.1804 0.060 Uiso 1 1 calc R . .
H29B H 0.3877 0.4362 1.1936 0.060 Uiso 1 1 calc R . .
C30 C 0.0093(3) 0.6648(3) 1.1586(3) 0.0843(13) Uani 1 1 d . . .
H30A H 0.0070 0.6487 1.2122 0.127 Uiso 1 1 calc R . .
H30B H -0.0443 0.6311 1.1150 0.127 Uiso 1 1 calc R . .
H30C H 0.0053 0.7404 1.1501 0.127 Uiso 1 1 calc R . .
C31 C 0.3077(5) 0.9462(5) 0.1206(4) 0.160(2) Uani 1 1 d . . .
H31A H 0.3178 1.0142 0.1481 0.240 Uiso 1 1 calc R . .
H31B H 0.3400 0.8920 0.1610 0.240 Uiso 1 1 calc R . .
H31C H 0.2394 0.9311 0.0958 0.240 Uiso 1 1 calc R . .
C32 C 0.4776(4) 0.9766(4) 0.1104(3) 0.131(2) Uani 1 1 d . . .
H32A H 0.5178 0.9778 0.0783 0.196 Uiso 1 1 calc R . .
H32B H 0.5005 0.9224 0.1528 0.196 Uiso 1 1 calc R . .
H32C H 0.4805 1.0445 0.1367 0.196 Uiso 1 1 calc R . .

loop_
 _atom_site_aniso_label
 _atom_site_aniso_U_11
 _atom_site_aniso_U_22
 _atom_site_aniso_U_33
 _atom_site_aniso_U_23
 _atom_site_aniso_U_13
 _atom_site_aniso_U_12
N1 0.0395(14) 0.0396(14) 0.0378(15) -0.0038(11) 0.0124(12) 0.0019(11)
N2 0.0452(15) 0.0420(14) 0.0408(15) -0.0039(12) 0.0169(12) -0.0001(12)
N3 0.0456(16) 0.0525(15) 0.0461(16) -0.0080(13) 0.0123(14) -0.0112(13)
O1 0.194(4) 0.132(3) 0.117(3) 0.058(2) 0.011(3) -0.009(3)
S1 0.2214(19) 0.0868(9) 0.0747(9) 0.0063(7) 0.0413(11) 0.0049(11)
Br1 0.0735(3) 0.0609(2) 0.0643(3) 0.01566(18) 0.0338(2) 0.01743(19)
C1 0.0425(18) 0.0430(17) 0.0447(19) 0.0003(14) 0.0145(15) 0.0018(14)
C2 0.0322(16) 0.0437(17) 0.0412(18) -0.0030(14) 0.0106(14) -0.0001(13)
C3 0.050(2) 0.0457(18) 0.052(2) -0.0069(15) 0.0160(17) -0.0020(15)

C4 0.060(2) 0.0443(18) 0.077(3) 0.0051(19) 0.021(2) -0.0041(17)
C5 0.054(2) 0.064(2) 0.053(2) 0.0112(18) 0.0177(18) -0.0021(18)
C6 0.0444(19) 0.058(2) 0.0433(19) -0.0002(16) 0.0151(16) 0.0021(15)
C7 0.0310(16) 0.0443(17) 0.0434(18) -0.0016(14) 0.0117(14) -0.0003(13)
C8 0.050(2) 0.0446(17) 0.0435(19) -0.0090(15) 0.0164(17) -0.0067(15)
C9 0.060(2) 0.0526(19) 0.053(2) -0.0077(17) 0.0211(18) -0.0051(17)
C10 0.077(3) 0.055(2) 0.074(3) -0.0109(19) 0.039(2) -0.011(2)
C11 0.059(3) 0.076(3) 0.087(3) -0.021(2) 0.034(2) -0.017(2)
C12 0.049(2) 0.069(2) 0.070(3) -0.016(2) 0.014(2) -0.0016(19)
C13 0.048(2) 0.0518(19) 0.049(2) -0.0126(16) 0.0108(17) -0.0031(16)
C14 0.064(2) 0.0506(19) 0.043(2) -0.0039(16) 0.0130(18) 0.0012(17)
C15 0.088(3) 0.074(2) 0.052(2) -0.005(2) 0.012(2) 0.003(2)
C16 0.126(4) 0.077(3) 0.048(2) 0.002(2) 0.027(3) 0.006(3)
C17 0.127(4) 0.072(3) 0.065(3) -0.002(2) 0.053(3) -0.008(3)
C18 0.087(3) 0.061(2) 0.062(3) -0.011(2) 0.037(2) -0.011(2)
C19 0.066(2) 0.0466(18) 0.045(2) -0.0068(16) 0.0190(18) -0.0041(17)
C20 0.0441(19) 0.0510(18) 0.054(2) -0.0132(16) 0.0162(17) -0.0054(15)
C21 0.0448(19) 0.0472(17) 0.057(2) -0.0094(15) 0.0153(17) 0.0043(15)
C22 0.0436(18) 0.0428(16) 0.0478(19) -0.0085(14) 0.0132(16) 0.0034(14)
C23 0.054(2) 0.0414(16) 0.0415(18) -0.0031(14) 0.0214(16) -0.0034(15)
C24 0.058(2) 0.063(2) 0.060(2) -0.0191(18) 0.0267(19) -0.0026(18)
C25 0.058(2) 0.082(3) 0.064(2) -0.011(2) 0.025(2) -0.007(2)
C26 0.069(3) 0.0506(19) 0.066(2) 0.0108(18) 0.039(2) 0.0057(18)
C27 0.087(3) 0.076(3) 0.116(4) -0.044(3) 0.050(3) -0.004(2)
C28 0.066(3) 0.080(3) 0.103(3) -0.046(2) 0.037(2) -0.011(2)
C29 0.060(2) 0.0529(18) 0.0369(18) -0.0066(15) 0.0186(16) -0.0003(16)
C30 0.087(3) 0.075(3) 0.114(4) 0.014(2) 0.065(3) 0.019(2)
C31 0.207(7) 0.175(6) 0.117(5) 0.020(4) 0.083(5) 0.019(5)
C32 0.167(6) 0.122(4) 0.091(4) 0.014(3) 0.037(4) 0.044(4)
_geom_special_details
;
 All esds (except the esd in the dihedral angle between two l.s. planes)
 are estimated using the full covariance matrix. The cell esds are taken
 into account individually in the estimation of esds in distances, angles
 and torsion angles; correlations between esds in cell parameters are only
 used when they are defined by crystal symmetry. An approximate (isotropic)
 treatment of cell esds is used for estimating esds involving l.s. planes.
;

loop_
 _geom_bond_atom_site_label_1
 _geom_bond_atom_site_label_2
 _geom_bond_distance
 _geom_bond_site_symmetry_2

_geom_bond_publ_flag
N1 C1 1.324(3) . ?
N1 C7 1.394(3) . ?
N1 C22 1.473(3) . ?
N2 C1 1.332(3) . ?
N2 C2 1.386(3) . ?
N2 C29 1.468(3) . ?
N3 C8 1.384(4) . ?
N3 C19 1.387(4) . ?
N3 C20 1.441(4) . ?
O1 S1 1.465(4) . ?
S1 C31 1.743(5) . ?
S1 C32 1.787(6) . ?
C1 H1 0.9300 . ?
C2 C7 1.386(4) . ?
C2 C3 1.387(4) . ?
C3 C4 1.372(4) . ?
C3 H3 0.9300 . ?
C4 C5 1.389(4) . ?
C4 H4 0.9300 . ?
C5 C6 1.384(4) . ?
C5 H5 0.9300 . ?
C6 C7 1.388(4) . ?
C6 H6 0.9300 . ?
C8 C9 1.385(4) . ?
C8 C13 1.413(4) . ?
C9 C10 1.387(4) . ?
C9 H9 0.9300 . ?
C10 C11 1.385(5) . ?
C10 H10 0.9300 . ?
C11 C12 1.365(5) . ?
C11 H11 0.9300 . ?
C12 C13 1.401(4) . ?
C12 H12 0.9300 . ?
C13 C14 1.438(4) . ?
C14 C15 1.388(5) . ?
C14 C19 1.408(5) . ?
C15 C16 1.383(6) . ?
C15 H15 0.9300 . ?
C16 C17 1.373(6) . ?
C16 H16 0.9300 . ?
C17 C18 1.380(5) . ?
C17 H17 0.9300 . ?
C18 C19 1.393(5) . ?

C18 H18 0.9300 . ?
C20 C21 1.519(4) . ?
C20 H20A 0.9700 . ?
C20 H20B 0.9700 . ?
C21 C22 1.506(4) . ?
C21 H21A 0.9700 . ?
C21 H21B 0.9700 . ?
C22 H22A 0.9700 . ?
C22 H22B 0.9700 . ?
C23 C24 1.366(4) . ?
C23 C28 1.367(4) . ?
C23 C29 1.510(4) . ?
C24 C25 1.387(4) . ?
C24 H24 0.9300 . ?
C25 C26 1.372(4) . ?
C25 H25 0.9300 . ?
C26 C27 1.359(5) . ?
C26 C30 1.497(5) . ?
C27 C28 1.378(5) . ?
C27 H27 0.9300 . ?
C28 H28 0.9300 . ?
C29 H29A 0.9700 . ?
C29 H29B 0.9700 . ?
C30 H30A 0.9600 . ?
C30 H30B 0.9600 . ?
C30 H30C 0.9600 . ?
C31 H31A 0.9600 . ?
C31 H31B 0.9600 . ?
C31 H31C 0.9600 . ?
C32 H32A 0.9600 . ?
C32 H32B 0.9600 . ?
C32 H32C 0.9600 . ?

loop_
 _geom_angle_atom_site_label_1
 _geom_angle_atom_site_label_2
 _geom_angle_atom_site_label_3
 _geom_angle _geom_angle_site_symmetry_1
 _geom_angle_site_symmetry_3
 _geom_angle_publ_flag
C1 N1 C7 108.0(2) . . ?
C1 N1 C22 125.9(2) . . ?
C7 N1 C22 126.0(2) . . ?
C1 N2 C2 108.0(2) . . ?

C1 N2 C29 125.6(2) . . ?
C2 N2 C29 126.3(2) . . ?
C8 N3 C19 108.2(3) . . ?
C8 N3 C20 125.2(3) . . ?
C19 N3 C20 125.9(3) . . ?
O1 S1 C31 105.8(3) . . ?
O1 S1 C32 107.2(3) . . ?
C31 S1 C32 96.5(3) . . ?
N1 C1 N2 110.6(3) . . ?
N1 C1 H1 124.7 . . ?
N2 C1 H1 124.7 . . ?
C7 C2 N2 106.8(2) . . ?
C7 C2 C3 121.8(3) . . ?
N2 C2 C3 131.4(3) . . ?
C4 C3 C2 116.0(3) . . ?
C4 C3 H3 122.0 . . ?
C2 C3 H3 122.0 . . ?
C3 C4 C5 122.5(3) . . ?
C3 C4 H4 118.8 . . ?
C5 C4 H4 118.8 . . ?
C6 C5 C4 121.9(3) . . ?
C6 C5 H5 119.1 . . ?
C4 C5 H5 119.1 . . ?
C5 C6 C7 115.6(3) . . ?
C5 C6 H6 122.2 . . ?
C7 C6 H6 122.2 . . ?
C2 C7 C6 122.3(3) . . ?
C2 C7 N1 106.5(2) . . ?
C6 C7 N1 131.1(3) . . ?
N3 C8 C9 129.0(3) . . ?
N3 C8 C13 109.2(3) . . ?
C9 C8 C13 121.8(3) . . ?
C8 C9 C10 118.0(3) . . ?
C8 C9 H9 121.0 . . ?
C10 C9 H9 121.0 . . ?
C11 C10 C9 121.0(3) . . ?
C11 C10 H10 119.5 . . ?
C9 C10 H10 119.5 . . ?
C12 C11 C10 121.1(3) . . ?
C12 C11 H11 119.5 . . ?
C10 C11 H11 119.5 . . ?
C11 C12 C13 120.0(4) . . ?
C11 C12 H12 120.0 . . ?
C13 C12 H12 120.0 . . ?

C12 C13 C8 118.2(3) . . ?
C12 C13 C14 135.2(3) . . ?
C8 C13 C14 106.6(3) . . ?
C15 C14 C19 119.3(3) . . ?
C15 C14 C13 134.0(4) . . ?
C19 C14 C13 106.7(3) . . ?
C16 C15 C14 118.3(4) . . ?
C16 C15 H15 120.9 . . ?
C14 C15 H15 120.9 . . ?
C17 C16 C15 121.8(4) . . ?
C17 C16 H16 119.1 . . ?
C15 C16 H16 119.1 . . ?
C16 C17 C18 121.7(4) . . ?
C16 C17 H17 119.2 . . ?
C18 C17 H17 119.2 . . ?
C17 C18 C19 116.9(4) . . ?
C17 C18 H18 121.5 . . ?
C19 C18 H18 121.5 . . ?
N3 C19 C18 128.7(3) . . ?
N3 C19 C14 109.3(3) . . ?
C18 C19 C14 122.0(3) . . ?
N3 C20 C21 110.9(2) . . ?
N3 C20 H20A 109.5 . . ?
C21 C20 H20A 109.5 . . ?
N3 C20 H20B 109.5 . . ?
C21 C20 H20B 109.5 . . ?
H20A C20 H20B 108.0 . . ?
C22 C21 C20 112.7(2) . . ?
C22 C21 H21A 109.0 . . ?
C20 C21 H21A 109.0 . . ?
C22 C21 H21B 109.0 . . ?
C20 C21 H21B 109.0 . . ?
H21A C21 H21B 107.8 . . ?
N1 C22 C21 110.4(2) . . ?
N1 C22 H22A 109.6 . . ?
C21 C22 H22A 109.6 . . ?
N1 C22 H22B 109.6 . . ?
C21 C22 H22B 109.6 . . ?
H22A C22 H22B 108.1 . . ?
C24 C23 C28 117.8(3) . . ?
C24 C23 C29 122.3(3) . . ?
C28 C23 C29 119.8(3) . . ?
C23 C24 C25 120.4(3) . . ?
C23 C24 H24 119.8 . . ?

C25 C24 H24 119.8 . . ?
C26 C25 C24 122.1(3) . . ?
C26 C25 H25 118.9 . . ?
C24 C25 H25 118.9 . . ?
C27 C26 C25 116.3(3) . . ?
C27 C26 C30 122.8(3) . . ?
C25 C26 C30 120.9(4) . . ?
C26 C27 C28 122.4(3) . . ?
C26 C27 H27 118.8 . . ?
C28 C27 H27 118.8 . . ?
C23 C28 C27 120.9(4) . . ?
C23 C28 H28 119.5 . . ?
C27 C28 H28 119.5 . . ?
N2 C29 C23 112.7(2) . . ?
N2 C29 H29A 109.0 . . ?
C23 C29 H29A 109.0 . . ?
N2 C29 H29B 109.0 . . ?
C23 C29 H29B 109.0 . . ?
H29A C29 H29B 107.8 . . ?
C26 C30 H30A 109.5 . . ?
C26 C30 H30B 109.5 . . ?
H30A C30 H30B 109.5 . . ?
C26 C30 H30C 109.5 . . ?
H30A C30 H30C 109.5 . . ?
H30B C30 H30C 109.5 . . ?
S1 C31 H31A 109.5 . . ?
S1 C31 H31B 109.5 . . ?
H31A C31 H31B 109.5 . . ?
S1 C31 H31C 109.5 . . ?
H31A C31 H31C 109.5 . . ?
H31B C31 H31C 109.5 . . ?
S1 C32 H32A 109.5 . . ?
S1 C32 H32B 109.5 . . ?
H32A C32 H32B 109.5 . . ?
S1 C32 H32C 109.5 . . ?
H32A C32 H32C 109.5 . . ?
H32B C32 H32C 109.5 . . ?

loop_
_geom_torsion_atom_site_label_1
_geom_torsion_atom_site_label_2
_geom_torsion_atom_site_label_3
_geom_torsion_atom_site_label_4
_geom_torsion

_geom_torsion_site_symmetry_1
_geom_torsion_site_symmetry_2
_geom_torsion_site_symmetry_3
_geom_torsion_site_symmetry_4
_geom_torsion_publ_flag
C7 N1 C1 N2 0.4(3) . . . . ?
C22 N1 C1 N2 -175.6(2) . . . . ?
C2 N2 C1 N1 -0.2(3) . . . . ?
C29 N2 C1 N1 -178.7(2) . . . . ?
C1 N2 C2 C7 -0.1(3) . . . . ?
C29 N2 C2 C7 178.4(3) . . . . ?
C1 N2 C2 C3 -178.6(3) . . . . ?
C29 N2 C2 C3 -0.1(5) . . . . ?
C7 C2 C3 C4 -0.7(4) . . . . ?
N2 C2 C3 C4 177.7(3) . . . . ?
C2 C3 C4 C5 -1.0(5) . . . . ?
C3 C4 C5 C6 1.9(5) . . . . ?
C4 C5 C6 C7 -1.0(5) . . . . ?
N2 C2 C7 C6 -177.1(3) . . . . ?
C3 C2 C7 C6 1.6(4) . . . . ?
N2 C2 C7 N1 0.3(3) . . . . ?
C3 C2 C7 N1 179.0(3) . . . . ?
C5 C6 C7 C2 -0.7(4) . . . . ?
C5 C6 C7 N1 -177.4(3) . . . . ?
C1 N1 C7 C2 -0.4(3) . . . . ?
C22 N1 C7 C2 175.6(2) . . . . ?
C1 N1 C7 C6 176.7(3) . . . . ?
C22 N1 C7 C6 -7.4(5) . . . . ?
C19 N3 C8 C9 -178.2(3) . . . . ?
C20 N3 C8 C9 -7.2(5) . . . . ?
C19 N3 C8 C13 1.0(3) . . . . ?
C20 N3 C8 C13 172.0(3) . . . . ?
N3 C8 C9 C10 178.9(3) . . . . ?
C13 C8 C9 C10 -0.2(4) . . . . ?
C8 C9 C10 C11 0.1(5) . . . . ?
C9 C10 C11 C12 0.2(5) . . . . ?
C10 C11 C12 C13 -0.2(5) . . . . ?
C11 C12 C13 C8 0.0(5) . . . . ?
C11 C12 C13 C14 -177.6(3) . . . . ?
N3 C8 C13 C12 -179.1(3) . . . . ?
C9 C8 C13 C12 0.2(4) . . . . ?
N3 C8 C13 C14 -0.8(3) . . . . ?
C9 C8 C13 C14 178.5(3) . . . . ?
C12 C13 C14 C15 -2.2(6) . . . . ?

C8 C13 C14 C15 179.9(4) . . . . ?
C12 C13 C14 C19 178.1(3) . . . . ?
C8 C13 C14 C19 0.2(3) . . . . ?
C19 C14 C15 C16 -0.3(5) . . . . ?
C13 C14 C15 C16 -180.0(3) . . . . ?
C14 C15 C16 C17 0.7(6) . . . . ?
C15 C16 C17 C18 -0.9(6) . . . . ?
C16 C17 C18 C19 0.6(6) . . . . ?
C8 N3 C19 C18 179.7(3) . . . . ?
C20 N3 C19 C18 8.8(5) . . . . ?
C8 N3 C19 C14 -0.9(3) . . . . ?
C20 N3 C19 C14 -171.8(3) . . . . ?
C17 C18 C19 N3 179.1(3) . . . . ?
C17 C18 C19 C14 -0.2(5) . . . . ?
C15 C14 C19 N3 -179.4(3) . . . . ?
C13 C14 C19 N3 0.4(3) . . . . ?
C15 C14 C19 C18 0.1(5) . . . . ?
C13 C14 C19 C18 179.8(3) . . . . ?
C8 N3 C20 C21 -84.2(4) . . . . ?
C19 N3 C20 C21 85.3(4) . . . . ?
N3 C20 C21 C22 -177.8(3) . . . . ?
C1 N1 C22 C21 98.0(3) . . . . ?
C7 N1 C22 C21 -77.3(4) . . . . ?
C20 C21 C22 N1 174.6(3) . . . . ?
C28 C23 C24 C25 -1.4(5) . . . . ?
C29 C23 C24 C25 174.9(3) . . . . ?
C23 C24 C25 C26 0.8(5) . . . . ?
C24 C25 C26 C27 0.1(5) . . . . ?
C24 C25 C26 C30 -179.1(3) . . . . ?
C25 C26 C27 C28 -0.3(6) . . . . ?
C30 C26 C27 C28 178.9(4) . . . . ?
C24 C23 C28 C27 1.3(6) . . . . ?
C29 C23 C28 C27 -175.1(4) . . . . ?
C26 C27 C28 C23 -0.4(7) . . . . ?
C1 N2 C29 C23 -104.6(3) . . . . ?
C2 N2 C29 C23 77.1(3) . . . . ?
C24 C23 C29 N2 39.0(4) . . . . ?
C28 C23 C29 N2 -144.7(3) . . . . ?

_diffrn_measured_fraction_theta_max 0.954
_diffrn_reflns_theta_full 28.18
_diffrn_measured_fraction_theta_full 0.954
_refine_diff_density_max 0.564
_refine_diff_density_min -0.505

_refine_diff_density_rms 0.052

Compound **30**

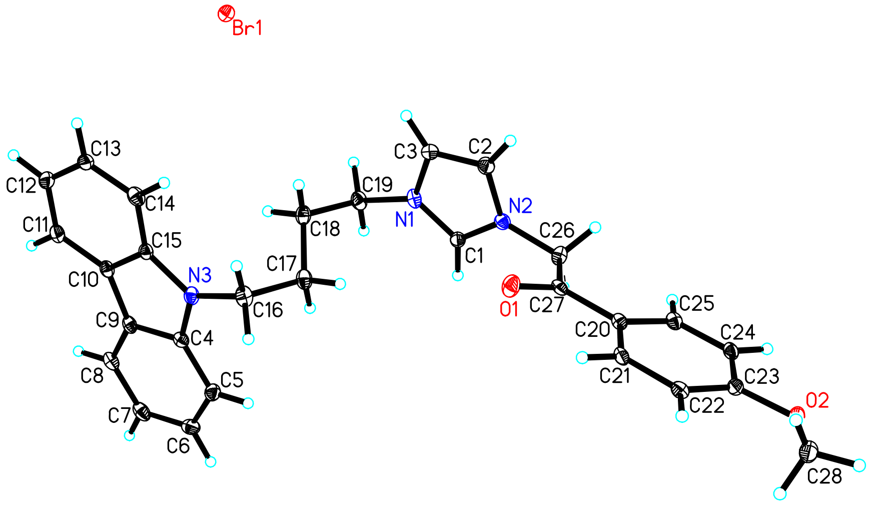

X-ray crystal structure of compound **30**.

data_**30**

_audit_creation_method	SHELXL-97
_chemical_formula_moiety	'C28 H28 N3 O2, Br, 2(H2 O)'
_chemical_formula_sum	'C28 H32 Br N3 O4'
_chemical_formula_weight	554.48

loop_
 _atom_type_symbol
 _atom_type_description
 _atom_type_scat_dispersion_real
 _atom_type_scat_dispersion_imag
 _atom_type_scat_source
 'C' 'C' 0.0033 0.0016
 'International Tables Vol C Tables 4.2.6.8 and 6.1.1.4'
 'H' 'H' 0.0000 0.0000
 'International Tables Vol C Tables 4.2.6.8 and 6.1.1.4'

```
 'N'  'N'   0.0061   0.0033
 'International Tables Vol C Tables 4.2.6.8 and 6.1.1.4'
 'O'  'O'   0.0106   0.0060
 'International Tables Vol C Tables 4.2.6.8 and 6.1.1.4'
 'Br'  'Br'  -0.2901   2.4595
 'International Tables Vol C Tables 4.2.6.8 and 6.1.1.4'

_symmetry_cell_setting            'Monoclinic'
_symmetry_space_group_name_H-M    'P 21/c'
_symmetry_space_group_name_Hall   '-P 2ybc'

loop_
 _symmetry_equiv_pos_as_xyz
 'x, y, z'
 '-x, y+1/2, -z+1/2'
 '-x, -y, -z'
 'x, -y-1/2, z-1/2'

_cell_length_a                    17.043(3)
_cell_length_b                    12.876(3)
_cell_length_c                    12.422(2)
_cell_angle_alpha                 90.00
_cell_angle_beta                  109.493(3)
_cell_angle_gamma                 90.00
_cell_volume                      2569.8(9)
_cell_formula_units_Z             4
_cell_measurement_temperature     100(2)
_cell_measurement_reflns_used     3279
_cell_measurement_theta_min       2.54
_cell_measurement_theta_max       24.27

_exptl_crystal_description        'piece'
_exptl_crystal_colour             'colourless'
_exptl_crystal_size_max           0.50
_exptl_crystal_size_mid           0.17
_exptl_crystal_size_min           0.01
_exptl_crystal_density_meas       ?
_exptl_crystal_density_diffrn     1.433
_exptl_crystal_density_method     'not measured'
_exptl_crystal_F_000              1152
_exptl_absorpt_coefficient_mu     1.639
_exptl_absorpt_correction_type    'multi-scan'
_exptl_absorpt_correction_T_min   0.4945
_exptl_absorpt_correction_T_max   0.9838
```

_exptl_absorpt_process_details 'sadabs'

_exptl_special_details
;
?
;

_diffrn_ambient_temperature 100(2)
_diffrn_radiation_wavelength 0.71073
_diffrn_radiation_type MoK\a
_diffrn_radiation_source 'fine-focus sealed tube'
_diffrn_radiation_monochromator graphite
_diffrn_measurement_device_type 'Bruker APEX-II CCD'
_diffrn_measurement_method '\f and \w scans'
_diffrn_detector_area_resol_mean ?
_diffrn_reflns_number 24253
_diffrn_reflns_av_R_equivalents 0.0900
_diffrn_reflns_av_sigmaI/netI 0.0895
_diffrn_reflns_limit_h_min -22
_diffrn_reflns_limit_h_max 22
_diffrn_reflns_limit_k_min -17
_diffrn_reflns_limit_k_max 17
_diffrn_reflns_limit_l_min -16
_diffrn_reflns_limit_l_max 16
_diffrn_reflns_theta_min 1.27
_diffrn_reflns_theta_max 28.25
_reflns_number_total 6313
_reflns_number_gt 3853
_reflns_threshold_expression >2sigma(I)

_computing_data_collection 'Bruker APEX2'
_computing_cell_refinement 'Bruker SAINT'
_computing_data_reduction 'Bruker SAINT'
_computing_structure_solution 'SHELXS-97 (Sheldrick, 2008)'
_computing_structure_refinement 'SHELXL-97 (Sheldrick, 2008)'
_computing_molecular_graphics 'Bruker SHELXTL'
_computing_publication_material 'Bruker SHELXTL'

_refine_special_details
;
Refinement of F^2^ against ALL reflections. The weighted R-factor wR and
goodness of fit S are based on F^2^, conventional R-factors R are based
on F, with F set to zero for negative F^2^. The threshold expression of
F^2^ > 2sigma(F^2^) is used only for calculating R-factors(gt) etc. and is

not relevant to the choice of reflections for refinement. R-factors based
on F^2^ are statistically about twice as large as those based on F, and R-
factors based on ALL data will be even larger.
;

_refine_ls_structure_factor_coef Fsqd
_refine_ls_matrix_type full
_refine_ls_weighting_scheme calc
_refine_ls_weighting_details
'calc w=1/[\s^2^(Fo^2^)+(0.1023P)^2^+1.5741P] where P=(Fo^2^+2Fc^2^)/3'
_atom_sites_solution_primary direct
_atom_sites_solution_secondary difmap
_atom_sites_solution_hydrogens geom
_refine_ls_hydrogen_treatment mixed
_refine_ls_extinction_method none
_refine_ls_extinction_coef ?
_refine_ls_number_reflns 6313
_refine_ls_number_parameters 338
_refine_ls_number_restraints 6
_refine_ls_R_factor_all 0.1186
_refine_ls_R_factor_gt 0.0591
_refine_ls_wR_factor_ref 0.1932
_refine_ls_wR_factor_gt 0.1536
_refine_ls_goodness_of_fit_ref 1.051
_refine_ls_restrained_S_all 1.051
_refine_ls_shift/su_max 0.000
_refine_ls_shift/su_mean 0.000

loop_
_atom_site_label
_atom_site_type_symbol
_atom_site_fract_x
_atom_site_fract_y
_atom_site_fract_z
_atom_site_U_iso_or_equiv
_atom_site_adp_type
_atom_site_occupancy
_atom_site_symmetry_multiplicity
_atom_site_calc_flag
_atom_site_refinement_flags
_atom_site_disorder_assembly
_atom_site_disorder_group
Br1 Br 0.33835(3) 0.49417(3) 0.83576(4) 0.02564(16) Uani 1 1 d . . .
O2 O 0.9030(2) 0.0569(3) -0.0129(3) 0.0279(8) Uani 1 1 d . . .

O1S O 0.5637(2) 0.5786(3) 0.3388(3) 0.0351(9) Uani 1 1 d D . .
H1SA H 0.584(4) 0.580(4) 0.288(4) 0.053 Uiso 1 1 d D . .
H1SB H 0.553(4) 0.5169(15) 0.343(5) 0.053 Uiso 1 1 d D . .
O1 O 0.6040(2) 0.1162(3) 0.1782(3) 0.0297(8) Uani 1 1 d . . .
C22 C 0.7656(3) 0.0346(4) 0.0008(4) 0.0221(9) Uani 1 1 d . . .
H22 H 0.7496 -0.0142 -0.0599 0.026 Uiso 1 1 calc R . .
N2 N 0.6328(2) 0.2471(3) 0.3531(3) 0.0201(8) Uani 1 1 d . . .
C1 C 0.5770(3) 0.3227(3) 0.3371(4) 0.0197(9) Uani 1 1 d . . .
H1 H 0.5724 0.3814 0.2890 0.024 Uiso 1 1 calc R . .
N1 N 0.5289(2) 0.3027(3) 0.3997(3) 0.0217(8) Uani 1 1 d . . .
N3 N 0.2041(2) 0.3333(3) 0.1608(3) 0.0217(8) Uani 1 1 d . . .
C28 C 0.8860(3) -0.0244(4) -0.0958(4) 0.0308(11) Uani 1 1 d . . .
H28A H 0.8670 -0.0862 -0.0657 0.046 Uiso 1 1 calc R . .
H28B H 0.9368 -0.0410 -0.1127 0.046 Uiso 1 1 calc R . .
H28C H 0.8426 -0.0017 -0.1659 0.046 Uiso 1 1 calc R . .
C23 C 0.8441(3) 0.0792(3) 0.0347(4) 0.0212(9) Uani 1 1 d . . .
C21 C 0.7104(3) 0.0625(3) 0.0569(4) 0.0199(9) Uani 1 1 d . . .
H21 H 0.6566 0.0318 0.0343 0.024 Uiso 1 1 calc R . .
C20 C 0.7325(3) 0.1346(3) 0.1458(4) 0.0187(9) Uani 1 1 d . . .
C27 C 0.6718(3) 0.1580(3) 0.2038(4) 0.0192(9) Uani 1 1 d . . .
C26 C 0.6957(3) 0.2400(3) 0.2977(4) 0.0235(10) Uani 1 1 d . . .
H26A H 0.7500 0.2218 0.3552 0.028 Uiso 1 1 calc R . .
H26B H 0.7016 0.3082 0.2645 0.028 Uiso 1 1 calc R . .
C19 C 0.4595(3) 0.3671(4) 0.4085(4) 0.0258(10) Uani 1 1 d . . .
H19A H 0.4628 0.4371 0.3773 0.031 Uiso 1 1 calc R . .
H19B H 0.4648 0.3750 0.4899 0.031 Uiso 1 1 calc R . .
C18 C 0.3753(3) 0.3187(4) 0.3436(4) 0.0244(10) Uani 1 1 d . . .
H18A H 0.3310 0.3594 0.3590 0.029 Uiso 1 1 calc R . .
H18B H 0.3735 0.2473 0.3722 0.029 Uiso 1 1 calc R . .
C17 C 0.3576(3) 0.3146(4) 0.2148(4) 0.0266(10) Uani 1 1 d . . .
H17A H 0.4008 0.2717 0.1991 0.032 Uiso 1 1 calc R . .
H17B H 0.3613 0.3856 0.1865 0.032 Uiso 1 1 calc R . .
C16 C 0.2716(3) 0.2692(4) 0.1497(4) 0.0260(10) Uani 1 1 d . . .
H16A H 0.2654 0.2629 0.0678 0.031 Uiso 1 1 calc R . .
H16B H 0.2674 0.1987 0.1790 0.031 Uiso 1 1 calc R . .
C4 C 0.1793(3) 0.4276(3) 0.1047(4) 0.0191(9) Uani 1 1 d . . .
C9 C 0.1132(3) 0.4687(3) 0.1351(4) 0.0206(9) Uani 1 1 d . . .
C8 C 0.0764(3) 0.5621(3) 0.0873(4) 0.0247(10) Uani 1 1 d . . .
H8 H 0.0324 0.5914 0.1081 0.030 Uiso 1 1 calc R . .
C7 C 0.1054(3) 0.6114(4) 0.0085(4) 0.0285(11) Uani 1 1 d . . .
H7 H 0.0808 0.6749 -0.0253 0.034 Uiso 1 1 calc R . .
C15 C 0.1548(3) 0.3138(3) 0.2283(4) 0.0189(9) Uani 1 1 d . . .
C14 C 0.1558(3) 0.2300(4) 0.2999(4) 0.0255(10) Uani 1 1 d . . .
H14 H 0.1950 0.1752 0.3104 0.031 Uiso 1 1 calc R . .

C13 C 0.0981(3) 0.2300(4) 0.3543(4) 0.0263(10) Uani 1 1 d . . .
H13 H 0.0972 0.1731 0.4026 0.032 Uiso 1 1 calc R . .
C12 C 0.0400(3) 0.3103(4) 0.3419(4) 0.0275(11) Uani 1 1 d . . .
H12 H 0.0016 0.3075 0.3824 0.033 Uiso 1 1 calc R . .
C11 C 0.0386(3) 0.3935(4) 0.2709(4) 0.0239(10) Uani 1 1 d . . .
H11 H -0.0013 0.4474 0.2606 0.029 Uiso 1 1 calc R . .
C10 C 0.0974(3) 0.3966(3) 0.2145(4) 0.0201(9) Uani 1 1 d . . .
C6 C 0.1702(3) 0.5685(4) -0.0216(4) 0.0279(11) Uani 1 1 d . . .
H6 H 0.1886 0.6035 -0.0762 0.034 Uiso 1 1 calc R . .
C5 C 0.2087(3) 0.4769(3) 0.0252(4) 0.0240(10) Uani 1 1 d . . .
H5 H 0.2532 0.4487 0.0045 0.029 Uiso 1 1 calc R . .
C3 C 0.5545(3) 0.2109(4) 0.4562(4) 0.0245(10) Uani 1 1 d . . .
H3 H 0.5308 0.1778 0.5065 0.029 Uiso 1 1 calc R . .
C2 C 0.6195(3) 0.1761(3) 0.4274(4) 0.0239(10) Uani 1 1 d . . .
H2 H 0.6502 0.1142 0.4536 0.029 Uiso 1 1 calc R . .
C25 C 0.8117(3) 0.1796(3) 0.1777(4) 0.0209(9) Uani 1 1 d . . .
H25 H 0.8271 0.2300 0.2368 0.025 Uiso 1 1 calc R . .
C24 C 0.8675(3) 0.1518(3) 0.1249(4) 0.0214(9) Uani 1 1 d . . .
H24 H 0.9216 0.1813 0.1489 0.026 Uiso 1 1 calc R . .
O2S O 0.4685(10) 0.4409(12) 0.0897(15) 0.208(5) Uani 1 1 d D . .
H2SA H 0.444(14) 0.428(16) 0.135(17) 0.312 Uiso 1 1 d D . .
H2SB H 0.471(19) 0.5052(19) 0.09(2) 0.312 Uiso 1 1 d D . .

loop_
 _atom_site_aniso_label
 _atom_site_aniso_U_11
 _atom_site_aniso_U_22
 _atom_site_aniso_U_33
 _atom_site_aniso_U_23
 _atom_site_aniso_U_13
 _atom_site_aniso_U_12
Br1 0.0277(3) 0.0259(3) 0.0245(2) -0.0039(2) 0.01027(19) -0.0019(2)
O2 0.0216(17) 0.0340(19) 0.0312(19) -0.0043(15) 0.0129(15) -0.0010(15)
O1S 0.040(2) 0.0345(19) 0.040(2) 0.0028(17) 0.0245(18) 0.0022(17)
O1 0.0234(18) 0.0338(19) 0.033(2) -0.0074(15) 0.0111(15) -0.0090(15)
C22 0.020(2) 0.024(2) 0.019(2) 0.0005(17) 0.0025(19) 0.0009(18)
N2 0.0197(19) 0.0188(18) 0.021(2) 0.0005(15) 0.0056(16) 0.0003(15)
C1 0.019(2) 0.019(2) 0.022(2) 0.0018(17) 0.0070(19) 0.0003(17)
N1 0.0161(19) 0.0185(18) 0.028(2) -0.0034(15) 0.0046(16) 0.0006(15)
N3 0.018(2) 0.0196(18) 0.028(2) 0.0007(15) 0.0083(17) 0.0038(15)
C28 0.023(2) 0.042(3) 0.028(3) -0.004(2) 0.010(2) 0.001(2)
C23 0.017(2) 0.024(2) 0.022(2) 0.0061(18) 0.0070(19) 0.0001(18)
C21 0.014(2) 0.019(2) 0.024(2) 0.0042(17) 0.0027(19) 0.0008(17)
C20 0.017(2) 0.0169(19) 0.021(2) 0.0015(17) 0.0036(18) 0.0009(17)

C27 0.017(2) 0.017(2) 0.019(2) 0.0042(17) 0.0001(18) 0.0016(17)
C26 0.021(2) 0.022(2) 0.030(3) -0.0005(19) 0.013(2) -0.0008(18)
C19 0.023(2) 0.025(2) 0.029(3) -0.004(2) 0.008(2) 0.006(2)
C18 0.020(2) 0.024(2) 0.031(3) -0.0048(19) 0.011(2) 0.0023(19)
C17 0.021(2) 0.025(2) 0.034(3) -0.001(2) 0.010(2) 0.0048(19)
C16 0.023(2) 0.024(2) 0.030(3) -0.011(2) 0.007(2) 0.0020(19)
C4 0.019(2) 0.0160(19) 0.019(2) -0.0010(16) 0.0028(18) -0.0022(17)
C9 0.016(2) 0.0170(19) 0.024(2) -0.0032(17) 0.0013(19) -0.0039(17)
C8 0.019(2) 0.023(2) 0.026(2) -0.0009(18) 0.001(2) 0.0019(18)
C7 0.024(3) 0.020(2) 0.032(3) 0.0040(19) -0.002(2) -0.0019(19)
C15 0.016(2) 0.020(2) 0.019(2) -0.0023(17) 0.0041(18) -0.0017(17)
C14 0.023(2) 0.022(2) 0.028(3) -0.0012(19) 0.003(2) -0.0004(19)
C13 0.021(2) 0.034(3) 0.021(2) 0.0044(19) 0.003(2) -0.008(2)
C12 0.022(2) 0.037(3) 0.023(2) 0.000(2) 0.007(2) -0.007(2)
C11 0.017(2) 0.028(2) 0.025(2) -0.0026(19) 0.005(2) 0.0022(19)
C10 0.016(2) 0.021(2) 0.020(2) -0.0043(17) 0.0030(18) -0.0013(17)
C6 0.030(3) 0.028(2) 0.022(2) 0.0038(19) 0.003(2) -0.011(2)
C5 0.022(2) 0.026(2) 0.025(2) -0.0012(18) 0.008(2) -0.0038(18)
C3 0.024(2) 0.029(2) 0.021(2) 0.0035(19) 0.008(2) 0.001(2)
C2 0.023(2) 0.020(2) 0.029(2) 0.0049(19) 0.009(2) 0.0029(18)
C25 0.019(2) 0.016(2) 0.026(2) 0.0030(17) 0.0056(19) 0.0007(17)
C24 0.014(2) 0.023(2) 0.025(2) 0.0064(18) 0.0038(19) -0.0014(17)
O2S 0.194(12) 0.207(11) 0.267(16) -0.036(12) 0.136(11) -0.014(11)

_geom_special_details
;
 All esds (except the esd in the dihedral angle between two l.s. planes)
 are estimated using the full covariance matrix. The cell esds are taken
 into account individually in the estimation of esds in distances, angles
 and torsion angles; correlations between esds in cell parameters are only
 used when they are defined by crystal symmetry. An approximate (isotropic)
 treatment of cell esds is used for estimating esds involving l.s. planes.
;

loop_
 _geom_bond_atom_site_label_1
 _geom_bond_atom_site_label_2
 _geom_bond_distance
 _geom_bond_site_symmetry_2
 _geom_bond_publ_flag
O2 C23 1.355(5) . ?
O2 C28 1.428(6) . ?
O1S H1SA 0.822(10) . ?
O1S H1SB 0.822(10) . ?

O1 C27 1.218(5) . ?
C22 C23 1.386(6) . ?
C22 C21 1.392(6) . ?
C22 H22 0.9500 . ?
N2 C1 1.328(5) . ?
N2 C2 1.370(6) . ?
N2 C26 1.458(6) . ?
C1 N1 1.330(6) . ?
C1 H1 0.9500 . ?
N1 C3 1.369(6) . ?
N1 C19 1.478(6) . ?
N3 C15 1.393(6) . ?
N3 C4 1.393(5) . ?
N3 C16 1.460(5) . ?
C28 H28A 0.9800 . ?
C28 H28B 0.9800 . ?
C28 H28C 0.9800 . ?
C23 C24 1.410(6) . ?
C21 C20 1.395(6) . ?
C21 H21 0.9500 . ?
C20 C25 1.399(6) . ?
C20 C27 1.475(6) . ?
C27 C26 1.525(6) . ?
C26 H26A 0.9900 . ?
C26 H26B 0.9900 . ?
C19 C18 1.526(7) . ?
C19 H19A 0.9900 . ?
C19 H19B 0.9900 . ?
C18 C17 1.526(7) . ?
C18 H18A 0.9900 . ?
C18 H18B 0.9900 . ?
C17 C16 1.535(7) . ?
C17 H17A 0.9900 . ?
C17 H17B 0.9900 . ?
C16 H16A 0.9900 . ?
C16 H16B 0.9900 . ?
C4 C5 1.401(6) . ?
C4 C9 1.406(6) . ?
C9 C8 1.394(6) . ?
C9 C10 1.444(6) . ?
C8 C7 1.389(7) . ?
C8 H8 0.9500 . ?
C7 C6 1.393(7) . ?
C7 H7 0.9500 . ?

C15 C14 1.394(6) . ?
C15 C10 1.418(6) . ?
C14 C13 1.367(7) . ?
C14 H14 0.9500 . ?
C13 C12 1.405(7) . ?
C13 H13 0.9500 . ?
C12 C11 1.382(7) . ?
C12 H12 0.9500 . ?
C11 C10 1.402(6) . ?
C11 H11 0.9500 . ?
C6 C5 1.380(7) . ?
C6 H6 0.9500 . ?
C5 H5 0.9500 . ?
C3 C2 1.349(6) . ?
C3 H3 0.9500 . ?
C2 H2 0.9500 . ?
C25 C24 1.370(6) . ?
C25 H25 0.9500 . ?
C24 H24 0.9500 . ?
O2S H2SA 0.828(10) . ?
O2S H2SB 0.830(10) . ?

loop_
_geom_angle_atom_site_label_1
_geom_angle_atom_site_label_2
_geom_angle_atom_site_label_3
_geom_angle
_geom_angle_site_symmetry_1
_geom_angle_site_symmetry_3
_geom_angle_publ_flag
C23 O2 C28 118.0(4) . . ?
H1SA O1S H1SB 104(2) . . ?
C23 C22 C21 119.1(4) . . ?
C23 C22 H22 120.4 . . ?
C21 C22 H22 120.4 . . ?
C1 N2 C2 108.6(4) . . ?
C1 N2 C26 125.3(4) . . ?
C2 N2 C26 126.1(4) . . ?
N2 C1 N1 108.7(4) . . ?
N2 C1 H1 125.7 . . ?
N1 C1 H1 125.7 . . ?
C1 N1 C3 108.3(4) . . ?
C1 N1 C19 126.8(4) . . ?
C3 N1 C19 124.9(4) . . ?

C15 N3 C4 108.4(3) . . ?
C15 N3 C16 127.4(4) . . ?
C4 N3 C16 124.2(4) . . ?
O2 C28 H28A 109.5 . . ?
O2 C28 H28B 109.5 . . ?
H28A C28 H28B 109.5 . . ?
O2 C28 H28C 109.5 . . ?
H28A C28 H28C 109.5 . . ?
H28B C28 H28C 109.5 . . ?
O2 C23 C22 124.1(4) . . ?
O2 C23 C24 115.7(4) . . ?
C22 C23 C24 120.2(4) . . ?
C22 C21 C20 121.2(4) . . ?
C22 C21 H21 119.4 . . ?
C20 C21 H21 119.4 . . ?
C21 C20 C25 118.7(4) . . ?
C21 C20 C27 118.3(4) . . ?
C25 C20 C27 123.0(4) . . ?
O1 C27 C20 122.8(4) . . ?
O1 C27 C26 119.6(4) . . ?
C20 C27 C26 117.6(4) . . ?
N2 C26 C27 110.7(4) . . ?
N2 C26 H26A 109.5 . . ?
C27 C26 H26A 109.5 . . ?
N2 C26 H26B 109.5 . . ?
C27 C26 H26B 109.5 . . ?
H26A C26 H26B 108.1 . . ?
N1 C19 C18 111.5(4) . . ?
N1 C19 H19A 109.3 . . ?
C18 C19 H19A 109.3 . . ?
N1 C19 H19B 109.3 . . ?
C18 C19 H19B 109.3 . . ?
H19A C19 H19B 108.0 . . ?
C19 C18 C17 113.0(4) . . ?
C19 C18 H18A 109.0 . . ?
C17 C18 H18A 109.0 . . ?
C19 C18 H18B 109.0 . . ?
C17 C18 H18B 109.0 . . ?
H18A C18 H18B 107.8 . . ?
C18 C17 C16 112.7(4) . . ?
C18 C17 H17A 109.1 . . ?
C16 C17 H17A 109.1 . . ?
C18 C17 H17B 109.1 . . ?
C16 C17 H17B 109.1 . . ?

H17A C17 H17B 107.8 . . ?
N3 C16 C17 112.3(4) . . ?
N3 C16 H16A 109.1 . . ?
C17 C16 H16A 109.1 . . ?
N3 C16 H16B 109.1 . . ?
C17 C16 H16B 109.1 . . ?
H16A C16 H16B 107.9 . . ?
N3 C4 C5 128.9(4) . . ?
N3 C4 C9 109.3(4) . . ?
C5 C4 C9 121.7(4) . . ?
C8 C9 C4 119.8(4) . . ?
C8 C9 C10 133.4(4) . . ?
C4 C9 C10 106.8(4) . . ?
C7 C8 C9 118.7(4) . . ?
C7 C8 H8 120.7 . . ?
C9 C8 H8 120.7 . . ?
C8 C7 C6 120.6(4) . . ?
C8 C7 H7 119.7 . . ?
C6 C7 H7 119.7 . . ?
N3 C15 C14 129.9(4) . . ?
N3 C15 C10 108.8(4) . . ?
C14 C15 C10 121.3(4) . . ?
C13 C14 C15 117.3(4) . . ?
C13 C14 H14 121.4 . . ?
C15 C14 H14 121.4 . . ?
C14 C13 C12 122.8(4) . . ?
C14 C13 H13 118.6 . . ?
C12 C13 H13 118.6 . . ?
C11 C12 C13 120.1(4) . . ?
C11 C12 H12 119.9 . . ?
C13 C12 H12 119.9 . . ?
C12 C11 C10 118.6(4) . . ?
C12 C11 H11 120.7 . . ?
C10 C11 H11 120.7 . . ?
C11 C10 C15 119.8(4) . . ?
C11 C10 C9 133.4(4) . . ?
C15 C10 C9 106.8(4) . . ?
C5 C6 C7 122.3(4) . . ?
C5 C6 H6 118.9 . . ?
C7 C6 H6 118.9 . . ?
C6 C5 C4 116.9(4) . . ?
C6 C5 H5 121.5 . . ?
C4 C5 H5 121.5 . . ?
C2 C3 N1 107.5(4) . . ?

C2 C3 H3 126.3 . . ?
N1 C3 H3 126.3 . . ?
C3 C2 N2 107.0(4) . . ?
C3 C2 H2 126.5 . . ?
N2 C2 H2 126.5 . . ?
C24 C25 C20 121.0(4) . . ?
C24 C25 H25 119.5 . . ?
C20 C25 H25 119.5 . . ?
C25 C24 C23 119.8(4) . . ?
C25 C24 H24 120.1 . . ?
C23 C24 H24 120.1 . . ?
H2SA O2S H2SB 103(3) . . ?

loop_
 _geom_torsion_atom_site_label_1
 _geom_torsion_atom_site_label_2
 _geom_torsion_atom_site_label_3
 _geom_torsion_atom_site_label_4
 _geom_torsion
 _geom_torsion_site_symmetry_1
 _geom_torsion_site_symmetry_2
 _geom_torsion_site_symmetry_3
 _geom_torsion_site_symmetry_4
 _geom_torsion_publ_flag
C2 N2 C1 N1 0.4(5) . . . . ?
C26 N2 C1 N1 178.8(4) . . . . ?
N2 C1 N1 C3 -0.5(5) . . . . ?
N2 C1 N1 C19 179.2(4) . . . . ?
C28 O2 C23 C22 -6.0(6) . . . . ?
C28 O2 C23 C24 173.2(4) . . . . ?
C21 C22 C23 O2 179.1(4) . . . . ?
C21 C22 C23 C24 0.0(6) . . . . ?
C23 C22 C21 C20 0.4(6) . . . . ?
C22 C21 C20 C25 0.3(6) . . . . ?
C22 C21 C20 C27 -178.1(4) . . . . ?
C21 C20 C27 O1 0.8(6) . . . . ?
C25 C20 C27 O1 -177.6(4) . . . . ?
C21 C20 C27 C26 -177.8(4) . . . . ?
C25 C20 C27 C26 3.9(6) . . . . ?
C1 N2 C26 C27 -104.7(5) . . . . ?
C2 N2 C26 C27 73.4(6) . . . . ?
O1 C27 C26 N2 6.5(6) . . . . ?
C20 C27 C26 N2 -174.9(4) . . . . ?
C1 N1 C19 C18 106.1(5) . . . . ?

C3 N1 C19 C18 -74.2(6) . . . . ?
N1 C19 C18 C17 -65.4(5) . . . . ?
C19 C18 C17 C16 -178.0(4) . . . . ?
C15 N3 C16 C17 -104.4(5) . . . . ?
C4 N3 C16 C17 74.5(6) . . . . ?
C18 C17 C16 N3 63.6(5) . . . . ?
C15 N3 C4 C5 -177.5(4) . . . . ?
C16 N3 C4 C5 3.4(7) . . . . ?
C15 N3 C4 C9 -0.6(5) . . . . ?
C16 N3 C4 C9 -179.7(4) . . . . ?
N3 C4 C9 C8 -178.5(4) . . . . ?
C5 C4 C9 C8 -1.3(7) . . . . ?
N3 C4 C9 C10 0.6(5) . . . . ?
C5 C4 C9 C10 177.8(4) . . . . ?
C4 C9 C8 C7 1.2(6) . . . . ?
C10 C9 C8 C7 -177.6(5) . . . . ?
C9 C8 C7 C6 -0.3(7) . . . . ?
C4 N3 C15 C14 -179.9(4) . . . . ?
C16 N3 C15 C14 -0.9(7) . . . . ?
C4 N3 C15 C10 0.3(5) . . . . ?
C16 N3 C15 C10 179.4(4) . . . . ?
N3 C15 C14 C13 -178.4(4) . . . . ?
C10 C15 C14 C13 1.4(7) . . . . ?
C15 C14 C13 C12 -0.9(7) . . . . ?
C14 C13 C12 C11 1.0(7) . . . . ?
C13 C12 C11 C10 -1.5(7) . . . . ?
C12 C11 C10 C15 2.0(6) . . . . ?
C12 C11 C10 C9 179.0(5) . . . . ?
N3 C15 C10 C11 177.8(4) . . . . ?
C14 C15 C10 C11 -2.0(7) . . . . ?
N3 C15 C10 C9 0.1(5) . . . . ?
C14 C15 C10 C9 -179.7(4) . . . . ?
C8 C9 C10 C11 1.2(9) . . . . ?
C4 C9 C10 C11 -177.7(5) . . . . ?
C8 C9 C10 C15 178.5(5) . . . . ?
C4 C9 C10 C15 -0.4(5) . . . . ?
C8 C7 C6 C5 -0.5(7) . . . . ?
C7 C6 C5 C4 0.4(7) . . . . ?
N3 C4 C5 C6 177.0(4) . . . . ?
C9 C4 C5 C6 0.5(7) . . . . ?
C1 N1 C3 C2 0.4(5) . . . . ?
C19 N1 C3 C2 -179.4(4) . . . . ?
N1 C3 C2 N2 -0.2(5) . . . . ?
C1 N2 C2 C3 -0.2(5) . . . . ?

C26 N2 C2 C3 -178.5(4) . . . . ?
C21 C20 C25 C24 -1.5(6) . . . . ?
C27 C20 C25 C24 176.8(4) . . . . ?
C20 C25 C24 C23 1.9(6) . . . . ?
O2 C23 C24 C25 179.6(4) . . . . ?
C22 C23 C24 C25 -1.2(6) . . . . ?

_diffrn_measured_fraction_theta_max 0.992
_diffrn_reflns_theta_full 28.25
_diffrn_measured_fraction_theta_full 0.992
_refine_diff_density_max 1.337
_refine_diff_density_min -1.297
_refine_diff_density_rms 0.127

4. Biological Assay Procedures and Results

4.1 MTS assay.

Cytotoxicity was determined by performing MTS assay. Briefly, 100 ml of cells suspension were seeded in 96-well cell culture plates and allowed to adhere overnight. The cells were treated with drugs for 48 hours, and then 20 ml of CellTiter 96® AQ_{ueous} One Solution Reagent (Promega, Madison, USA) was added and the cells were further incubated at 37 °C for 1–2 h. Cell viability was measured by reading the absorbance at a wavelength of 490 nm. Concentrations of 50% inhibition of growth (IC50) were calculated on the basis of the relative survival curve.

Entry	Compound	HL-60	SMMC-7721	A549	MCF-7	SW480
1	**5**	>40	27.31	>40	>40	>40
2	**6**	7.91	21.59	25.96	13.99	25.84
3	**7**	ND[c]	ND	ND	ND	ND
4	**8**	21.89	>40	37.38	>40	>40
5	**9**	3.11	3.21	12.36	5.06	18.25
6	**10**	20.58	20.31	19.36	17.80	20.01
7	**11**	14.43	>40	31.86	20.74	>40
8	**12**	14.11	>40	37.97	27.65	>40
9	**13**	14.71	14.10	17.64	18.79	17.47
10	**14**	6.23	24.62	>40	12.39	>40
11	**15**	2.44	13.83	25.11	8.78	19.61
12	**16**	2.79	6.99	15.44	4.60	9.53
13	**17**	3.38	11.89	19.62	8.74	12.49
14	**18**	3.09	13.48	24.78	8.25	12.20
15	**19**	2.15	13.65	19.82	6.90	14.98
16	**20**	3.22	15.79	25.87	13.99	15.00
17	**21**	2.28	11.58	15.57	5.92	12.26
18	**22**	2.80	3.27	5.65	2.69	3.28
19	**23**	2.95	15.67	18.19	3.88	9.57
20	**24**	1.17	10.24	12.66	3.85	5.22
21	**25**	1.94	8.54	12.24	3.78	7.41
22	**26**	1.74	3.19	3.89	2.66	3.32
23	**27**	1.99	6.59	11.11	2.46	3.38
24	**28**	9.93	4.89	9.14	10.10	13.67
25	**29**	ND	ND	ND	ND	ND
26	**30**	1.34	8.41	11.07	2.54	11.74
27	**31**	2.42	10.22	15.70	3.95	14.16
28	**32**	2.98	11.69	19.04	19.98	16.39
29	**33**	0.84	5.74	3.92	2.24	9.56

30	**34**	0.49	3.04	2.92	1.95	4.33
31	**35**	2.37	3.53	2.80	2.41	3.33
32	**36**	0.56	2.78	5.16	2.39	3.37
33	**37**	2.30	3.56	3.74	2.54	2.80
34	**38**	0.98	6.32	12.94	2.98	3.84
35	**39**	2.60	3.57	3.15	2.32	3.59
36	**40**	0.71	3.66	3.58	2.14	3.08
37	**41**	3.34	2.41	3.16	1.65	2.50
38	**42**	3.71	2.34	3.60	1.78	2.31
39	**43**	1.80	3.71	4.40	3.35	3.38
40	**44**	0.56	3.74	6.32	2.88	2.97
41	**45**	0.54	2.78	2.83	4.49	5.62
42	**46**	0.70	3.30	3.10	4.10	6.58
43	**47**	0.68	6.34	4.83	3.04	8.69
44	**48**	0.87	2.93	2.99	2.59	4.50
45	**49**	0.55	3.05	2.29	1.91	4.45
46	**50**	2.67	5.41	14.03	3.13	3.83
47	**51**	0.66	2.16	2.80	1.60	2.43
48	**52**	1.36	2.58	3.02	2.25	3.40
49	**53**	2.19	2.88	3.89	3.88	3.39
50	**54**	0.57	2.55	2.65	2.82	3.19
51	**55**	0.64	2.16	3.00	2.39	2.54
52	**56**	1.25	3.31	4.19	3.21	3.48
53	**57**	0.94	2.83	3.39	2.50	3.58
54	**58**	0.76	2.21	2.98	1.94	3.23
55	**59**	2.60	2.71	3.74	3.32	3.64
56	**60**	0.56	2.00	2.84	2.10	2.88
57	**61**	0.51	2.38	3.12	1.40	2.48
58	DDP	1.32	6.24	11.83	15.17	12.95

[a] Cytotoxicity as IC_{50} for each cell line, is the concentration of compound which reduced by 50% the optical density of treated cells with respect to untreated cells using the MTT assay.

[b] Data represent the mean values of three independent determinations.

[c] ND: not determined.

4.2 Cell apoptosis assay.

To analyze the cells for apoptosis, cells were plated and allowed to adhere overnight. Cells were treated with drugs indicated for 48 hours and then analyzed for apoptosis using Annexin-V-FITC/Propidium iodide staining. Cells were trypsinized, leted, washed in PBS, and resuspended in 1×binding buffer containing Apelnnexin-V-FITC and propidium iodide (BD Pharmingen) according to the

manufacturer's instructions. The samples were analyzed for the apoptosis using a FACSCalibur flow cytometer (BD Biosciences, Franklin Lakes, NJ).

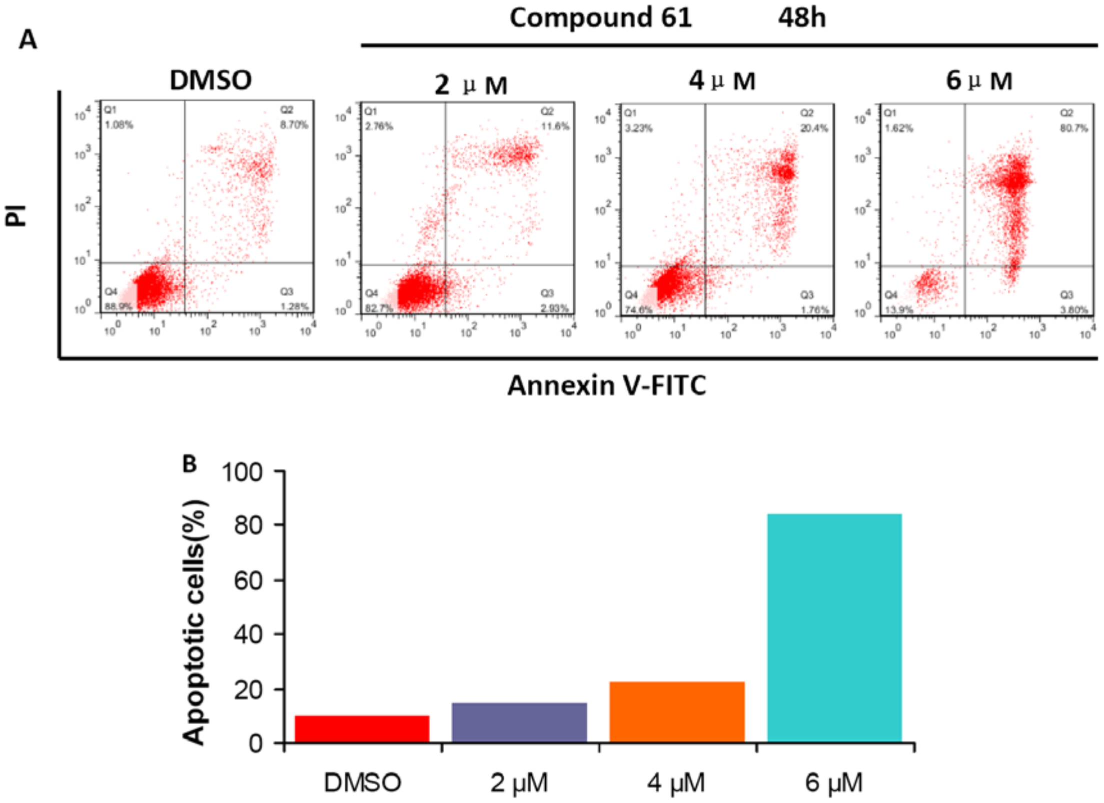

Compound 61 induce significant apoptosis of SMMC-7721 cells. (A) Cells were treated with 2, 4 and 6 μM compound **61** for 48 h. Treatment with **61** increased the early apoptotic (Annexin V+/PI−, lower right quadrant) and late apoptotic (Annexin V+/PI+, upper right quadrant) cells. (B) The quantification of cell apoptosis. Data represents the mean of three independent experiments.

4.3 Cell cycle analysis.

To analyze the DNA content by flow cytometry, cells were collected and washed twice with PBS. Cells were fixed with 70% ethanol overnight. Fixed cells were washed with PBS, and then stained with a 50 μg/ml propidium iodide (PI) solution containing 50 μg/ml RNase A for 30 min at room temperature. Fluorescence intensity was analyzed by FACSCalibur flow cytometer (BD Biosciences, San Jose, CA, USA).

The percentages of the cells distributed in different phases of the cell cycle were determined using ModFIT LT 2.0.

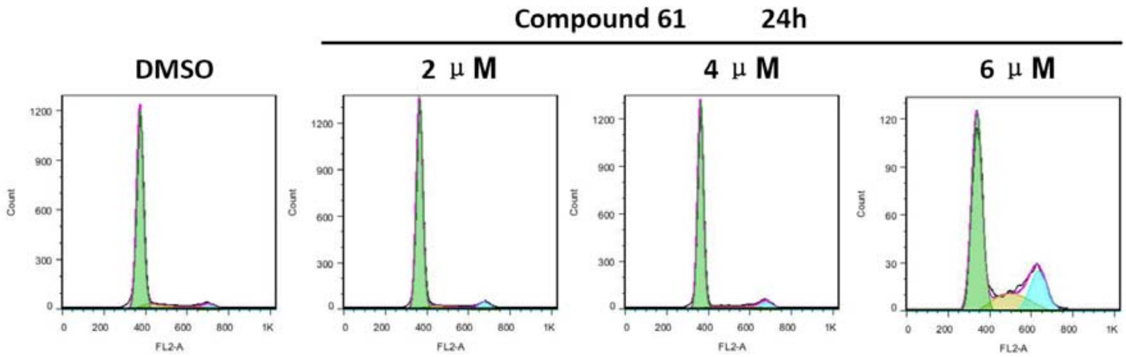

Treatment	Cells (%)		
	G0/G1	S	G2/M
DMSO	81.98	12.28	3.18
Compound **61** (2 μM)	85.85	6.75	5.70
Compound **61** (4 μM)	82.33	9.57	6.13
Compound **61** (6 μM)	61.88	18.50	20.46

Compound 61 induces G2/M phase arrest in SMMC-7721 cells. (A) Cells were treated with 2, 4 and 6 μM of compound **61** for 24 h. Cell cycle was determined by PI staining and cell cytometry. (B) The percentages of cells in different phases were quantified. At least three independent experiments were performed and data of one representative experiment is shown.

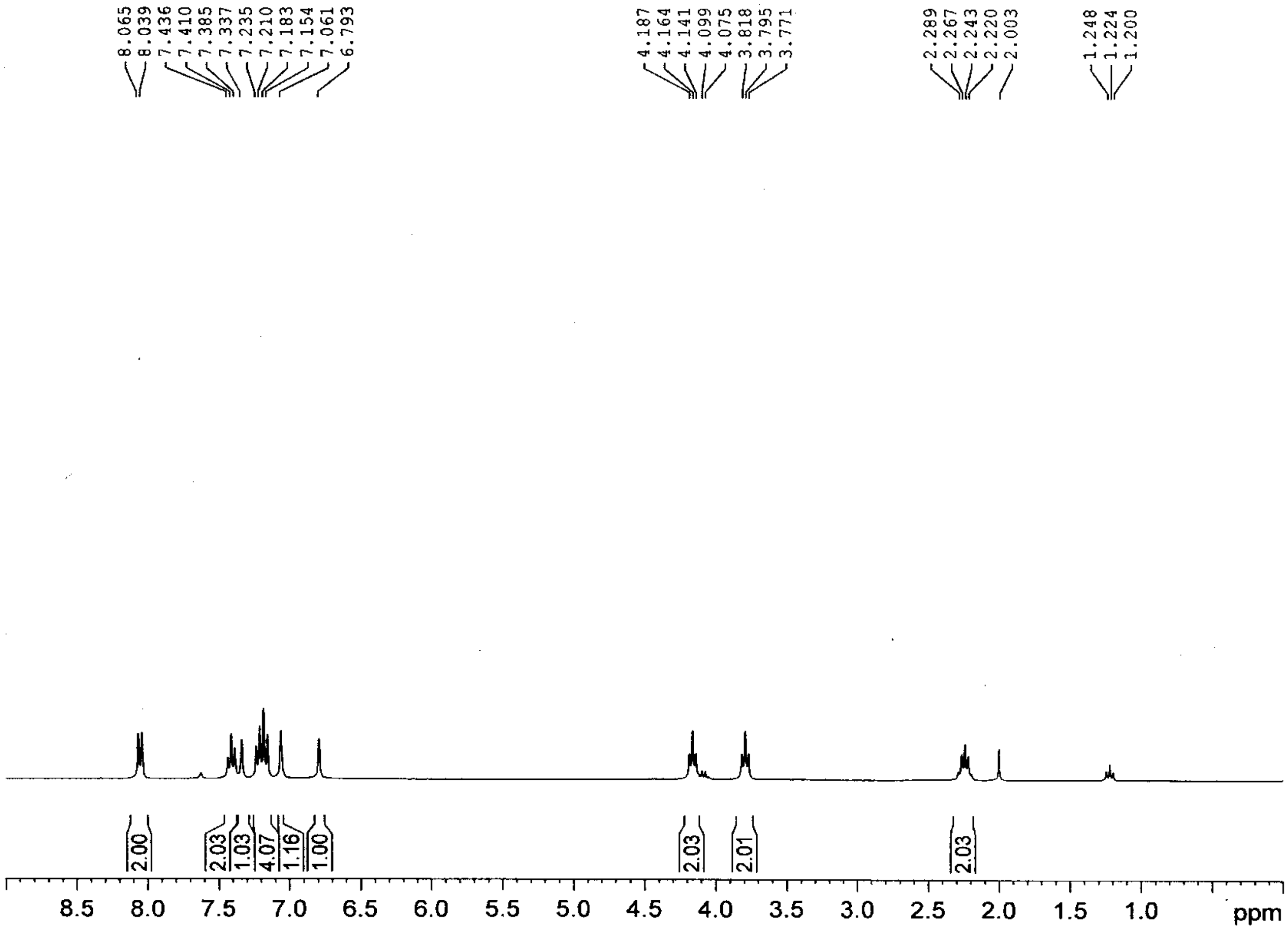

NAME	11x
EXPNO	428
PROCNO	1
Date_	20120813
Time	21.52
INSTRUM	av300
PROBHD	5 mm QNP 1H/13
PULPROG	zg30
TD	65536
SOLVENT	CDCl3
NS	16
DS	2
SWH	6172.839 Hz
FIDRES	0.094190 Hz
AQ	5.3084660 sec
RG	71.8
DW	81.000 usec
DE	6.50 usec
TE	300.9 K
D1	1.00000000 sec
TD0	1
======== CHANNEL f1 ========	
NUC1	1H
P1	7.90 usec
PL1	-2.00 dB
SFO1	300.1318534 MHz
SI	32768
SF	300.1300184 MHz
WDW	EM
SSB	0
LB	0.30 Hz
GB	0
PC	1.00

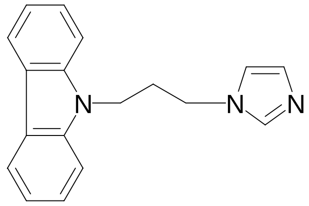

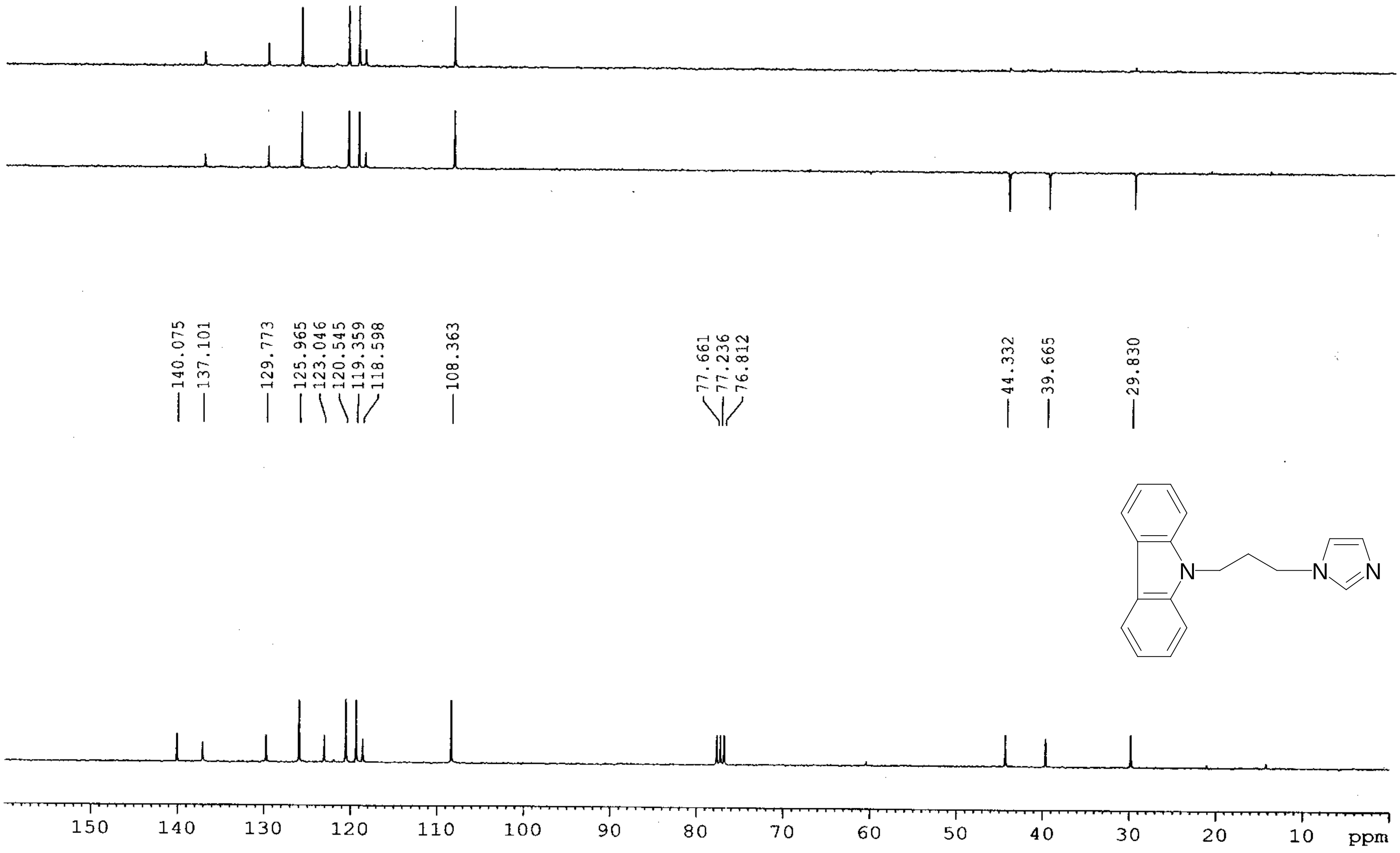
140.075
137.101
129.773
125.965
123.046
120.545
119.359
118.598
108.363
77.661
77.236
76.812
44.332
39.665
29.830
N
N
N
150
140
130
120
110
100
90
80
70
60
50
40
30
20
10
ppm

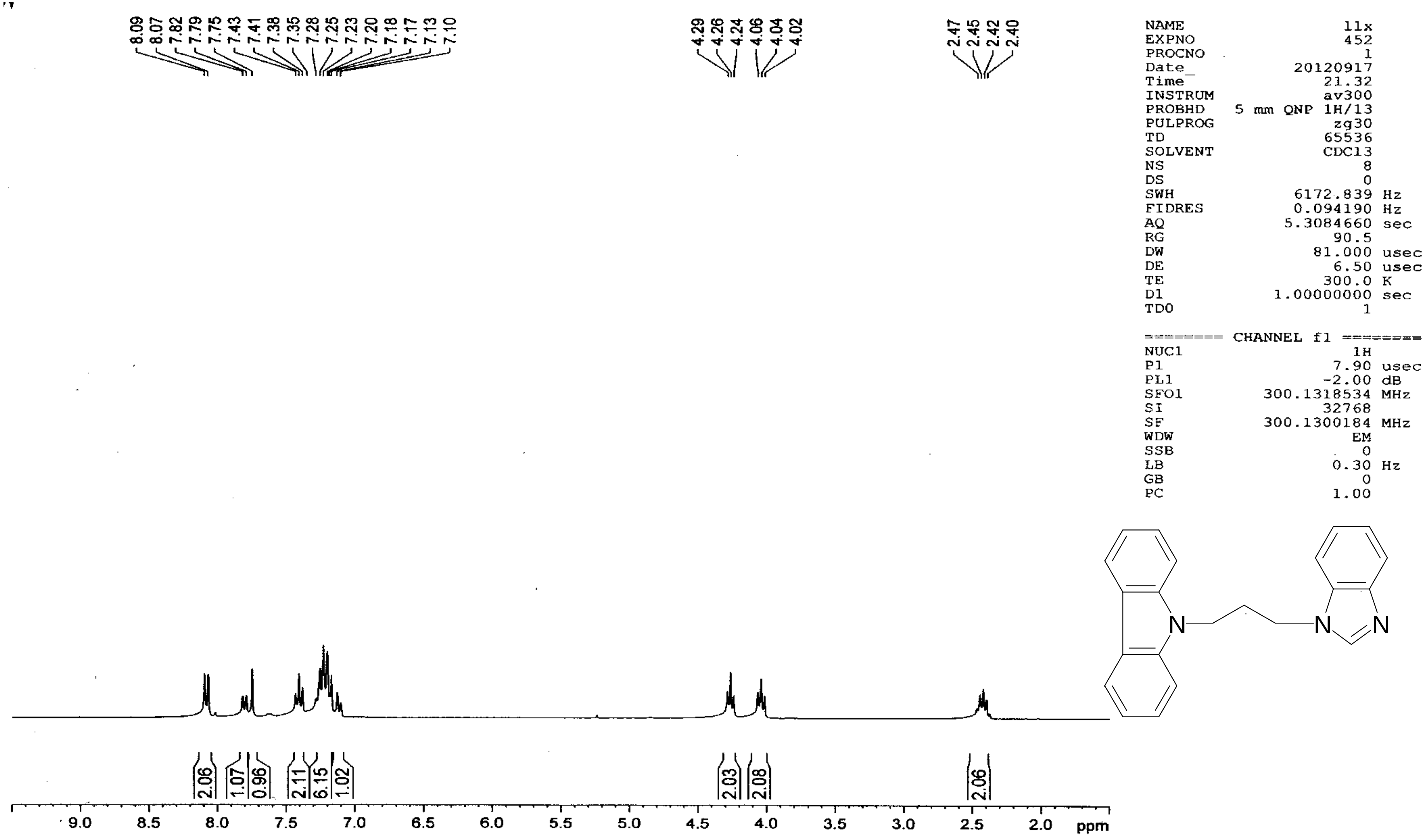
8.09 8.07 7.82 7.79 7.75 7.43 7.41 7.38 7.35 7.28 7.25 7.23 7.20 7.18 7.17 7.13 7.10
4.29 4.26 4.24 4.06 4.04 4.02
2.47 2.45 2.42 2.40
NAME 11x
EXPNO 452
PROCNO 1
Date_ 20120917
Time 21.32
INSTRUM av300
PROBHD 5 mm QNP 1H/13
PULPROG zg30
TD 65536
SOLVENT CDCl3
NS 8
DS 0
SWH 6172.839 Hz
FIDRES 0.094190 Hz
AQ 5.3084660 sec
RG 90.5
DW 81.000 usec
DE 6.50 usec
TE 300.0 K
D1 1.00000000 sec
TD0 1
======== CHANNEL f1 ========
NUC1 1H
P1 7.90 usec
PL1 -2.00 dB
SFO1 300.1318534 MHz
SI 32768
SF 300.1300184 MHz
WDW EM
SSB 0
LB 0.30 Hz
GB 0
PC 1.00
N
N
N
2.06 1.07 0.96 2.11 6.15 1.02 2.03 2.08 2.06
9.0 8.5 8.0 7.5 7.0 6.5 6.0 5.5 5.0 4.5 4.0 3.5 3.0 2.5 2.0 ppm

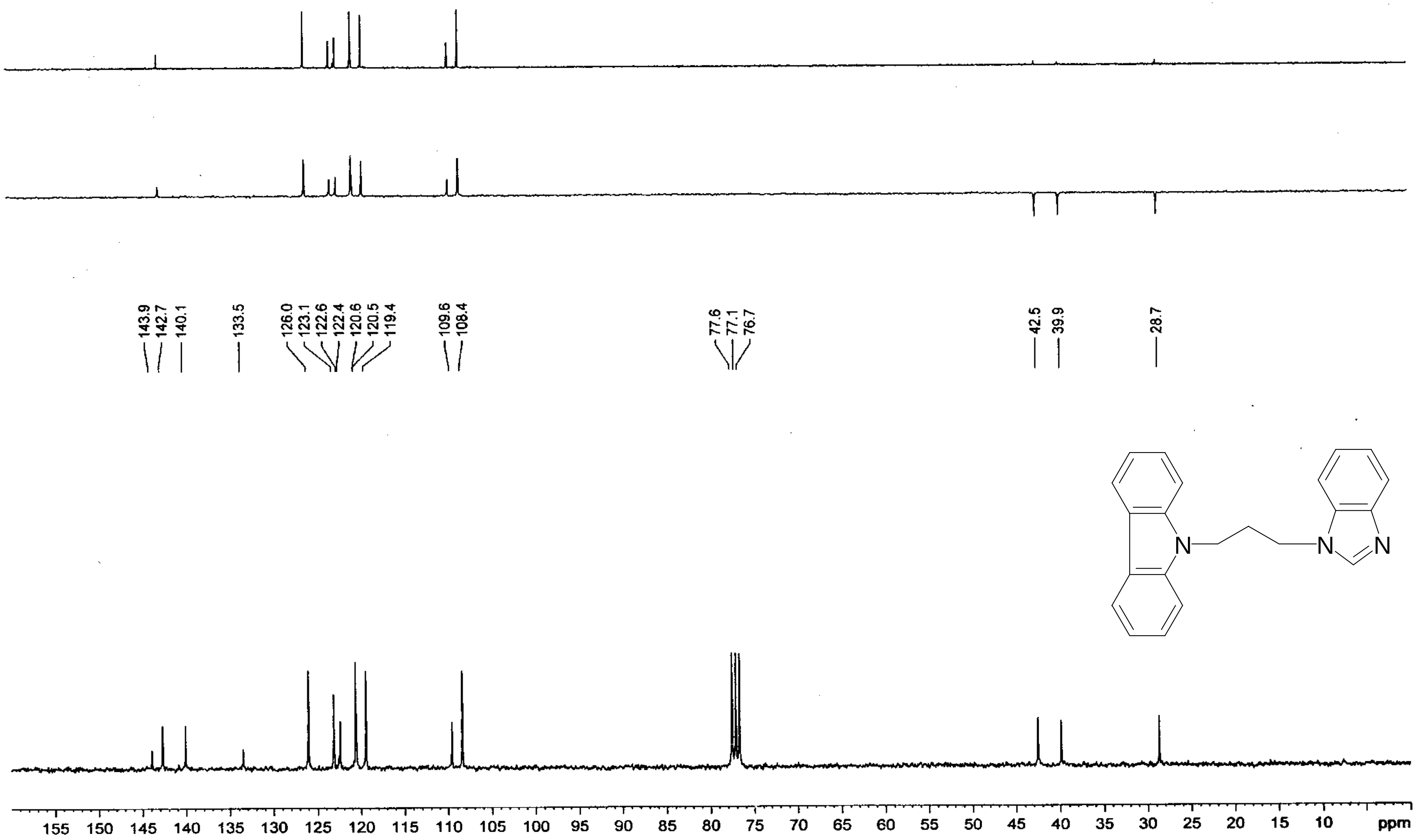
143.9
142.7
140.1
133.5
126.0
123.1
122.6
122.4
120.6
120.5
119.4
109.6
108.4
77.6
77.1
76.7
42.5
39.9
28.7
N
N
N
155 150 145 140 135 130 125 120 115 110 105 100 95 90 85 80 75 70 65 60 55 50 45 40 35 30 25 20 15 10 ppm

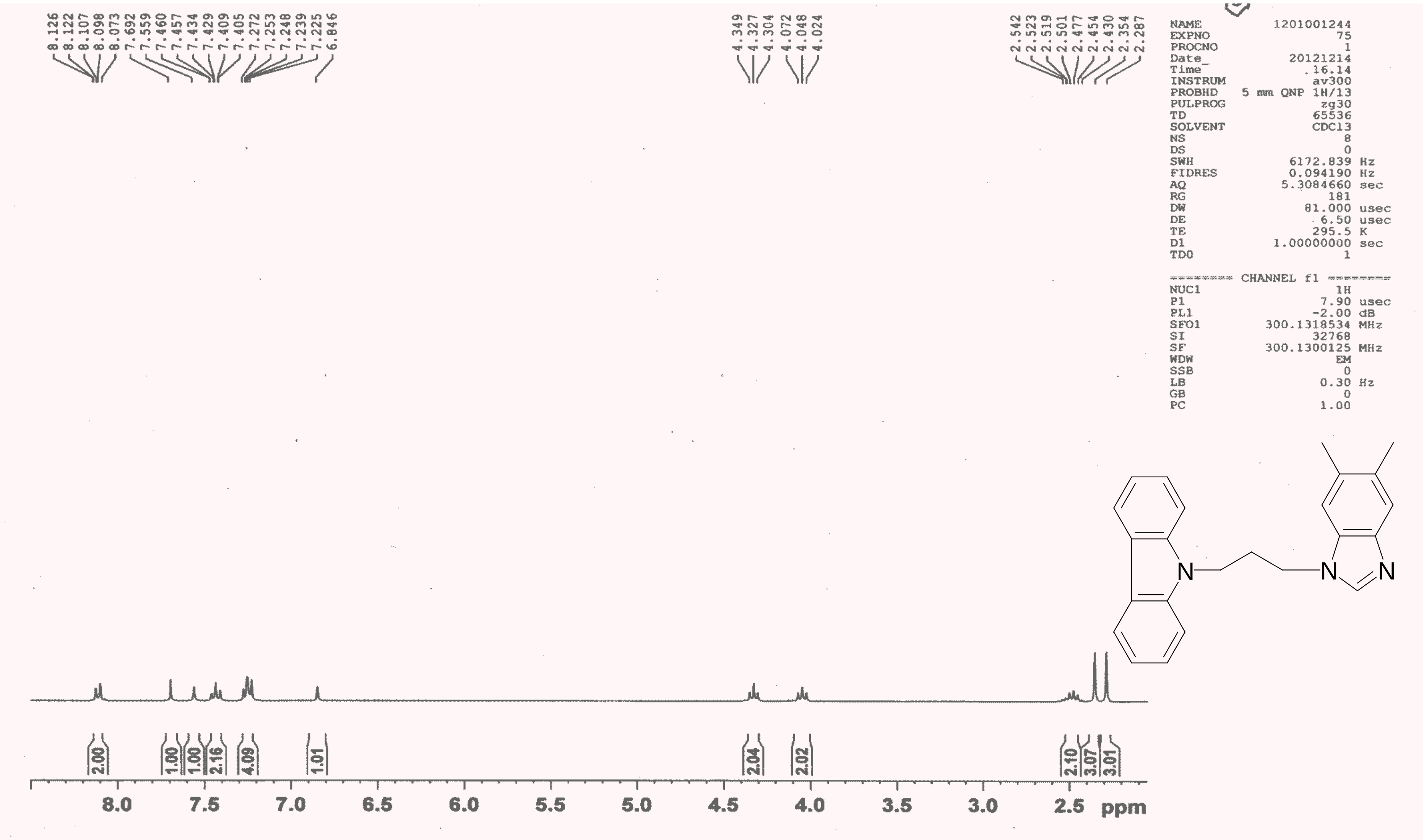

8.126 8.122 8.107 8.098 8.073 7.692 7.559 7.460 7.457 7.434 7.429 7.409 7.405 7.272 7.253 7.248 7.239 7.225 6.846
4.349 4.327 4.304 4.072 4.048 4.024
2.542 2.523 2.519 2.501 2.477 2.454 2.430 2.354 2.287
NAME 1201001244
EXPNO 75
PROCNO 1
Date_ 20121214
Time 16.14
INSTRUM av300
PROBHD 5 mm QNP 1H/13
PULPROG zg30
TD 65536
SOLVENT CDCl3
NS 8
DS 0
SWH 6172.839 Hz
FIDRES 0.094190 Hz
AQ 5.3084660 sec
RG 181
DW 81.000 usec
DE 6.50 usec
TE 295.5 K
D1 1.00000000 sec
TD0 1
======== CHANNEL f1 ========
NUC1 1H
P1 7.90 usec
PL1 -2.00 dB
SFO1 300.1318534 MHz
SI 32768
SF 300.1300125 MHz
WDW EM
SSB 0
LB 0.30 Hz
GB 0
PC 1.00
N
N
N
2.00 1.00 1.00 2.16 4.09 1.01 2.04 2.02 2.10 3.07 3.01
8.0 7.5 7.0 6.5 6.0 5.5 5.0 4.5 4.0 3.5 3.0 2.5 ppm

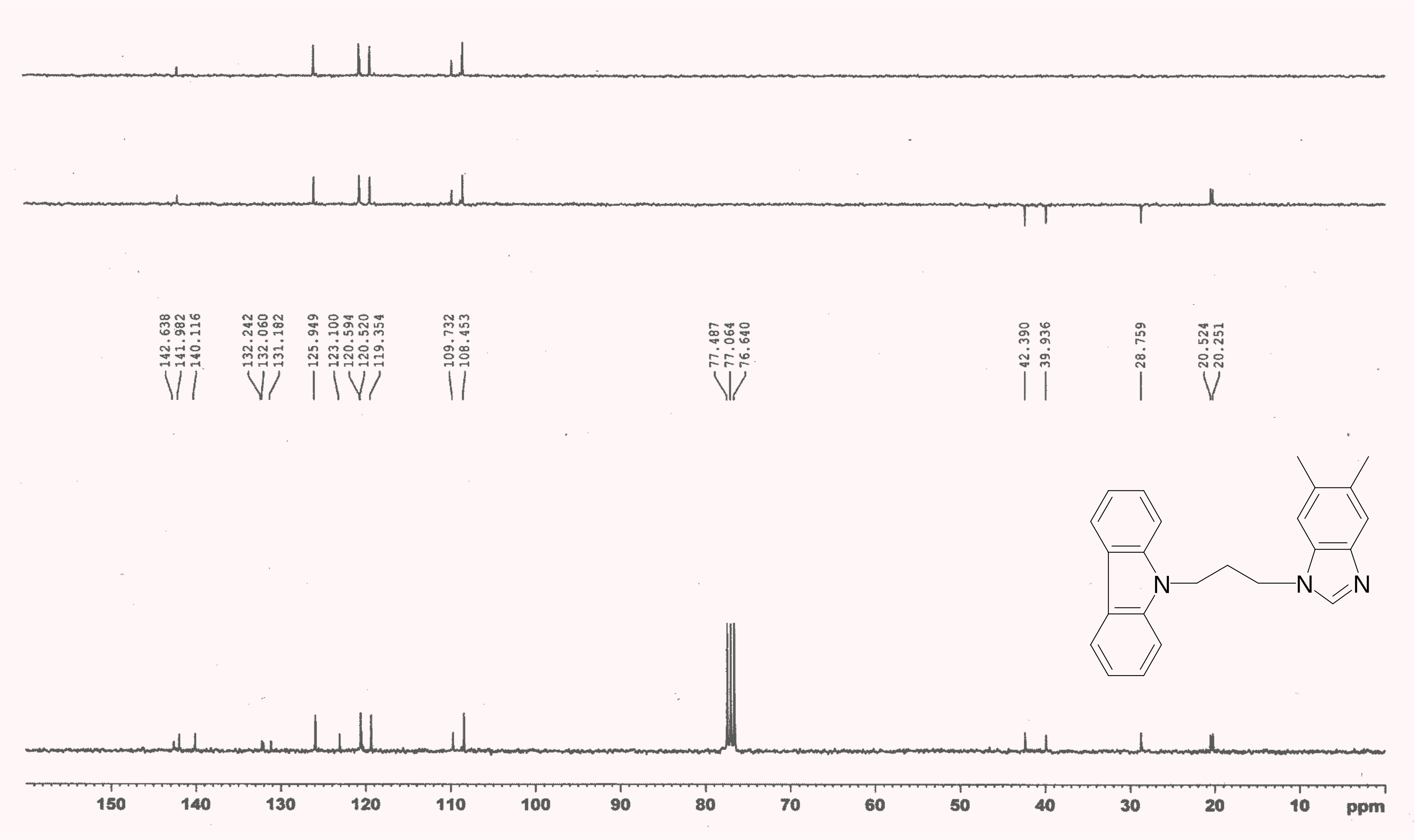
142.638
141.982
140.116
132.242
132.060
131.182
125.949
123.100
120.594
120.520
119.354
109.732
108.453
77.487
77.064
76.640
42.390
39.936
28.759
20.524
20.251
N
N
N
150
140
130
120
110
100
90
80
70
60
50
40
30
20
10
ppm

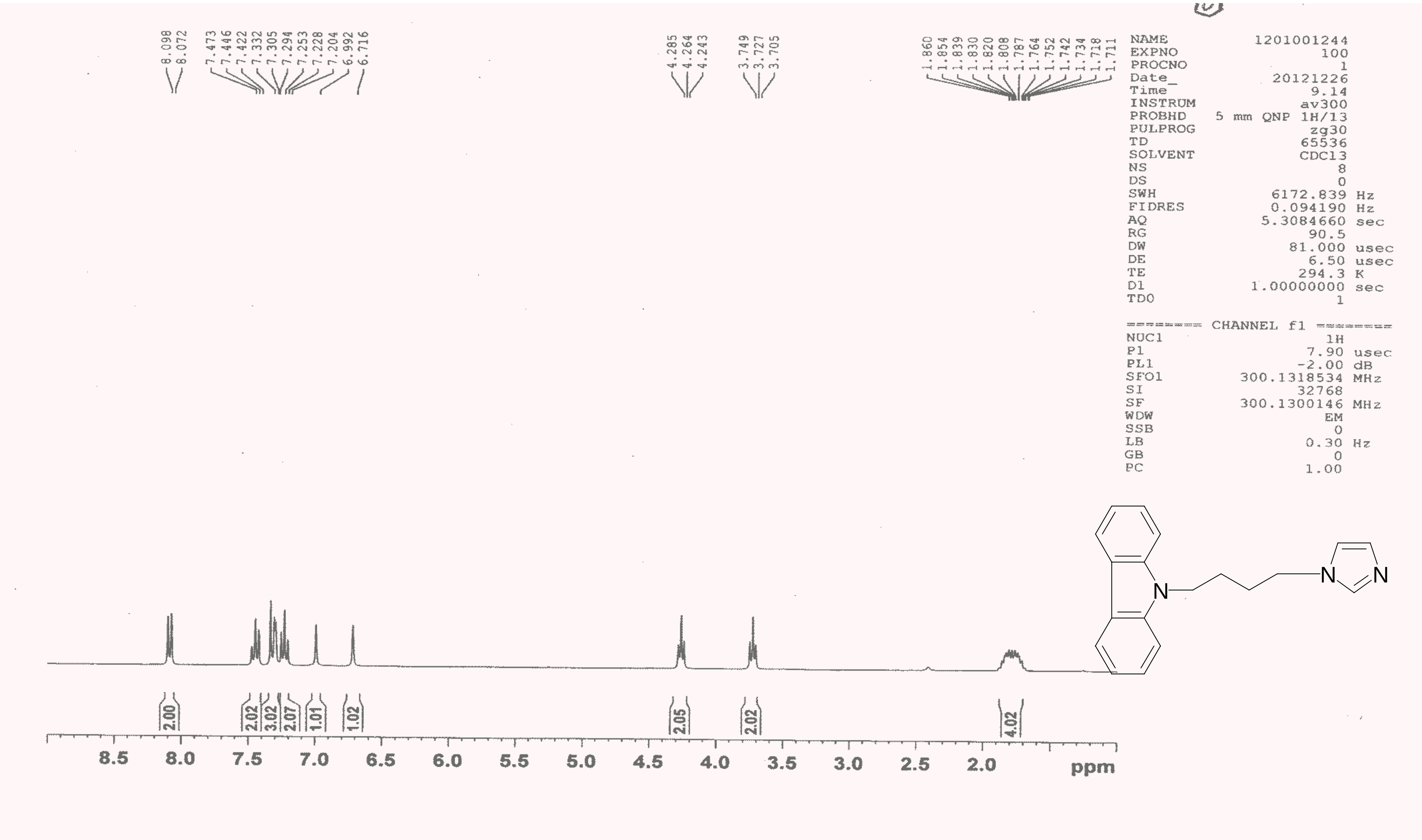
8.098
8.072
7.473
7.446
7.422
7.332
7.305
7.294
7.253
7.228
7.204
6.992
6.716
4.285
4.264
4.243
3.749
3.727
3.705
1.860
1.854
1.839
1.830
1.820
1.808
1.787
1.764
1.752
1.742
1.734
1.718
1.711
NAME 1201001244
EXPNO 100
PROCNO 1
Date_ 20121226
Time 9.14
INSTRUM av300
PROBHD 5 mm QNP 1H/13
PULPROG zg30
TD 65536
SOLVENT CDCl3
NS 8
DS 0
SWH 6172.839 Hz
FIDRES 0.094190 Hz
AQ 5.3084660 sec
RG 90.5
DW 81.000 usec
DE 6.50 usec
TE 294.3 K
D1 1.00000000 sec
TD0 1
======== CHANNEL f1 ========
NUC1 1H
P1 7.90 usec
PL1 -2.00 dB
SFO1 300.1318534 MHz
SI 32768
SF 300.1300146 MHz
WDW EM
SSB 0
LB 0.30 Hz
GB 0
PC 1.00
N
N
N
2.00
2.02
3.02
2.07
1.01
1.02
2.05
2.02
4.02
8.5
8.0
7.5
7.0
6.5
6.0
5.5
5.0
4.5
4.0
3.5
3.0
2.5
2.0
ppm

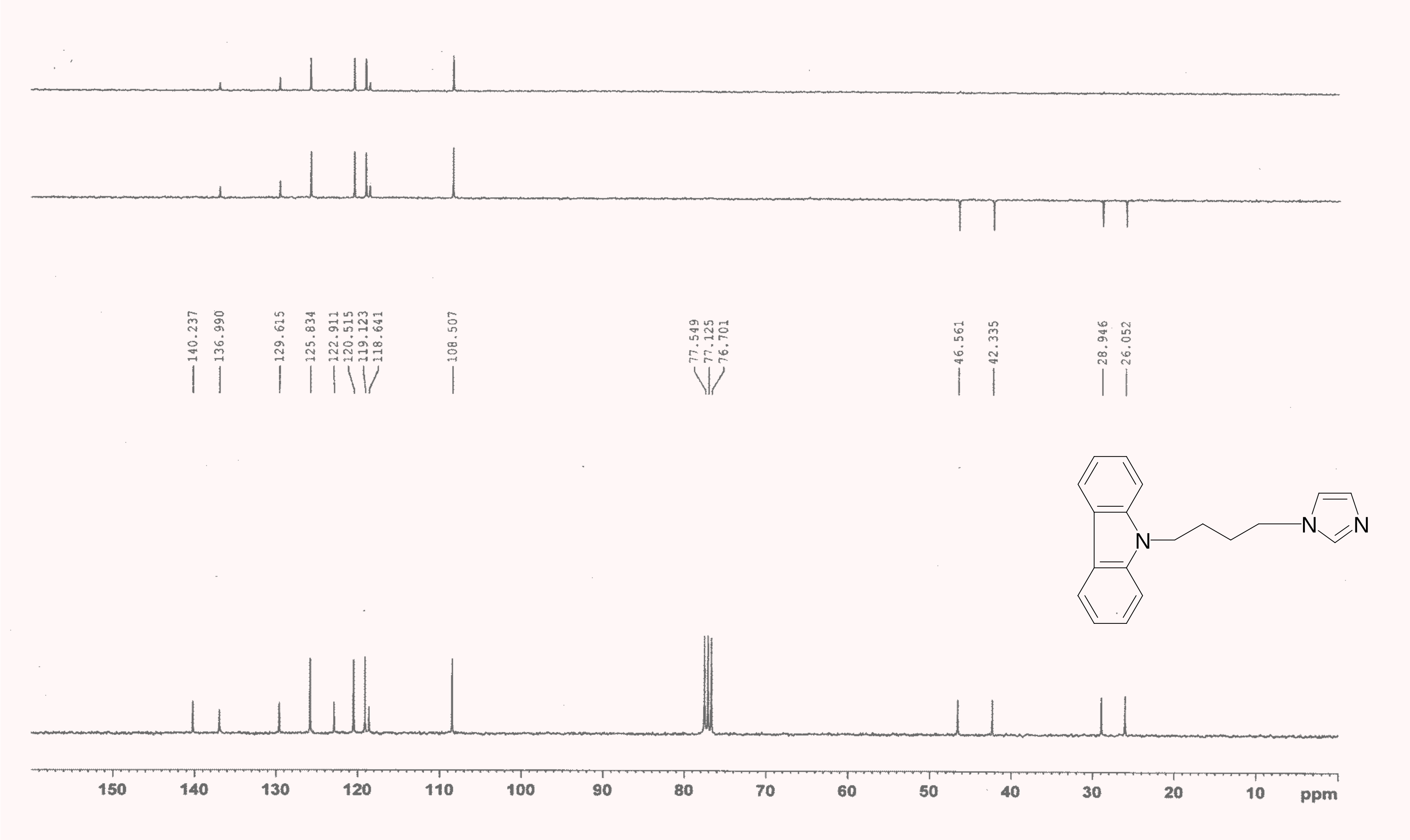
140.237
136.990
129.615
125.834
122.911
120.515
119.123
118.641
108.507
77.549
77.125
76.701
46.561
42.335
28.946
26.052
N
N
N
150
140
130
120
110
100
90
80
70
60
50
40
30
20
10
ppm

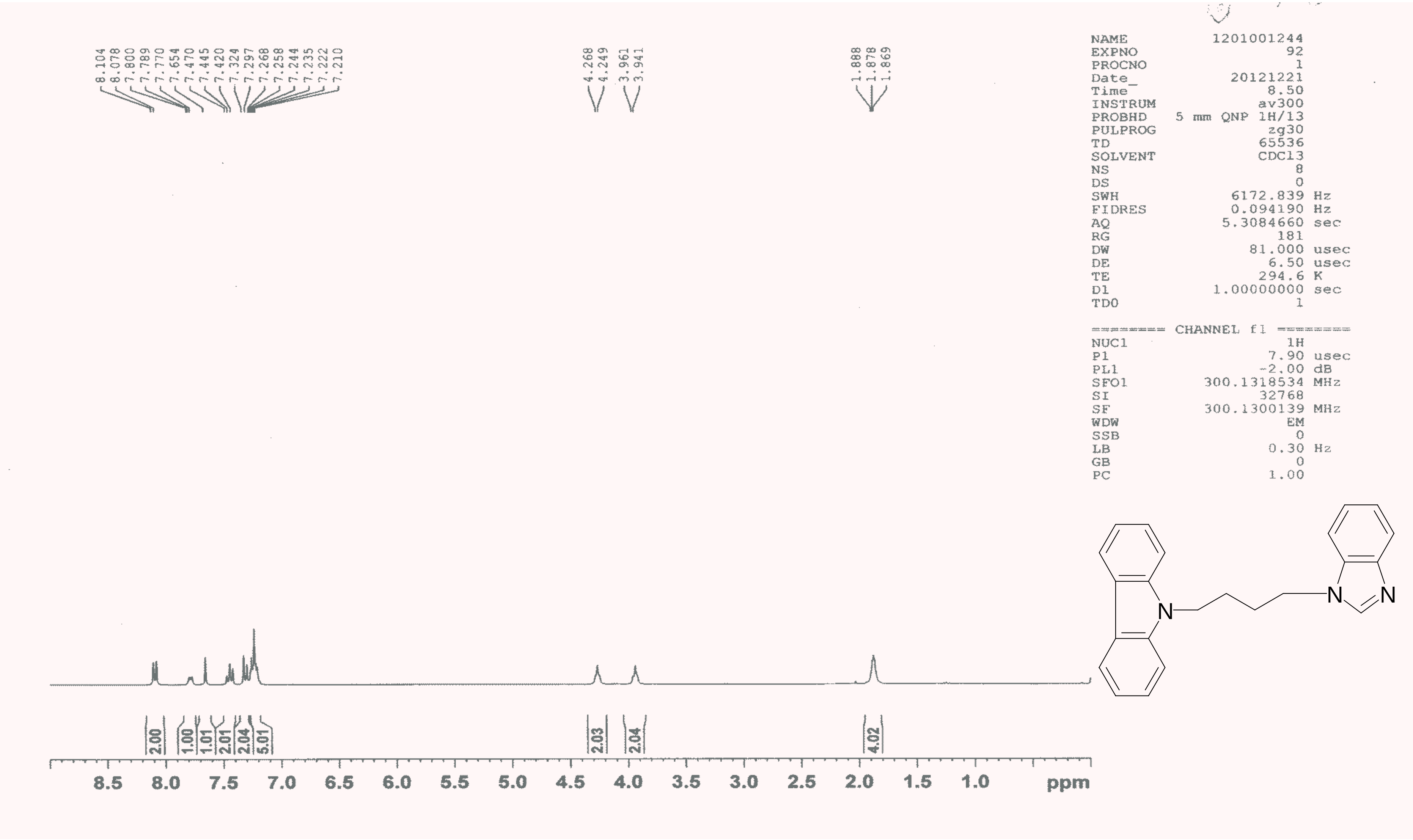
8.104
8.078
7.800
7.789
7.770
7.654
7.470
7.445
7.420
7.324
7.297
7.268
7.258
7.244
7.235
7.222
7.210
4.268
4.249
3.961
3.941
1.888
1.878
1.869
NAME 1201001244
EXPNO 92
PROCNO 1
Date_ 20121221
Time 8.50
INSTRUM av300
PROBHD 5 mm QNP 1H/13
PULPROG zg30
TD 65536
SOLVENT CDCl3
NS 8
DS 0
SWH 6172.839 Hz
FIDRES 0.094190 Hz
AQ 5.3084660 sec
RG 181
DW 81.000 usec
DE 6.50 usec
TE 294.6 K
D1 1.00000000 sec
TD0 1
======== CHANNEL f1 ========
NUC1 1H
P1 7.90 usec
PL1 -2.00 dB
SFO1 300.1318534 MHz
SI 32768
SF 300.1300139 MHz
WDW EM
SSB 0
LB 0.30 Hz
GB 0
PC 1.00
N
N
N
2.00
1.00
1.01
2.01
2.04
5.01
2.03
2.04
4.02
8.5
8.0
7.5
7.0
6.5
6.0
5.5
5.0
4.5
4.0
3.5
3.0
2.5
2.0
1.5
1.0
ppm

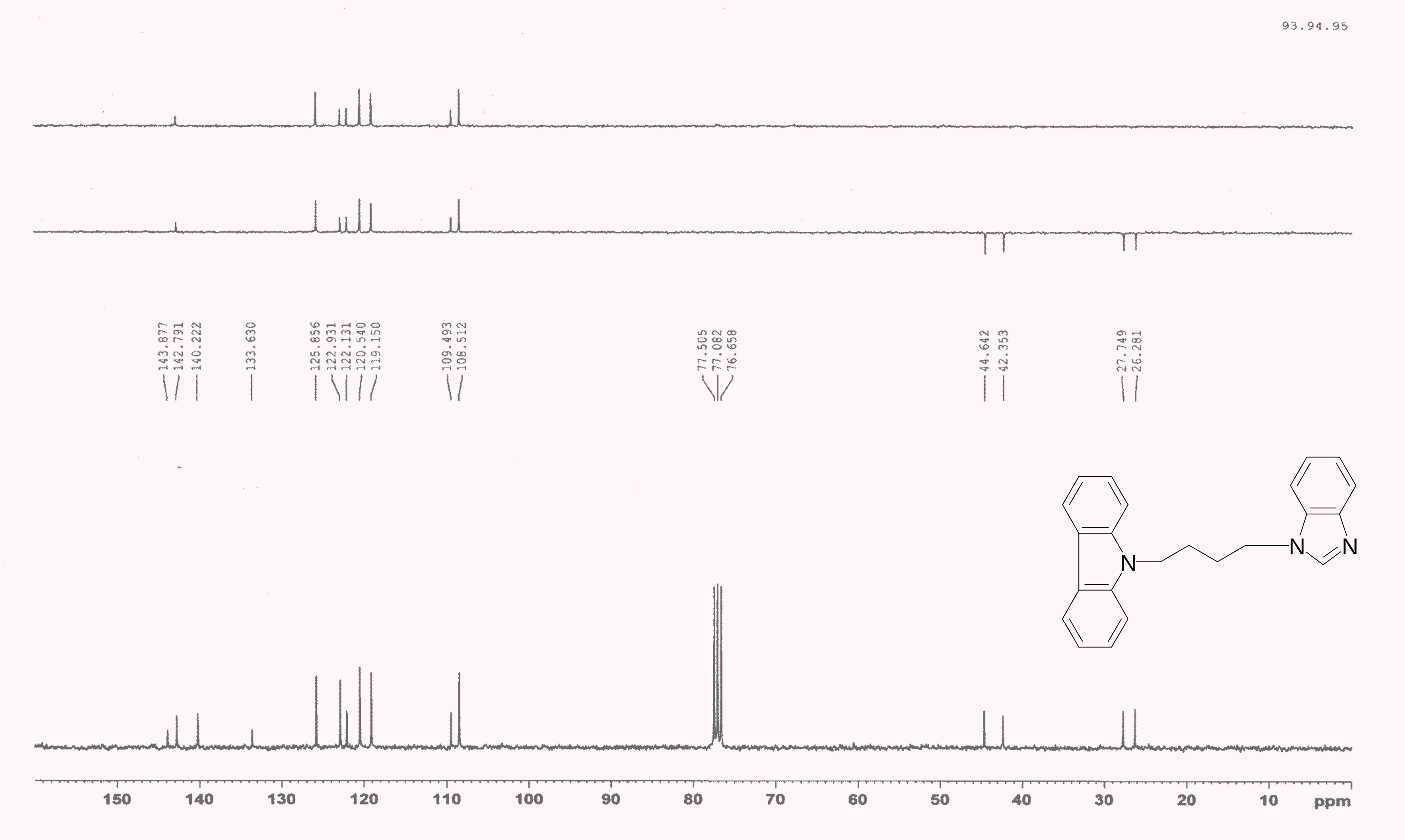
93.94.95
143.877
142.791
140.222
133.630
125.856
122.931
122.131
120.540
119.150
109.493
108.512
77.505
77.082
76.658
44.642
42.353
27.749
26.281
N
N
N
150
140
130
120
110
100
90
80
70
60
50
40
30
20
10
ppm

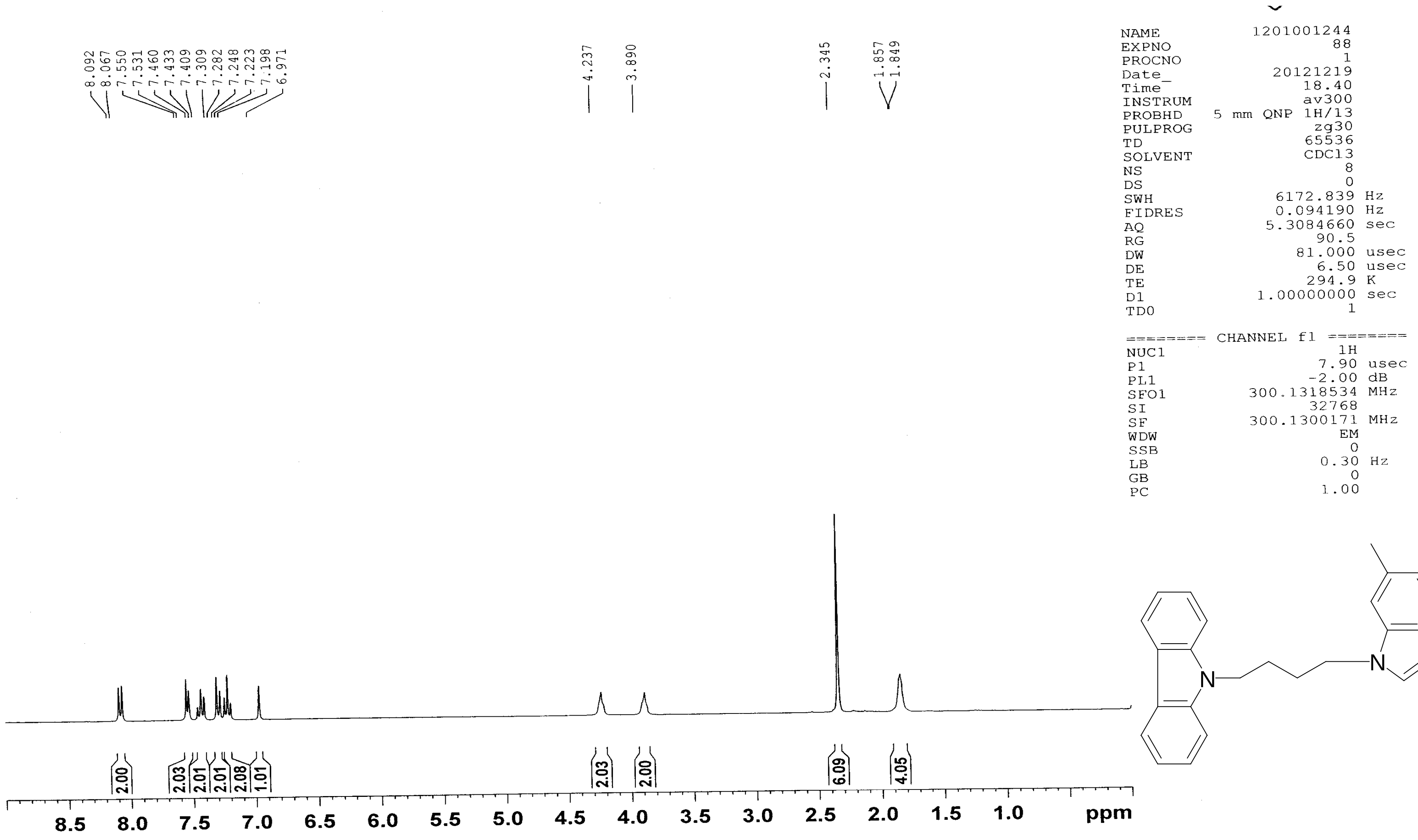

8.092
8.067
7.550
7.531
7.460
7.433
7.409
7.309
7.282
7.248
7.223
7.198
6.971
4.237
3.890
2.345
1.857
1.849
NAME 1201001244
EXPNO 88
PROCNO 1
Date_ 20121219
Time_ 18.40
INSTRUM av300
PROBHD 5 mm QNP 1H/13
PULPROG zg30
TD 65536
SOLVENT CDCl3
NS 8
DS 0
SWH 6172.839 Hz
FIDRES 0.094190 Hz
AQ 5.3084660 sec
RG 90.5
DW 81.000 usec
DE 6.50 usec
TE 294.9 K
D1 1.00000000 sec
TD0 1
======== CHANNEL f1 ========
NUC1 1H
P1 7.90 usec
PL1 -2.00 dB
SFO1 300.1318534 MHz
SI 32768
SF 300.1300171 MHz
WDW EM
SSB 0
LB 0.30 Hz
GB 0
PC 1.00
2.00
2.03
2.01
2.01
2.08
1.01
2.03
2.00
6.09
4.05
8.5
8.0
7.5
7.0
6.5
6.0
5.5
5.0
4.5
4.0
3.5
3.0
2.5
2.0
1.5
1.0
ppm
N
N
N

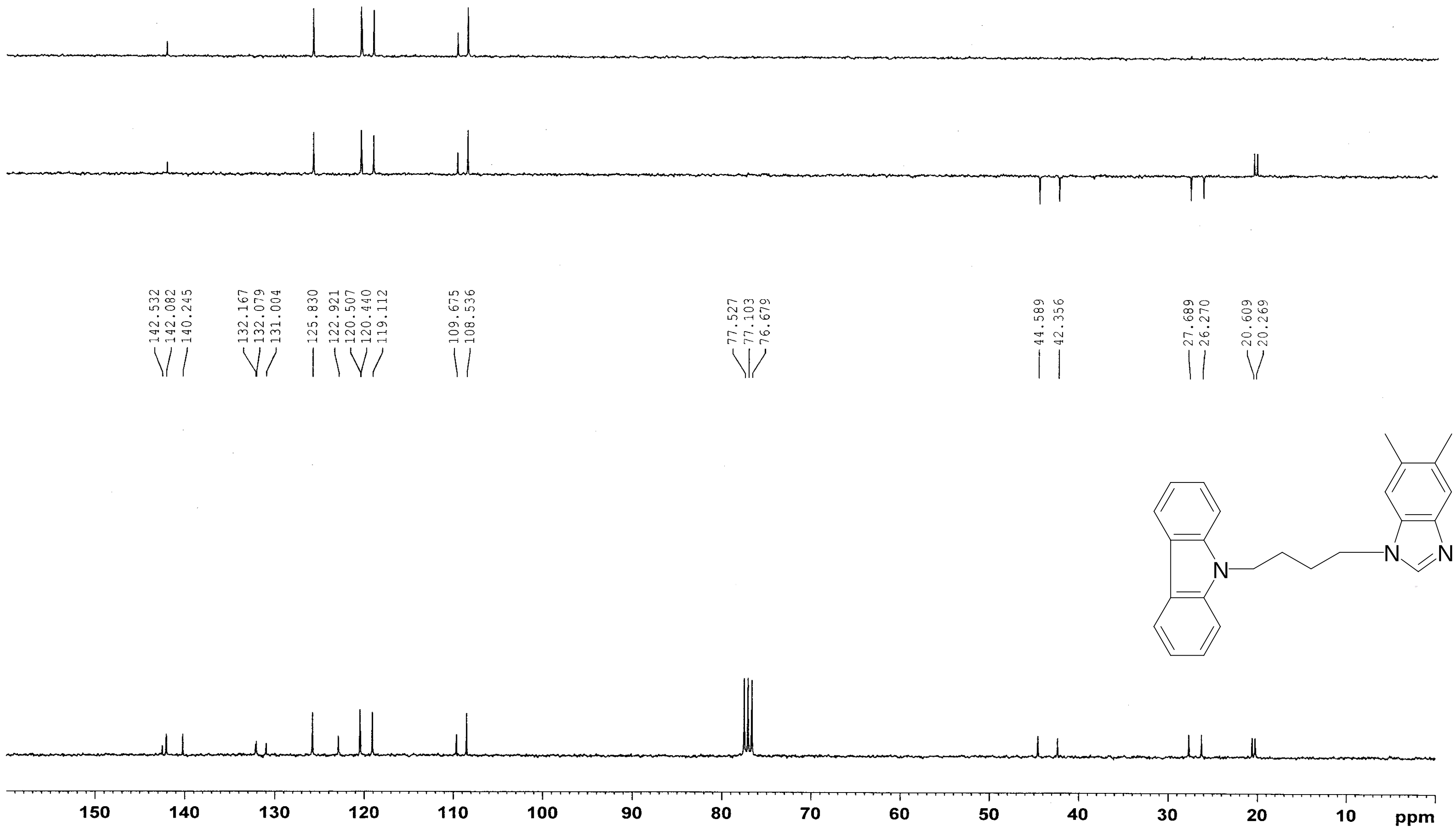

142.532
142.082
140.245
132.167
132.079
131.004
125.830
122.921
120.507
120.440
119.112
109.675
108.536
77.527
77.103
76.679
44.589
42.356
27.689
26.270
20.609
20.269
N
N
N
150
140
130
120
110
100
90
80
70
60
50
40
30
20
10
ppm

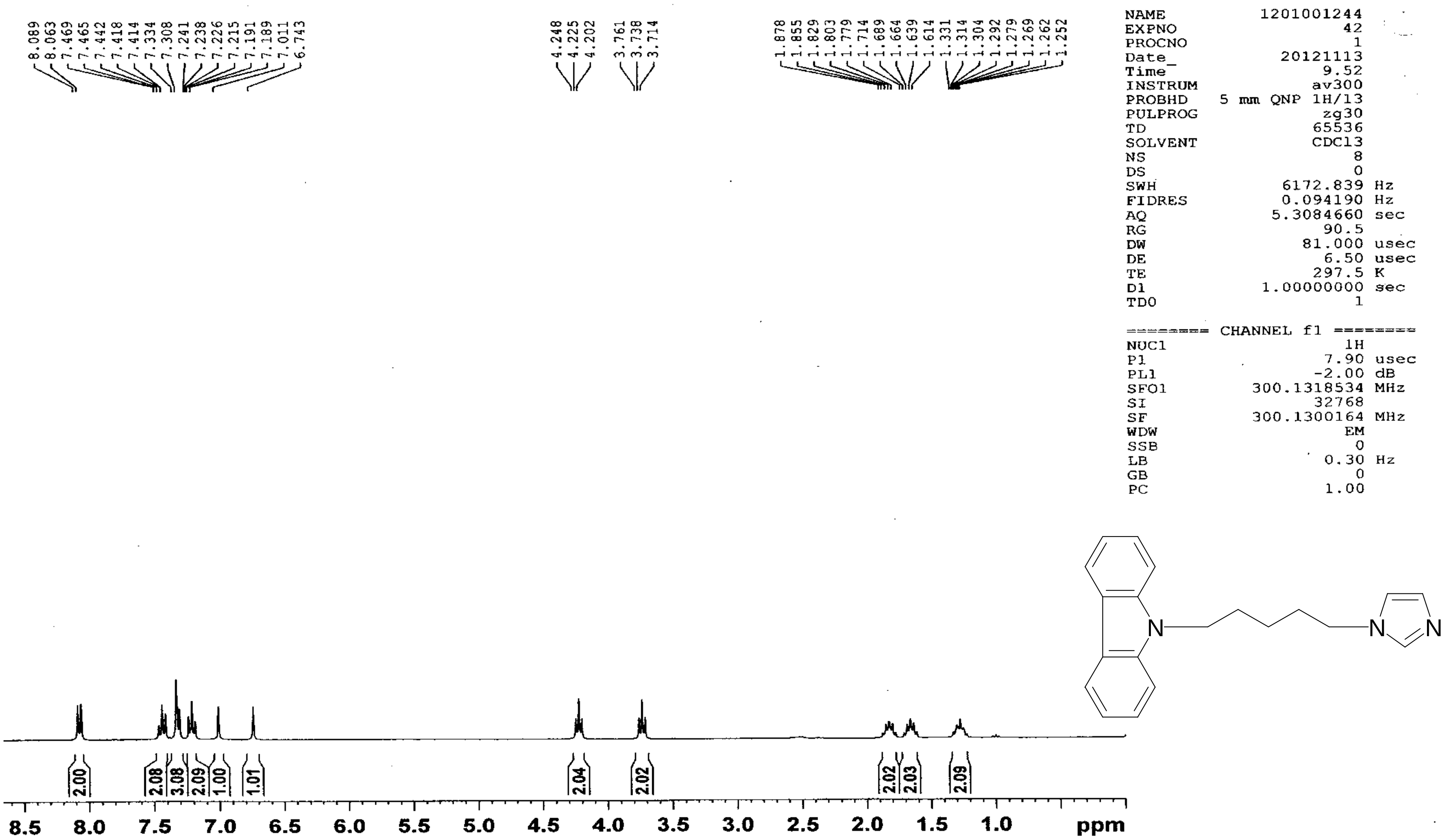
8.089 8.063 7.469 7.465 7.442 7.418 7.414 7.334 7.308 7.241 7.238 7.226 7.215 7.191 7.189 7.011 6.743
4.248 4.225 4.202
3.761 3.738 3.714
1.878 1.855 1.829 1.803 1.779 1.714 1.689 1.664 1.639 1.614 1.331 1.314 1.304 1.292 1.279 1.269 1.262 1.252
NAME 1201001244
EXPNO 42
PROCNO 1
Date_ 20121113
Time 9.52
INSTRUM av300
PROBHD 5 mm QNP 1H/13
PULPROG zg30
TD 65536
SOLVENT CDCl3
NS 8
DS 0
SWH 6172.839 Hz
FIDRES 0.094190 Hz
AQ 5.3084660 sec
RG 90.5
DW 81.000 usec
DE 6.50 usec
TE 297.5 K
D1 1.00000000 sec
TD0 1
======== CHANNEL f1 ========
NUC1 1H
P1 7.90 usec
PL1 -2.00 dB
SFO1 300.1318534 MHz
SI 32768
SF 300.1300164 MHz
WDW EM
SSB 0
LB 0.30 Hz
GB 0
PC 1.00
N
N
N
2.00 2.08 3.08 2.09 1.00 1.01 2.04 2.02 2.02 2.03 2.09
8.5 8.0 7.5 7.0 6.5 6.0 5.5 5.0 4.5 4.0 3.5 3.0 2.5 2.0 1.5 1.0 ppm

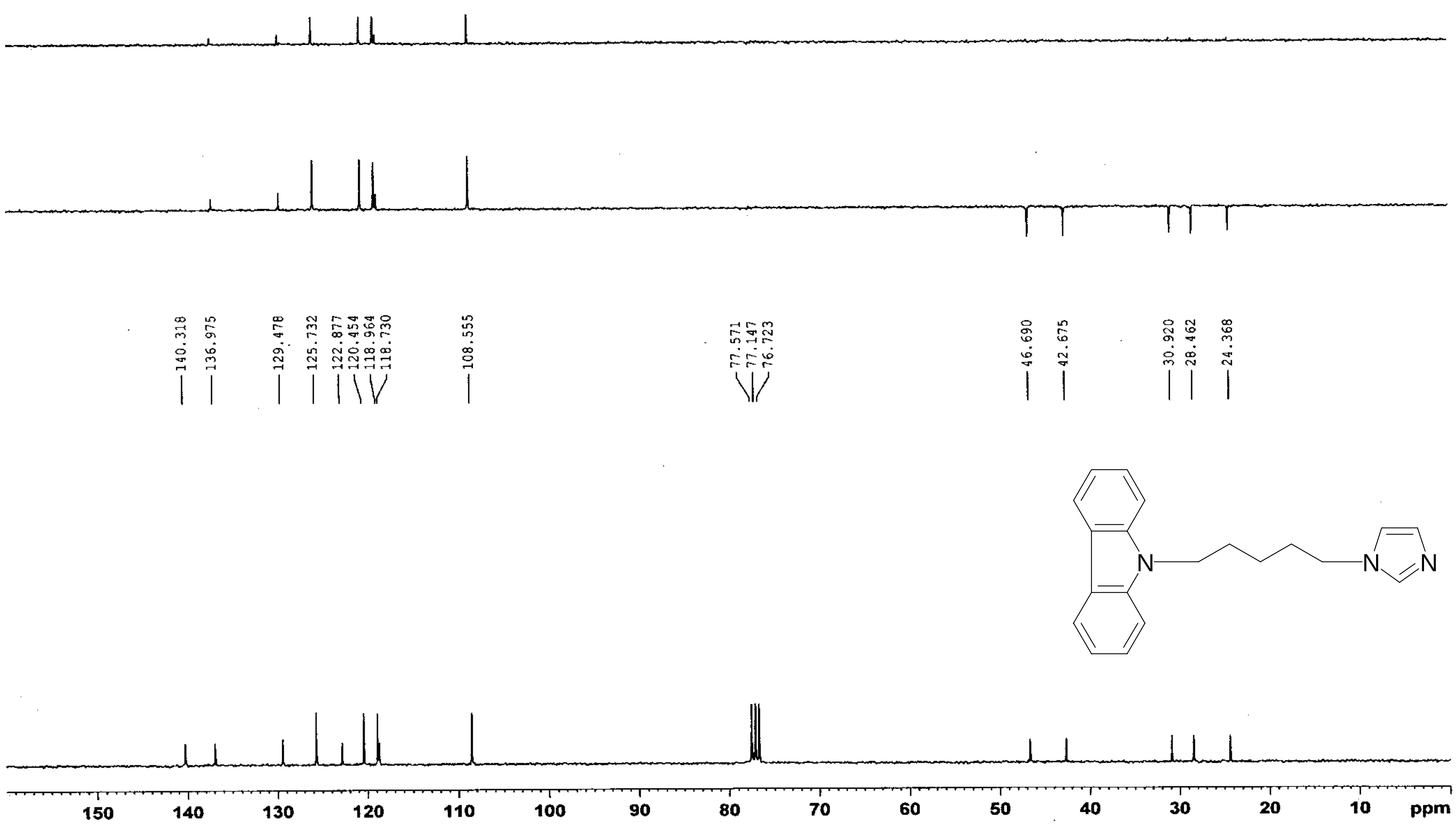
140.318
136.975
129.478
125.732
122.877
120.454
118.964
118.730
108.555
77.571
77.147
76.723
46.690
42.675
30.920
28.462
24.368
N
N
N
150
140
130
120
110
100
90
80
70
60
50
40
30
20
10
ppm

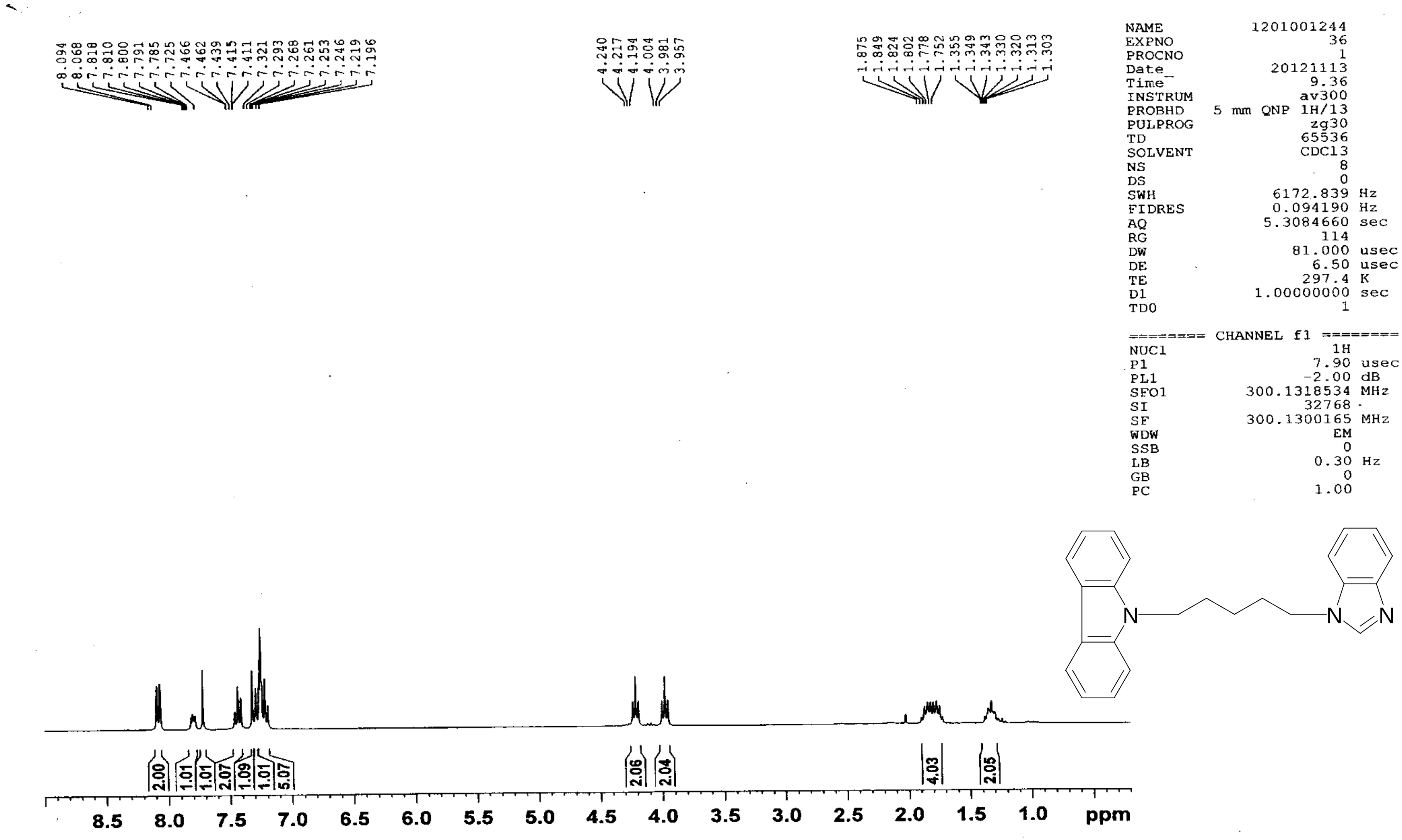
8.094
8.068
7.818
7.810
7.800
7.791
7.785
7.725
7.466
7.462
7.439
7.415
7.411
7.321
7.293
7.268
7.261
7.253
7.246
7.219
7.196
4.240
4.217
4.194
4.004
3.981
3.957
1.875
1.849
1.824
1.802
1.778
1.752
1.355
1.349
1.343
1.330
1.320
1.313
1.303
NAME 1201001244
EXPNO 36
PROCNO 1
Date_ 20121113
Time 9.36
INSTRUM av300
PROBHD 5 mm QNP 1H/13
PULPROG zg30
TD 65536
SOLVENT CDCl3
NS 8
DS 0
SWH 6172.839 Hz
FIDRES 0.094190 Hz
AQ 5.3084660 sec
RG 114
DW 81.000 usec
DE 6.50 usec
TE 297.4 K
D1 1.00000000 sec
TD0 1
======== CHANNEL f1 ========
NUC1 1H
P1 7.90 usec
PL1 -2.00 dB
SFO1 300.1318534 MHz
SI 32768
SF 300.1300165 MHz
WDW EM
SSB 0
LB 0.30 Hz
GB 0
PC 1.00
N
N
N
2.00
1.01
1.01
2.07
1.09
1.01
5.07
2.06
2.04
4.03
2.05
8.5
8.0
7.5
7.0
6.5
6.0
5.5
5.0
4.5
4.0
3.5
3.0
2.5
2.0
1.5
1.0
ppm

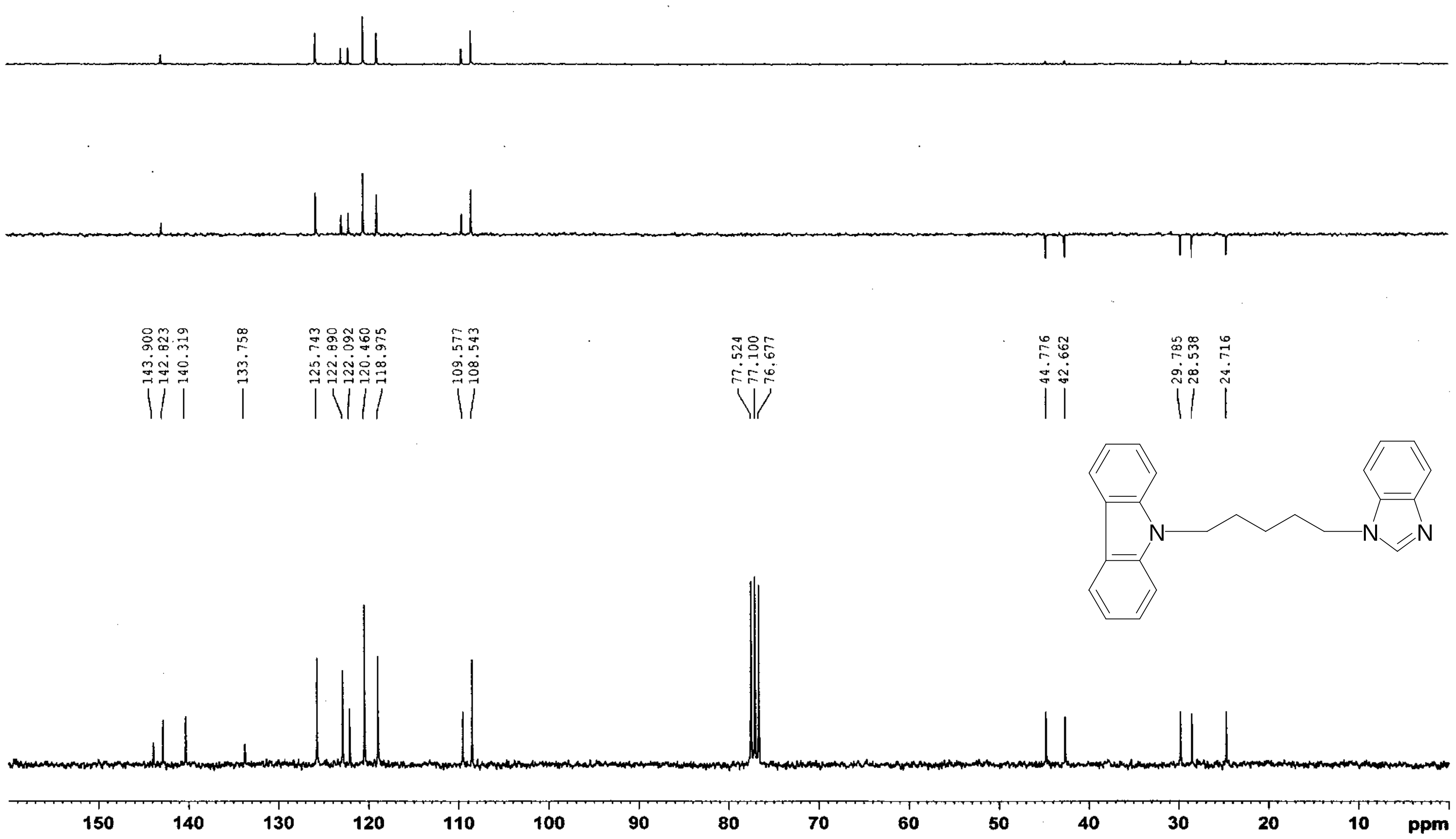

143.900
142.823
140.319
133.758
125.743
122.890
122.092
120.460
118.975
109.577
108.543
77.524
77.100
76.677
44.776
42.662
29.785
28.538
24.716
N
N
N
150
140
130
120
110
100
90
80
70
60
50
40
30
20
10
ppm

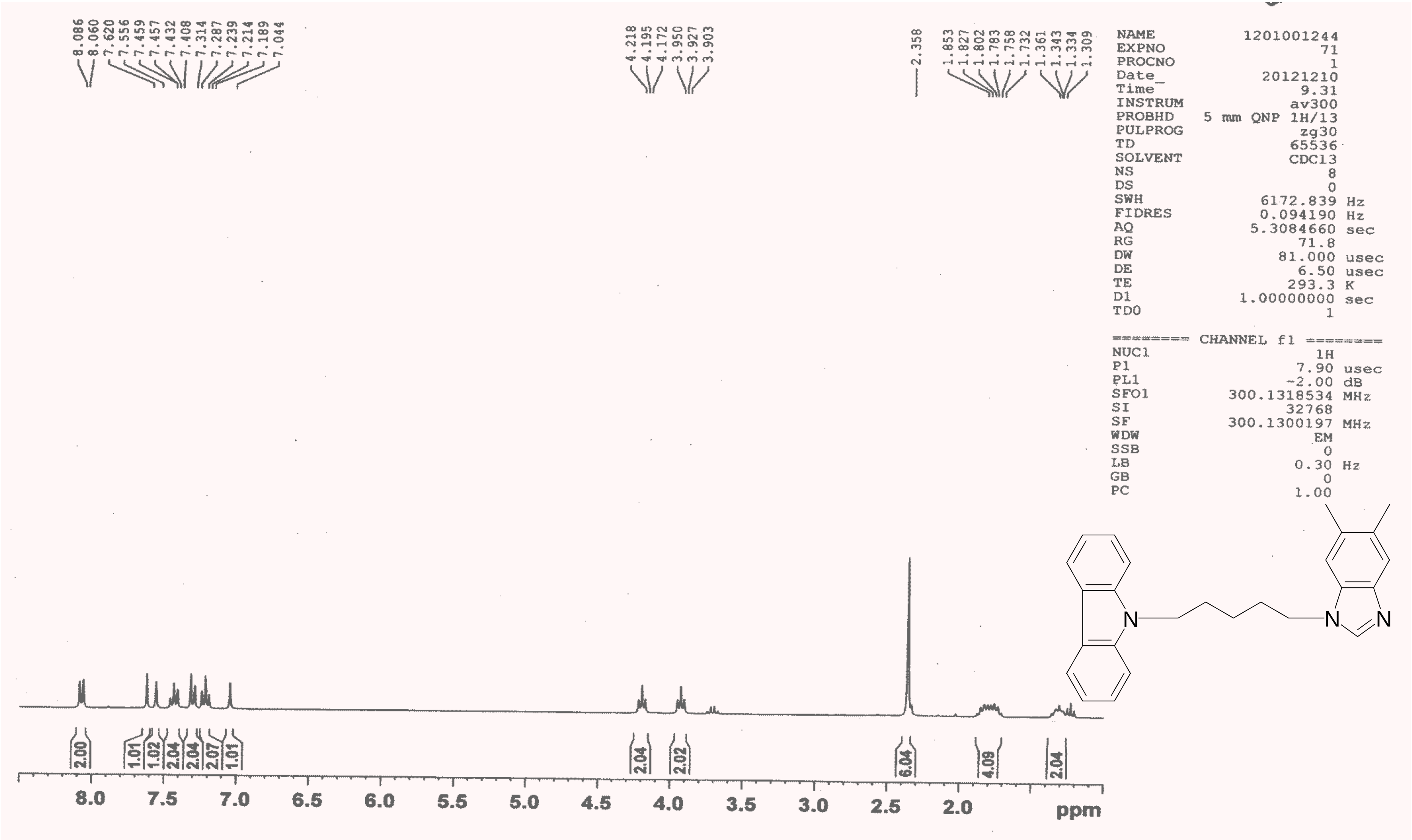
8.086
8.060
7.620
7.556
7.459
7.457
7.432
7.408
7.314
7.287
7.239
7.214
7.189
7.044
4.218
4.195
4.172
3.950
3.927
3.903
2.358
1.853
1.827
1.802
1.783
1.758
1.732
1.361
1.343
1.334
1.309
NAME 1201001244
EXPNO 71
PROCNO 1
Date_ 20121210
Time 9.31
INSTRUM av300
PROBHD 5 mm QNP 1H/13
PULPROG zg30
TD 65536
SOLVENT CDCl3
NS 8
DS 0
SWH 6172.839 Hz
FIDRES 0.094190 Hz
AQ 5.3084660 sec
RG 71.8
DW 81.000 usec
DE 6.50 usec
TE 293.3 K
D1 1.00000000 sec
TD0 1
======== CHANNEL f1 ========
NUC1 1H
P1 7.90 usec
PL1 -2.00 dB
SFO1 300.1318534 MHz
SI 32768
SF 300.1300197 MHz
WDW EM
SSB 0
LB 0.30 Hz
GB 0
PC 1.00
N
N
N
2.00
1.01
1.02
2.04
2.04
2.07
1.01
2.04
2.02
6.04
4.09
2.04
8.0
7.5
7.0
6.5
6.0
5.5
5.0
4.5
4.0
3.5
3.0
2.5
2.0
ppm

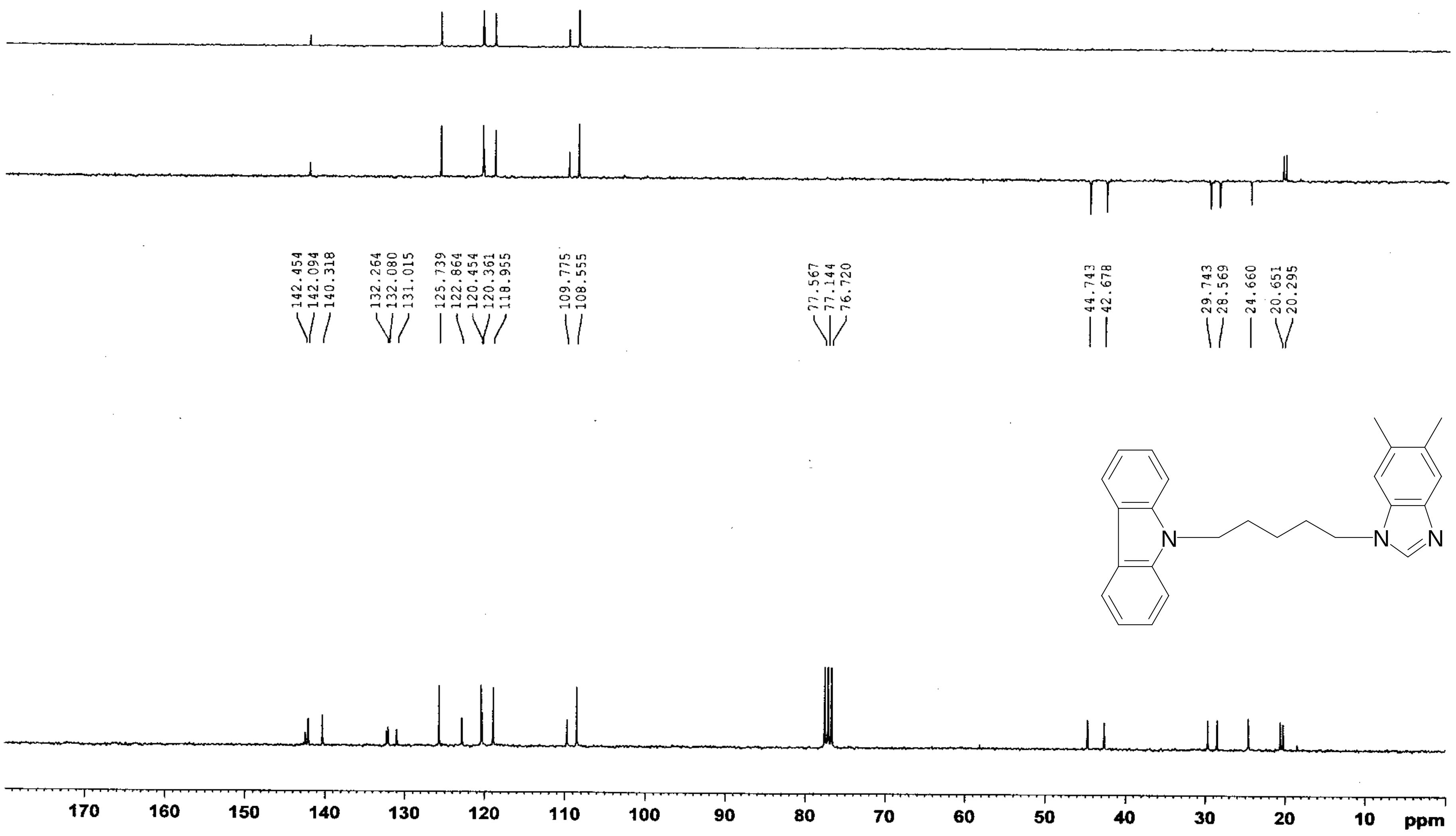

142.454
142.094
140.318
132.264
132.080
131.015
125.739
122.864
120.454
120.361
118.955
109.775
108.555
77.567
77.144
76.720
44.743
42.678
29.743
28.569
24.660
20.651
20.295
N
N
N
170 160 150 140 130 120 110 100 90 80 70 60 50 40 30 20 10 ppm

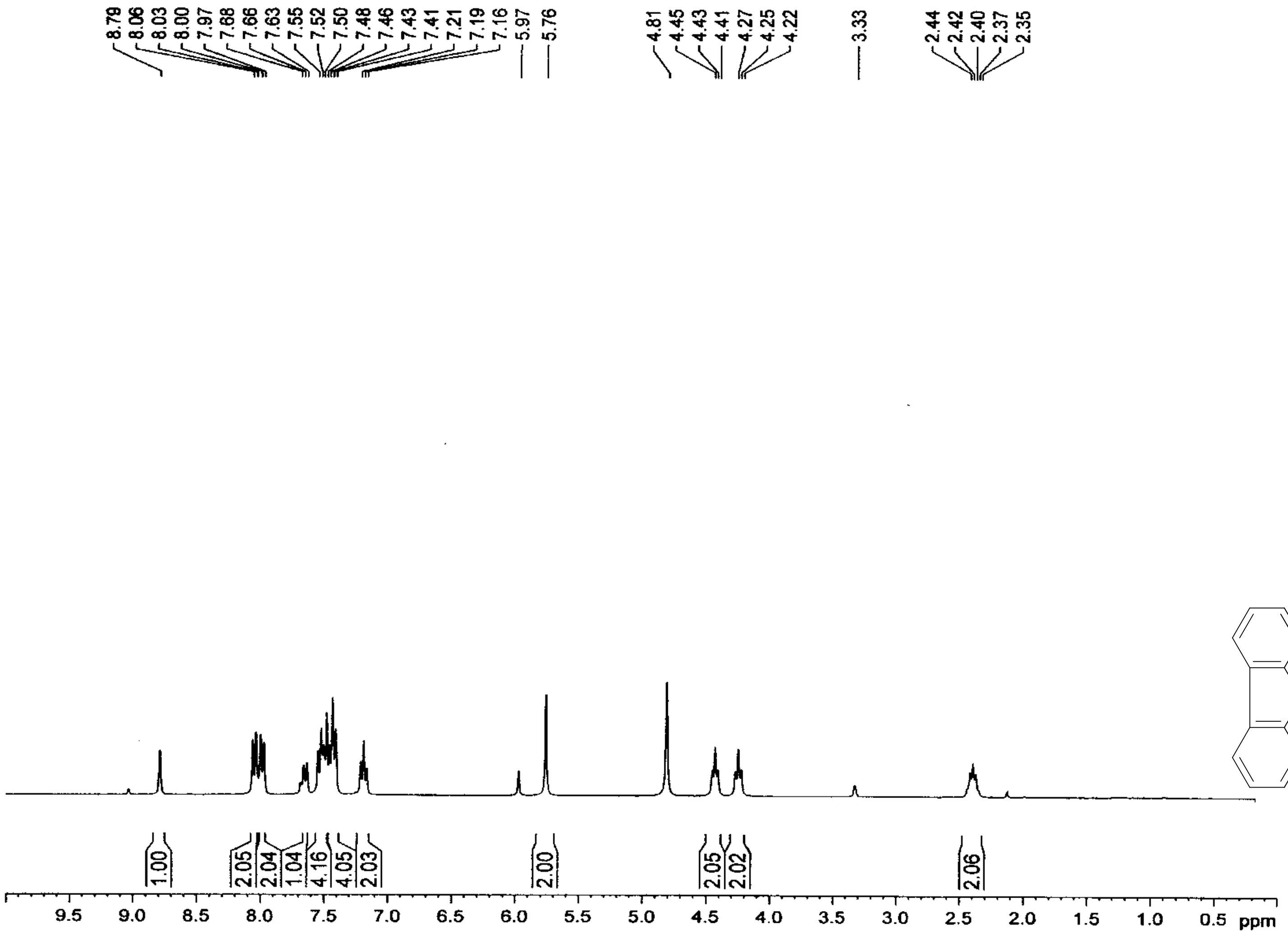

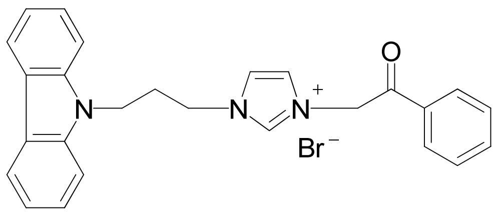

NAME	11x
EXPNO	433
PROCNO	1
Date_	20120821
Time	11.46
INSTRUM	av300
PROBHD	5 mm QNP 1H/13
PULPROG	zg30
TD	65536
SOLVENT	MeOD
NS	12
DS	2
SWH	6172.839 Hz
FIDRES	0.094190 Hz
AQ	5.3084660 sec
RG	90.5
DW	81.000 usec
DE	6.50 usec
TE	299.9 K
D1	1.00000000 sec
TD0	1

======== CHANNEL f1 ========

NUC1	1H
P1	7.90 usec
PL1	-2.00 dB
SFO1	300.1318534 MHz
SI	32768
SF	300.1300000 MHz
WDW	EM
SSB	0
LB	0.30 Hz
GB	0
PC	1.00

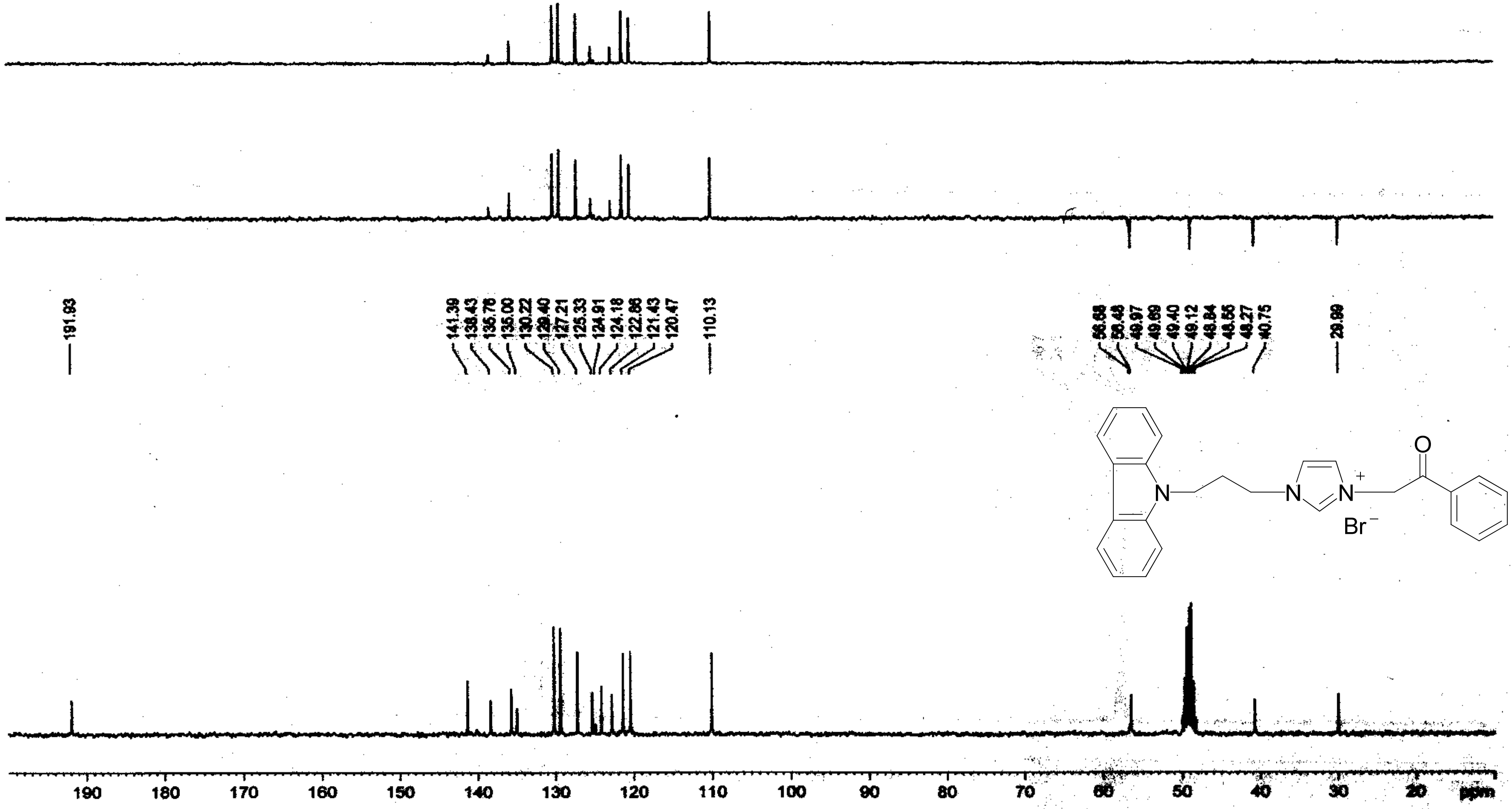

191.93
141.39
138.43
135.76
135.00
130.22
129.40
127.21
125.33
124.91
124.18
122.86
121.43
120.47
110.13
56.68
56.48
49.97
49.69
49.40
49.12
48.84
48.55
48.27
40.75
29.99
N
N
N
O
Br⁻
190 180 170 160 150 140 130 120 110 100 90 80 70 60 50 40 30 20 ppm

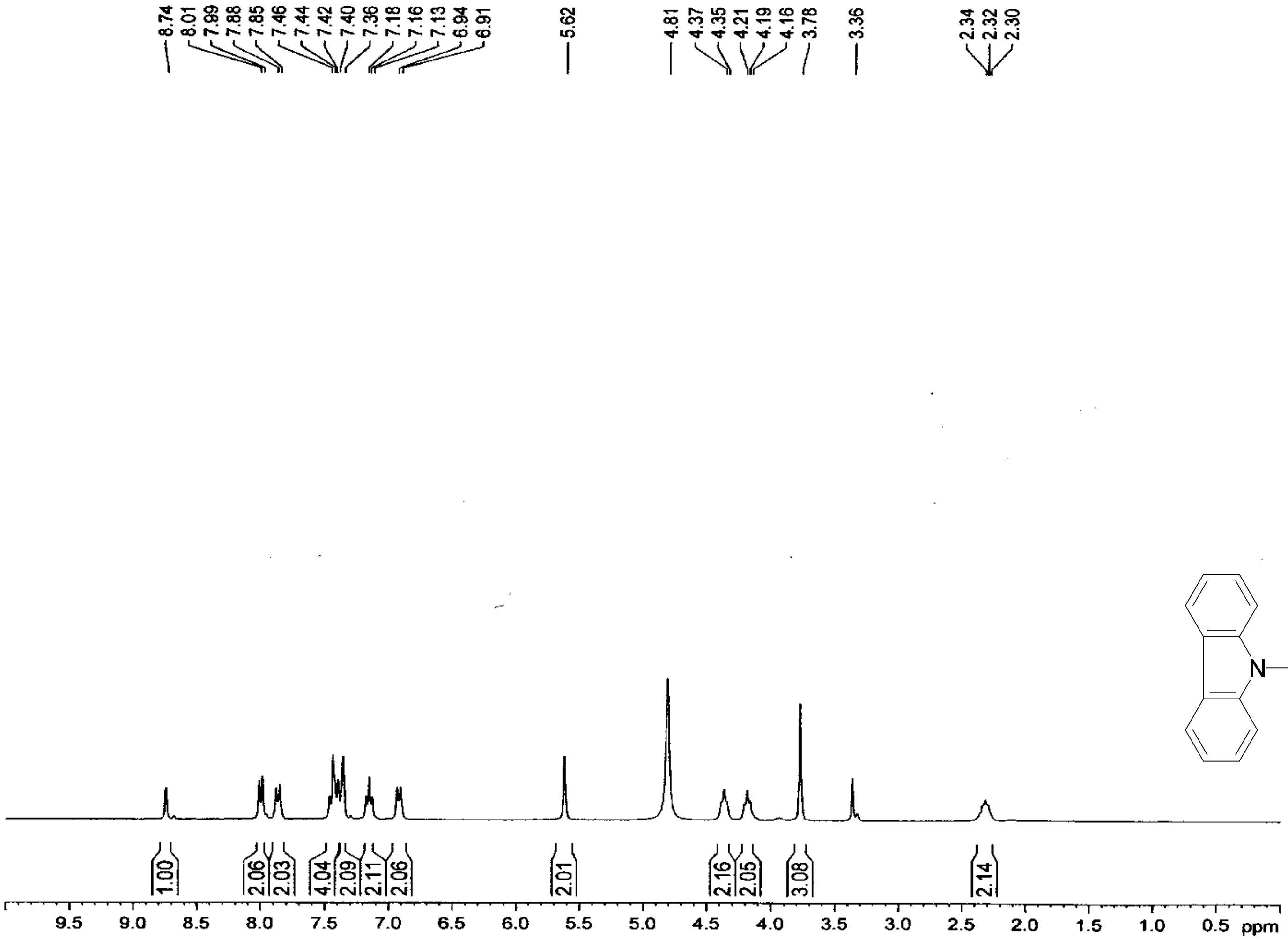

NAME	11x	
EXPNO	472	
PROCNO	1	
Date_	20120925	
Time	16.39	
INSTRUM	av300	
PROBHD	5 mm QNP 1H/13	
PULPROG	zg30	
TD	65536	
SOLVENT	MeOD	
NS	8	
DS	0	
SWH	6172.839	Hz
FIDRES	0.094190	Hz
AQ	5.3084660	sec
RG	45.3	
DW	81.000	usec
DE	6.50	usec
TE	299.0	K
D1	1.00000000	sec
TD0	1	
======== CHANNEL f1 ========		
NUC1	1H	
P1	7.90	usec
PL1	-2.00	dB
SFO1	300.1318534	MHz
SI	32768	
SF	300.1300000	MHz
WDW	EM	
SSB	0	
LB	0.30	Hz
GB	0	
PC	1.00	

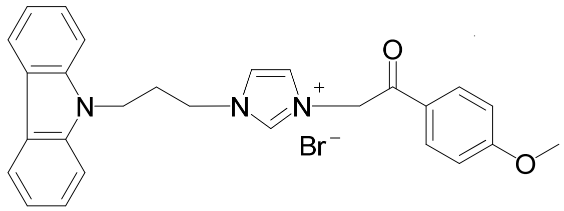

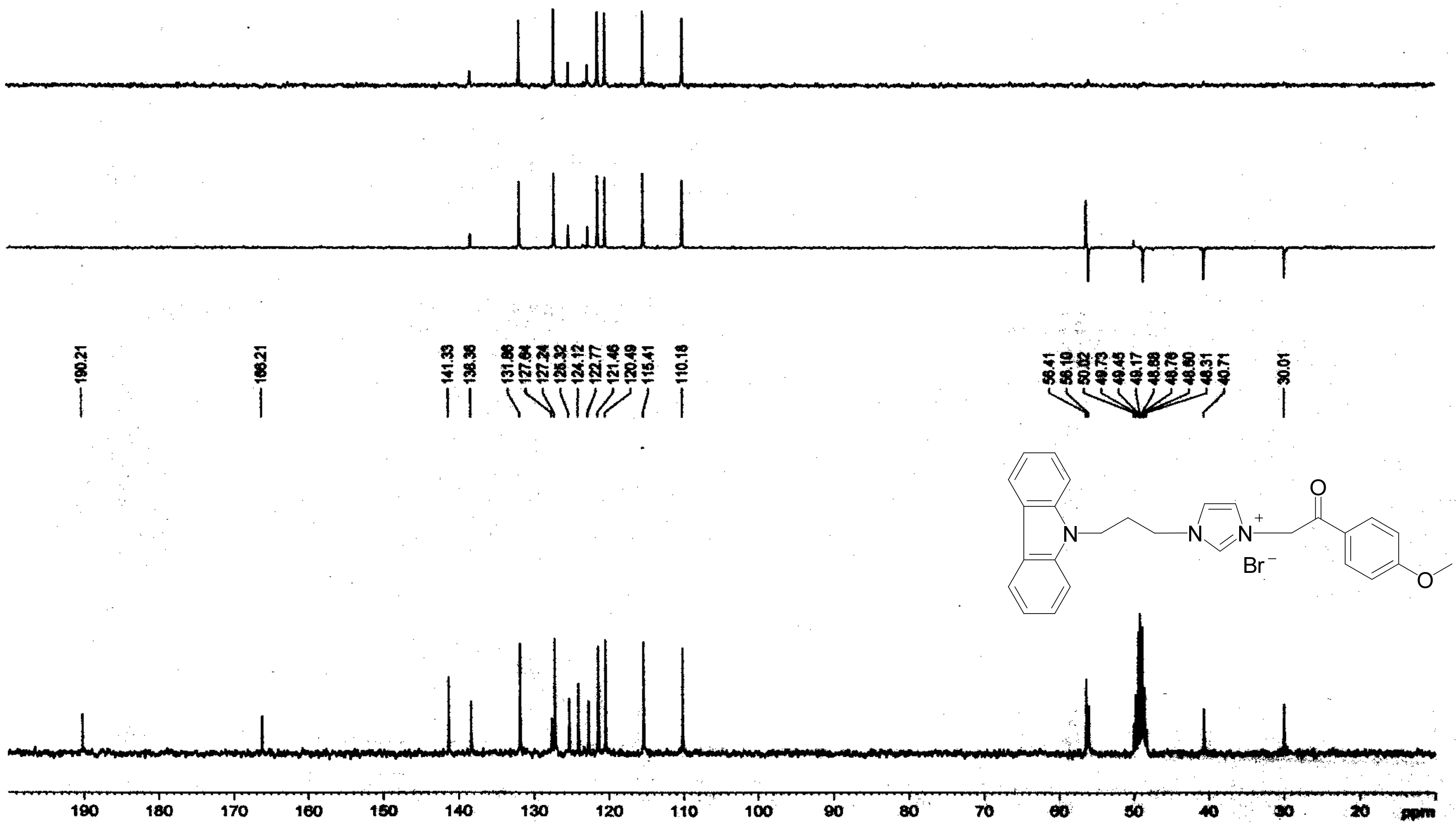

190.21
166.21
141.33
138.36
131.86
127.64
127.24
125.32
124.12
122.77
121.46
120.49
115.41
110.18
56.41
56.10
50.02
49.73
49.45
49.17
48.88
48.76
48.60
48.31
40.71
30.01
N
N
N
+
O
O
Br⁻
190
180
170
160
150
140
130
120
110
100
90
80
70
60
50
40
30
20
ppm

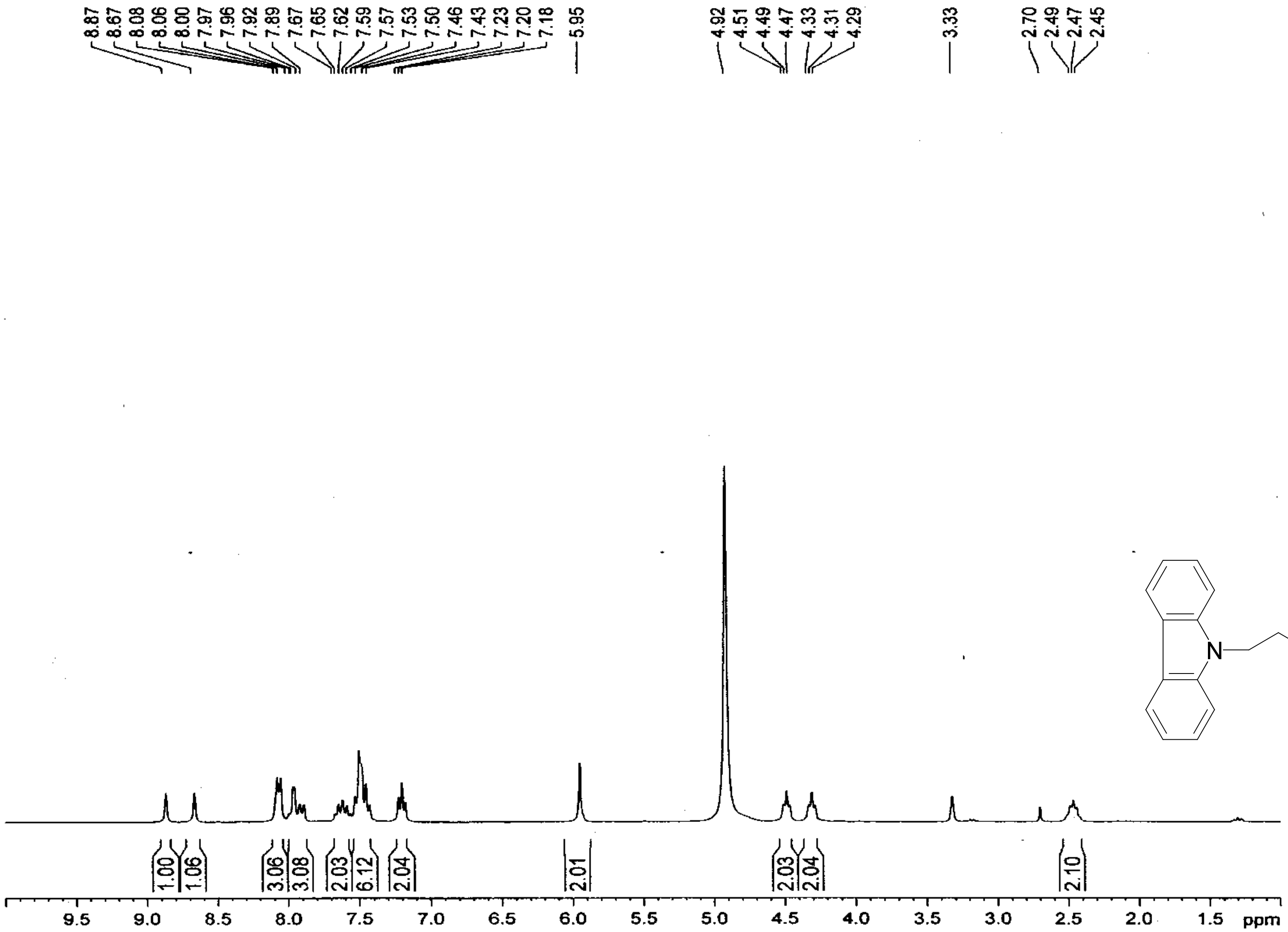

NAME	11x	
EXPNO	476	
PROCNO	1	
Date_	20120928	
Time	14.42	
INSTRUM	av300	
PROBHD	5 mm QNP 1H/13	
PULPROG	zg30	
TD	65536	
SOLVENT	MeOD	
NS	8	
DS	0	
SWH	6172.839	Hz
FIDRES	0.094190	Hz
AQ	5.3084660	sec
RG	128	
DW	81.000	usec
DE	6.50	usec
TE	296.5	K
D1	1.00000000	sec
TD0	1	

======== CHANNEL f1 ========

NUC1	1H	
P1	7.90	usec
PL1	-2.00	dB
SFO1	300.1318534	MHz
SI	32768	
SF	300.1300000	MHz
WDW	EM	
SSB	0	
LB	0.30	Hz
GB	0	
PC	1.00	

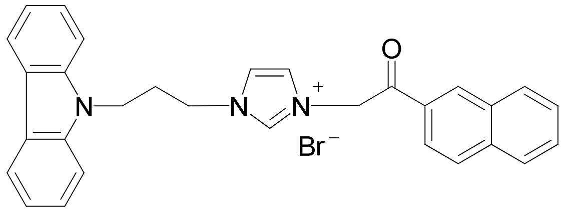

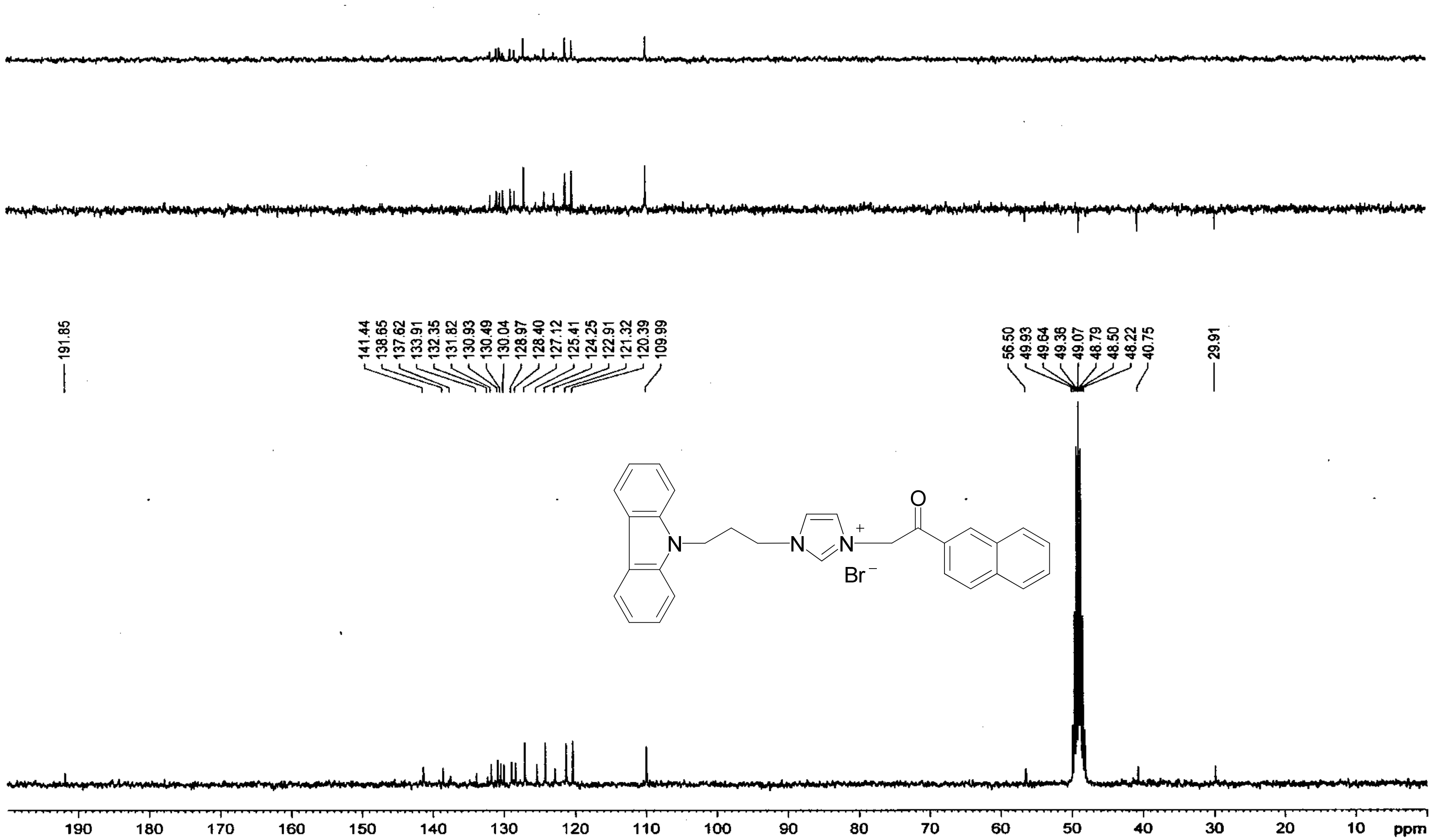

191.85
141.44
138.65
137.62
133.91
132.35
131.82
130.93
130.49
130.04
128.97
128.40
127.12
125.41
124.25
122.91
121.32
120.39
109.99
56.50
49.93
49.64
49.36
49.07
48.79
48.50
48.22
40.75
29.91
N
N
N
+
O
Br −
190 180 170 160 150 140 130 120 110 100 90 80 70 60 50 40 30 20 10 ppm

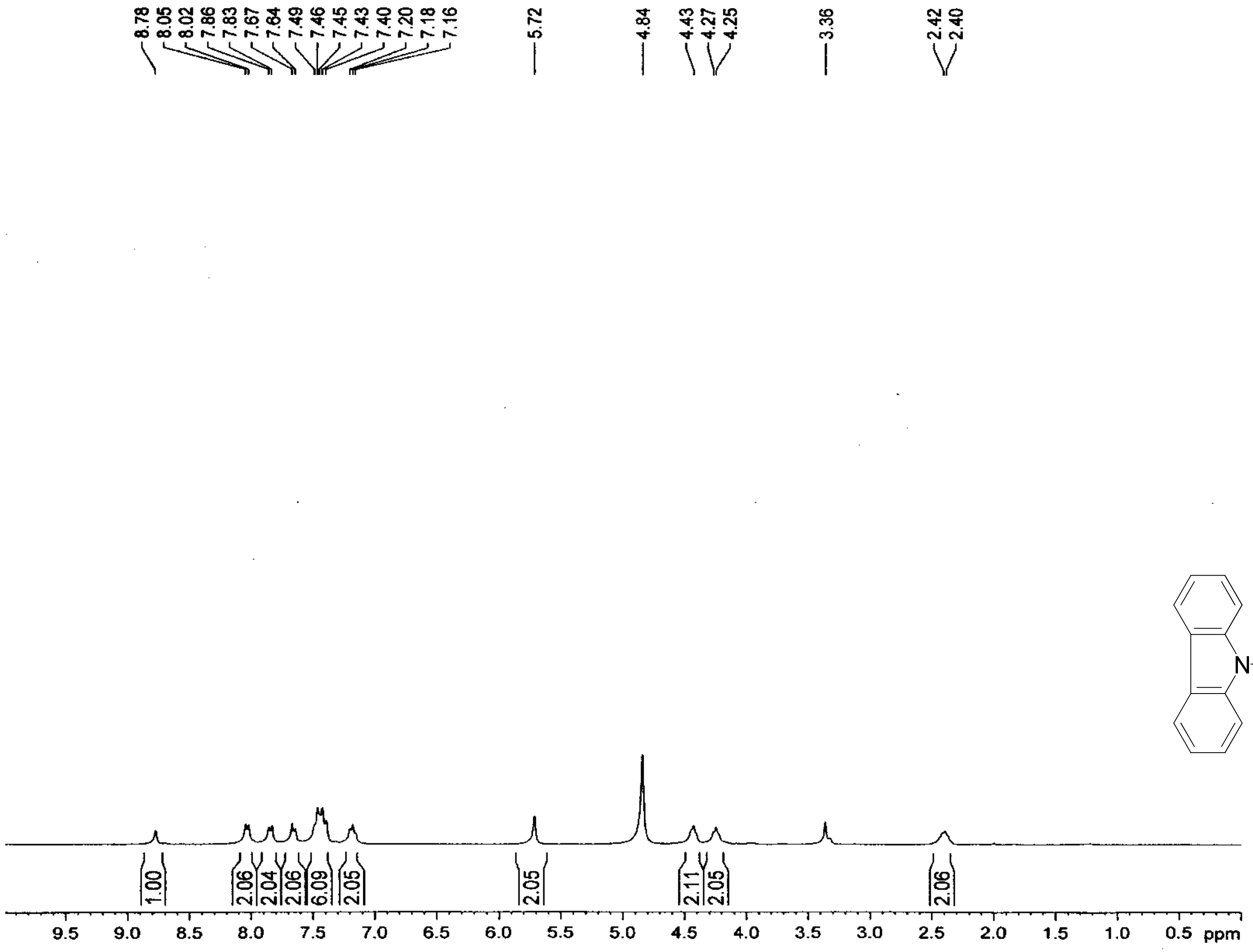

NAME	11x
EXPNO	464
PROCNO	1
Date_	20120918
Time	15.47
INSTRUM	av300
PROBHD	5 mm QNP 1H/13
PULPROG	zg30
TD	65536
SOLVENT	MeOD
NS	8
DS	0
SWH	6172.839 Hz
FIDRES	0.094190 Hz
AQ	5.3084660 sec
RG	71.8
DW	81.000 usec
DE	6.50 usec
TE	300.0 K
D1	1.00000000 sec
TD0	1
======== CHANNEL f1 ========	
NUC1	1H
P1	7.90 usec
PL1	-2.00 dB
SFO1	300.1318534 MHz
SI	32768
SF	300.1300000 MHz
WDW	EM
SSB	0
LB	0.30 Hz
GB	0
PC	1.00

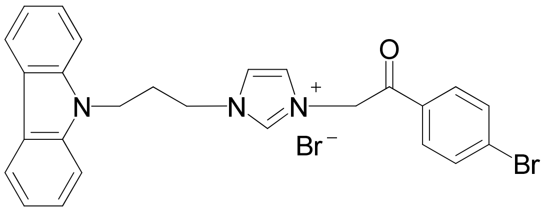

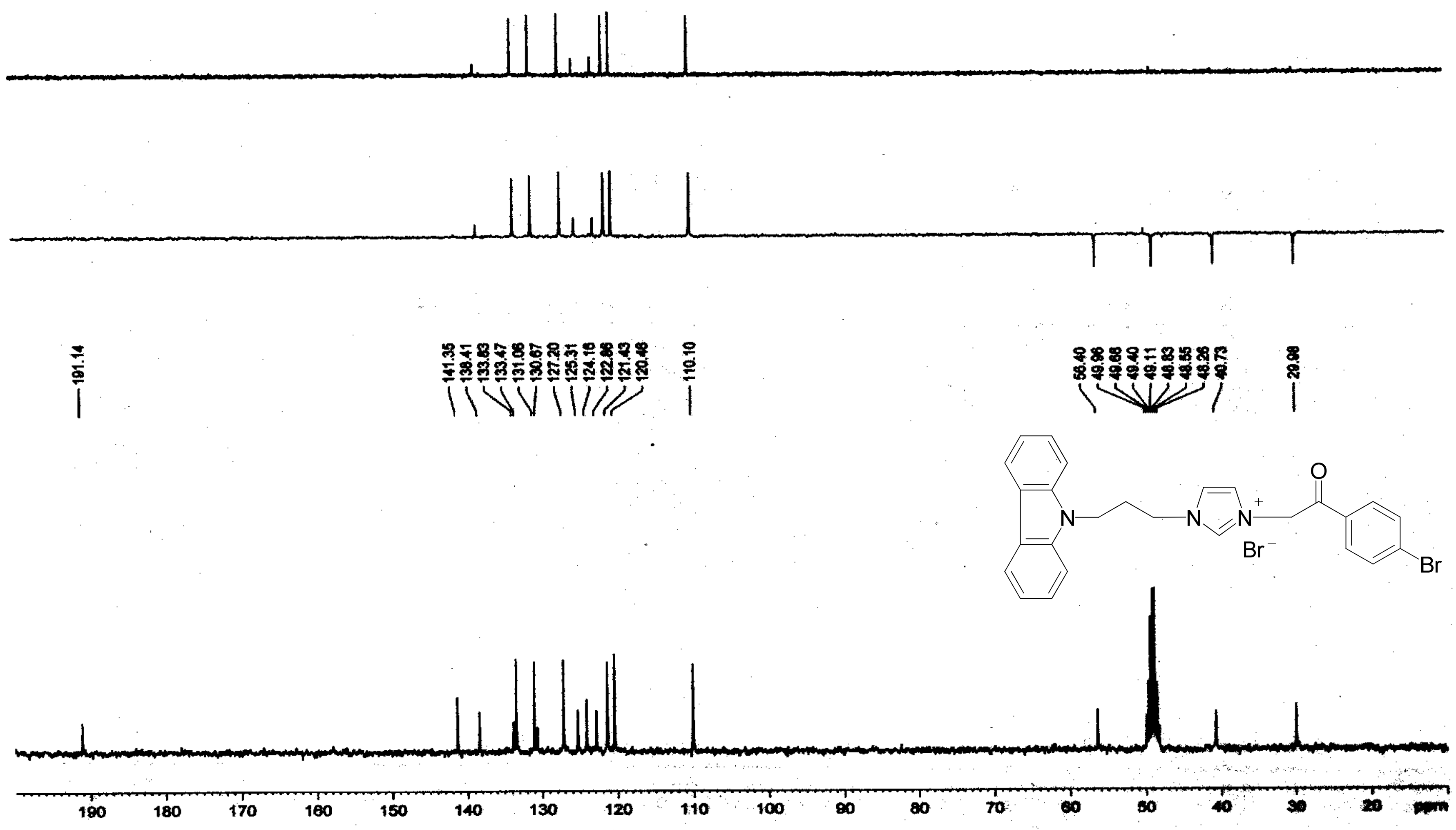

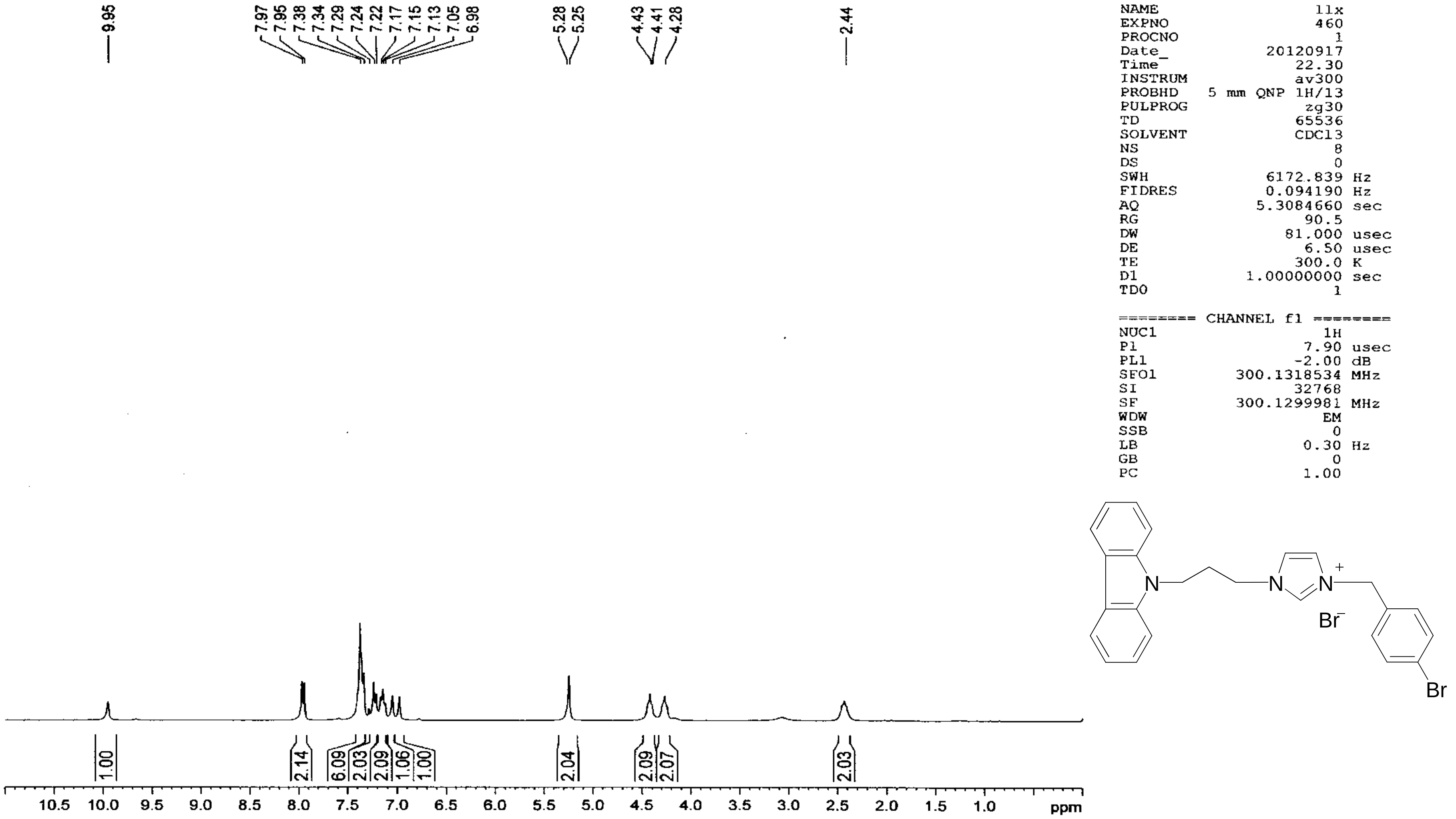
9.95
7.97
7.95
7.38
7.34
7.29
7.24
7.22
7.17
7.15
7.13
7.05
6.98
5.28
5.25
4.43
4.41
4.28
2.44
1.00
2.14
6.09
2.03
2.09
1.06
1.00
2.04
2.09
2.07
2.03
10.5 10.0 9.5 9.0 8.5 8.0 7.5 7.0 6.5 6.0 5.5 5.0 4.5 4.0 3.5 3.0 2.5 2.0 1.5 1.0 ppm
NAME 11x
EXPNO 460
PROCNO 1
Date_ 20120917
Time 22.30
INSTRUM av300
PROBHD 5 mm QNP 1H/13
PULPROG zg30
TD 65536
SOLVENT CDCl3
NS 8
DS 0
SWH 6172.839 Hz
FIDRES 0.094190 Hz
AQ 5.3084660 sec
RG 90.5
DW 81.000 usec
DE 6.50 usec
TE 300.0 K
D1 1.00000000 sec
TD0 1
======== CHANNEL f1 ========
NUC1 1H
P1 7.90 usec
PL1 -2.00 dB
SFO1 300.1318534 MHz
SI 32768
SF 300.1299981 MHz
WDW EM
SSB 0
LB 0.30 Hz
GB 0
PC 1.00
N
N
N
+
Br⁻
Br

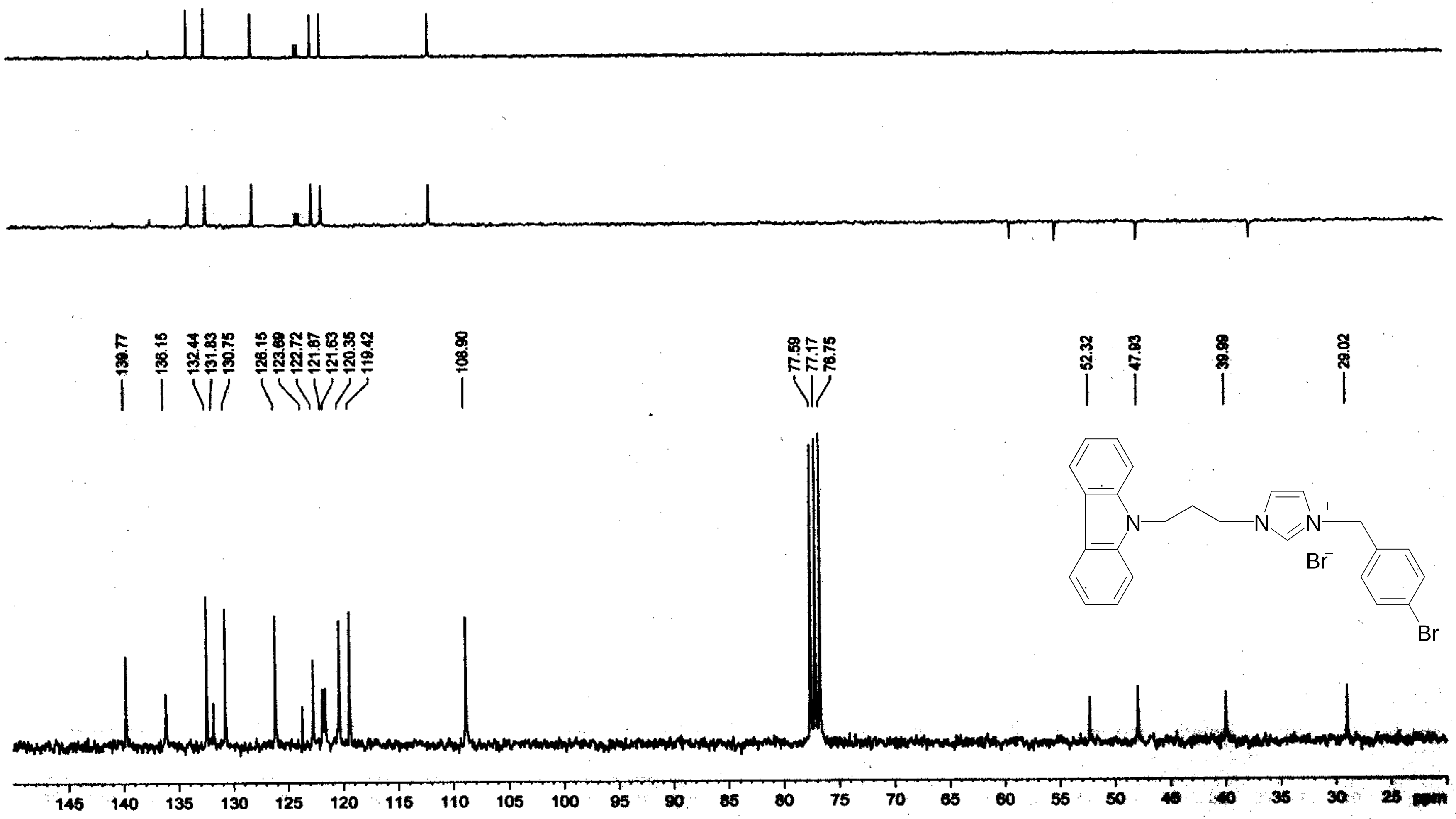

139.77
136.15
132.44
131.83
130.75
126.15
123.69
122.72
121.87
121.63
120.35
119.42
108.90
77.59
77.17
76.75
52.32
47.93
39.99
29.02
N
N
N
+
Br⁻
Br
ppm

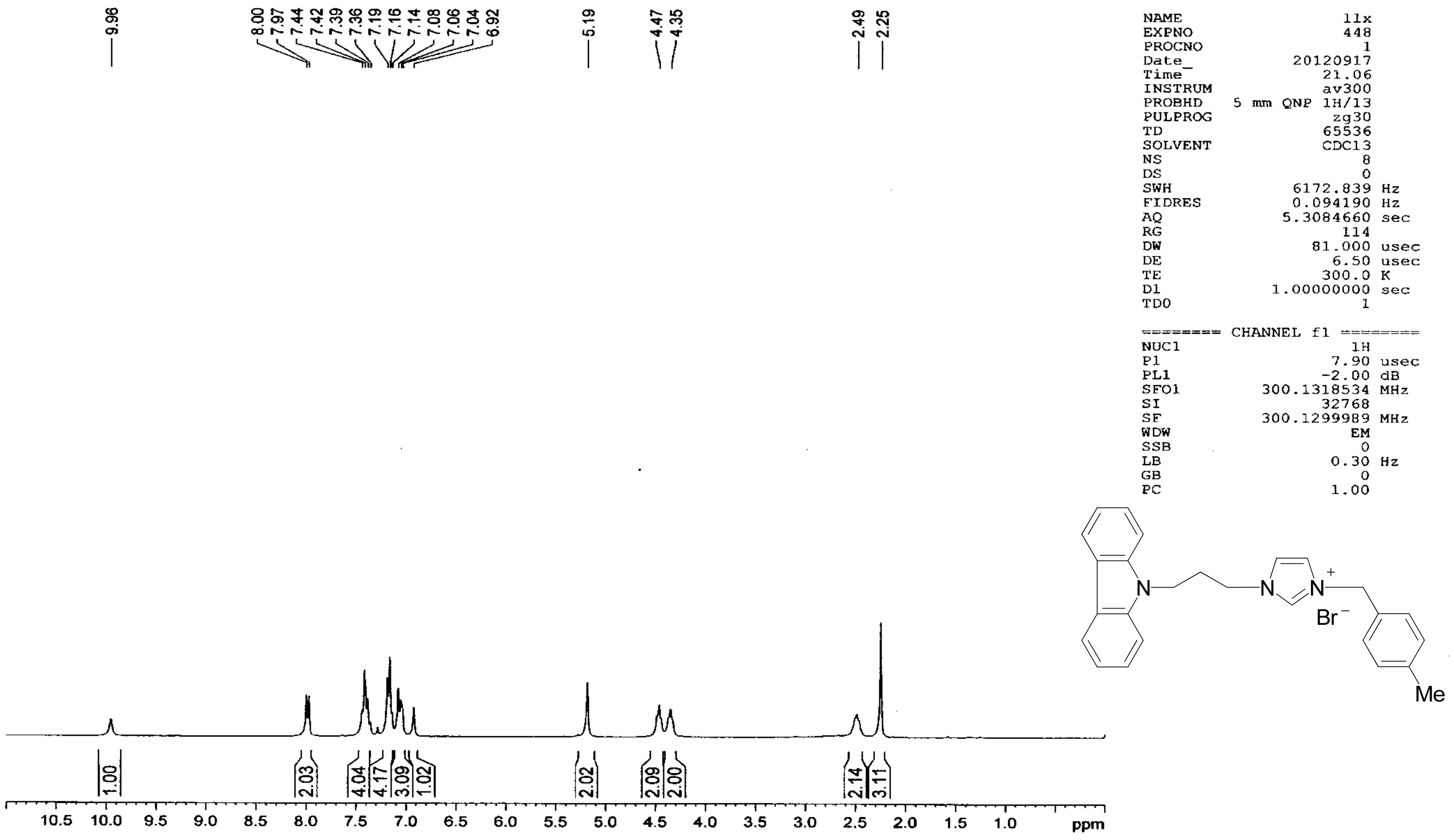

9.96
8.00 7.97 7.44 7.42 7.39 7.36 7.19 7.16 7.14 7.08 7.06 7.04 6.92
5.19
4.47 4.35
2.49 2.25
1.00 2.03 4.04 4.17 3.09 1.02 2.02 2.09 2.00 2.14 3.11
10.5 10.0 9.5 9.0 8.5 8.0 7.5 7.0 6.5 6.0 5.5 5.0 4.5 4.0 3.5 3.0 2.5 2.0 1.5 1.0 ppm
NAME 11x
EXPNO 448
PROCNO 1
Date_ 20120917
Time 21.06
INSTRUM av300
PROBHD 5 mm QNP 1H/13
PULPROG zg30
TD 65536
SOLVENT CDCl3
NS 8
DS 0
SWH 6172.839 Hz
FIDRES 0.094190 Hz
AQ 5.3084660 sec
RG 114
DW 81.000 usec
DE 6.50 usec
TE 300.0 K
D1 1.00000000 sec
TD0 1
======== CHANNEL f1 ========
NUC1 1H
P1 7.90 usec
PL1 -2.00 dB
SFO1 300.1318534 MHz
SI 32768
SF 300.1299989 MHz
WDW EM
SSB 0
LB 0.30 Hz
GB 0
PC 1.00
N
N
N+
Br−
Me

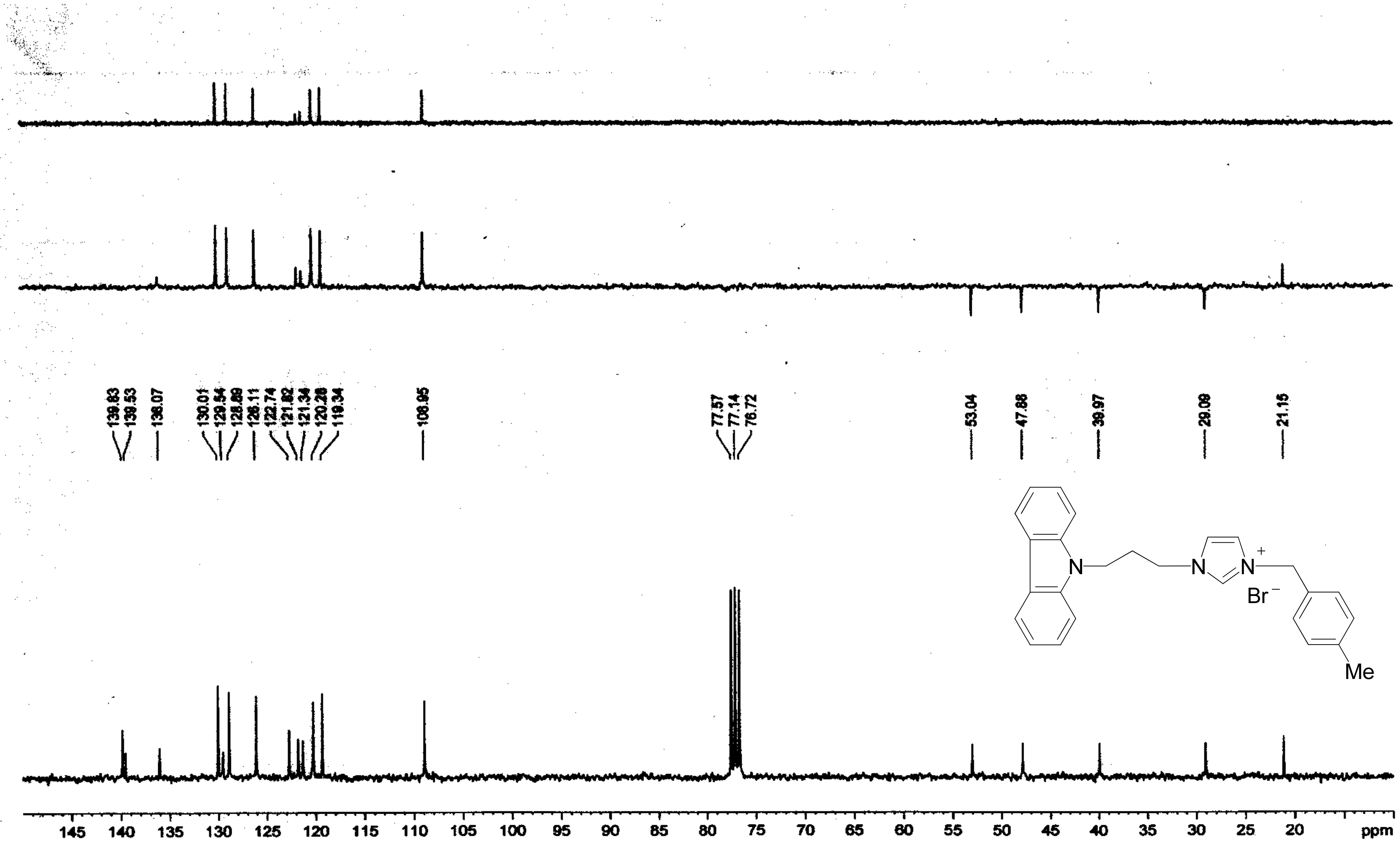
139.83
139.53
136.07
130.01
129.54
128.89
126.11
122.74
121.82
121.34
120.28
119.34
108.95
77.57
77.14
76.72
53.04
47.88
39.97
29.09
21.15
N
N
N
+
Br⁻
Me
145 140 135 130 125 120 115 110 105 100 95 90 85 80 75 70 65 60 55 50 45 40 35 30 25 20 ppm

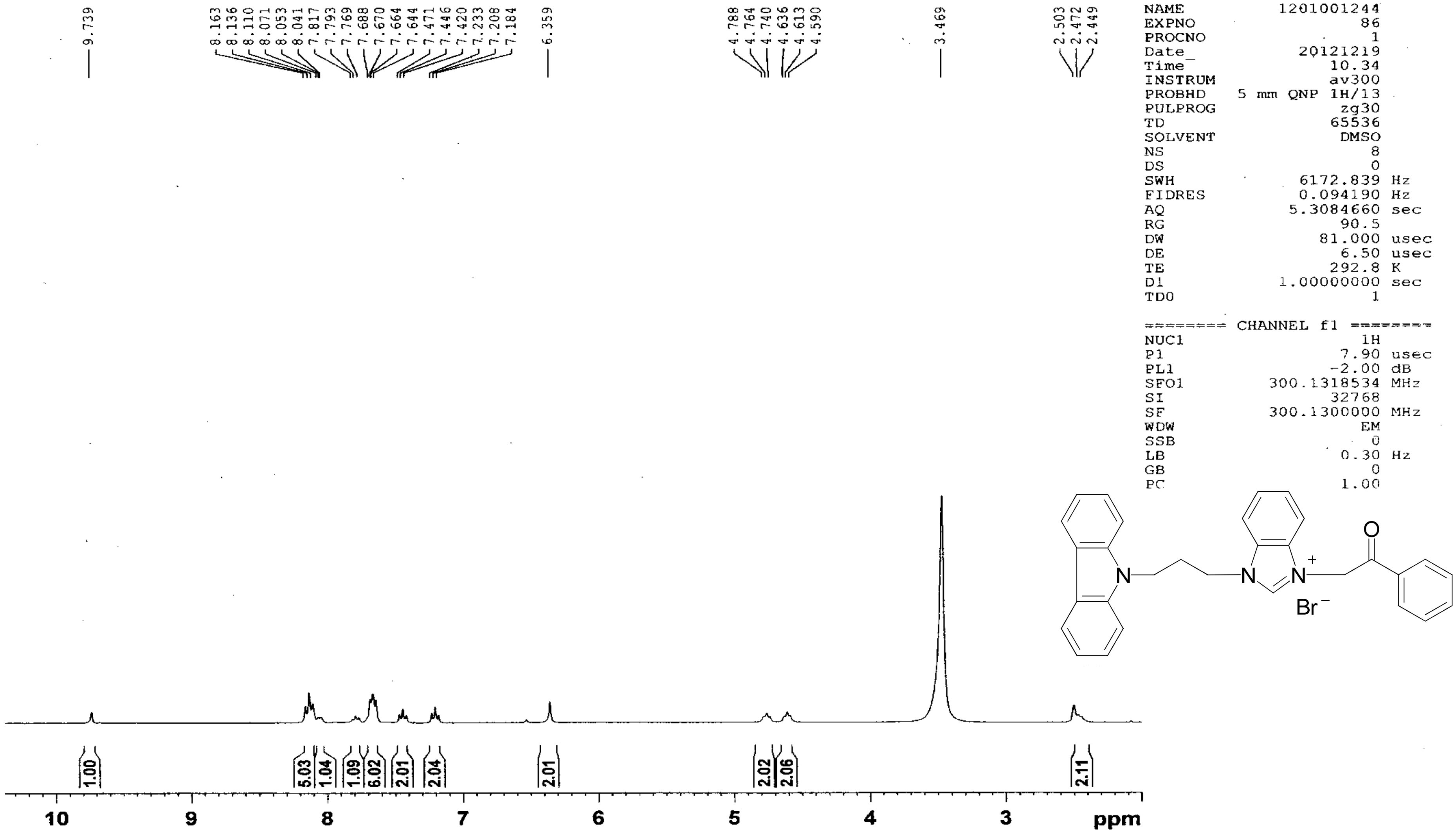
9.739
8.163 8.136 8.110 8.071 8.053 8.041 7.817 7.793 7.769 7.688 7.670 7.664 7.644 7.471 7.446 7.420 7.233 7.208 7.184
6.359
4.788 4.764 4.740 4.636 4.613 4.590
3.469
2.503 2.472 2.449
NAME 1201001244
EXPNO 86
PROCNO 1
Date_ 20121219
Time 10.34
INSTRUM av300
PROBHD 5 mm QNP 1H/13
PULPROG zg30
TD 65536
SOLVENT DMSO
NS 8
DS 0
SWH 6172.839 Hz
FIDRES 0.094190 Hz
AQ 5.3084660 sec
RG 90.5
DW 81.000 usec
DE 6.50 usec
TE 292.8 K
D1 1.00000000 sec
TD0 1
======== CHANNEL f1 ========
NUC1 1H
P1 7.90 usec
PL1 -2.00 dB
SFO1 300.1318534 MHz
SI 32768
SF 300.1300000 MHz
WDW EM
SSB 0
LB 0.30 Hz
GB 0
PC 1.00
N
N
N
O
Br⁻
1.00 5.03 1.04 1.09 6.02 2.01 2.04 2.01 2.02 2.06 2.11
10 9 8 7 6 5 4 3 ppm

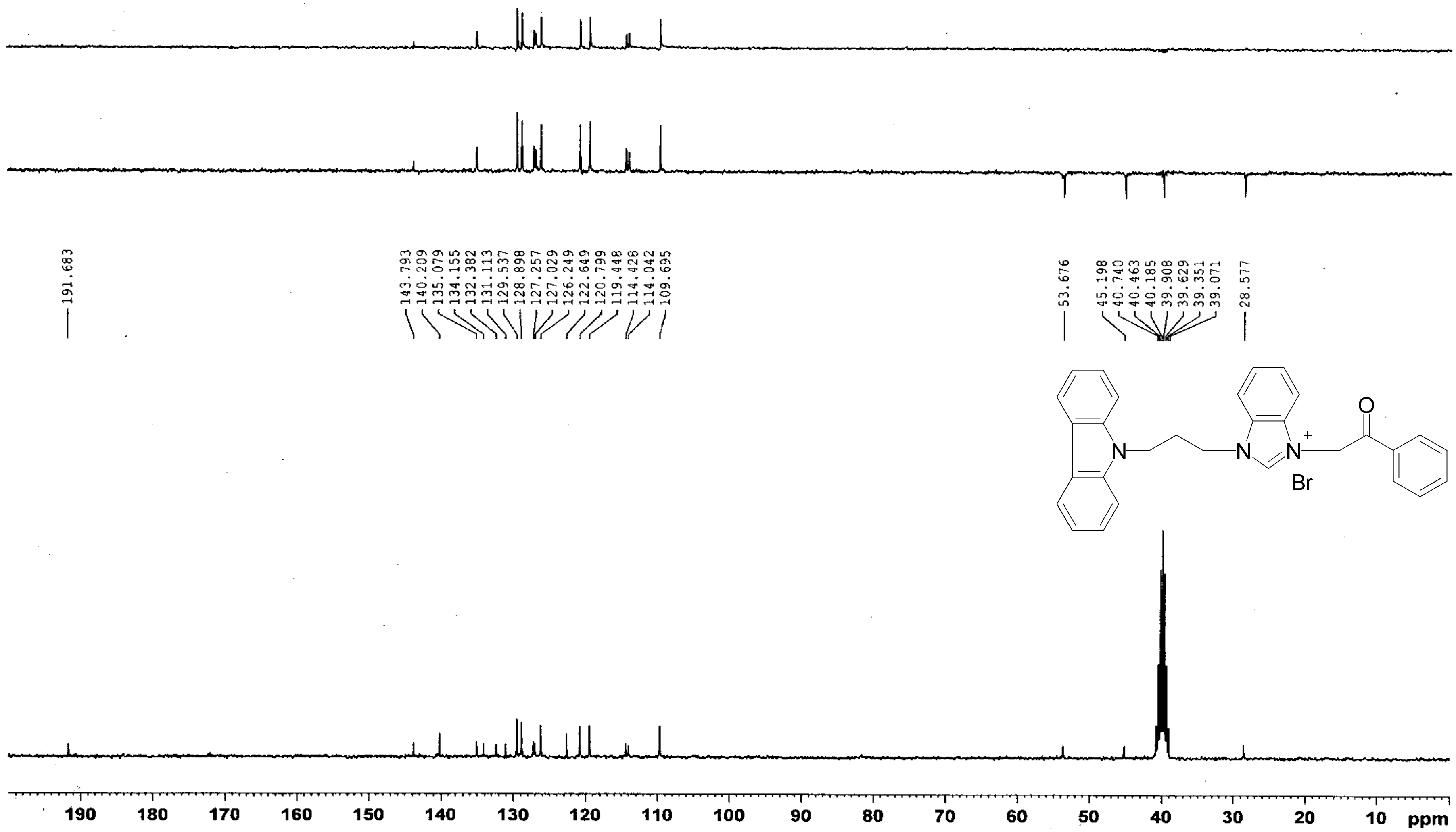
191.683
143.793
140.209
135.079
134.155
132.382
131.113
129.537
128.898
127.257
127.029
126.249
122.649
120.799
119.448
114.428
114.042
109.695
53.676
45.198
40.740
40.463
40.185
39.908
39.629
39.351
39.071
28.577
Br⁻
190
180
170
160
150
140
130
120
110
100
90
80
70
60
50
40
30
20
10
ppm

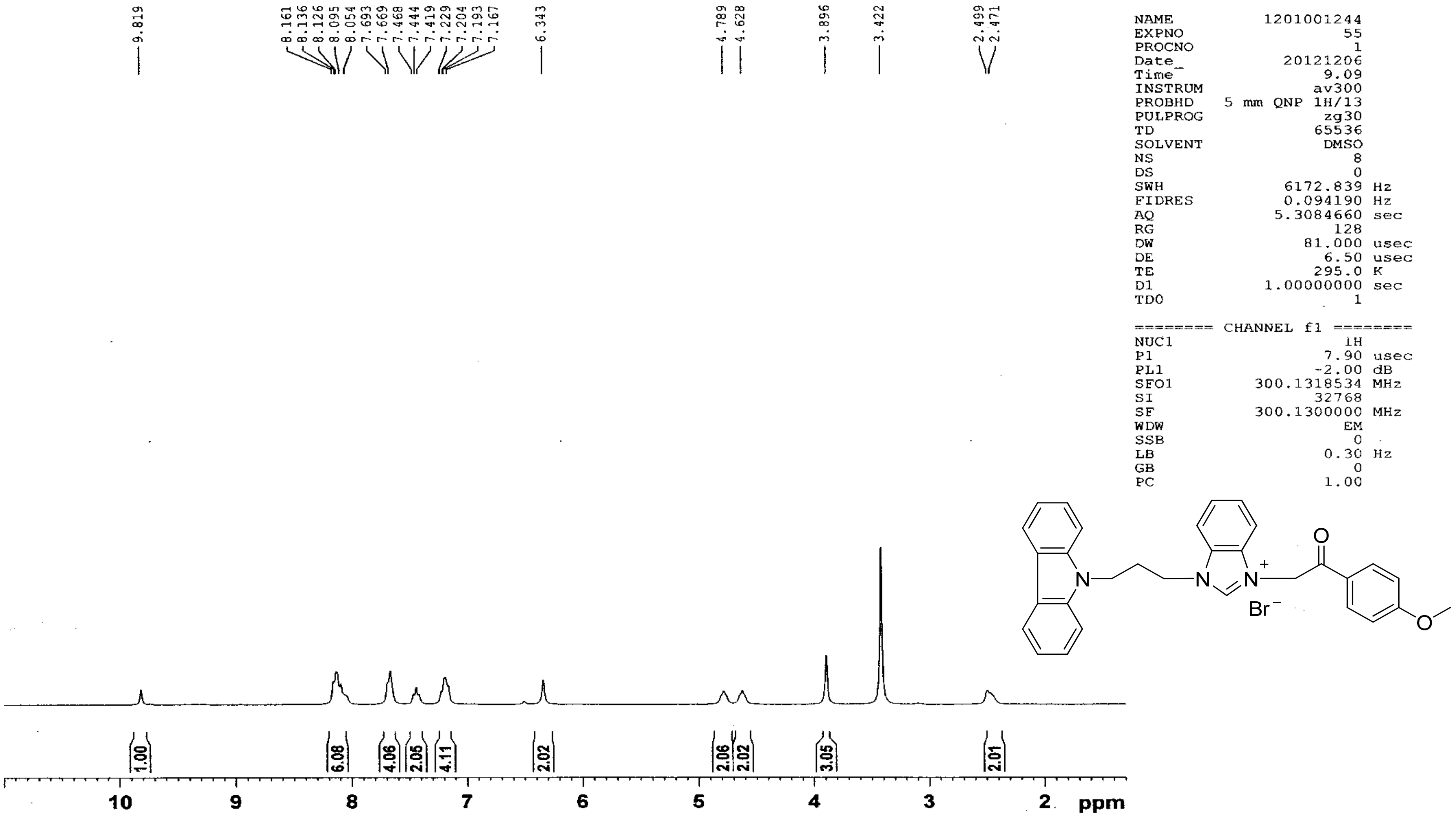
9.819
8.161
8.136
8.126
8.095
8.054
7.693
7.669
7.468
7.444
7.419
7.229
7.204
7.193
7.167
6.343
4.789
4.628
3.896
3.422
2.499
2.471
NAME 1201001244
EXPNO 55
PROCNO 1
Date_ 20121206
Time 9.09
INSTRUM av300
PROBHD 5 mm QNP 1H/13
PULPROG zg30
TD 65536
SOLVENT DMSO
NS 8
DS 0
SWH 6172.839 Hz
FIDRES 0.094190 Hz
AQ 5.3084660 sec
RG 128
DW 81.000 usec
DE 6.50 usec
TE 295.0 K
D1 1.00000000 sec
TD0 1
======== CHANNEL f1 ========
NUC1 1H
P1 7.90 usec
PL1 -2.00 dB
SFO1 300.1318534 MHz
SI 32768
SF 300.1300000 MHz
WDW EM
SSB 0
LB 0.30 Hz
GB 0
PC 1.00
N
N
N
+
O
Br⁻
O
1.00
6.08
4.06
2.05
4.11
2.02
2.06
2.02
3.05
2.01
10
9
8
7
6
5
4
3
2
ppm

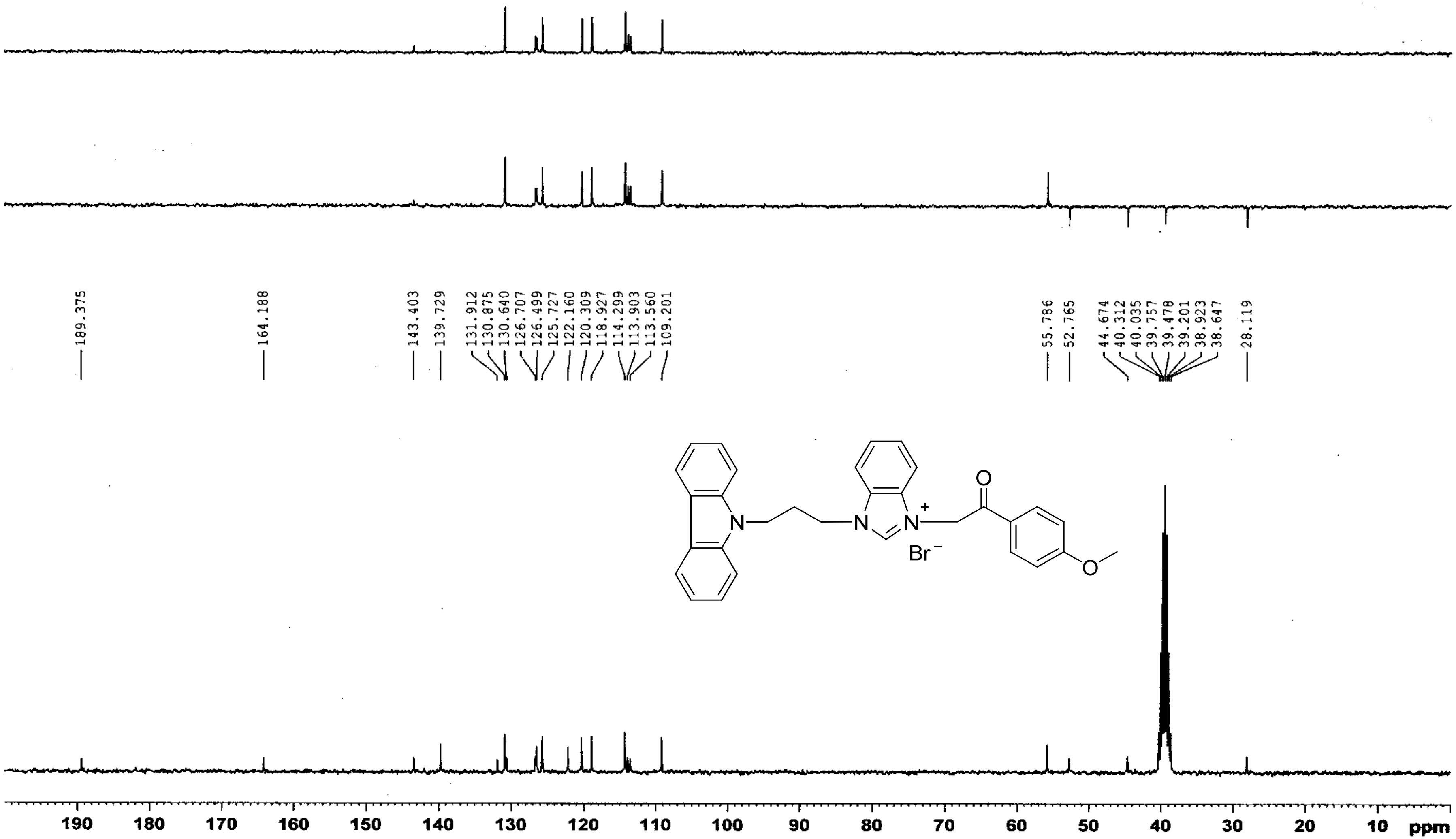

189.375
164.188
143.403
139.729
131.912
130.875
130.640
126.707
126.499
125.727
122.160
120.309
118.927
114.299
113.903
113.560
109.201
55.786
52.765
44.674
40.312
40.035
39.757
39.478
39.201
38.923
38.647
28.119
N
N
N+
O
O
Br−
190 180 170 160 150 140 130 120 110 100 90 80 70 60 50 40 30 20 10 ppm

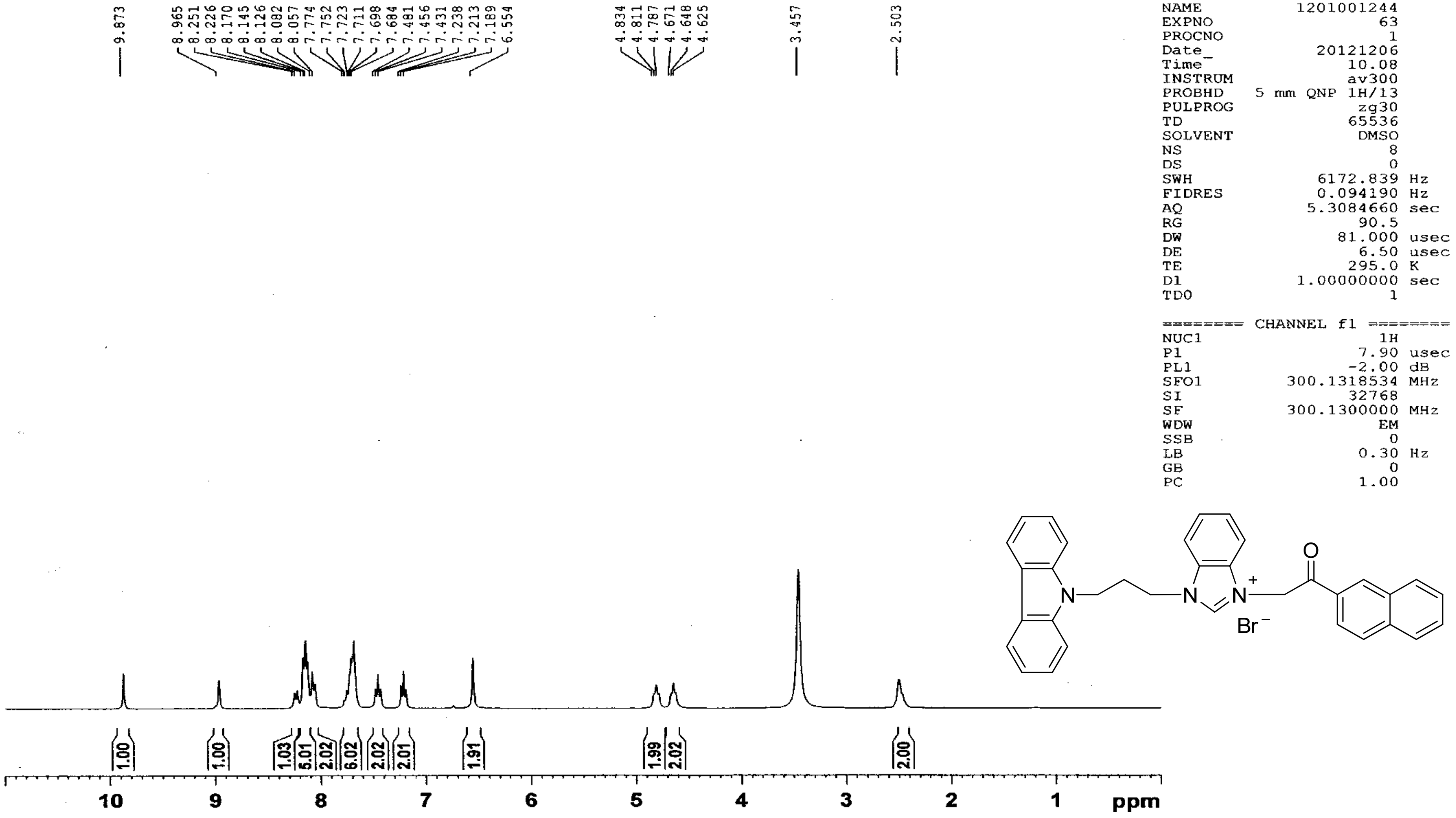

9.873
8.965
8.251
8.226
8.170
8.145
8.126
8.082
8.057
7.774
7.752
7.723
7.711
7.698
7.684
7.481
7.456
7.431
7.238
7.213
7.189
6.554
4.834
4.811
4.787
4.671
4.648
4.625
3.457
2.503
NAME 1201001244
EXPNO 63
PROCNO 1
Date_ 20121206
Time 10.08
INSTRUM av300
PROBHD 5 mm QNP 1H/13
PULPROG zg30
TD 65536
SOLVENT DMSO
NS 8
DS 0
SWH 6172.839 Hz
FIDRES 0.094190 Hz
AQ 5.3084660 sec
RG 90.5
DW 81.000 usec
DE 6.50 usec
TE 295.0 K
D1 1.00000000 sec
TD0 1
======== CHANNEL f1 ========
NUC1 1H
P1 7.90 usec
PL1 -2.00 dB
SFO1 300.1318534 MHz
SI 32768
SF 300.1300000 MHz
WDW EM
SSB 0
LB 0.30 Hz
GB 0
PC 1.00
N
N
N
+
O
Br⁻
1.00
1.00
1.03
5.01
2.02
6.02
2.02
2.01
1.91
1.99
2.02
2.00
10
9
8
7
6
5
4
3
2
1
ppm

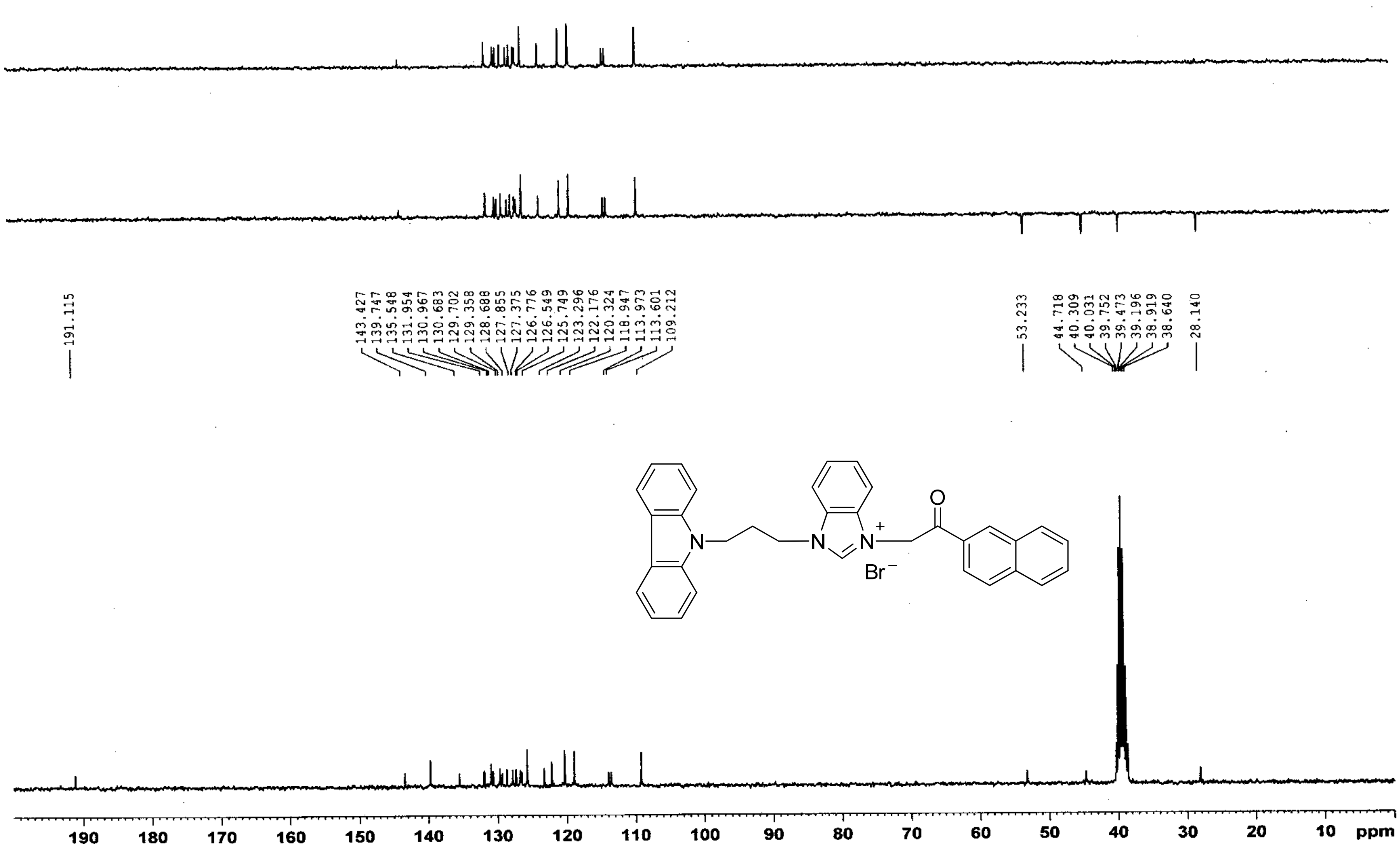
191.115
143.427
139.747
135.548
131.954
130.967
130.683
129.702
129.358
128.688
127.855
127.375
126.776
126.549
125.749
123.296
122.176
120.324
118.947
113.973
113.601
109.212
53.233
44.718
40.309
40.031
39.752
39.473
39.196
38.919
38.640
28.140
N
N
N
O
Br⁻
190 180 170 160 150 140 130 120 110 100 90 80 70 60 50 40 30 20 10 ppm

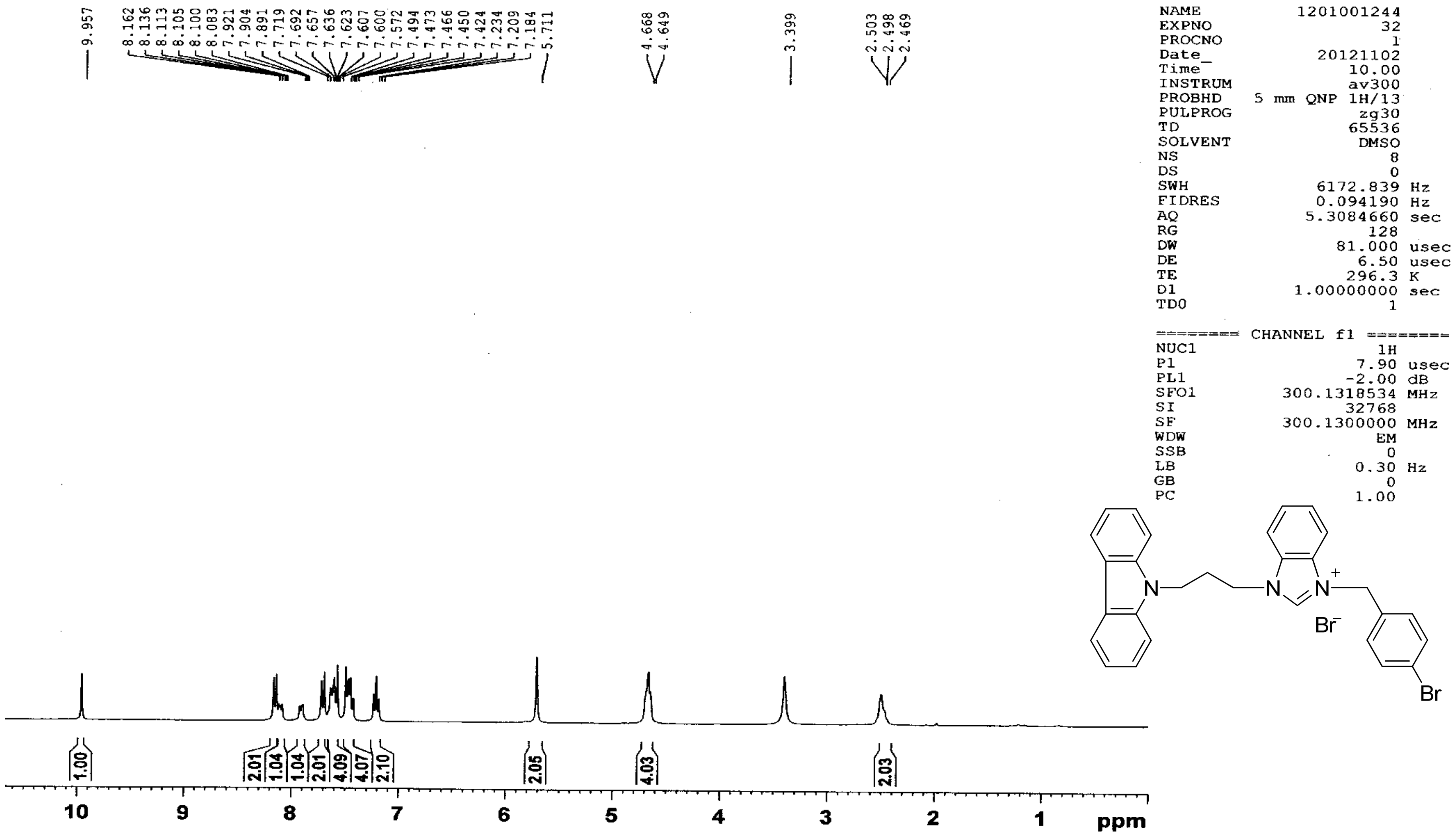
9.957
8.162 8.136 8.113 8.105 8.100 8.083 7.921 7.904 7.891 7.719 7.692 7.657 7.636 7.623 7.607 7.600 7.572 7.494 7.473 7.466 7.450 7.424 7.234 7.209 7.184 5.711
4.668 4.649
3.399
2.503 2.498 2.469
1.00 2.01 1.04 1.04 2.01 4.09 4.07 2.10 2.05 4.03 2.03
10 9 8 7 6 5 4 3 2 1 ppm
NAME 1201001244
EXPNO 32
PROCNO 1
Date_ 20121102
Time 10.00
INSTRUM av300
PROBHD 5 mm QNP 1H/13
PULPROG zg30
TD 65536
SOLVENT DMSO
NS 8
DS 0
SWH 6172.839 Hz
FIDRES 0.094190 Hz
AQ 5.3084660 sec
RG 128
DW 81.000 usec
DE 6.50 usec
TE 296.3 K
D1 1.00000000 sec
TD0 1
======== CHANNEL f1 ========
NUC1 1H
P1 7.90 usec
PL1 -2.00 dB
SFO1 300.1318534 MHz
SI 32768
SF 300.1300000 MHz
WDW EM
SSB 0
LB 0.30 Hz
GB 0
PC 1.00
N
N
N+
Br⁻
Br

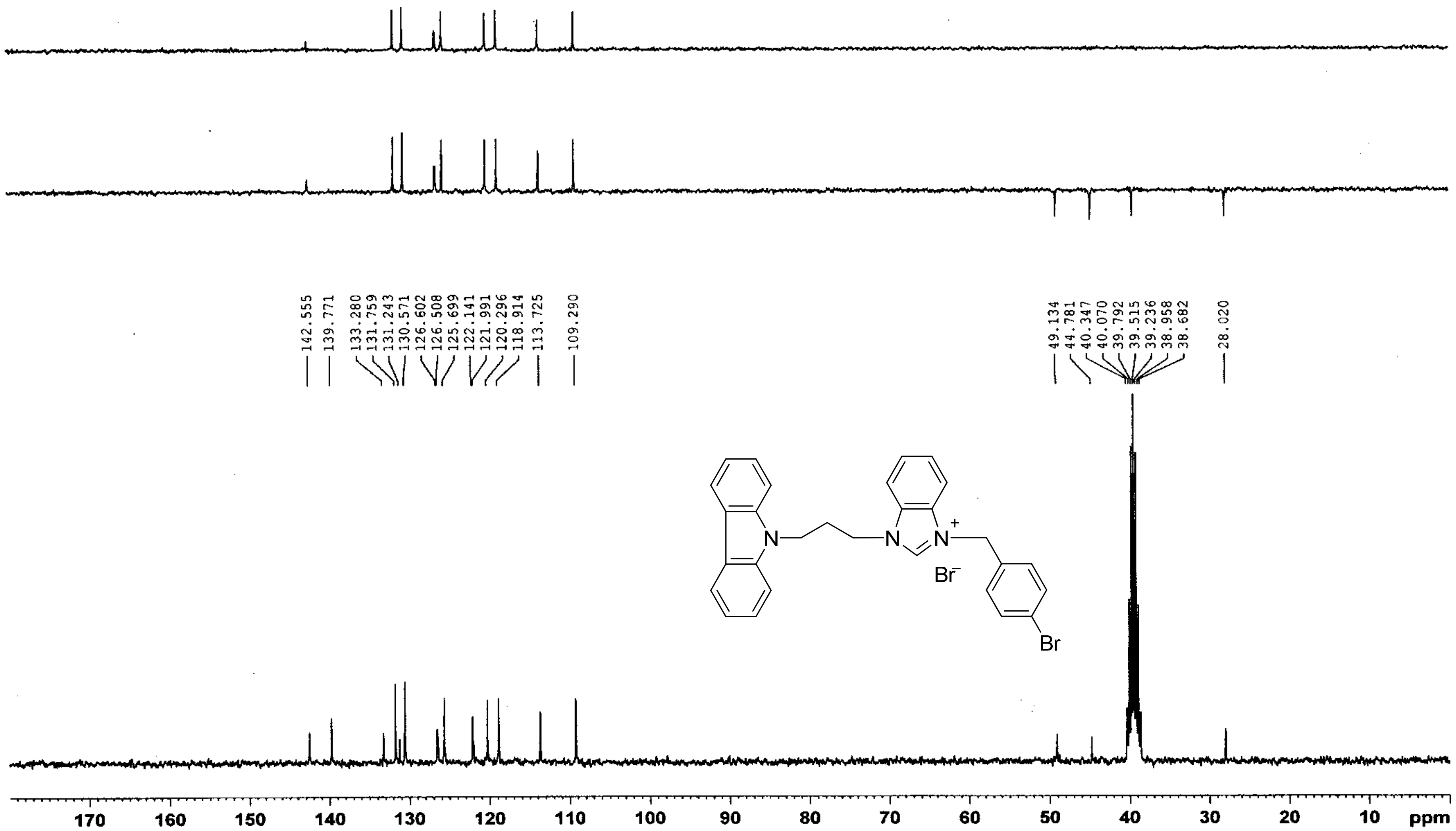

142.555
139.771
133.280
131.759
131.243
130.571
126.602
126.508
125.699
122.141
121.991
120.296
118.914
113.725
109.290
49.134
44.781
40.347
40.070
39.792
39.515
39.236
38.958
38.682
28.020
N
N
N
Br⁻
Br
170 160 150 140 130 120 110 100 90 80 70 60 50 40 30 20 10 ppm

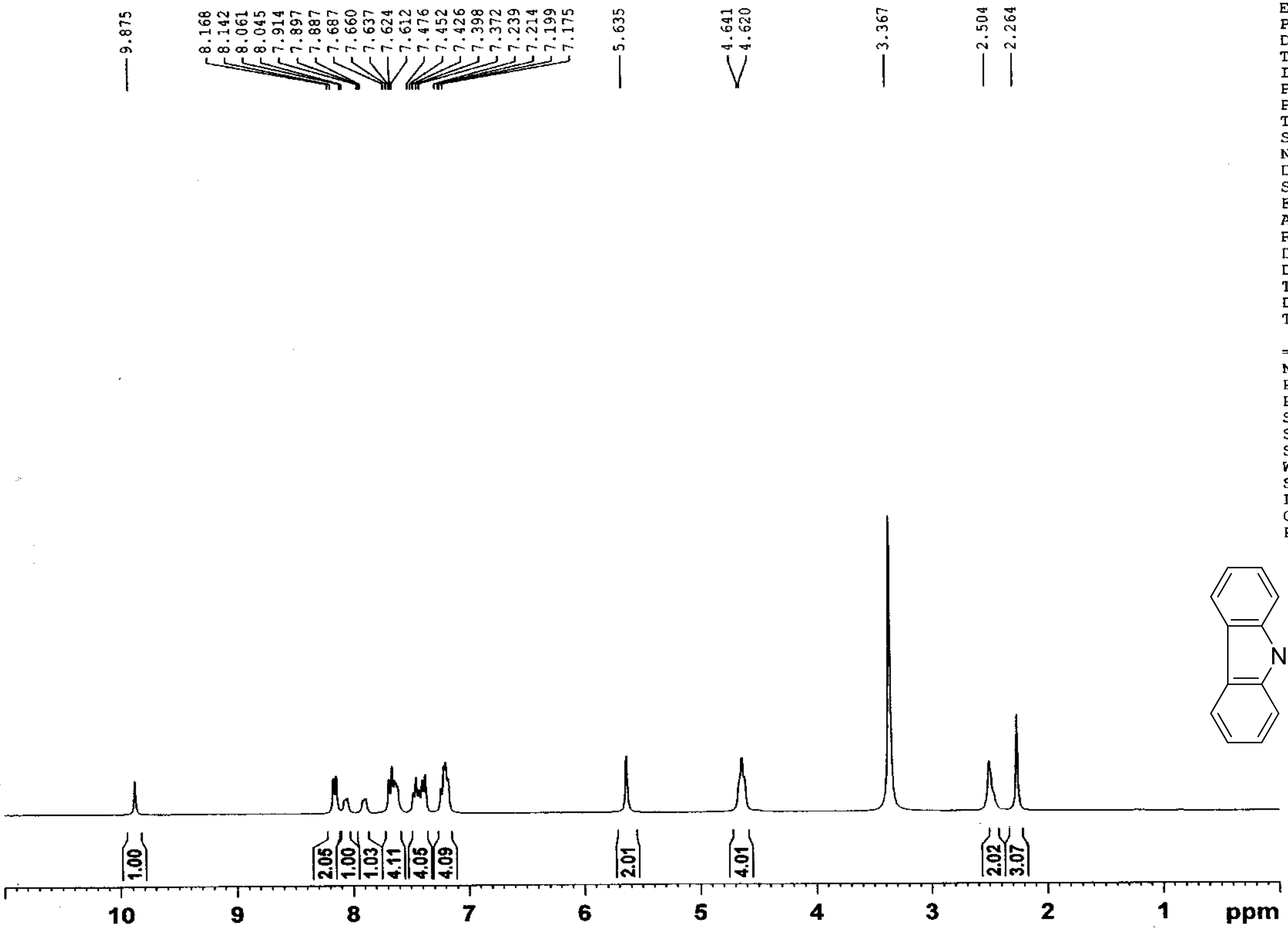


```
NAME          1201001244
EXPNO                 37
PROCNO                 1
Date_           20121102
Time               10.19
INSTRUM            av300
PROBHD   5 mm QNP 1H/13
PULPROG             zg30
TD                 65536
SOLVENT             DMSO
NS                     8
DS                     0
SWH             6172.839 Hz
FIDRES          0.094190 Hz
AQ             5.3084660 sec
RG                 228.1
DW                81.000 usec
DE                  6.50 usec
TE                 296.3 K
D1            1.00000000 sec
TD0                    1

======== CHANNEL f1 ========
NUC1                  1H
P1                  7.90 usec
PL1                -2.00 dB
SFO1         300.1318534 MHz
SI                 32768
SF           300.1300000 MHz
WDW                   EM
SSB                    0
LB                  0.30 Hz
GB                     0
PC                  1.00
```

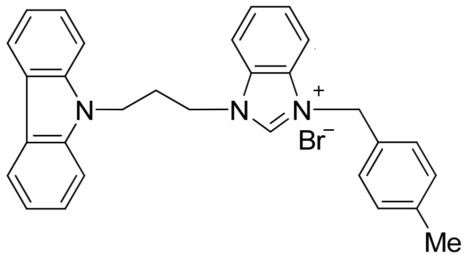

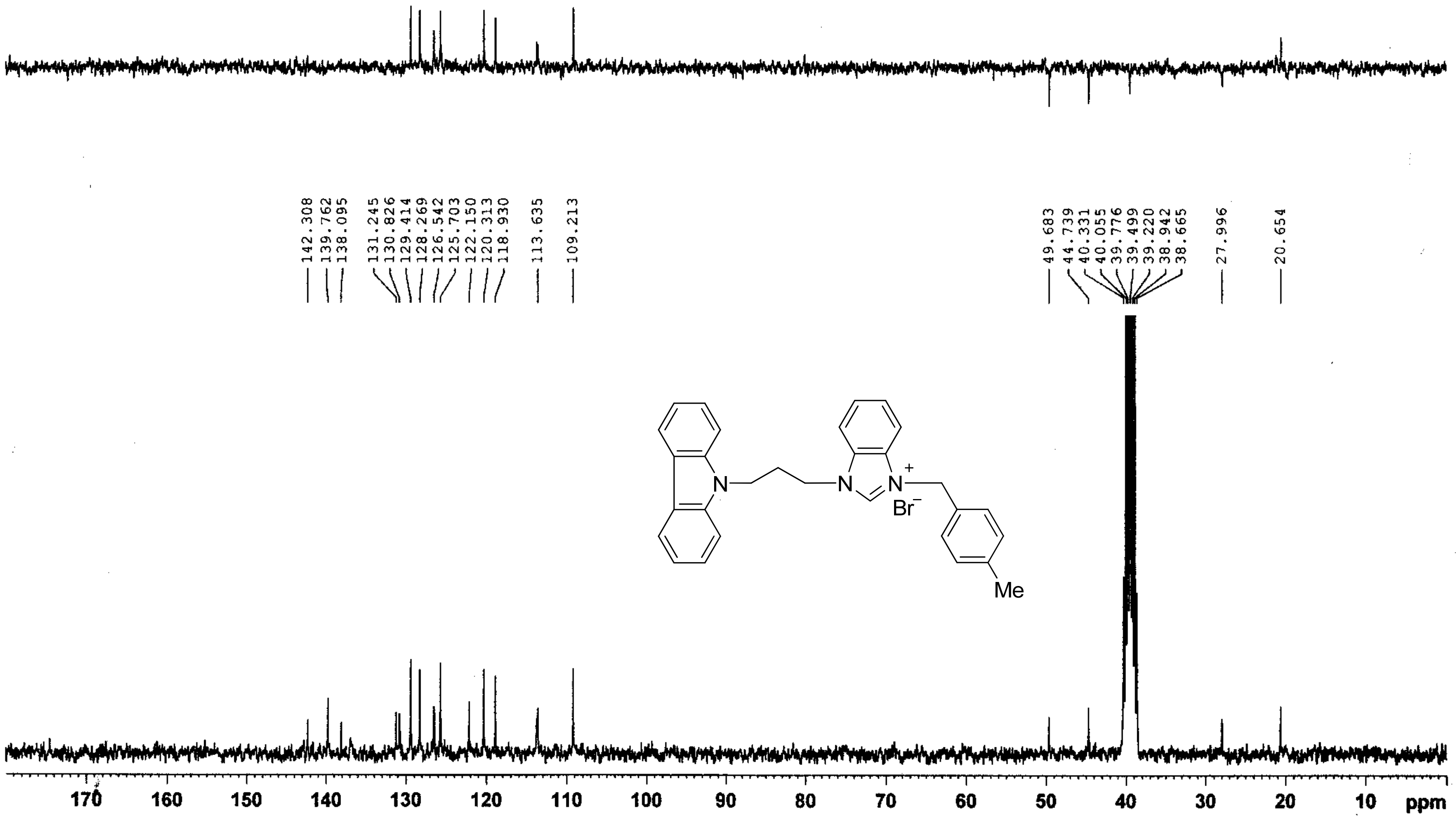
142.308
139.762
138.095
131.245
130.826
129.414
128.269
126.542
125.703
122.150
120.313
118.930
113.635
109.213
49.683
44.739
40.331
40.055
39.776
39.499
39.220
38.942
38.665
27.996
20.654
N
N
N+
Br−
Me
170 160 150 140 130 120 110 100 90 80 70 60 50 40 30 20 10 ppm

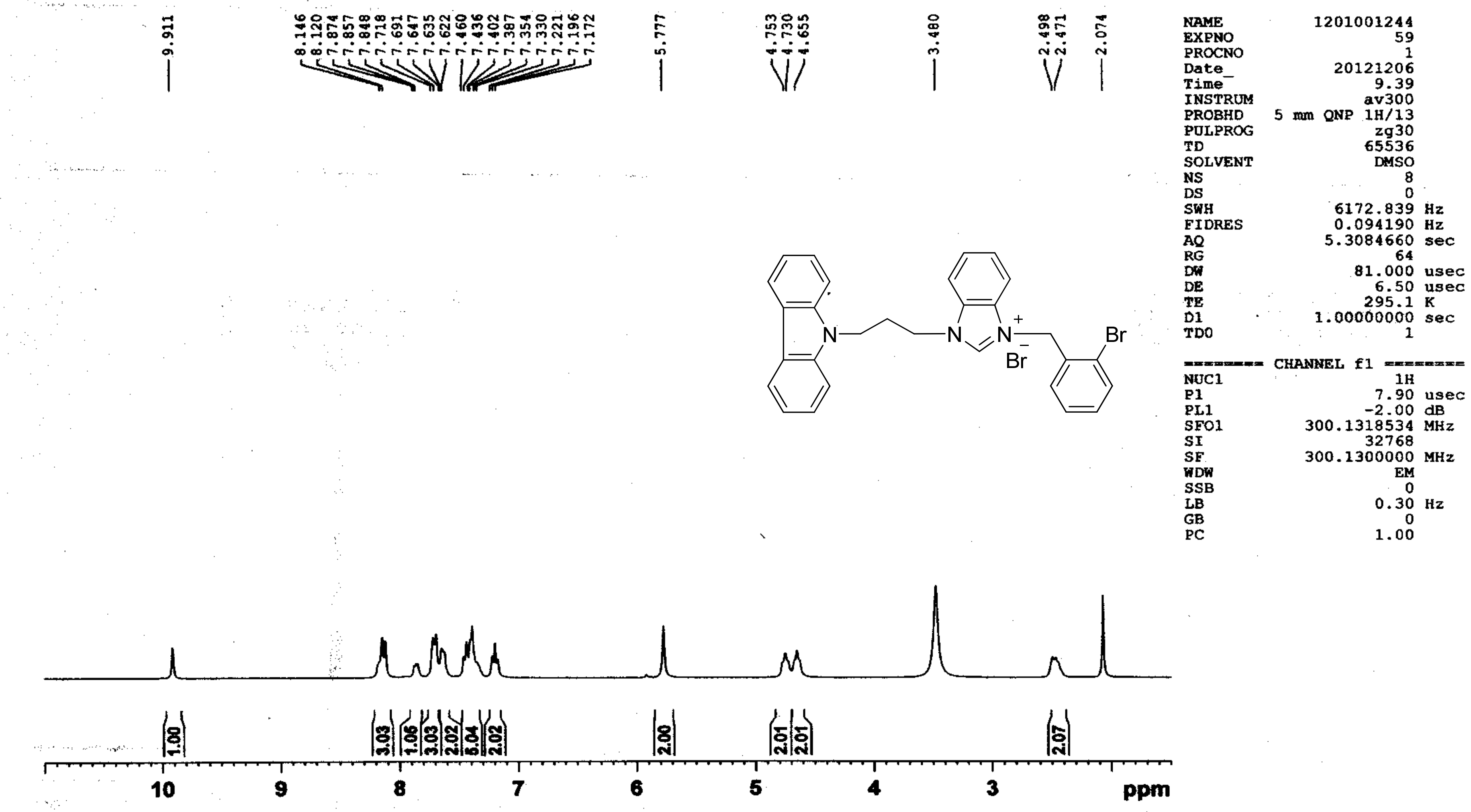

9.911
8.146
8.120
7.874
7.857
7.848
7.718
7.691
7.647
7.635
7.622
7.460
7.436
7.402
7.387
7.354
7.330
7.221
7.196
7.172
5.777
4.753
4.730
4.655
3.480
2.498
2.471
2.074
N
N
N
+
Br
Br
−
NAME 1201001244
EXPNO 59
PROCNO 1
Date_ 20121206
Time 9.39
INSTRUM av300
PROBHD 5 mm QNP 1H/13
PULPROG zg30
TD 65536
SOLVENT DMSO
NS 8
DS 0
SWH 6172.839 Hz
FIDRES 0.094190 Hz
AQ 5.3084660 sec
RG 64
DW 81.000 usec
DE 6.50 usec
TE 295.1 K
D1 1.00000000 sec
TD0 1
======== CHANNEL f1 ========
NUC1 1H
P1 7.90 usec
PL1 -2.00 dB
SFO1 300.1318534 MHz
SI 32768
SF 300.1300000 MHz
WDW EM
SSB 0
LB 0.30 Hz
GB 0
PC 1.00
1.00
3.03
1.06
3.03
2.02
6.04
2.02
2.00
2.01
2.01
2.07
10
9
8
7
6
5
4
3
ppm

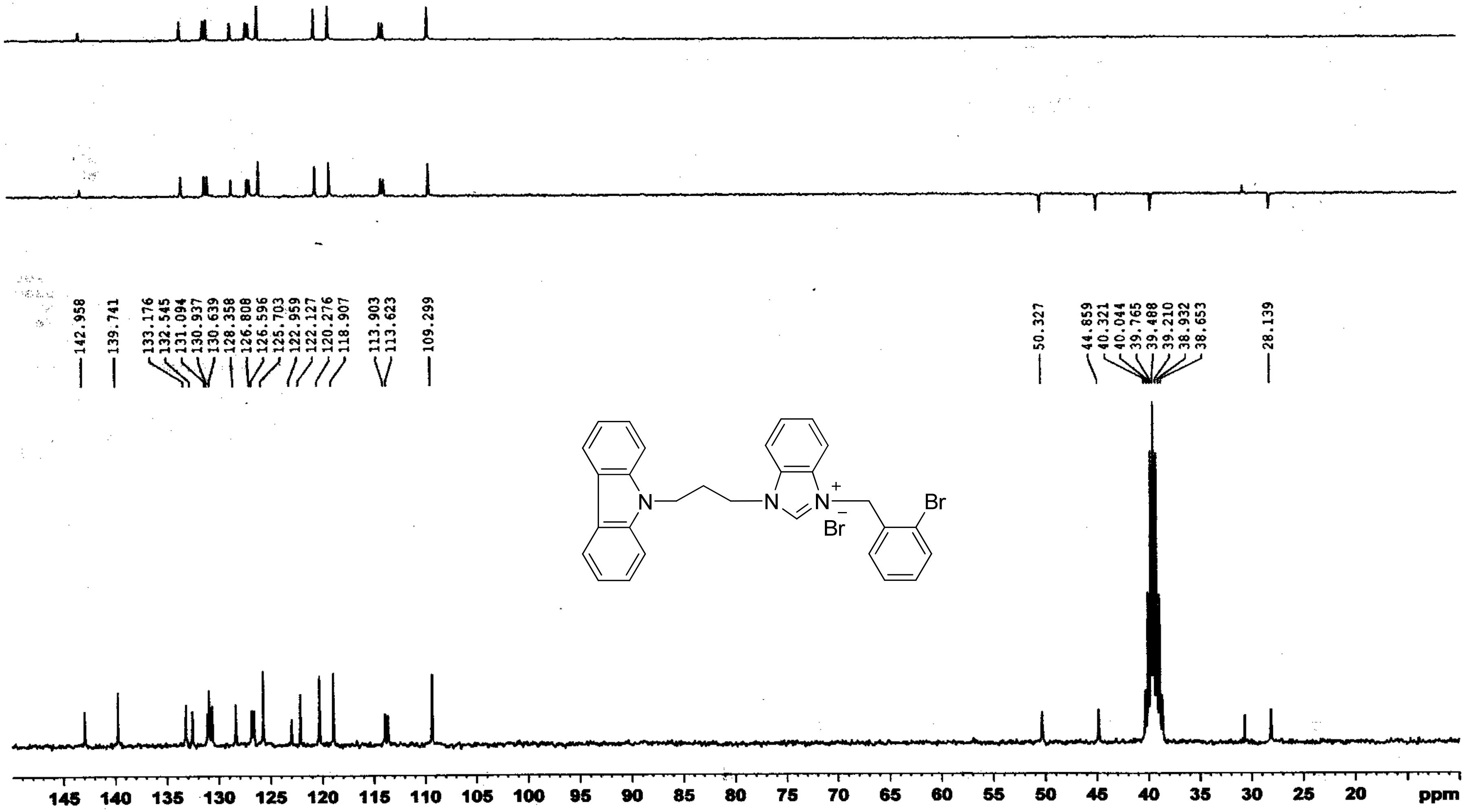

142.958
139.741
133.176
132.545
131.094
130.937
130.639
128.358
126.808
126.596
125.703
122.959
122.127
120.276
118.907
113.903
113.623
109.299
50.327
44.859
40.321
40.044
39.765
39.488
39.210
38.932
38.653
28.139
N
N
N
+
Br
−
Br
145
140
135
130
125
120
115
110
105
100
95
90
85
80
75
70
65
60
55
50
45
40
35
30
25
20
ppm

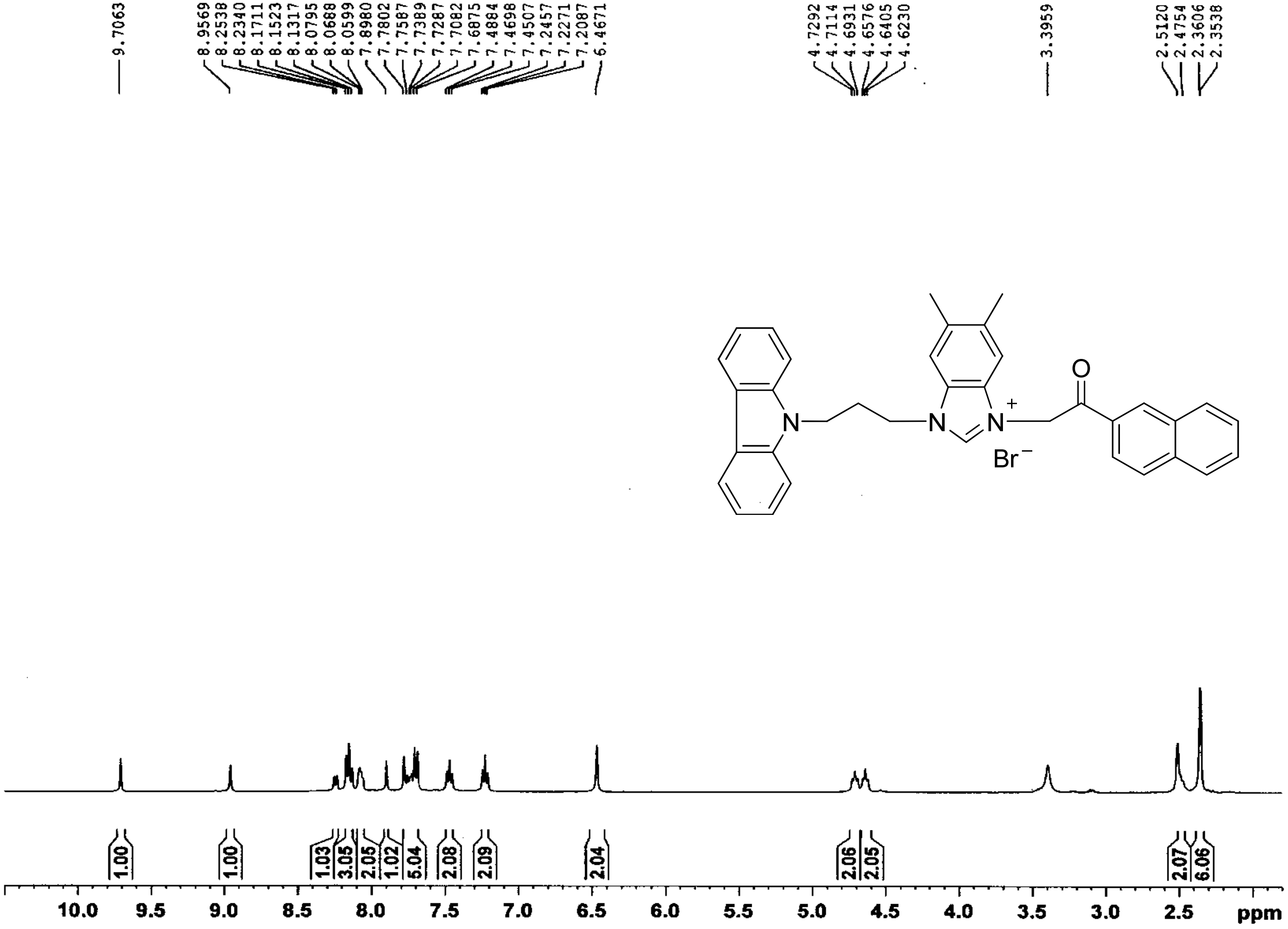

NAME	22012000180	
EXPNO	194	
PROCNO	1	
Date_	20121231	
Time	12.30	
INSTRUM	spect	
PROBHD	5 mm PABBO BB/	
PULPROG	zg30	
TD	65536	
SOLVENT	DMSO	
NS	8	
DS	0	
SWH	8012.820	Hz
FIDRES	0.122266	Hz
AQ	4.0894966	sec
RG	29.1	
DW	62.400	usec
DE	6.50	usec
TE	297.0	K
D1	1.00000000	sec
TD0	1	

======== CHANNEL f1 ========

SFO1	400.1524711	MHz
NUC1	1H	
P1	9.64	usec
SI	65536	
SF	400.1499974	MHz
WDW	EM	
SSB	0	
LB	0.30	Hz
GB	0	
PC	1.00	

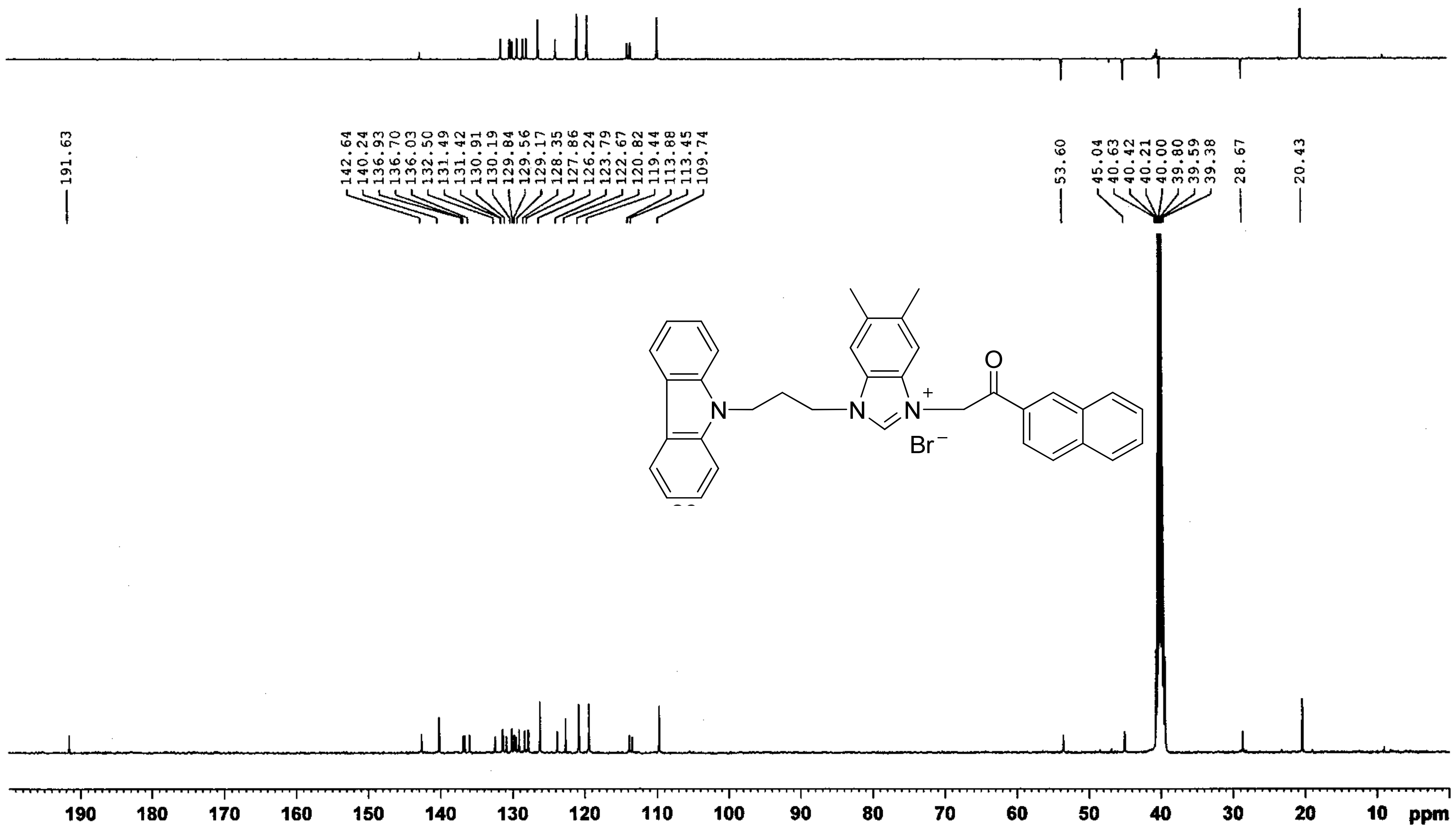
191.63
142.64
140.24
136.93
136.70
136.03
132.50
131.49
131.42
130.91
130.19
129.84
129.56
129.17
128.35
127.86
126.24
123.79
122.67
120.82
119.44
113.88
113.45
109.74
53.60
45.04
40.63
40.42
40.21
40.00
39.80
39.59
39.38
28.67
20.43
O
N
N
N
+
Br
−
190
180
170
160
150
140
130
120
110
100
90
80
70
60
50
40
30
20
10
ppm

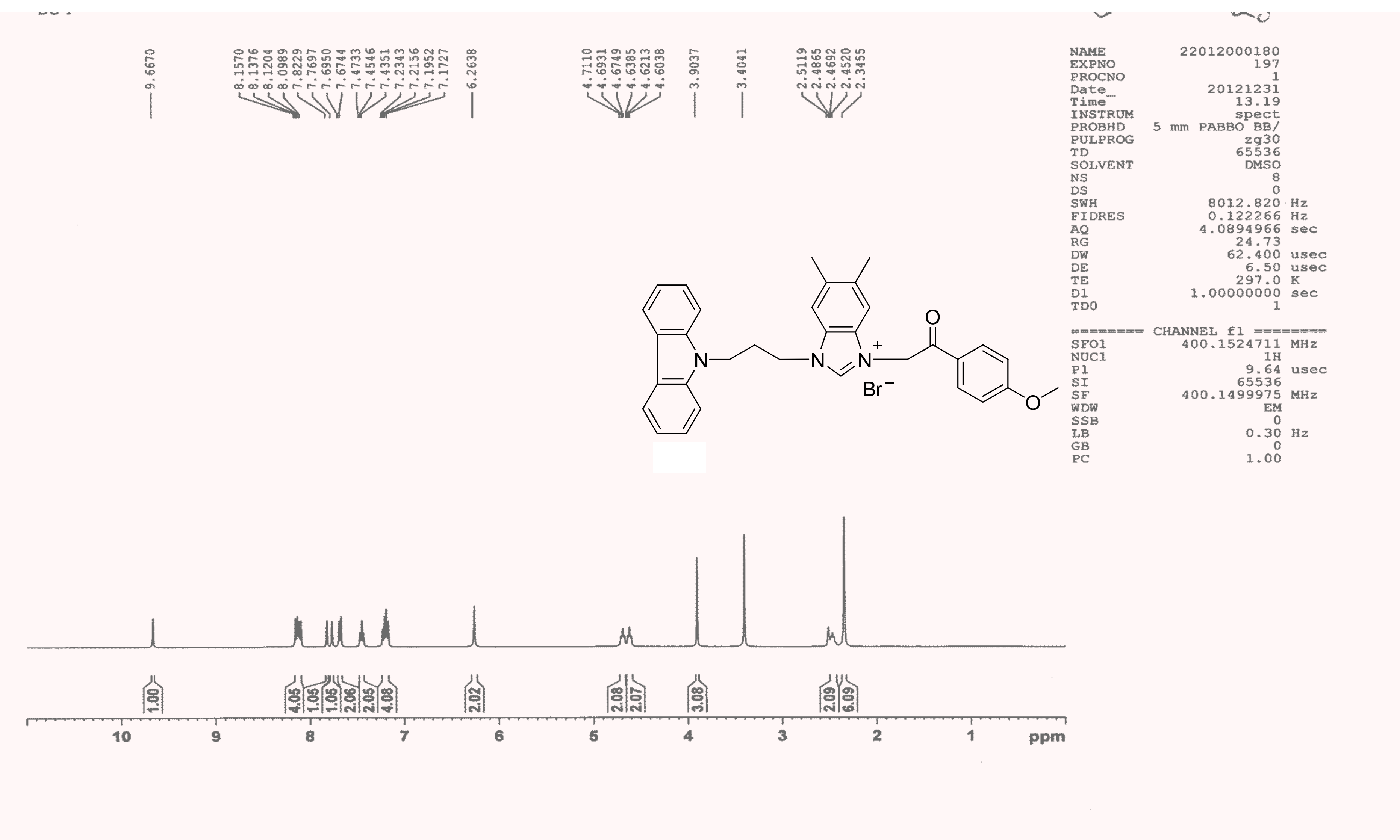
9.6670
8.1570
8.1376
8.1204
8.0989
7.8229
7.7697
7.6950
7.6744
7.4733
7.4546
7.4351
7.2343
7.2156
7.1952
7.1727
6.2638
4.7110
4.6931
4.6749
4.6385
4.6213
4.6038
3.9037
3.4041
2.5119
2.4865
2.4692
2.4520
2.3455
NAME 22012000180
EXPNO 197
PROCNO 1
Date_ 20121231
Time 13.19
INSTRUM spect
PROBHD 5 mm PABBO BB/
PULPROG zg30
TD 65536
SOLVENT DMSO
NS 8
DS 0
SWH 8012.820 Hz
FIDRES 0.122266 Hz
AQ 4.0894966 sec
RG 24.73
DW 62.400 usec
DE 6.50 usec
TE 297.0 K
D1 1.00000000 sec
TD0 1
======== CHANNEL f1 ========
SFO1 400.1524711 MHz
NUC1 1H
P1 9.64 usec
SI 65536
SF 400.1499975 MHz
WDW EM
SSB 0
LB 0.30 Hz
GB 0
PC 1.00
N
N
N+
Br−
O
O
1.00
4.05
1.05
1.05
2.06
2.06
2.05
4.08
2.02
2.08
2.07
3.08
2.09
6.09
10
9
8
7
6
5
4
3
2
1
ppm

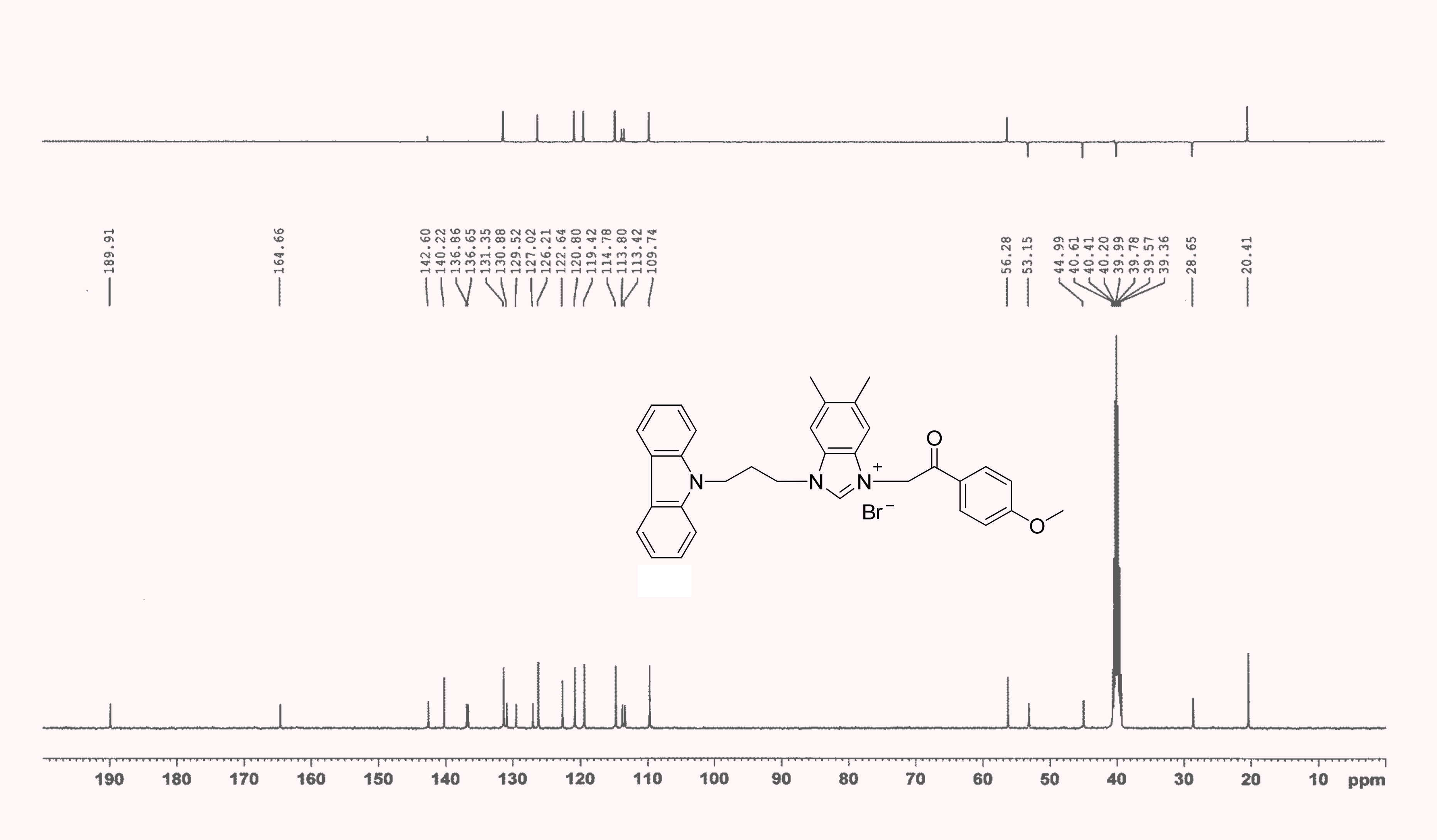
189.91
164.66
142.60
140.22
136.86
136.65
131.35
130.88
129.52
127.02
126.21
122.64
120.80
119.42
114.78
113.80
113.42
109.74
56.28
53.15
44.99
40.61
40.41
40.20
39.99
39.78
39.57
39.36
28.65
20.41
N
N
N
+
Br
−
O
O
190 180 170 160 150 140 130 120 110 100 90 80 70 60 50 40 30 20 10 ppm

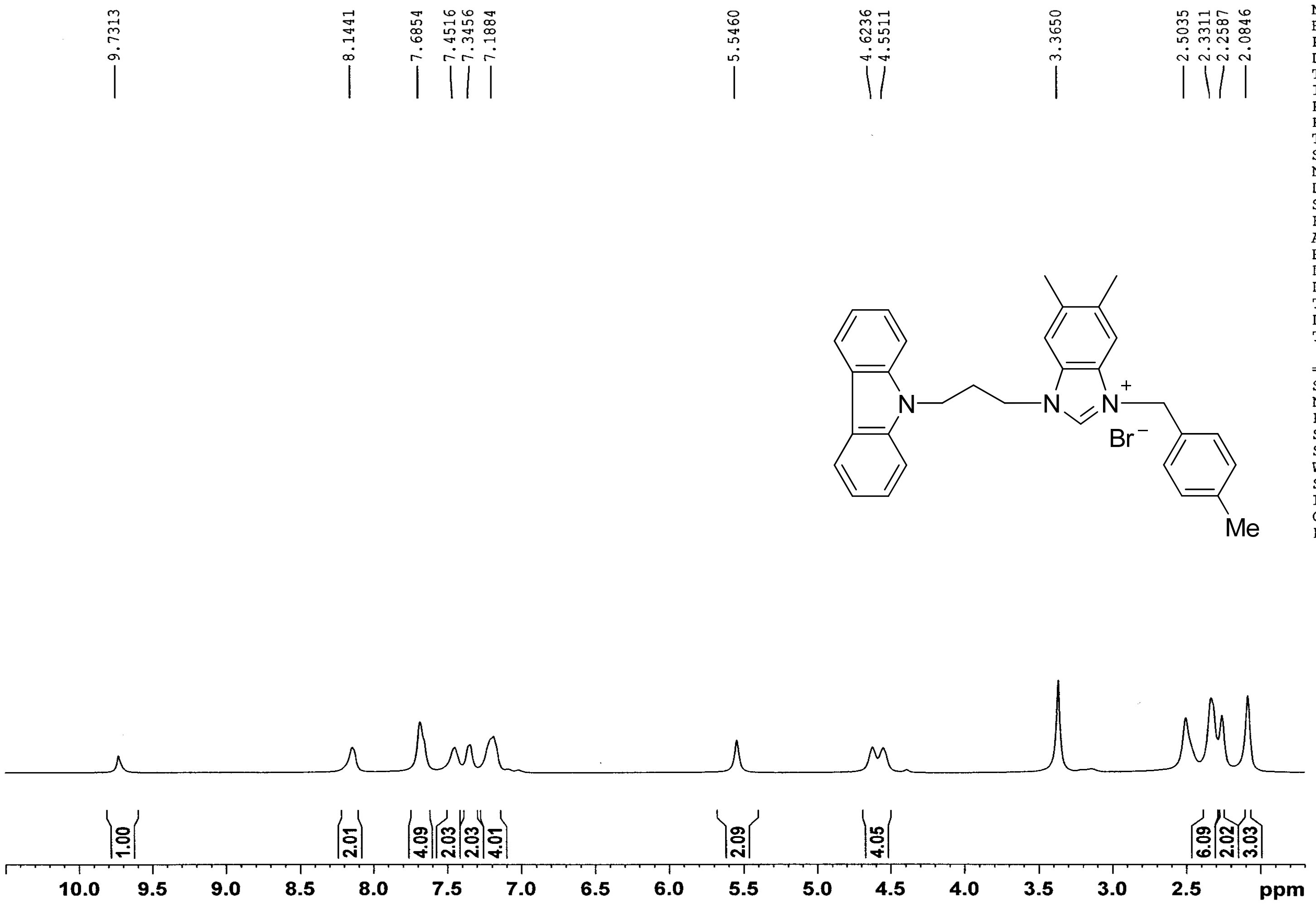


```
NAME            22012000180
EXPNO                   200
PROCNO                    1
Date_              20121231
Time                  14.09
INSTRUM               spect
PROBHD     5 mm PABBO BB/
PULPROG                zg30
TD                    65536
SOLVENT                DMSO
NS                        8
DS                        0
SWH               8012.820 Hz
FIDRES            0.122266 Hz
AQ               4.0894966 sec
RG                    36.33
DW                   62.400 usec
DE                     6.50 usec
TE                    297.0 K
D1               1.00000000 sec
TD0                       1

======== CHANNEL f1 ========
SFO1          400.1524711 MHz
NUC1                   1H
P1                   9.64 usec
SI                  65536
SF            400.1500000 MHz
WDW                    EM
SSB                     0
LB                   0.30 Hz
GB                      0
PC                   1.00
```

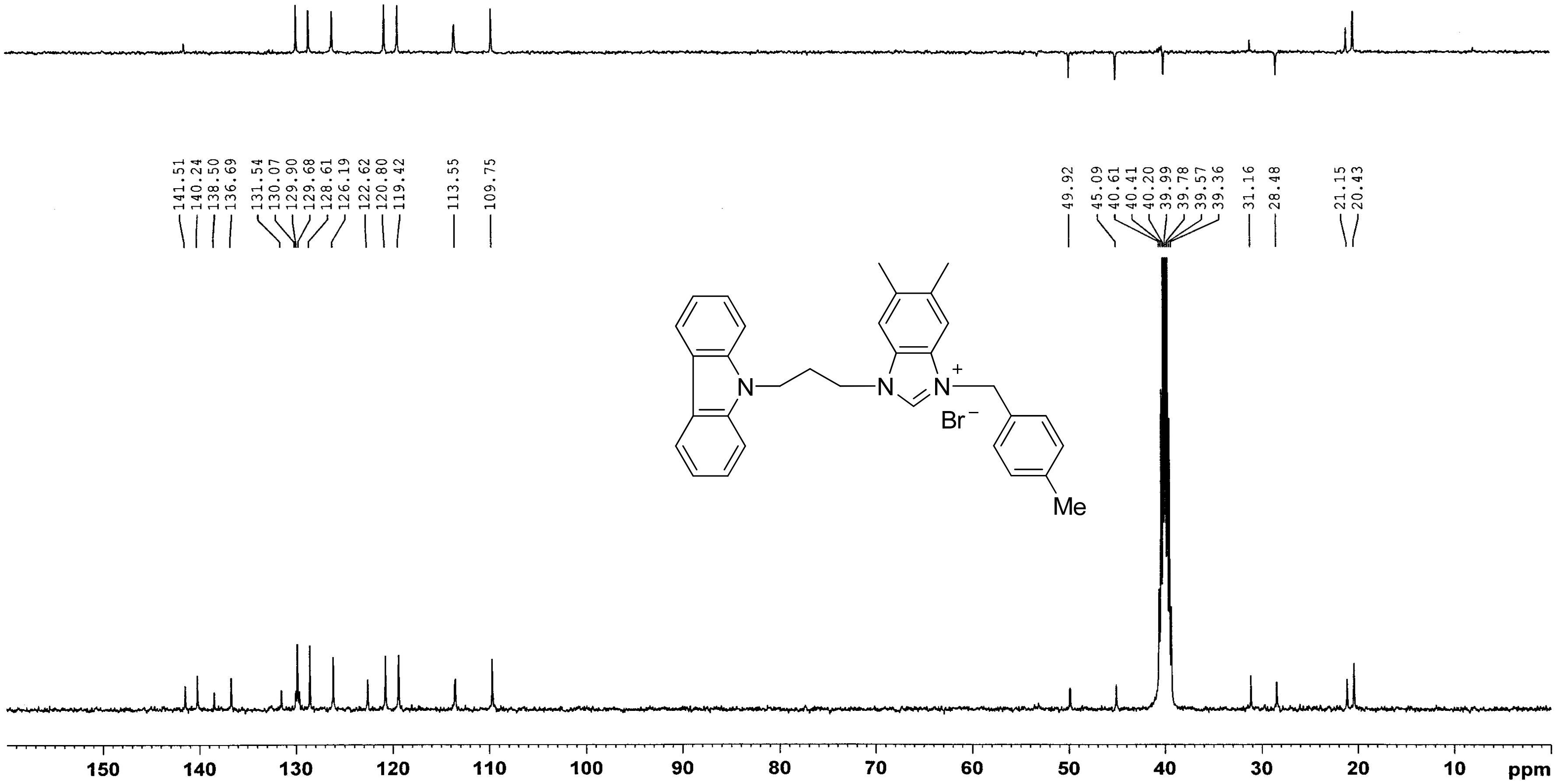
141.51
140.24
138.50
136.69
131.54
130.07
129.90
129.68
128.61
126.19
122.62
120.80
119.42
113.55
109.75
49.92
45.09
40.61
40.41
40.20
39.99
39.78
39.57
39.36
31.16
28.48
21.15
20.43
N
N
N
+
Br−
Me
150
140
130
120
110
100
90
80
70
60
50
40
30
20
10
ppm

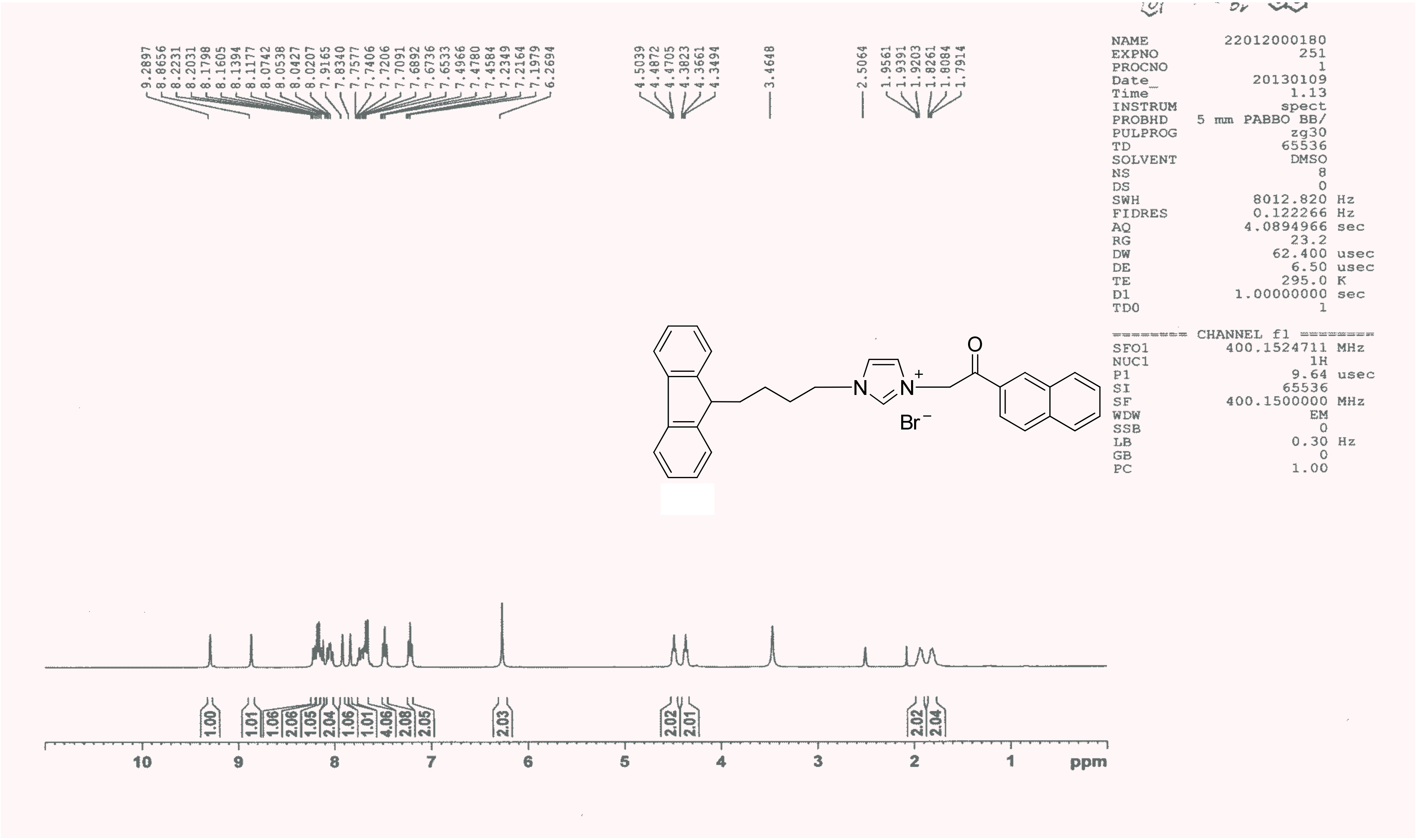

9.2897
8.8656
8.2231
8.2031
8.1798
8.1605
8.1394
8.1177
8.0742
8.0538
8.0427
8.0207
7.9165
7.8340
7.7577
7.7406
7.7206
7.7091
7.6892
7.6736
7.6533
7.4966
7.4780
7.4584
7.2349
7.2164
7.1979
6.2694
4.5039
4.4872
4.4705
4.3823
4.3661
4.3494
3.4648
2.5064
1.9561
1.9391
1.9203
1.8261
1.8084
1.7914
NAME 22012000180
EXPNO 251
PROCNO 1
Date_ 20130109
Time 1.13
INSTRUM spect
PROBHD 5 mm PABBO BB/
PULPROG zg30
TD 65536
SOLVENT DMSO
NS 8
DS 0
SWH 8012.820 Hz
FIDRES 0.122266 Hz
AQ 4.0894966 sec
RG 23.2
DW 62.400 usec
DE 6.50 usec
TE 295.0 K
D1 1.00000000 sec
TD0 1
======== CHANNEL f1 ========
SFO1 400.1524711 MHz
NUC1 1H
P1 9.64 usec
SI 65536
SF 400.1500000 MHz
WDW EM
SSB 0
LB 0.30 Hz
GB 0
PC 1.00
O
N
N+
Br−
1.00
1.01
1.06
2.06
1.05
2.04
1.06
1.01
4.06
2.08
2.05
2.03
2.02
2.01
2.02
2.04
10
9
8
7
6
5
4
3
2
1
ppm

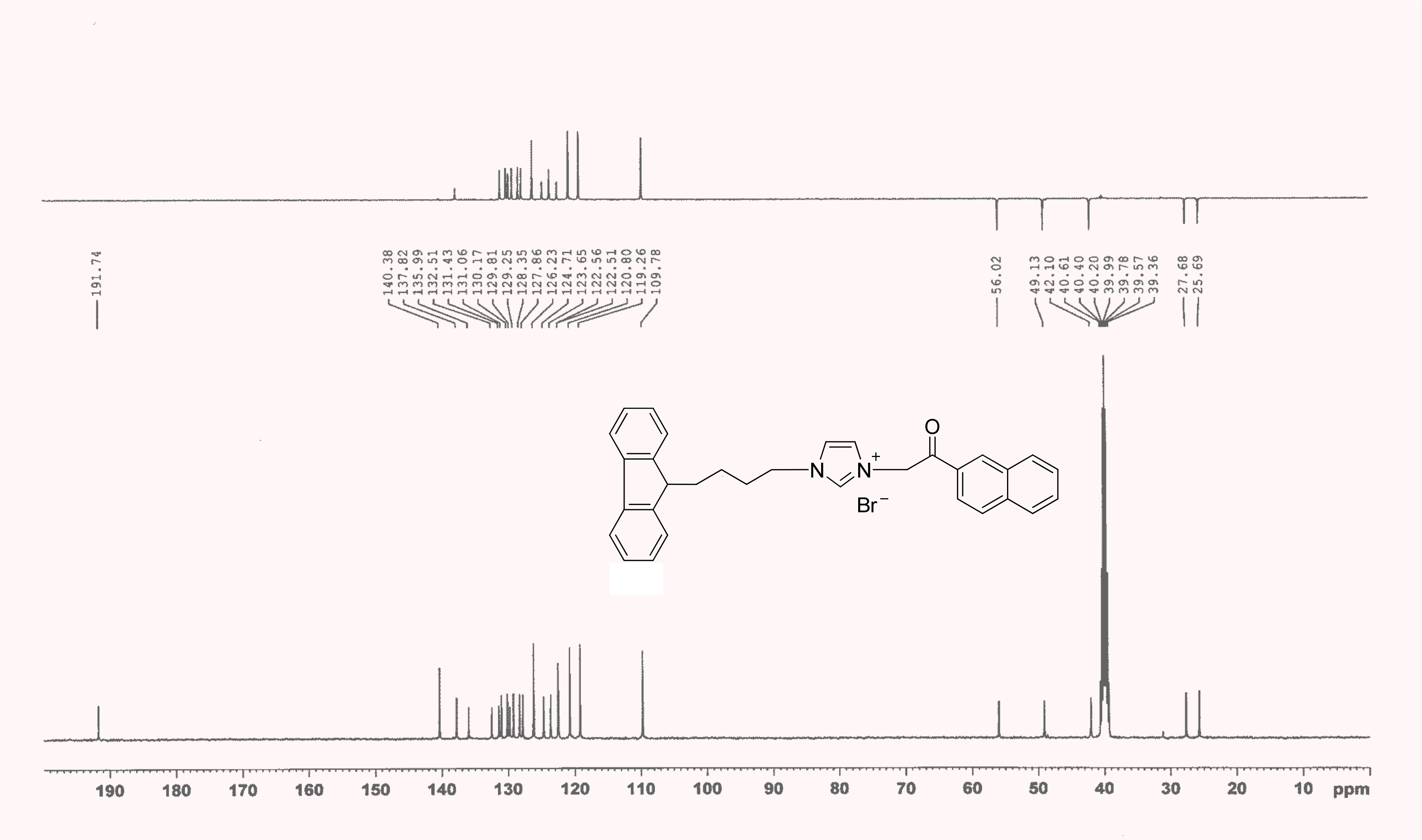
191.74
140.38
137.82
135.99
132.51
131.43
131.06
130.17
129.81
129.25
128.35
127.86
126.23
124.71
123.65
122.56
122.51
120.80
119.26
109.78
56.02
49.13
42.10
40.61
40.40
40.20
39.99
39.78
39.57
39.36
27.68
25.69
O
N
N+
Br−
190
180
170
160
150
140
130
120
110
100
90
80
70
60
50
40
30
20
10
ppm

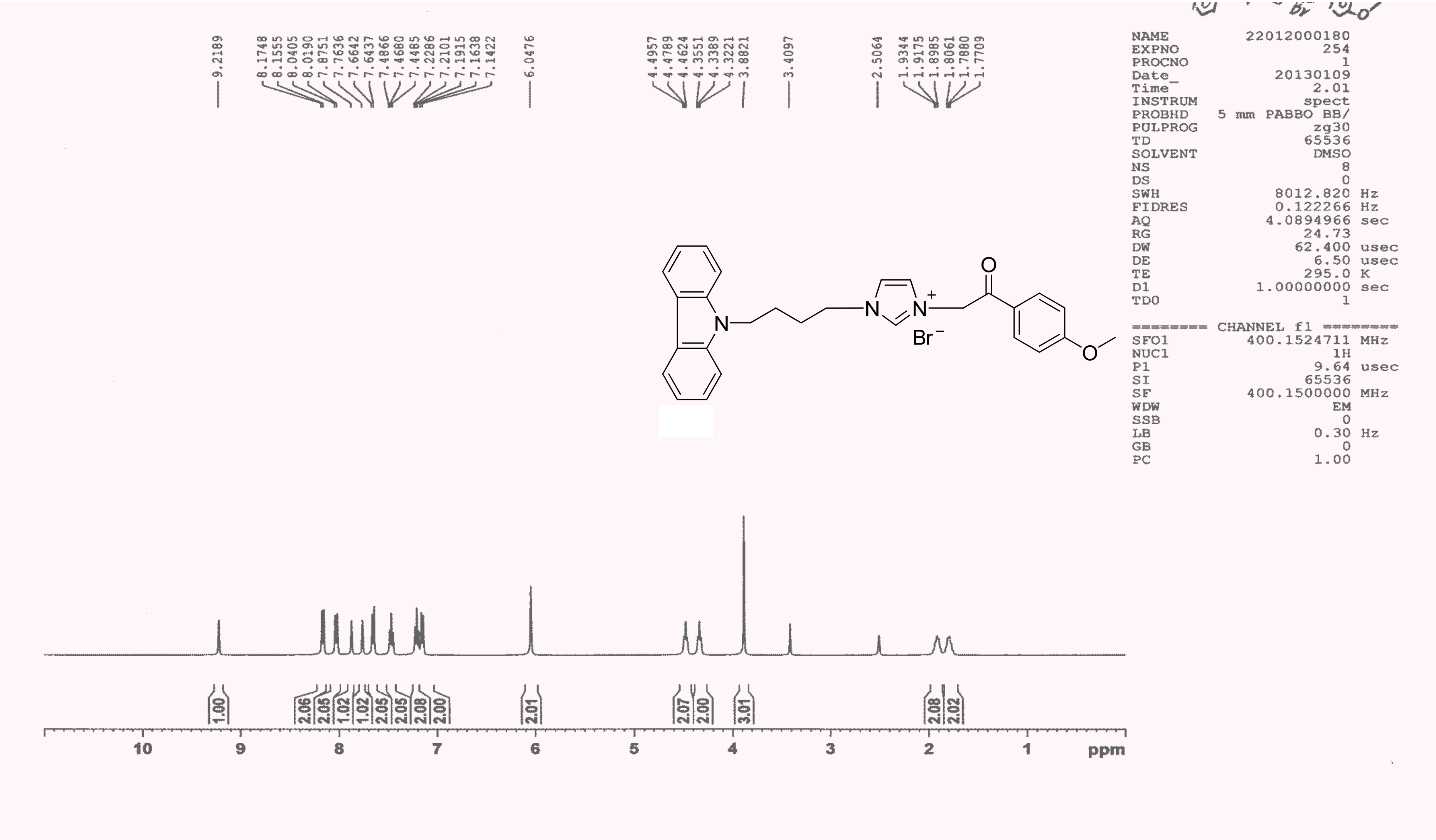
9.2189
8.1748
8.1555
8.0405
8.0190
7.8751
7.7636
7.6642
7.6437
7.4866
7.4680
7.4485
7.2286
7.2101
7.1915
7.1638
7.1422
6.0476
4.4957
4.4789
4.4624
4.3551
4.3389
4.3221
3.8821
3.4097
2.5064
1.9344
1.9175
1.8985
1.8061
1.7880
1.7709
N
N
N
+
Br−
O
O
NAME 22012000180
EXPNO 254
PROCNO 1
Date_ 20130109
Time 2.01
INSTRUM spect
PROBHD 5 mm PABBO BB/
PULPROG zg30
TD 65536
SOLVENT DMSO
NS 8
DS 0
SWH 8012.820 Hz
FIDRES 0.122266 Hz
AQ 4.0894966 sec
RG 24.73
DW 62.400 usec
DE 6.50 usec
TE 295.0 K
D1 1.00000000 sec
TD0 1
======== CHANNEL f1 ========
SFO1 400.1524711 MHz
NUC1 1H
P1 9.64 usec
SI 65536
SF 400.1500000 MHz
WDW EM
SSB 0
LB 0.30 Hz
GB 0
PC 1.00
1.00
2.06
2.05
1.02
1.02
2.05
2.05
2.08
2.00
2.01
2.07
2.00
3.01
2.08
2.02
10
9
8
7
6
5
4
3
2
1
ppm

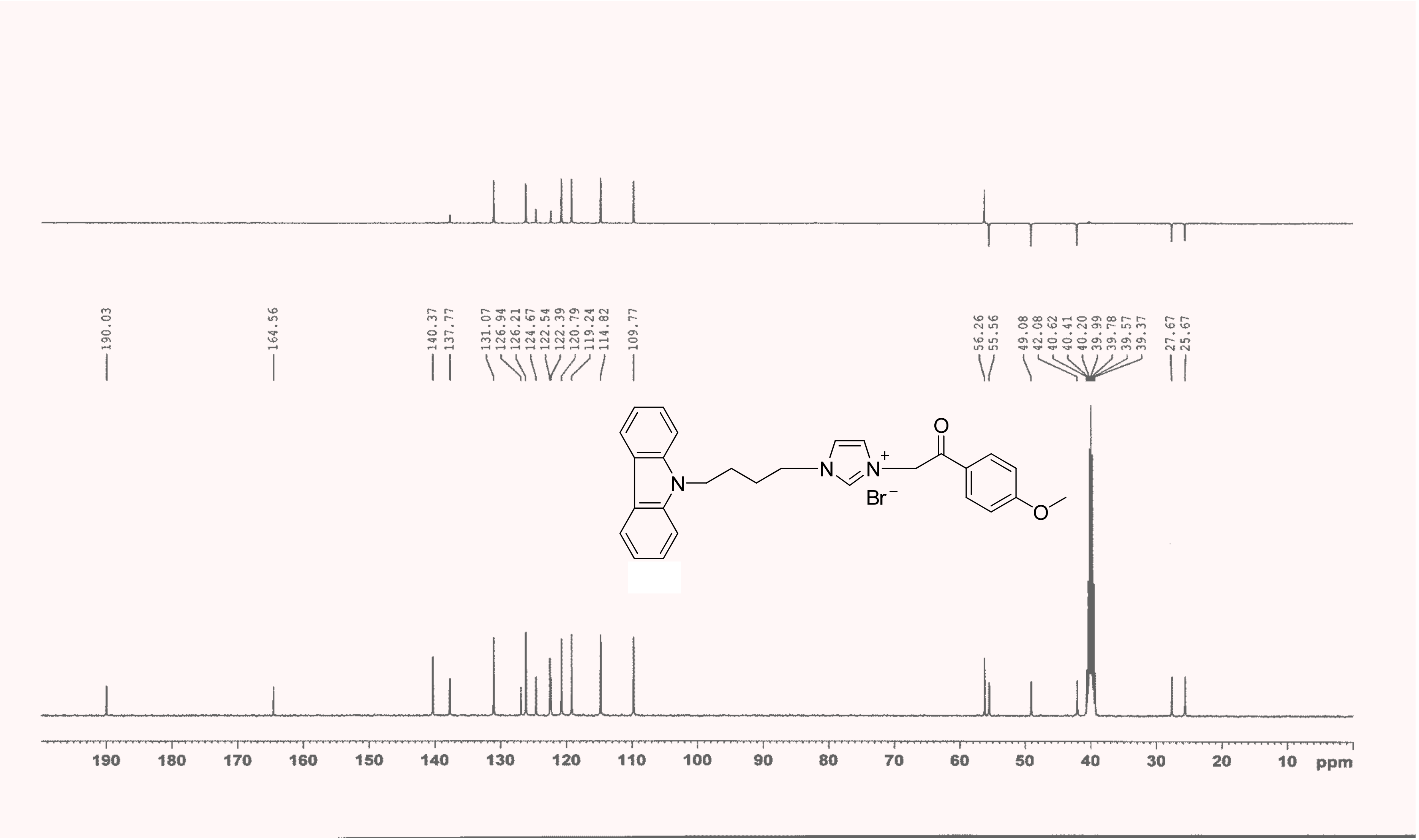

190.03
164.56
140.37
137.77
131.07
126.94
126.21
124.67
122.54
122.39
120.79
119.24
114.82
109.77
56.26
55.56
49.08
42.08
40.62
40.41
40.20
39.99
39.78
39.57
39.37
27.67
25.67
N
N
N+
Br−
O
O
190
180
170
160
150
140
130
120
110
100
90
80
70
60
50
40
30
20
10
ppm

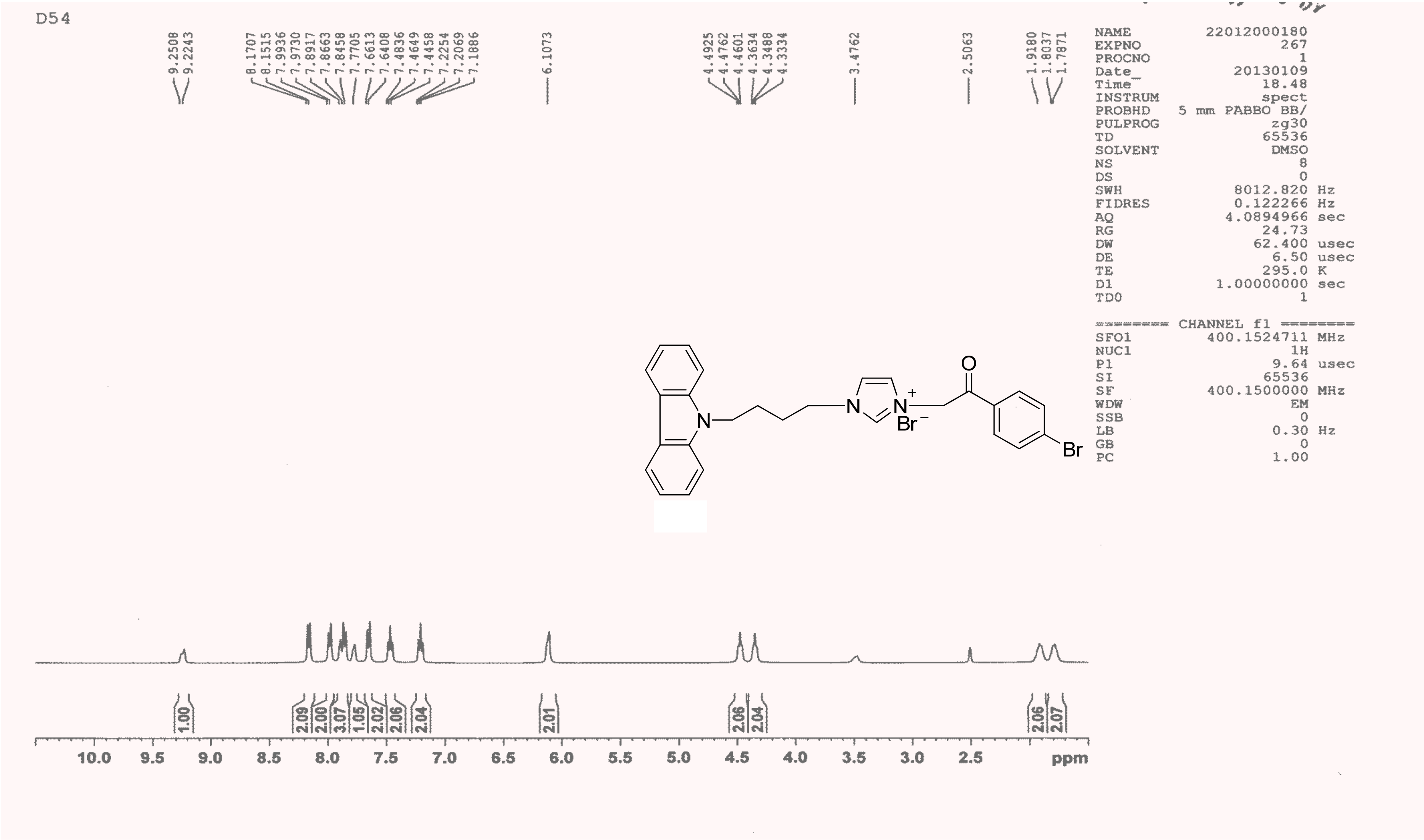
D54
9.2508
9.2243
8.1707
8.1515
7.9936
7.9730
7.8917
7.8663
7.8458
7.7705
7.6613
7.6408
7.4836
7.4649
7.4458
7.2254
7.2069
7.1886
6.1073
4.4925
4.4762
4.4601
4.3634
4.3488
4.3334
3.4762
2.5063
1.9180
1.8037
1.7871
NAME 22012000180
EXPNO 267
PROCNO 1
Date_ 20130109
Time 18.48
INSTRUM spect
PROBHD 5 mm PABBO BB/
PULPROG zg30
TD 65536
SOLVENT DMSO
NS 8
DS 0
SWH 8012.820 Hz
FIDRES 0.122266 Hz
AQ 4.0894966 sec
RG 24.73
DW 62.400 usec
DE 6.50 usec
TE 295.0 K
D1 1.00000000 sec
TD0 1
======== CHANNEL f1 ========
SFO1 400.1524711 MHz
NUC1 1H
P1 9.64 usec
SI 65536
SF 400.1500000 MHz
WDW EM
SSB 0
LB 0.30 Hz
GB 0
PC 1.00
N
N
N
+
Br
−
O
Br
1.00
2.09
2.00
3.07
1.05
2.02
2.06
2.04
2.01
2.06
2.04
2.06
2.07
10.0
9.5
9.0
8.5
8.0
7.5
7.0
6.5
6.0
5.5
5.0
4.5
4.0
3.5
3.0
2.5
ppm

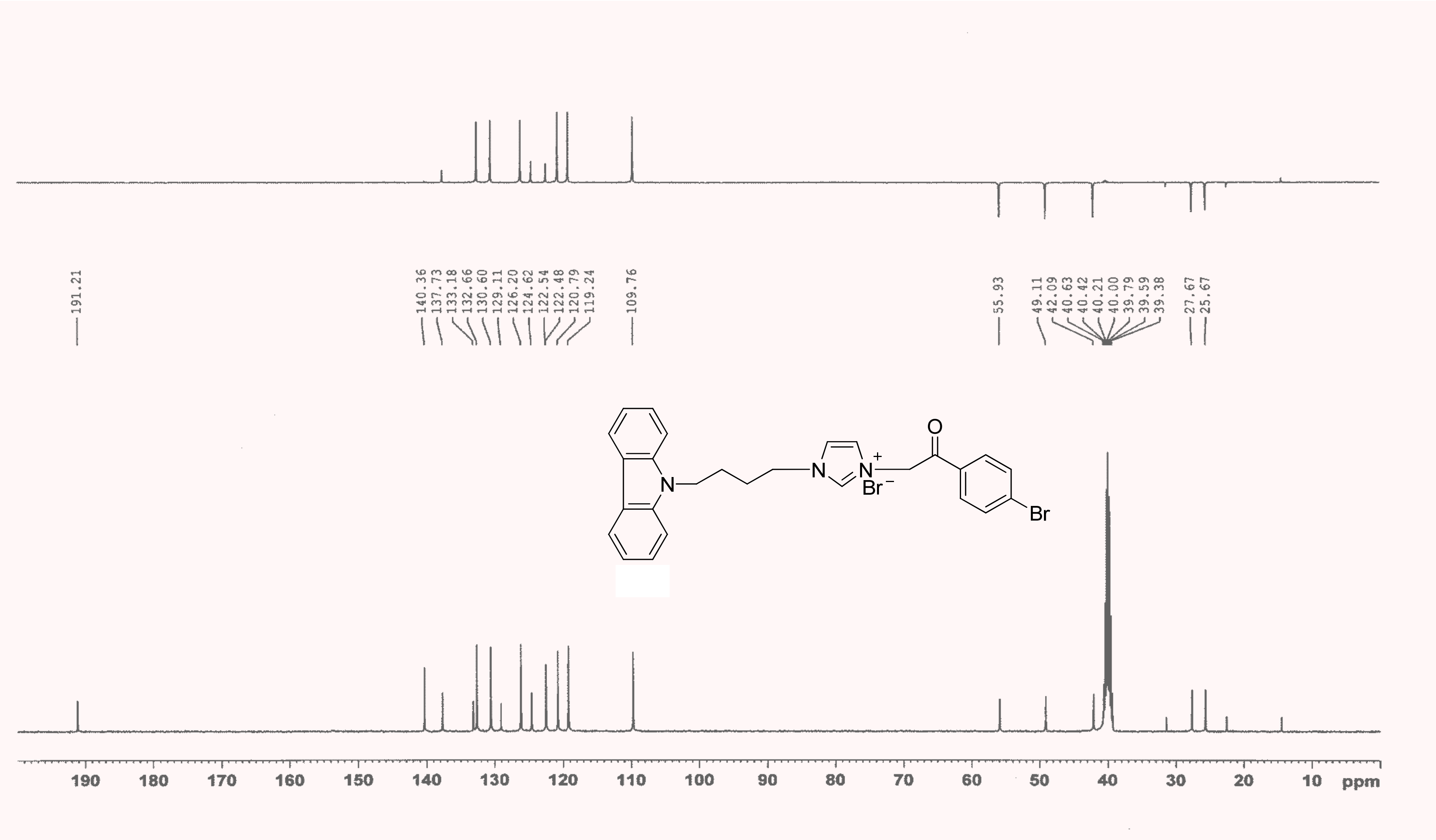
191.21
140.36
137.73
133.18
132.66
130.60
129.11
126.20
124.62
122.54
122.48
120.79
119.24
109.76
55.93
49.11
42.09
40.63
40.42
40.21
40.00
39.79
39.59
39.38
27.67
25.67
N
N
N+
Br−
O
Br
190
180
170
160
150
140
130
120
110
100
90
80
70
60
50
40
30
20
10
ppm

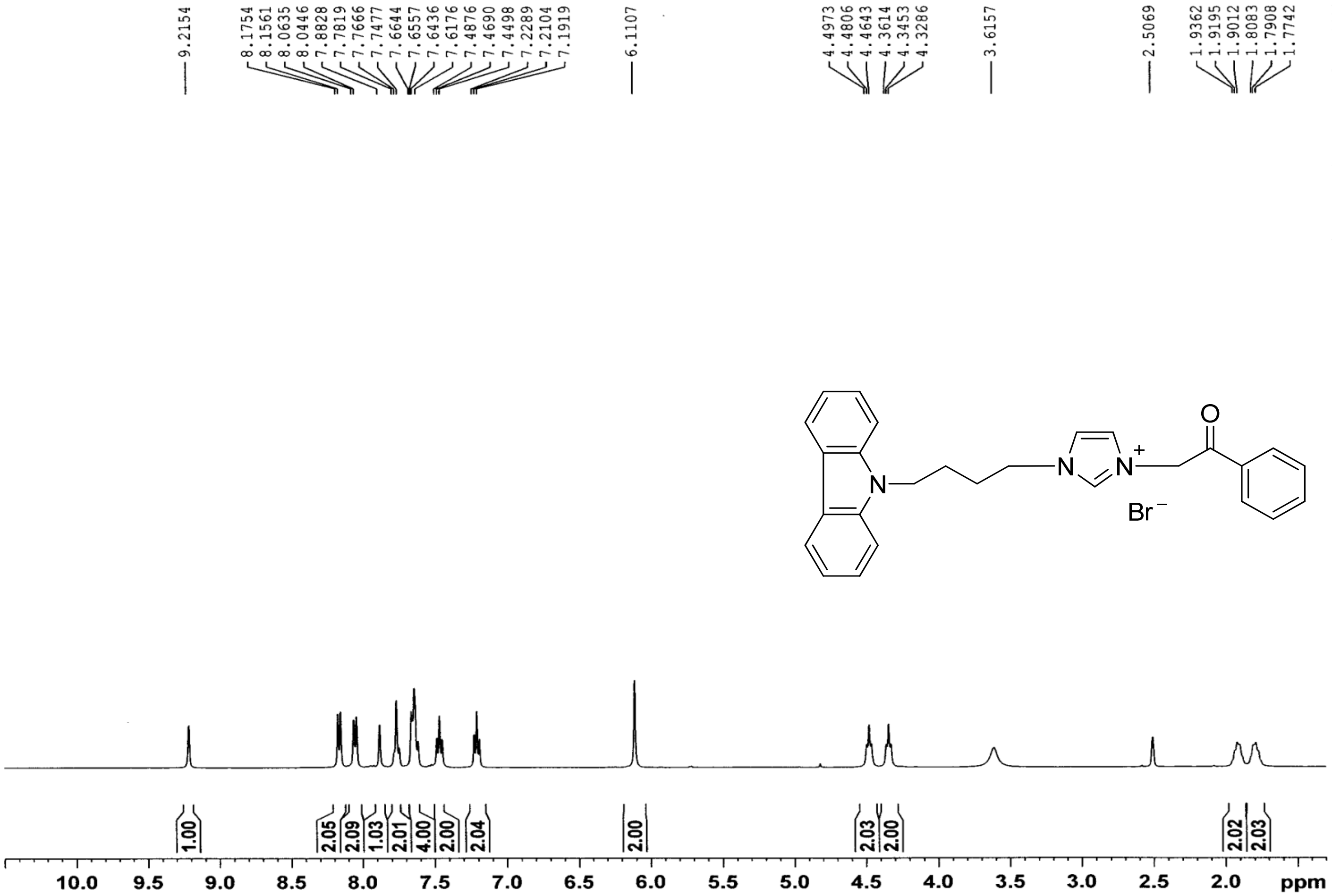

NAME	22012000180	
EXPNO	273	
PROCNO	1	
Date_	20130109	
Time_	20.25	
INSTRUM	spect	
PROBHD	5 mm PABBO BB/	
PULPROG	zg30	
TD	65536	
SOLVENT	DMSO	
NS	8	
DS	0	
SWH	8012.820	Hz
FIDRES	0.122266	Hz
AQ	4.0894966	sec
RG	24.73	
DW	62.400	usec
DE	6.50	usec
TE	295.0	K
D1	1.00000000	sec
TD0	1	
======== CHANNEL f1 ========		
SFO1	400.1524711	MHz
NUC1	1H	
P1	9.64	usec
SI	65536	
SF	400.1500000	MHz
WDW	EM	
SSB	0	
LB	0.30	Hz
GB	0	
PC	1.00	

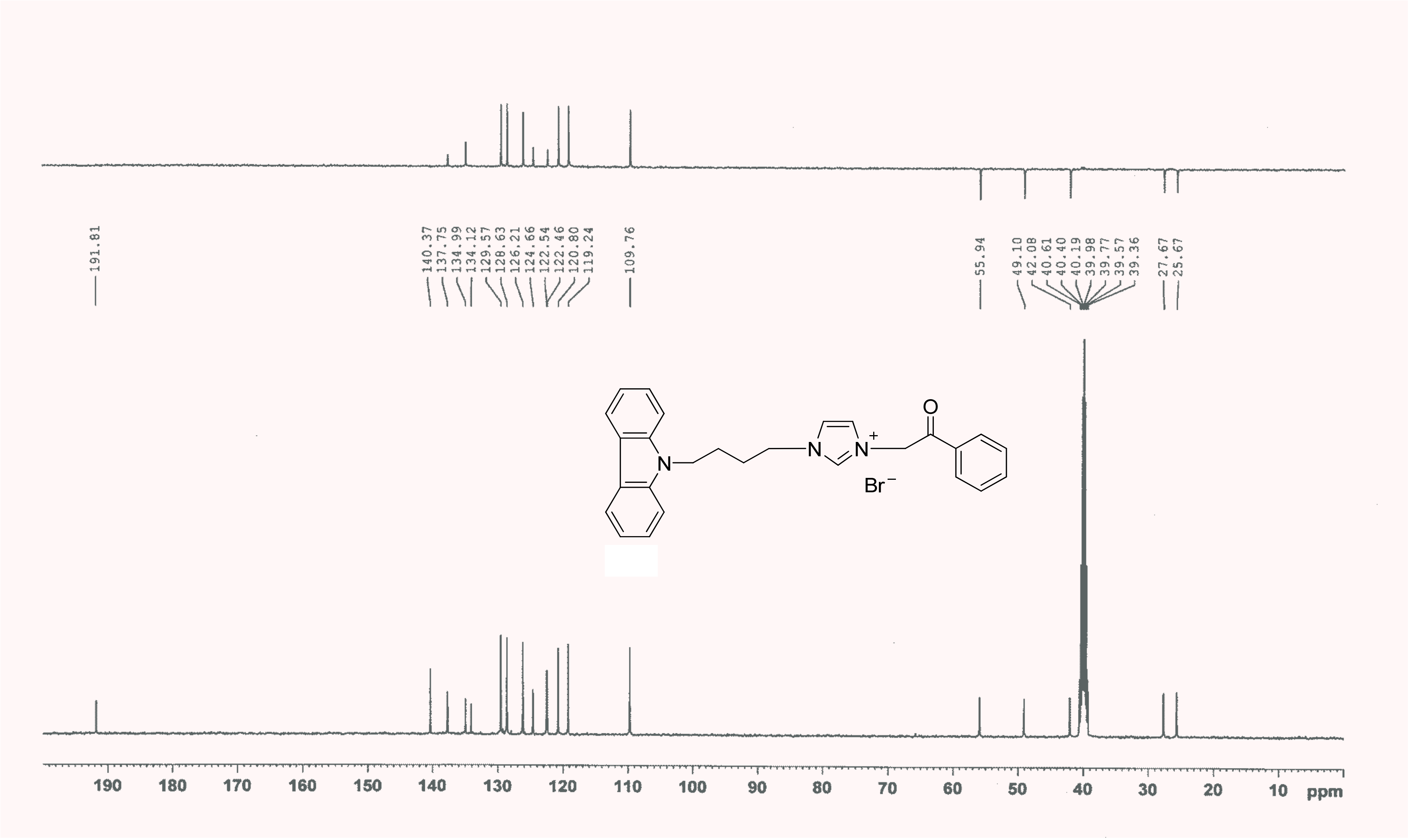
191.81
140.37
137.75
134.99
134.12
129.57
128.63
126.21
124.66
122.54
122.46
120.80
119.24
109.76
55.94
49.10
42.08
40.61
40.40
40.19
39.98
39.77
39.57
39.36
27.67
25.67
N
N
N
O
Br
190
180
170
160
150
140
130
120
110
100
90
80
70
60
50
40
30
20
10
ppm

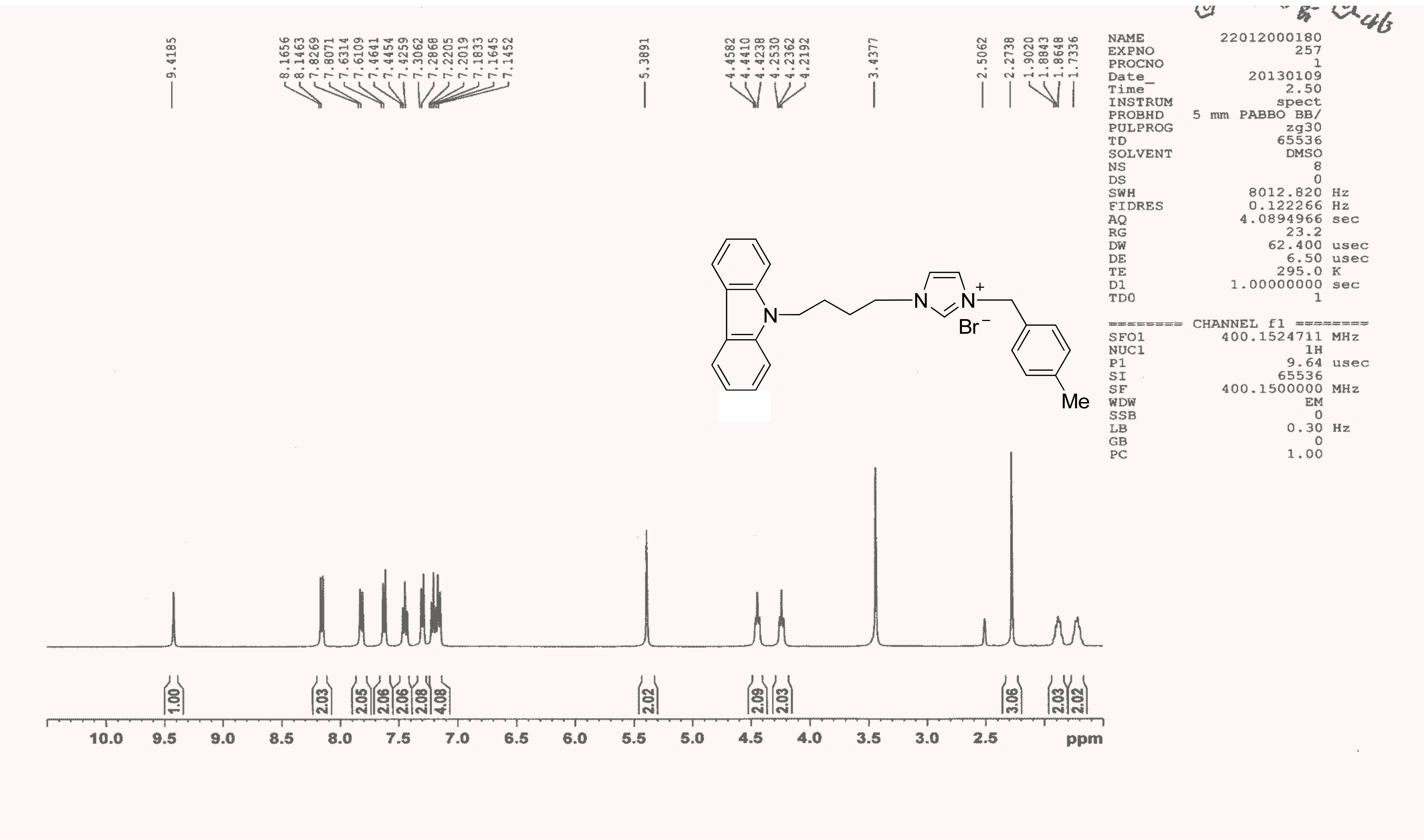
9.4185
8.1656
8.1463
7.8269
7.8071
7.6314
7.6109
7.4641
7.4454
7.4259
7.3062
7.2868
7.2205
7.2019
7.1833
7.1645
7.1452
5.3891
4.4582
4.4410
4.4238
4.2530
4.2362
4.2192
3.4377
2.5062
2.2738
1.9020
1.8843
1.8648
1.7336
N
N
N
Br⁻
Me
1.00
2.03
2.05
2.06
2.06
2.08
4.08
2.02
2.09
2.03
3.06
2.03
2.02
10.0 9.5 9.0 8.5 8.0 7.5 7.0 6.5 6.0 5.5 5.0 4.5 4.0 3.5 3.0 2.5 ppm
NAME 22012000180
EXPNO 257
PROCNO 1
Date_ 20130109
Time 2.50
INSTRUM spect
PROBHD 5 mm PABBO BB/
PULPROG zg30
TD 65536
SOLVENT DMSO
NS 8
DS 0
SWH 8012.820 Hz
FIDRES 0.122266 Hz
AQ 4.0894966 sec
RG 23.2
DW 62.400 usec
DE 6.50 usec
TE 295.0 K
D1 1.00000000 sec
TD0 1
======== CHANNEL f1 ========
SFO1 400.1524711 MHz
NUC1 1H
P1 9.64 usec
SI 65536
SF 400.1500000 MHz
WDW EM
SSB 0
LB 0.30 Hz
GB 0
PC 1.00

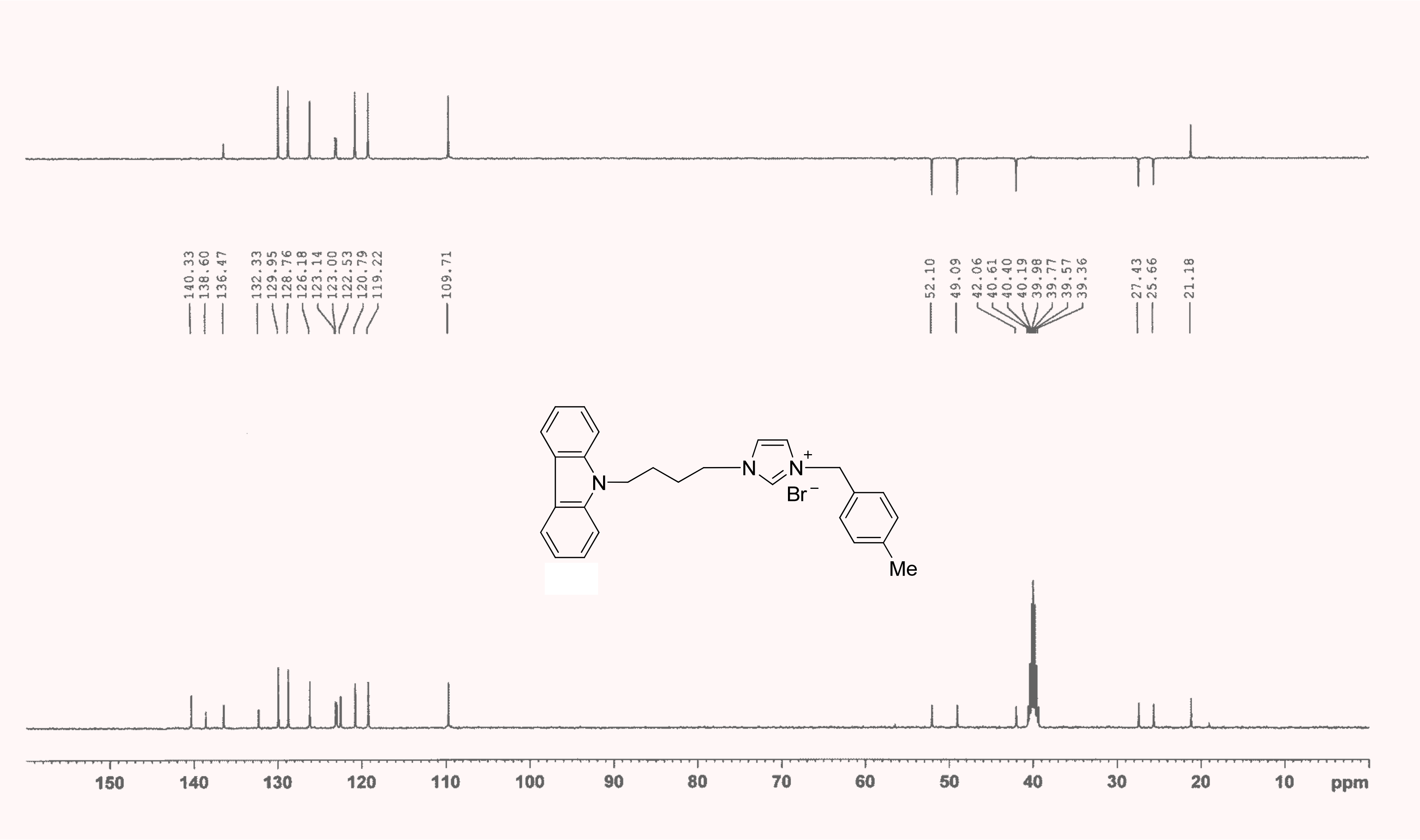
140.33
138.60
136.47
132.33
129.95
128.76
126.18
123.14
123.00
122.53
120.79
119.22
109.71
52.10
49.09
42.06
40.61
40.40
40.19
39.98
39.77
39.57
39.36
27.43
25.66
21.18
N
N
N+
Br−
Me
150
140
130
120
110
100
90
80
70
60
50
40
30
20
10
ppm

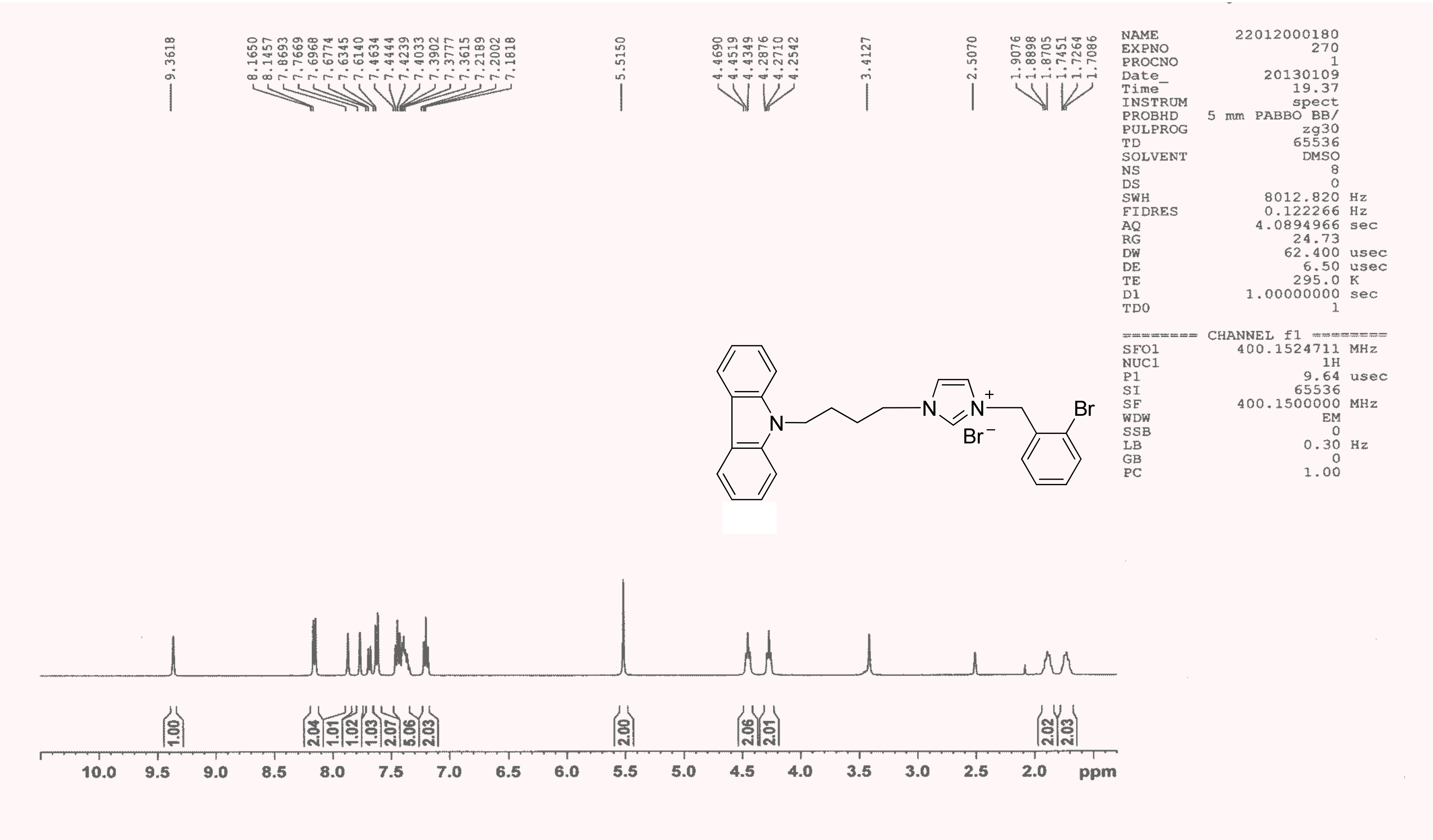
9.3618
8.1650
8.1457
7.8693
7.7669
7.6968
7.6774
7.6345
7.6140
7.4634
7.4444
7.4239
7.4033
7.3902
7.3777
7.3615
7.2189
7.2002
7.1818
5.5150
4.4690
4.4519
4.4349
4.2876
4.2710
4.2542
3.4127
2.5070
1.9076
1.8898
1.8705
1.7451
1.7264
1.7086
NAME 22012000180
EXPNO 270
PROCNO 1
Date_ 20130109
Time 19.37
INSTRUM spect
PROBHD 5 mm PABBO BB/
PULPROG zg30
TD 65536
SOLVENT DMSO
NS 8
DS 0
SWH 8012.820 Hz
FIDRES 0.122266 Hz
AQ 4.0894966 sec
RG 24.73
DW 62.400 usec
DE 6.50 usec
TE 295.0 K
D1 1.00000000 sec
TD0 1
======== CHANNEL f1 ========
SFO1 400.1524711 MHz
NUC1 1H
P1 9.64 usec
SI 65536
SF 400.1500000 MHz
WDW EM
SSB 0
LB 0.30 Hz
GB 0
PC 1.00
N
N
N
+
Br
Br
−
1.00
2.04
1.01
1.02
1.03
2.07
5.06
2.03
2.00
2.06
2.01
2.02
2.03
10.0
9.5
9.0
8.5
8.0
7.5
7.0
6.5
6.0
5.5
5.0
4.5
4.0
3.5
3.0
2.5
2.0
ppm

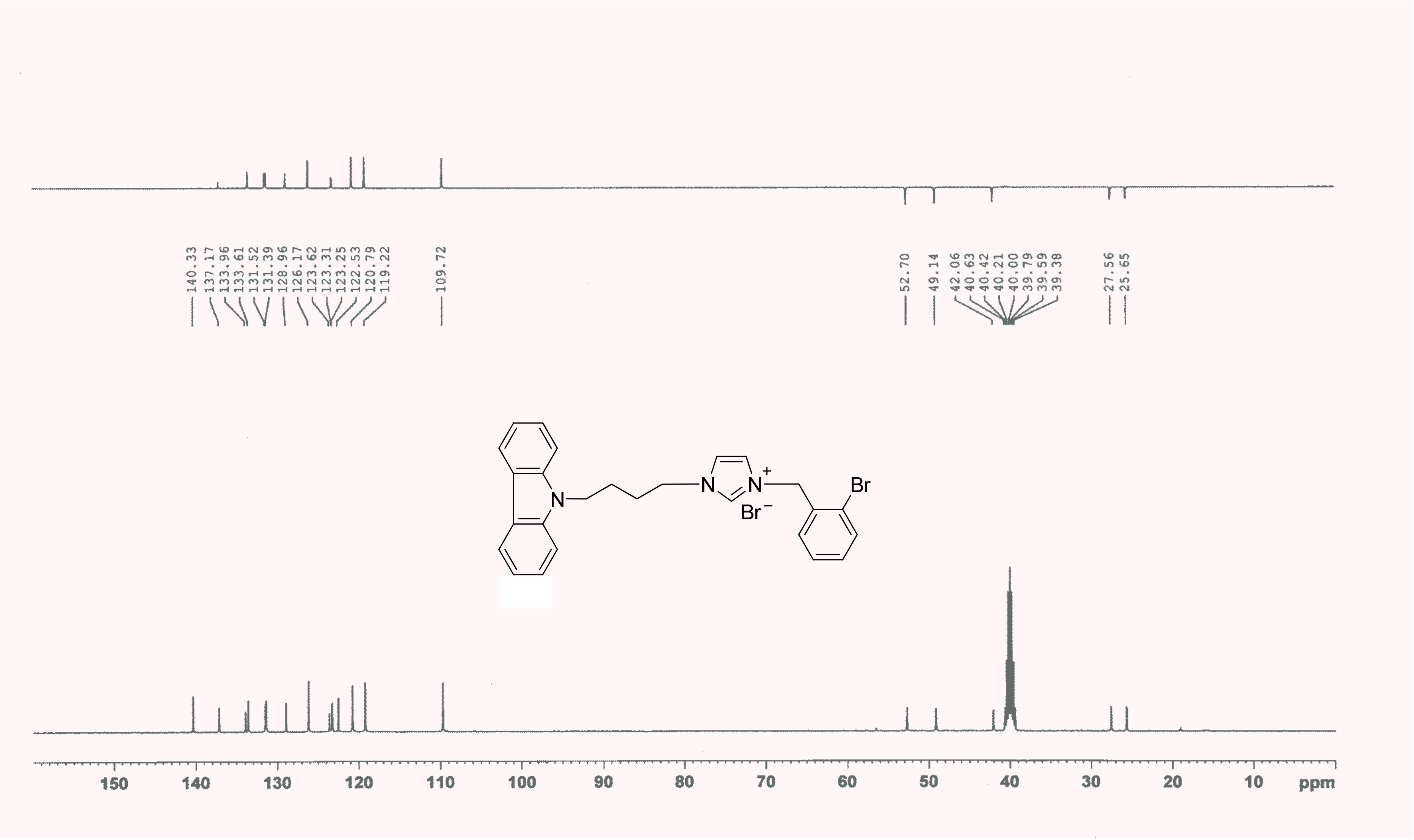
140.33
137.17
133.96
133.61
131.52
131.39
128.96
126.17
123.62
123.31
123.25
122.53
120.79
119.22
109.72
52.70
49.14
42.06
40.63
40.42
40.21
40.00
39.79
39.59
39.38
27.56
25.65
N
N
N+
Br
Br−
150
140
130
120
110
100
90
80
70
60
50
40
30
20
10
ppm

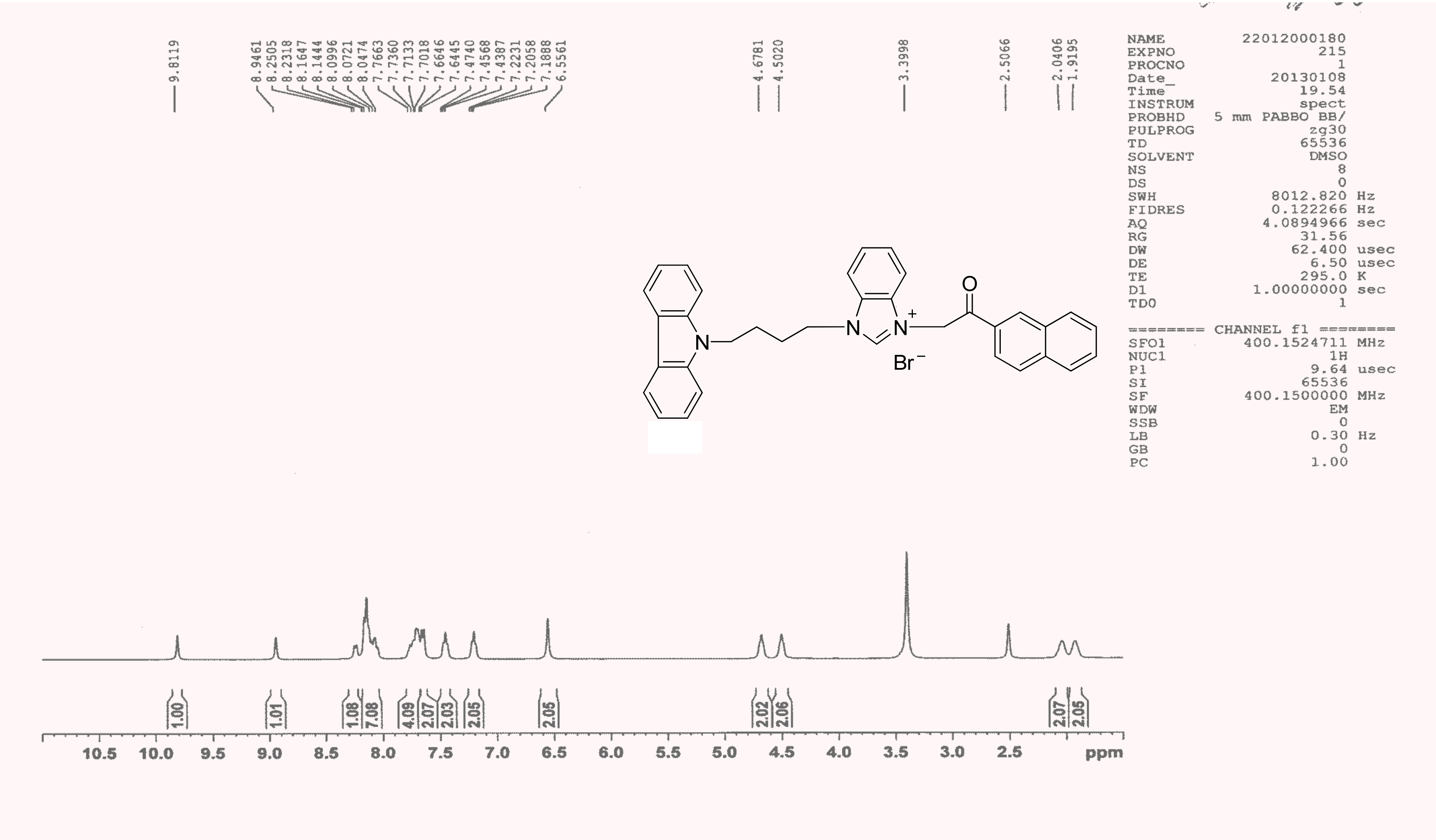

9.8119
8.9461
8.2505
8.2318
8.1647
8.1444
8.0996
8.0721
8.0474
7.7663
7.7360
7.7133
7.7018
7.6646
7.6445
7.4740
7.4568
7.4387
7.2231
7.2058
7.1888
6.5561
4.6781
4.5020
3.3998
2.5066
2.0406
1.9195
N
N
N+
O
Br−
NAME 22012000180
EXPNO 215
PROCNO 1
Date_ 20130108
Time 19.54
INSTRUM spect
PROBHD 5 mm PABBO BB/
PULPROG zg30
TD 65536
SOLVENT DMSO
NS 8
DS 0
SWH 8012.820 Hz
FIDRES 0.122266 Hz
AQ 4.0894966 sec
RG 31.56
DW 62.400 usec
DE 6.50 usec
TE 295.0 K
D1 1.00000000 sec
TD0 1
======== CHANNEL f1 ========
SFO1 400.1524711 MHz
NUC1 1H
P1 9.64 usec
SI 65536
SF 400.1500000 MHz
WDW EM
SSB 0
LB 0.30 Hz
GB 0
PC 1.00
1.00
1.01
1.08
7.08
4.09
2.07
2.03
2.05
2.05
2.02
2.06
2.07
2.05
10.5 10.0 9.5 9.0 8.5 8.0 7.5 7.0 6.5 6.0 5.5 5.0 4.5 4.0 3.5 3.0 2.5 ppm

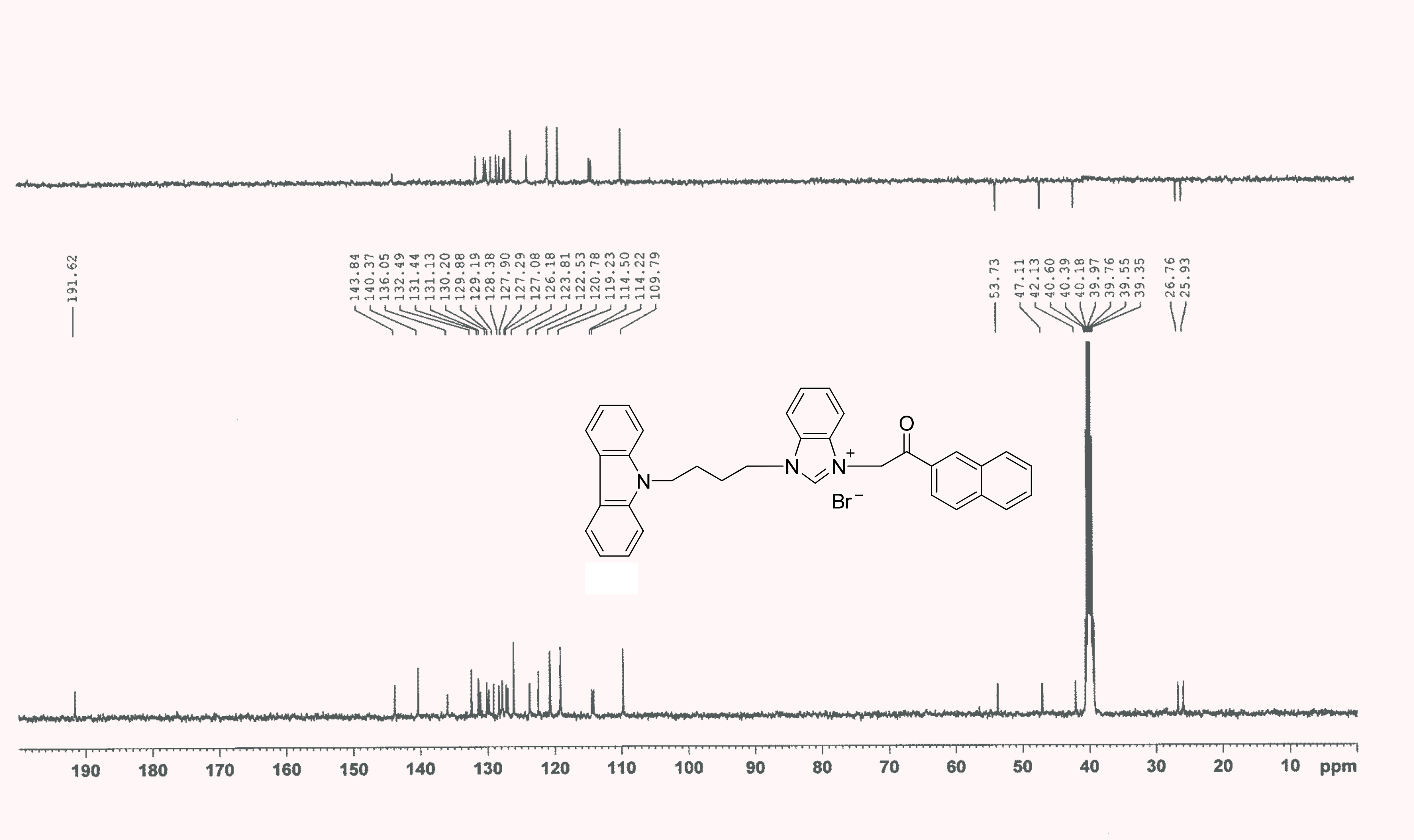
191.62
143.84
140.37
136.05
132.49
131.44
131.13
130.20
129.88
129.19
128.38
127.90
127.29
127.08
126.18
123.81
122.53
120.78
119.23
114.50
114.22
109.79
53.73
47.11
42.13
40.60
40.39
40.18
39.97
39.76
39.55
39.35
26.76
25.93
N
N
N+
O
Br−
190
180
170
160
150
140
130
120
110
100
90
80
70
60
50
40
30
20
10
ppm

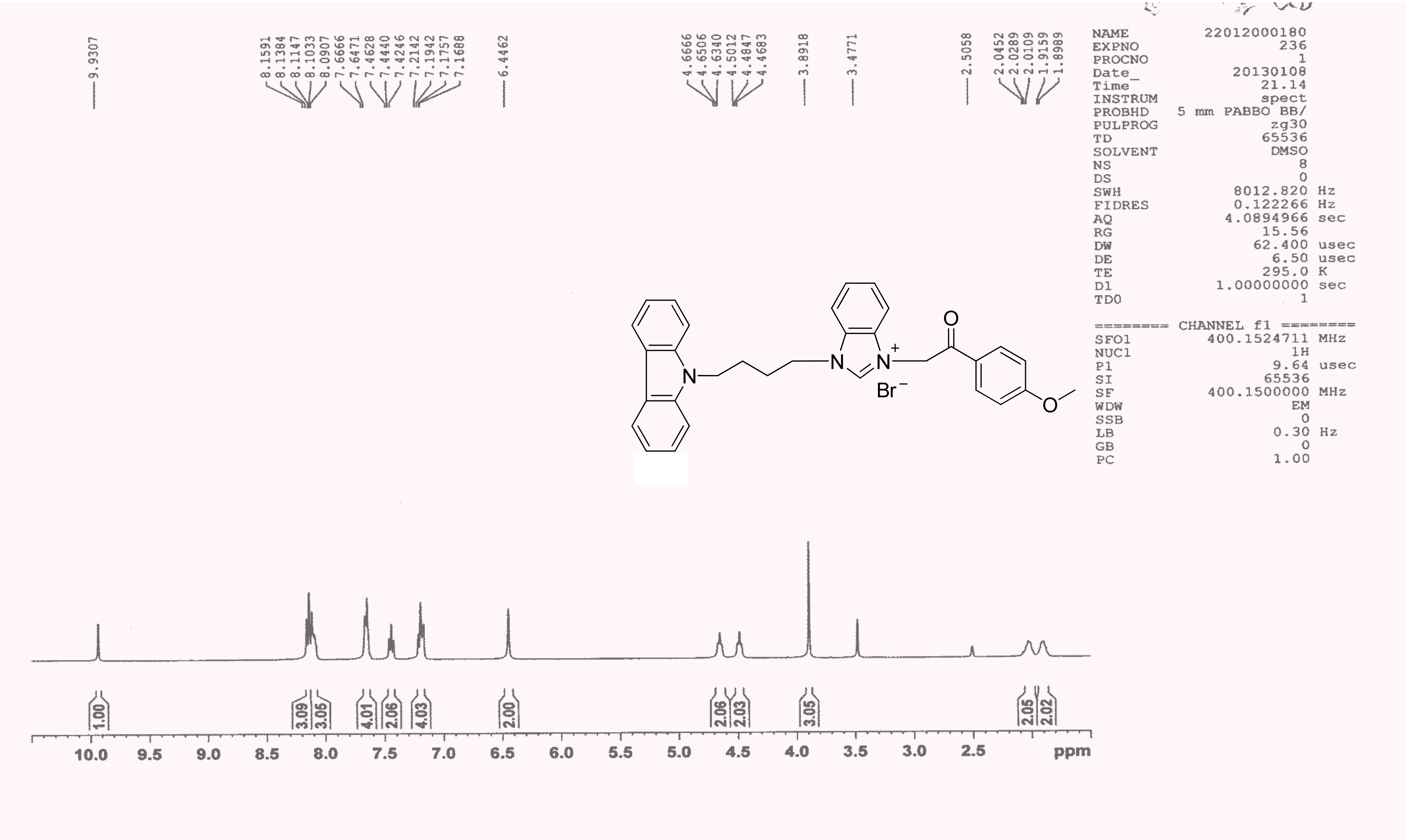

9.9307
8.1591 8.1384 8.1147 8.1033 8.0907 7.6666 7.6471 7.4628 7.4440 7.4246 7.2142 7.1942 7.1757 7.1688
6.4462
4.6666 4.6506 4.6340 4.5012 4.4847 4.4683
3.8918
3.4771
2.5058
2.0452 2.0289 2.0109 1.9159 1.8989
NAME 22012000180
EXPNO 236
PROCNO 1
Date_ 20130108
Time 21.14
INSTRUM spect
PROBHD 5 mm PABBO BB/
PULPROG zg30
TD 65536
SOLVENT DMSO
NS 8
DS 0
SWH 8012.820 Hz
FIDRES 0.122266 Hz
AQ 4.0894966 sec
RG 15.56
DW 62.400 usec
DE 6.50 usec
TE 295.0 K
D1 1.00000000 sec
TD0 1
======== CHANNEL f1 ========
SFO1 400.1524711 MHz
NUC1 1H
P1 9.64 usec
SI 65536
SF 400.1500000 MHz
WDW EM
SSB 0
LB 0.30 Hz
GB 0
PC 1.00
N
N
N+
Br−
O
O
1.00 3.09 3.05 4.01 2.06 4.03 2.00 2.06 2.03 3.05 2.05 2.02
10.0 9.5 9.0 8.5 8.0 7.5 7.0 6.5 6.0 5.5 5.0 4.5 4.0 3.5 3.0 2.5 ppm

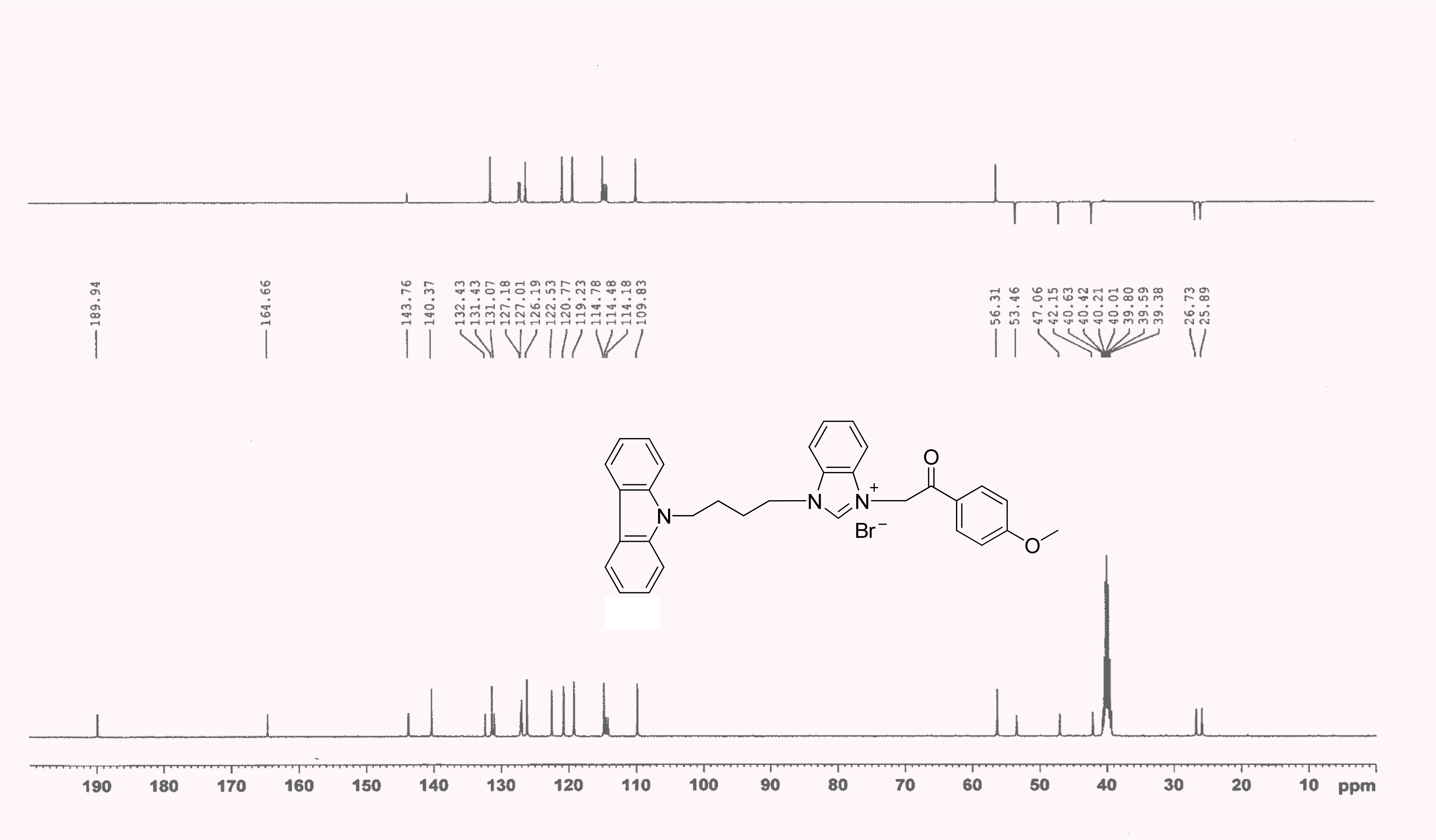
189.94
164.66
143.76
140.37
132.43
131.43
131.07
127.18
127.01
126.19
122.53
120.77
119.23
114.78
114.48
114.18
109.83
56.31
53.46
47.06
42.15
40.63
40.42
40.21
40.01
39.80
39.59
39.38
26.73
25.89
N
N
N
+
O
O
Br−
190 180 170 160 150 140 130 120 110 100 90 80 70 60 50 40 30 20 10 ppm

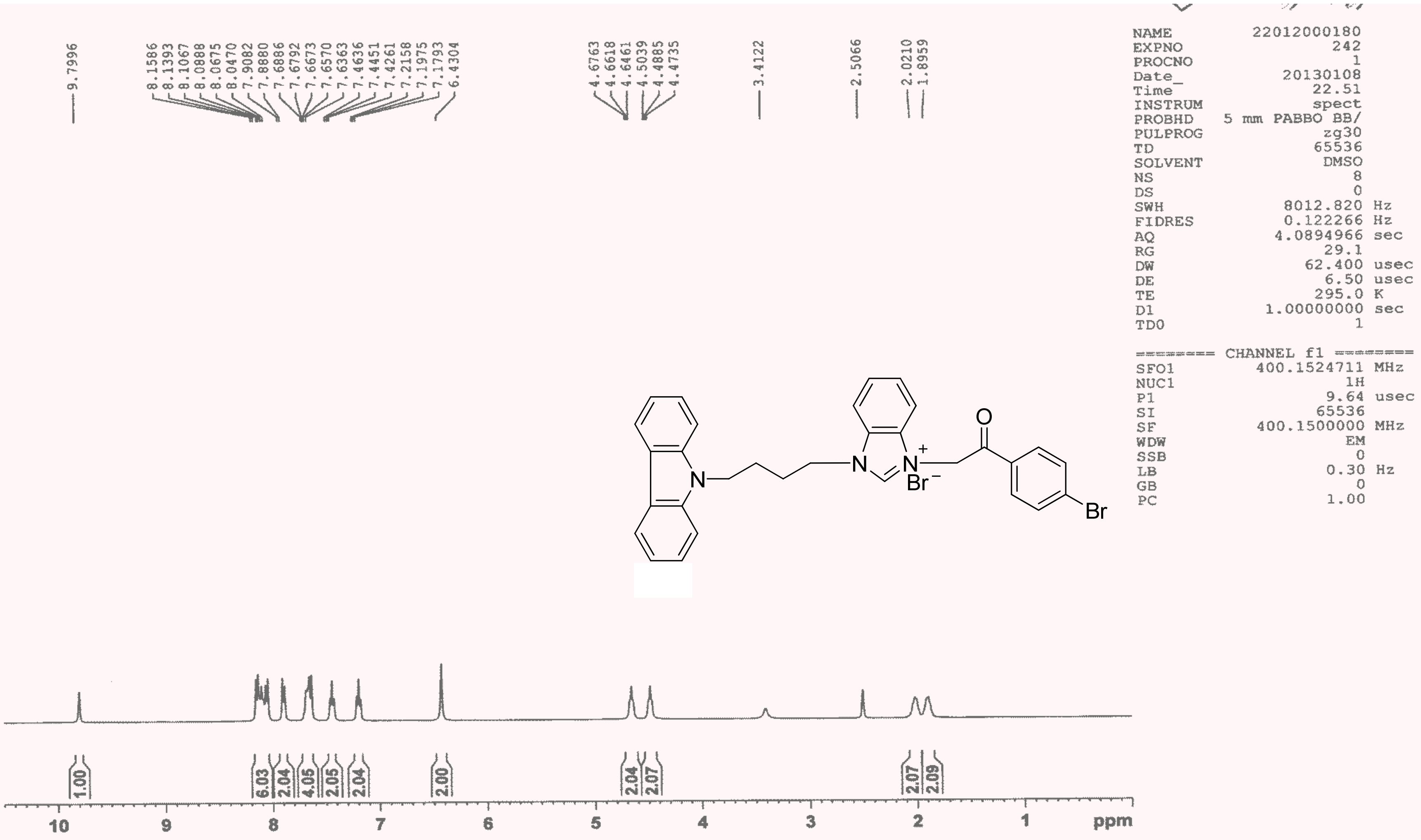
9.7996
8.1586 8.1393 8.1067 8.0888 8.0675 8.0470 7.9082 7.8880 7.6886 7.6792 7.6673 7.6570 7.6363 7.4636 7.4451 7.4261 7.2158 7.1975 7.1793 6.4304
4.6763 4.6618 4.6461 4.5039 4.4885 4.4735
3.4122
2.5066
2.0210 1.8959
NAME 22012000180
EXPNO 242
PROCNO 1
Date_ 20130108
Time 22.51
INSTRUM spect
PROBHD 5 mm PABBO BB/
PULPROG zg30
TD 65536
SOLVENT DMSO
NS 8
DS 0
SWH 8012.820 Hz
FIDRES 0.122266 Hz
AQ 4.0894966 sec
RG 29.1
DW 62.400 usec
DE 6.50 usec
TE 295.0 K
D1 1.00000000 sec
TD0 1
======== CHANNEL f1 ========
SFO1 400.1524711 MHz
NUC1 1H
P1 9.64 usec
SI 65536
SF 400.1500000 MHz
WDW EM
SSB 0
LB 0.30 Hz
GB 0
PC 1.00
N
N
N
O
Br
Br
1.00 6.03 2.04 4.05 2.05 2.04 2.00 2.04 2.07 2.07 2.09
10 9 8 7 6 5 4 3 2 1 ppm

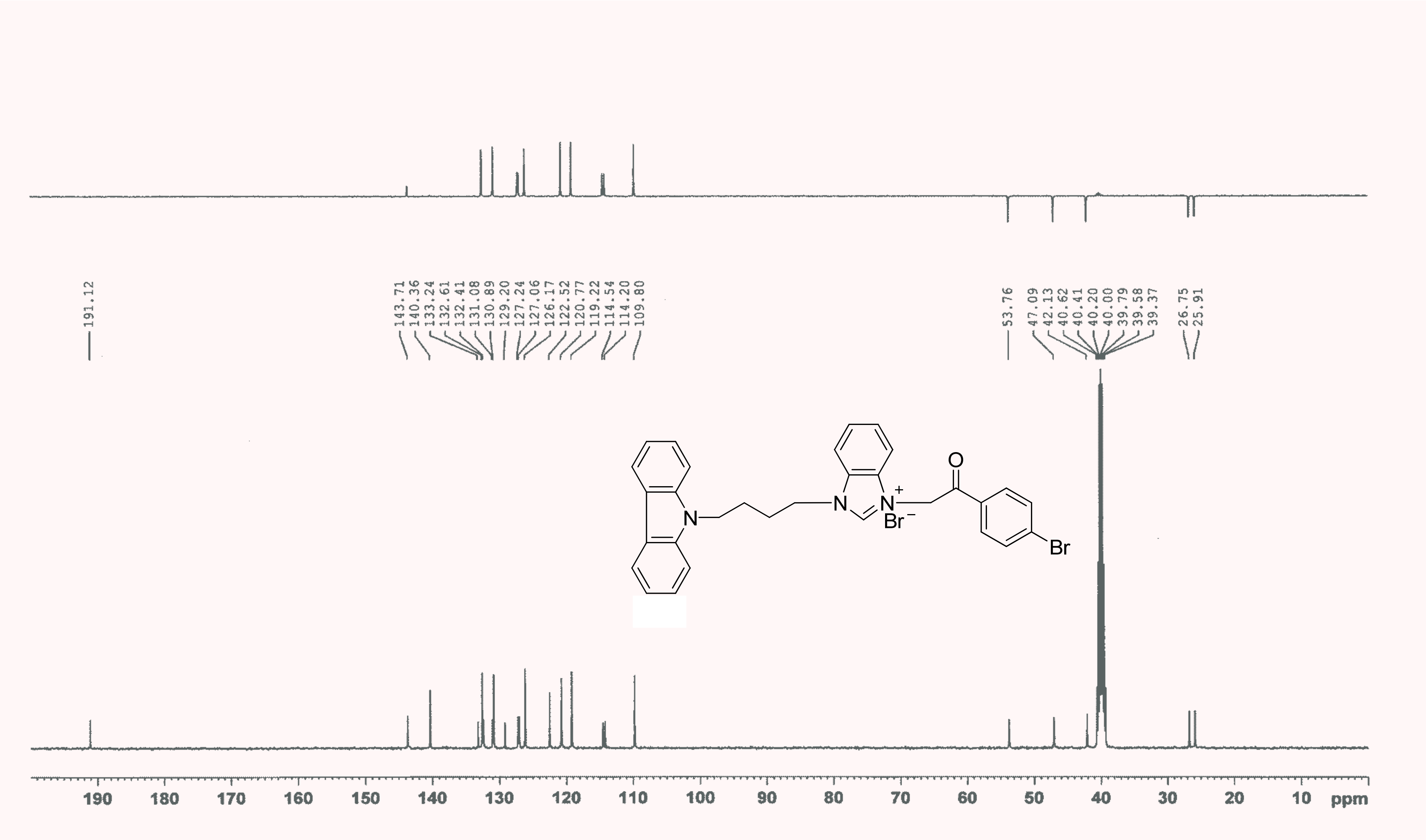

191.12
143.71
140.36
133.24
132.61
132.41
131.08
130.89
129.20
127.24
127.06
126.17
122.52
120.77
119.22
114.54
114.20
109.80
53.76
47.09
42.13
40.62
40.41
40.20
40.00
39.79
39.58
39.37
26.75
25.91
N
N
N
+
Br
−
O
Br
190 180 170 160 150 140 130 120 110 100 90 80 70 60 50 40 30 20 10 ppm

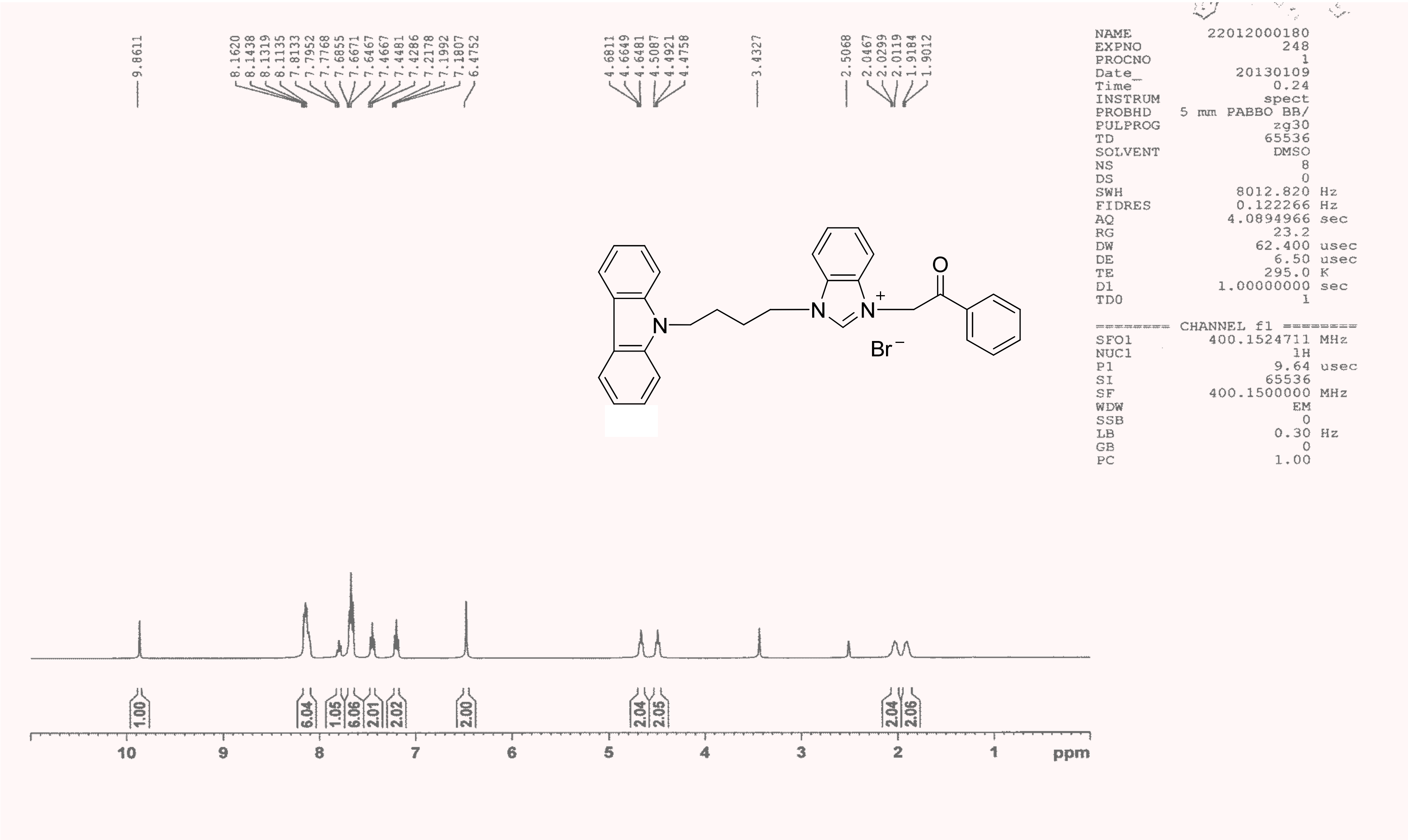

9.8611
8.1620 8.1438 8.1319 8.1135 7.8133 7.7952 7.7768 7.6855 7.6671 7.6467 7.4667 7.4481 7.4286 7.2178 7.1992 7.1807 6.4752
4.6811 4.6649 4.6481 4.5087 4.4921 4.4758
3.4327
2.5068 2.0467 2.0299 2.0119 1.9184 1.9012
NAME 22012000180
EXPNO 248
PROCNO 1
Date_ 20130109
Time 0.24
INSTRUM spect
PROBHD 5 mm PABBO BB/
PULPROG zg30
TD 65536
SOLVENT DMSO
NS 8
DS 0
SWH 8012.820 Hz
FIDRES 0.122266 Hz
AQ 4.0894966 sec
RG 23.2
DW 62.400 usec
DE 6.50 usec
TE 295.0 K
D1 1.00000000 sec
TD0 1
======== CHANNEL f1 ========
SFO1 400.1524711 MHz
NUC1 1H
P1 9.64 usec
SI 65536
SF 400.1500000 MHz
WDW EM
SSB 0
LB 0.30 Hz
GB 0
PC 1.00
N
N
N+
O
Br−
1.00 6.04 1.05 6.06 2.01 2.02 2.00 2.04 2.05 2.04 2.06
10 9 8 7 6 5 4 3 2 1 ppm

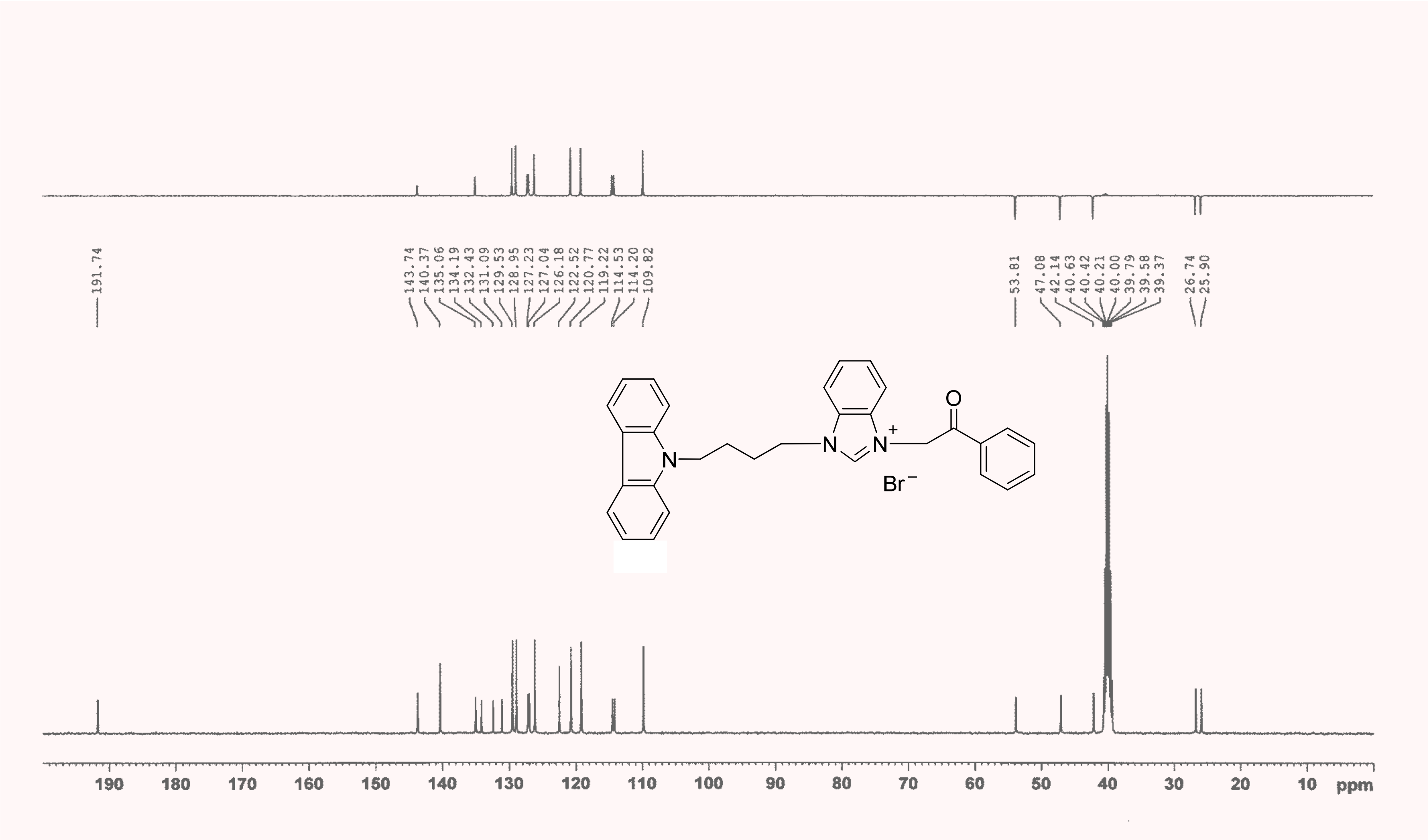
191.74
143.74
140.37
135.06
134.19
132.43
131.09
129.53
128.95
127.23
127.04
126.18
122.52
120.77
119.22
114.53
114.20
109.82
53.81
47.08
42.14
40.63
40.42
40.21
40.00
39.79
39.58
39.37
26.74
25.90
N
N
N+
O
Br−
190 180 170 160 150 140 130 120 110 100 90 80 70 60 50 40 30 20 10 ppm

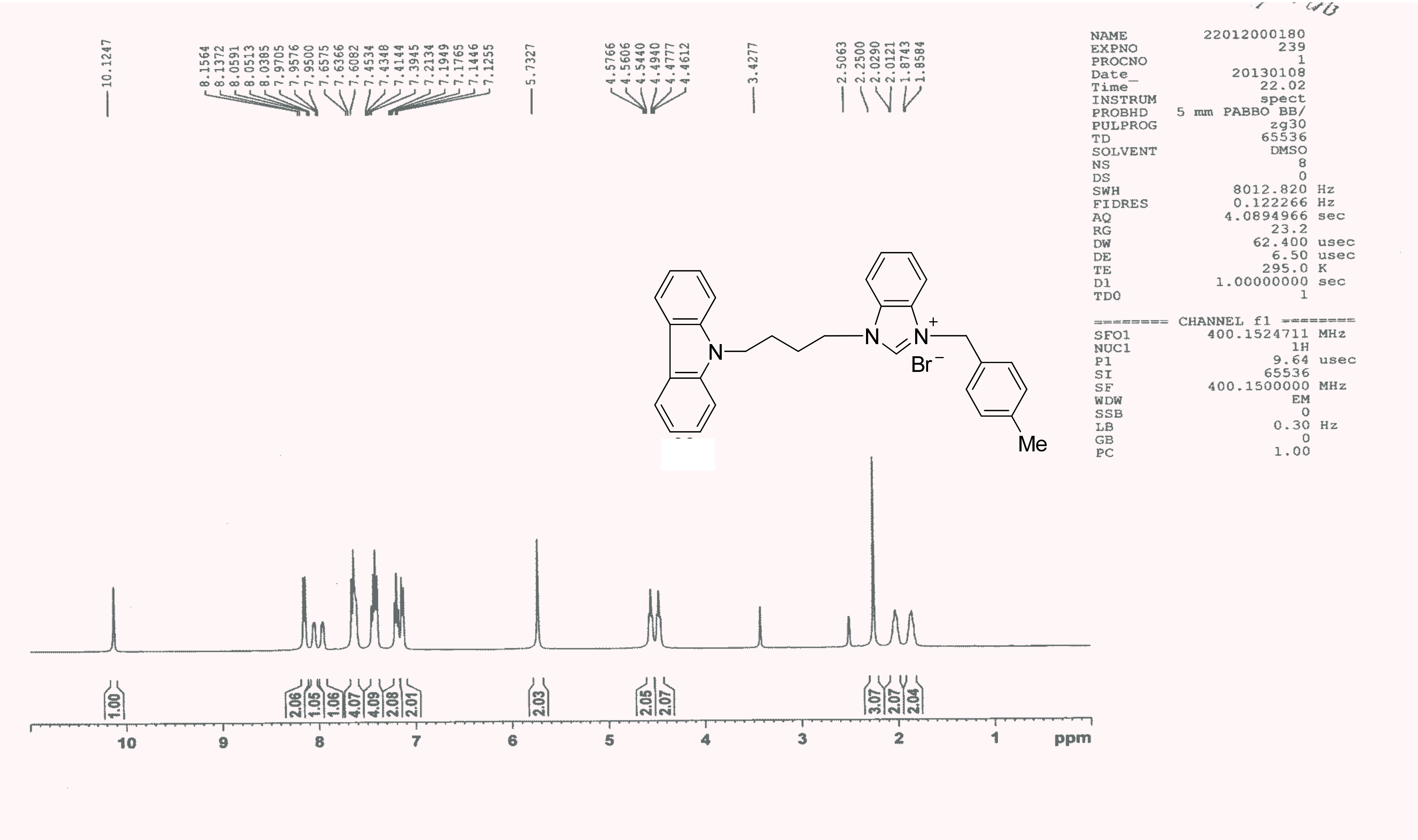
10.1247
8.1564
8.1372
8.0591
8.0513
8.0385
7.9705
7.9576
7.9500
7.6575
7.6366
7.6082
7.4534
7.4348
7.4144
7.3945
7.2134
7.1949
7.1765
7.1446
7.1255
5.7327
4.5766
4.5606
4.5440
4.4940
4.4777
4.4612
3.4277
2.5063
2.2500
2.0290
2.0121
1.8743
1.8584
NAME 22012000180
EXPNO 239
PROCNO 1
Date_ 20130108
Time 22.02
INSTRUM spect
PROBHD 5 mm PABBO BB/
PULPROG zg30
TD 65536
SOLVENT DMSO
NS 8
DS 0
SWH 8012.820 Hz
FIDRES 0.122266 Hz
AQ 4.0894966 sec
RG 23.2
DW 62.400 usec
DE 6.50 usec
TE 295.0 K
D1 1.00000000 sec
TD0 1
======== CHANNEL f1 ========
SFO1 400.1524711 MHz
NUC1 1H
P1 9.64 usec
SI 65536
SF 400.1500000 MHz
WDW EM
SSB 0
LB 0.30 Hz
GB 0
PC 1.00
N
N
N+
Br−
Me
1.00
2.06
1.05
1.06
4.07
4.09
2.08
2.01
2.03
2.05
2.07
3.07
2.07
2.04
10
9
8
7
6
5
4
3
2
1
ppm

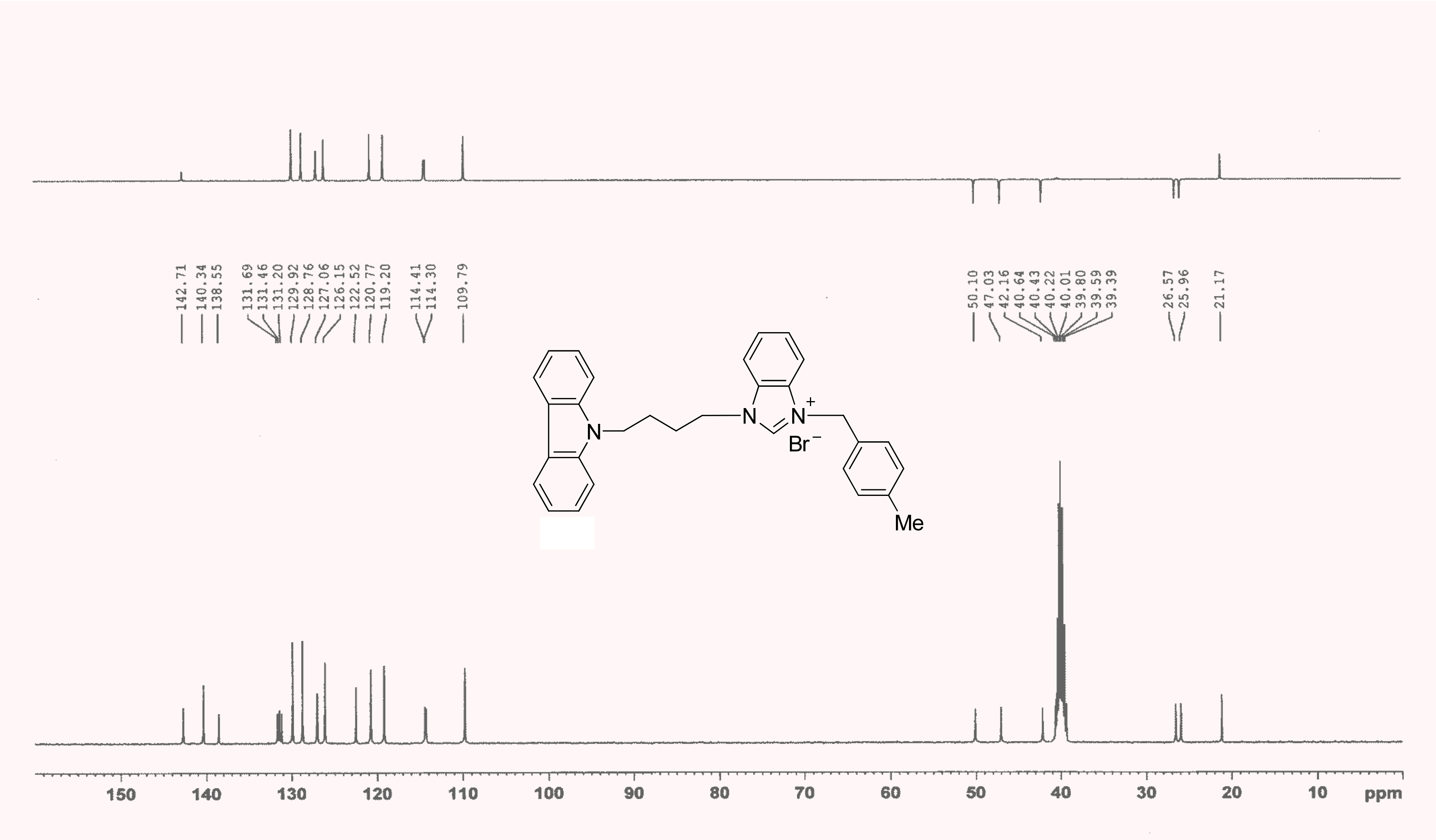
142.71
140.34
138.55
131.69
131.46
131.20
129.92
128.76
127.06
126.15
122.52
120.77
119.20
114.41
114.30
109.79
50.10
47.03
42.16
40.64
40.43
40.22
40.01
39.80
39.59
39.39
26.57
25.96
21.17
N
N
N+
Br−
Me
150
140
130
120
110
100
90
80
70
60
50
40
30
20
10
ppm

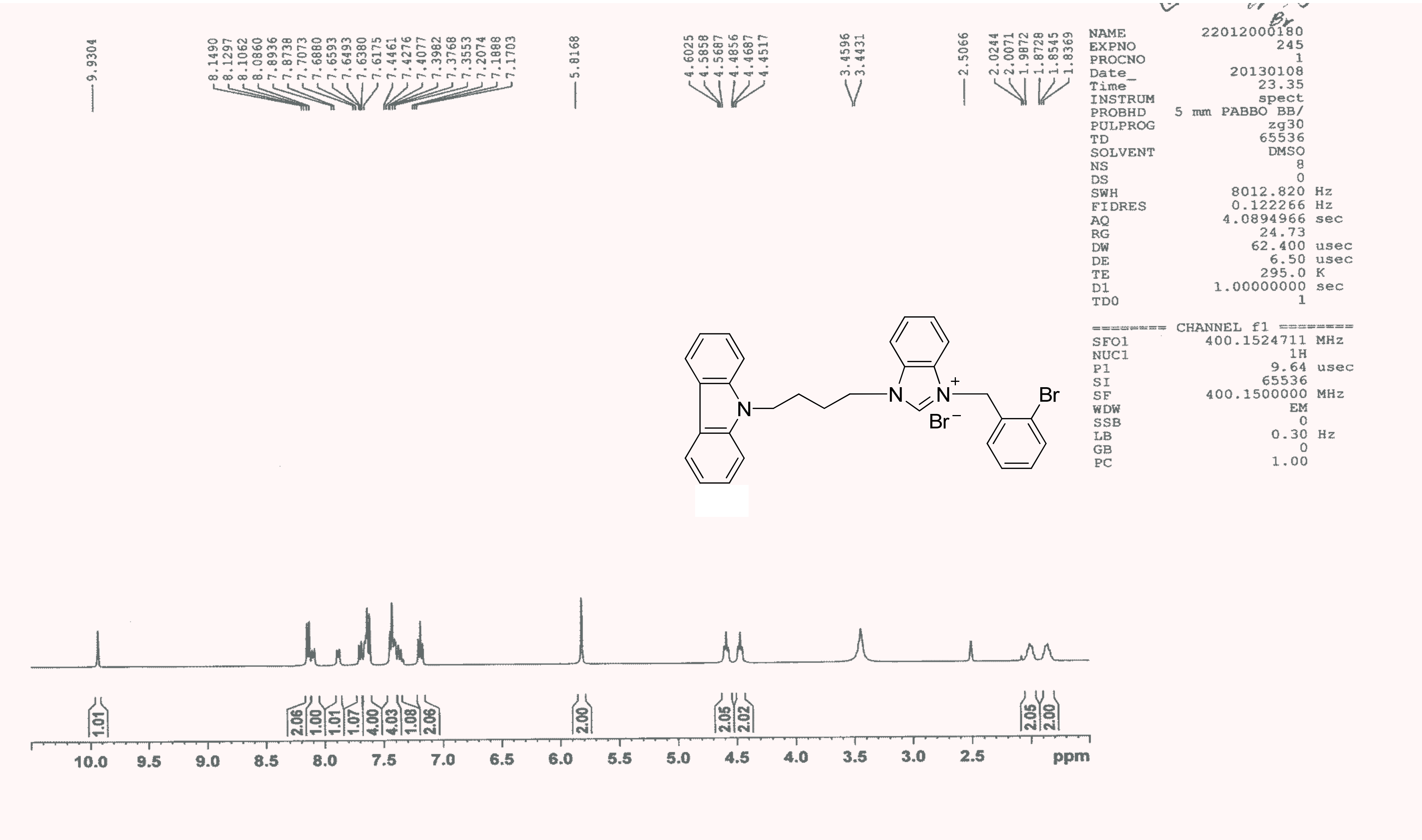

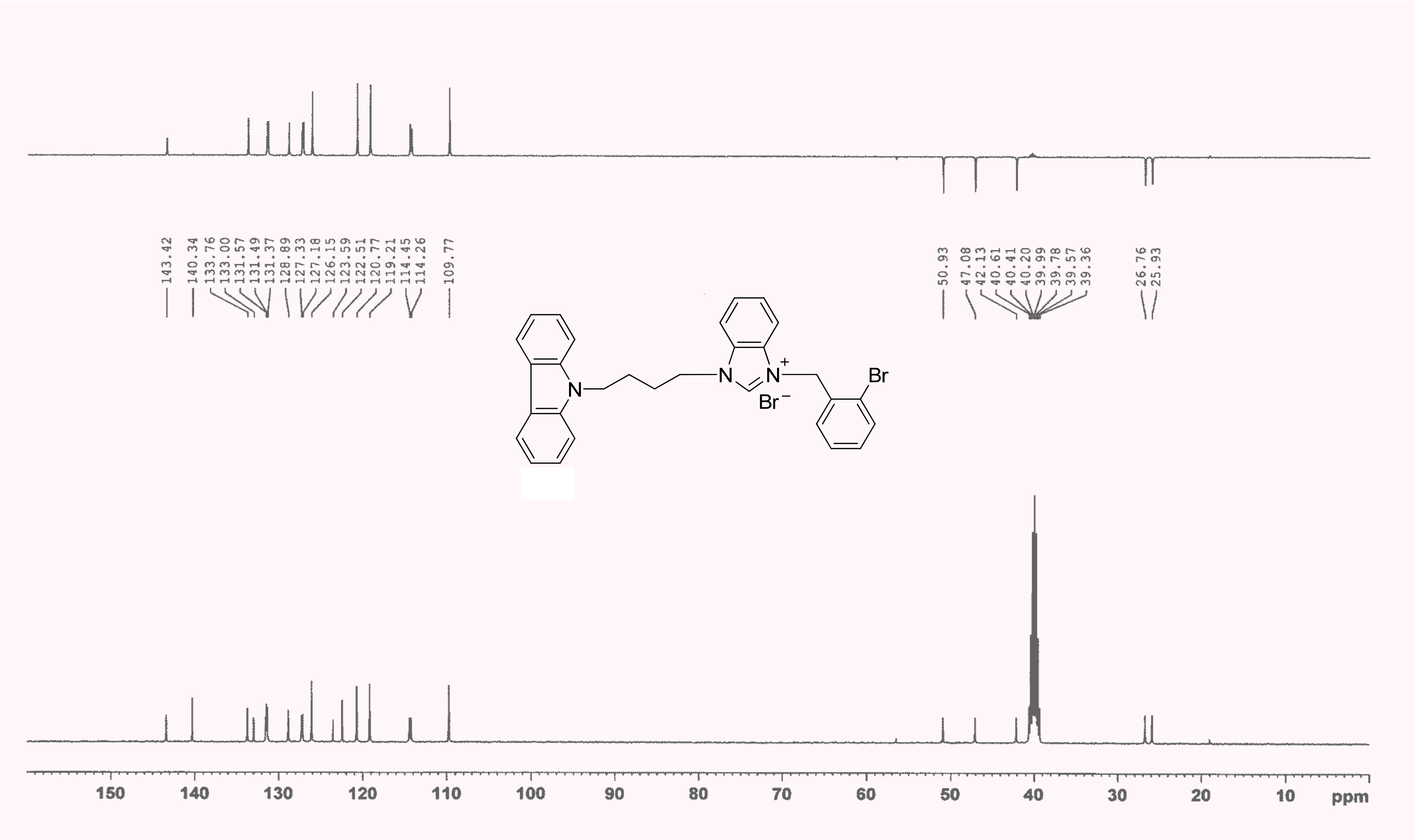
143.42
140.34
133.76
133.00
131.57
131.49
131.37
128.89
127.33
127.18
126.15
123.59
122.51
120.77
119.21
114.45
114.26
109.77
50.93
47.08
42.13
40.61
40.41
40.20
39.99
39.78
39.57
39.36
26.76
25.93
N
N
N
+
Br
Br−
150
140
130
120
110
100
90
80
70
60
50
40
30
20
10
ppm

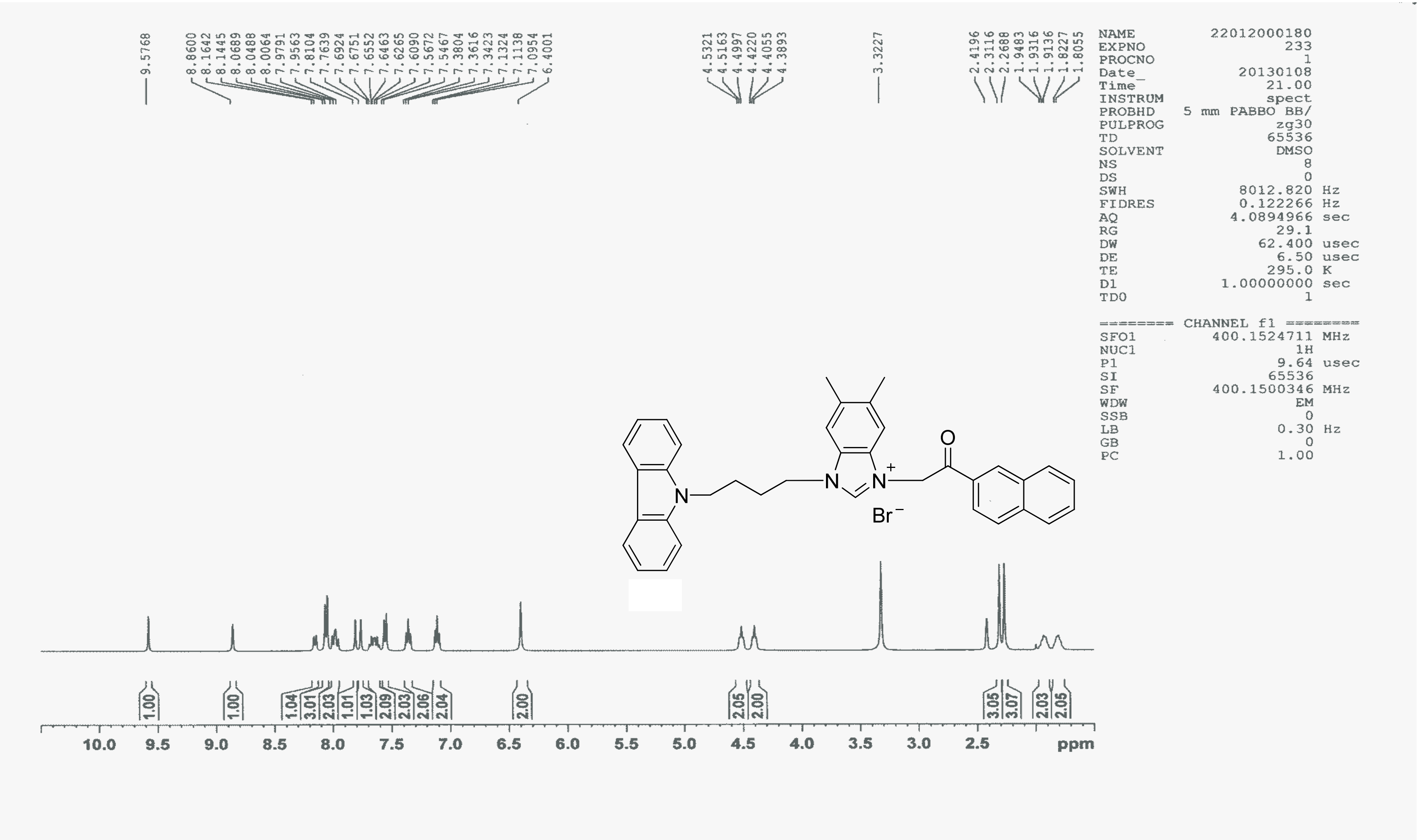
9.5768
8.8600 8.1642 8.1445 8.0689 8.0488 8.0064 7.9791 7.9563 7.8104 7.7639 7.6924 7.6751 7.6552 7.6463 7.6265 7.6090 7.5672 7.5467 7.3804 7.3616 7.3423 7.1324 7.1138 7.0954 6.4001
4.5321 4.5163 4.4997 4.4220 4.4055 4.3893
3.3227
2.4196 2.3116 2.2688 1.9483 1.9316 1.9136 1.8227 1.8055
NAME 22012000180
EXPNO 233
PROCNO 1
Date_ 20130108
Time 21.00
INSTRUM spect
PROBHD 5 mm PABBO BB/
PULPROG zg30
TD 65536
SOLVENT DMSO
NS 8
DS 0
SWH 8012.820 Hz
FIDRES 0.122266 Hz
AQ 4.0894966 sec
RG 29.1
DW 62.400 usec
DE 6.50 usec
TE 295.0 K
D1 1.00000000 sec
TD0 1
======== CHANNEL f1 ========
SFO1 400.1524711 MHz
NUC1 1H
P1 9.64 usec
SI 65536
SF 400.1500346 MHz
WDW EM
SSB 0
LB 0.30 Hz
GB 0
PC 1.00
N
N
N+
O
Br−
1.00 1.00 1.04 3.01 2.03 1.01 1.03 2.09 2.03 2.06 2.04 2.00 2.05 2.00 3.05 3.07 2.03 2.05
10.0 9.5 9.0 8.5 8.0 7.5 7.0 6.5 6.0 5.5 5.0 4.5 4.0 3.5 3.0 2.5 ppm

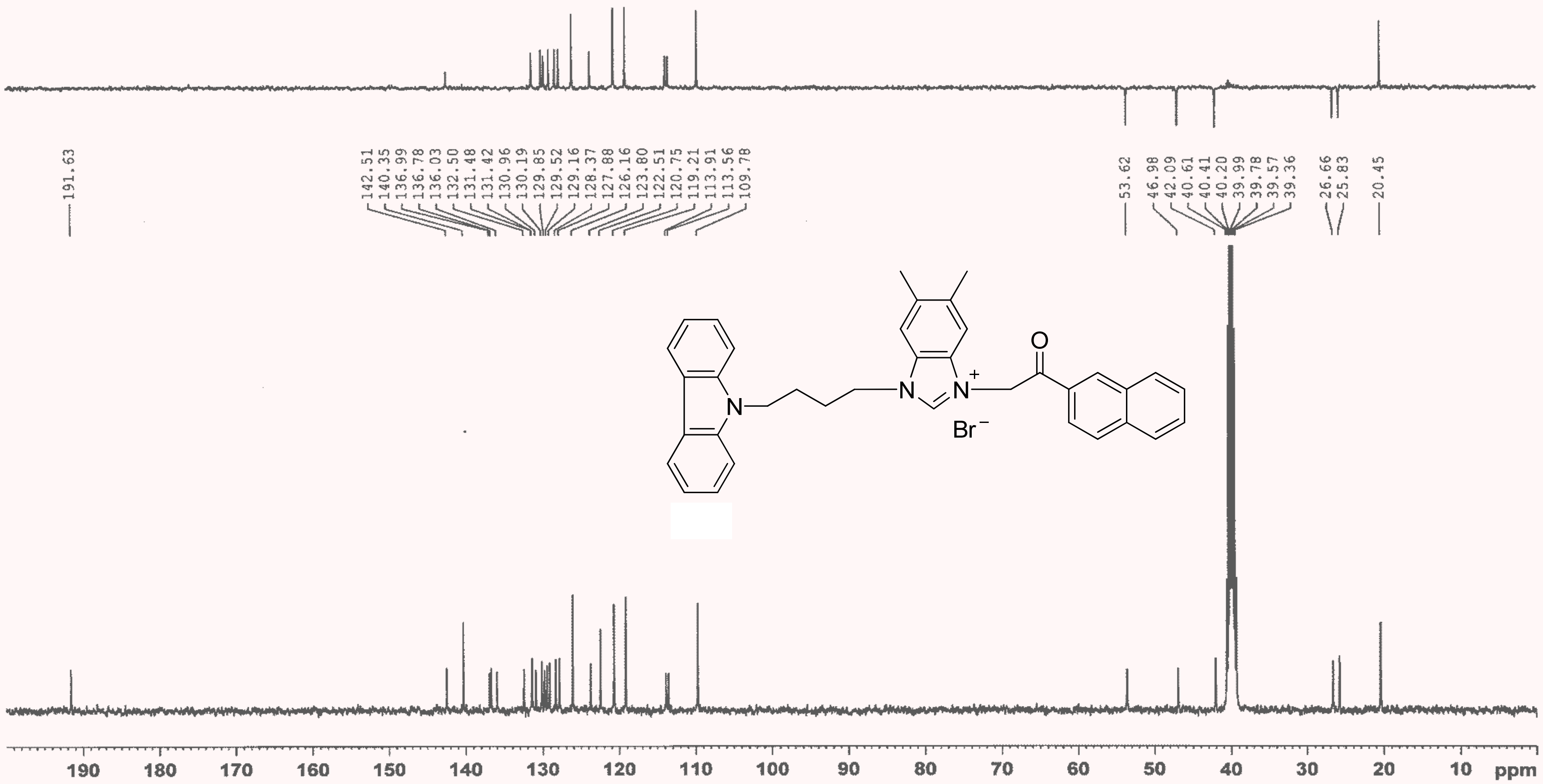
191.63
142.51
140.35
136.99
136.78
136.03
132.50
131.48
131.42
130.96
130.19
129.85
129.52
129.16
128.37
127.88
126.16
123.80
122.51
120.75
119.21
113.91
113.56
109.78
53.62
46.98
42.09
40.61
40.41
40.20
39.99
39.78
39.57
39.36
26.66
25.83
20.45
N
N
N+
O
Br−
190 180 170 160 150 140 130 120 110 100 90 80 70 60 50 40 30 20 10 ppm

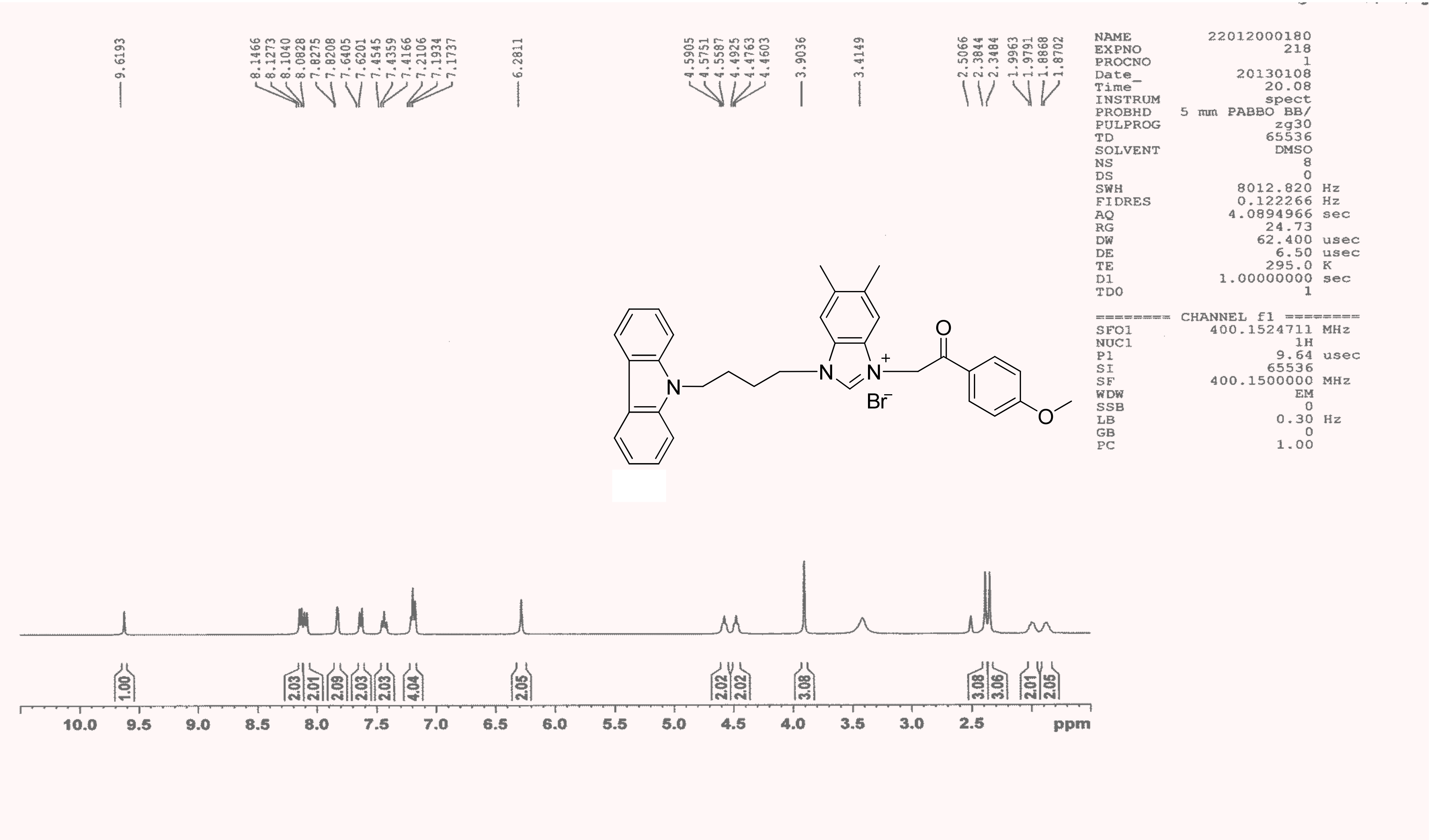
9.6193
8.1466 8.1273 8.1040 8.0828 7.8275 7.8208 7.6405 7.6201 7.4545 7.4359 7.4166 7.2106 7.1934 7.1737
6.2811
4.5905 4.5751 4.5587 4.4925 4.4763 4.4603
3.9036
3.4149
2.5066 2.3844 2.3484 1.9963 1.9791 1.8868 1.8702
NAME 22012000180
EXPNO 218
PROCNO 1
Date_ 20130108
Time_ 20.08
INSTRUM spect
PROBHD 5 mm PABBO BB/
PULPROG zg30
TD 65536
SOLVENT DMSO
NS 8
DS 0
SWH 8012.820 Hz
FIDRES 0.122266 Hz
AQ 4.0894966 sec
RG 24.73
DW 62.400 usec
DE 6.50 usec
TE 295.0 K
D1 1.00000000 sec
TD0 1
======== CHANNEL f1 ========
SFO1 400.1524711 MHz
NUC1 1H
P1 9.64 usec
SI 65536
SF 400.1500000 MHz
WDW EM
SSB 0
LB 0.30 Hz
GB 0
PC 1.00
O
N
N
N+
Br−
O
1.00 2.03 2.01 2.09 2.03 2.03 4.04 2.05 2.02 2.02 3.08 3.08 3.06 2.01 2.05
10.0 9.5 9.0 8.5 8.0 7.5 7.0 6.5 6.0 5.5 5.0 4.5 4.0 3.5 3.0 2.5 ppm

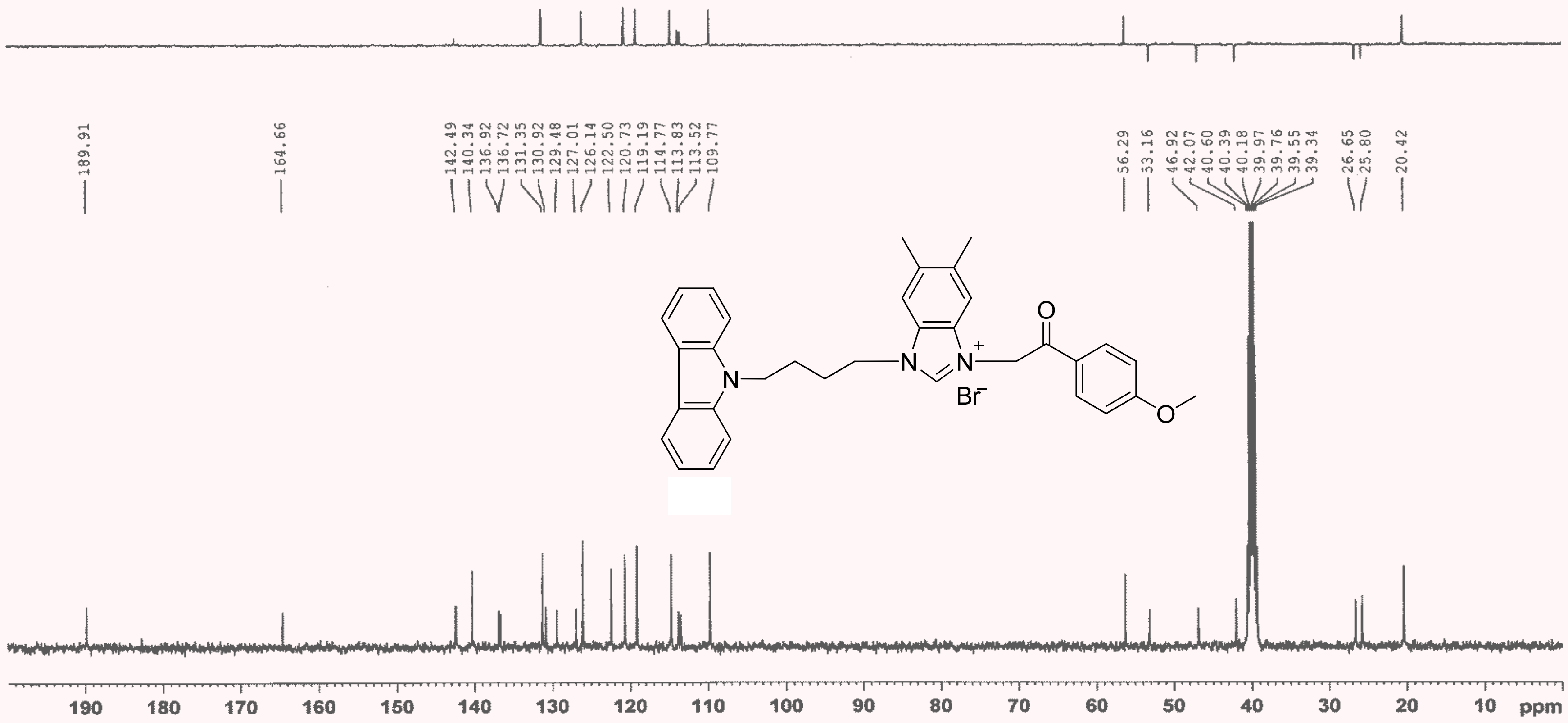

189.91
164.66
142.49
140.34
136.92
136.72
131.35
130.92
129.48
127.01
126.14
122.50
120.73
119.19
114.77
113.83
113.52
109.77
56.29
53.16
46.92
42.07
40.60
40.39
40.18
39.97
39.76
39.55
39.34
26.65
25.80
20.42
O
N
N
N+
Br−
O
190
180
170
160
150
140
130
120
110
100
90
80
70
60
50
40
30
20
10
ppm

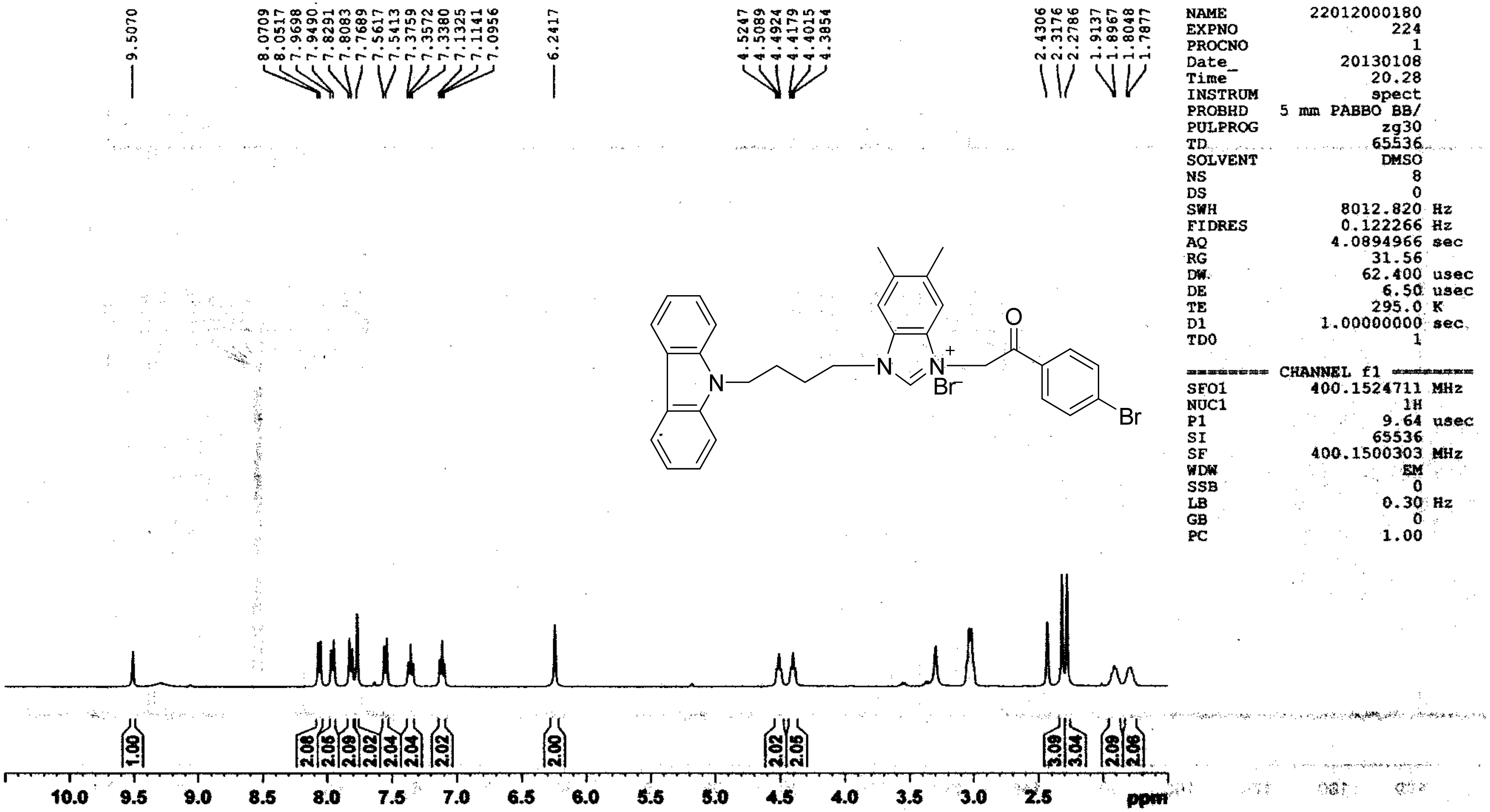
9.5070
8.0709
8.0517
7.9698
7.9490
7.8291
7.8083
7.7689
7.5617
7.5413
7.3759
7.3572
7.3380
7.1325
7.1141
7.0956
6.2417
4.5247
4.5089
4.4924
4.4179
4.4015
4.3854
2.4306
2.3176
2.2786
1.9137
1.8967
1.8048
1.7877
NAME 22012000180
EXPNO 224
PROCNO 1
Date_ 20130108
Time 20.28
INSTRUM spect
PROBHD 5 mm PABBO BB/
PULPROG zg30
TD 65536
SOLVENT DMSO
NS 8
DS 0
SWH 8012.820 Hz
FIDRES 0.122266 Hz
AQ 4.0894966 sec
RG 31.56
DW 62.400 usec
DE 6.50 usec
TE 295.0 K
D1 1.00000000 sec
TD0 1
======== CHANNEL f1 ========
SFO1 400.1524711 MHz
NUC1 1H
P1 9.64 usec
SI 65536
SF 400.1500303 MHz
WDW EM
SSB 0
LB 0.30 Hz
GB 0
PC 1.00
N
N
N+
Br−
O
Br
1.00
2.08
2.05
2.09
2.02
2.04
2.04
2.02
2.00
2.02
2.05
3.09
3.04
2.09
2.06
10.0
9.5
9.0
8.5
8.0
7.5
7.0
6.5
6.0
5.5
5.0
4.5
4.0
3.5
3.0
2.5
ppm

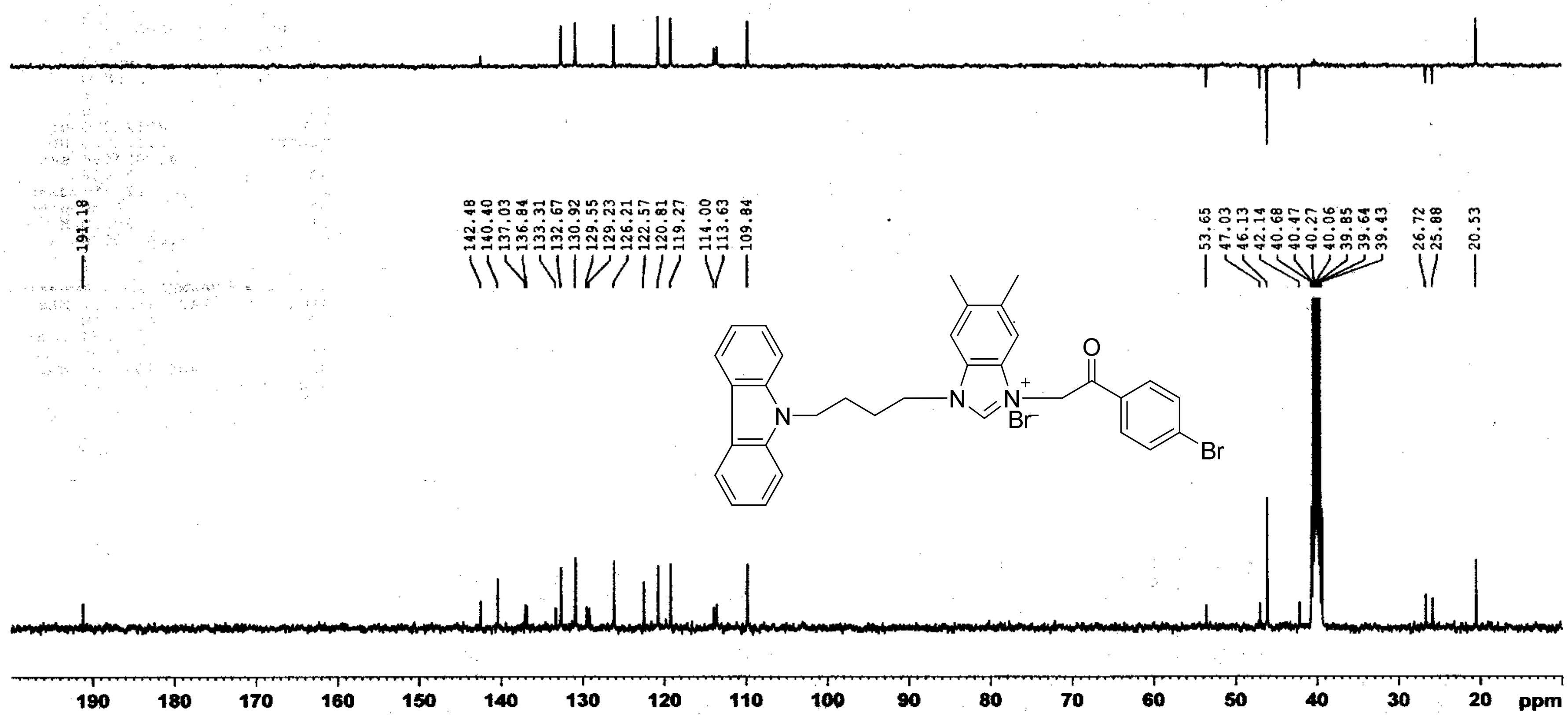

191.18
142.48
140.40
137.03
136.84
133.31
132.67
130.92
129.55
129.23
126.21
122.57
120.81
119.27
114.00
113.63
109.84
53.65
47.03
46.13
42.14
40.68
40.47
40.27
40.06
39.85
39.64
39.43
26.72
25.88
20.53
N
N
N
Br⁻
O
Br
190 180 170 160 150 140 130 120 110 100 90 80 70 60 50 40 30 20 ppm

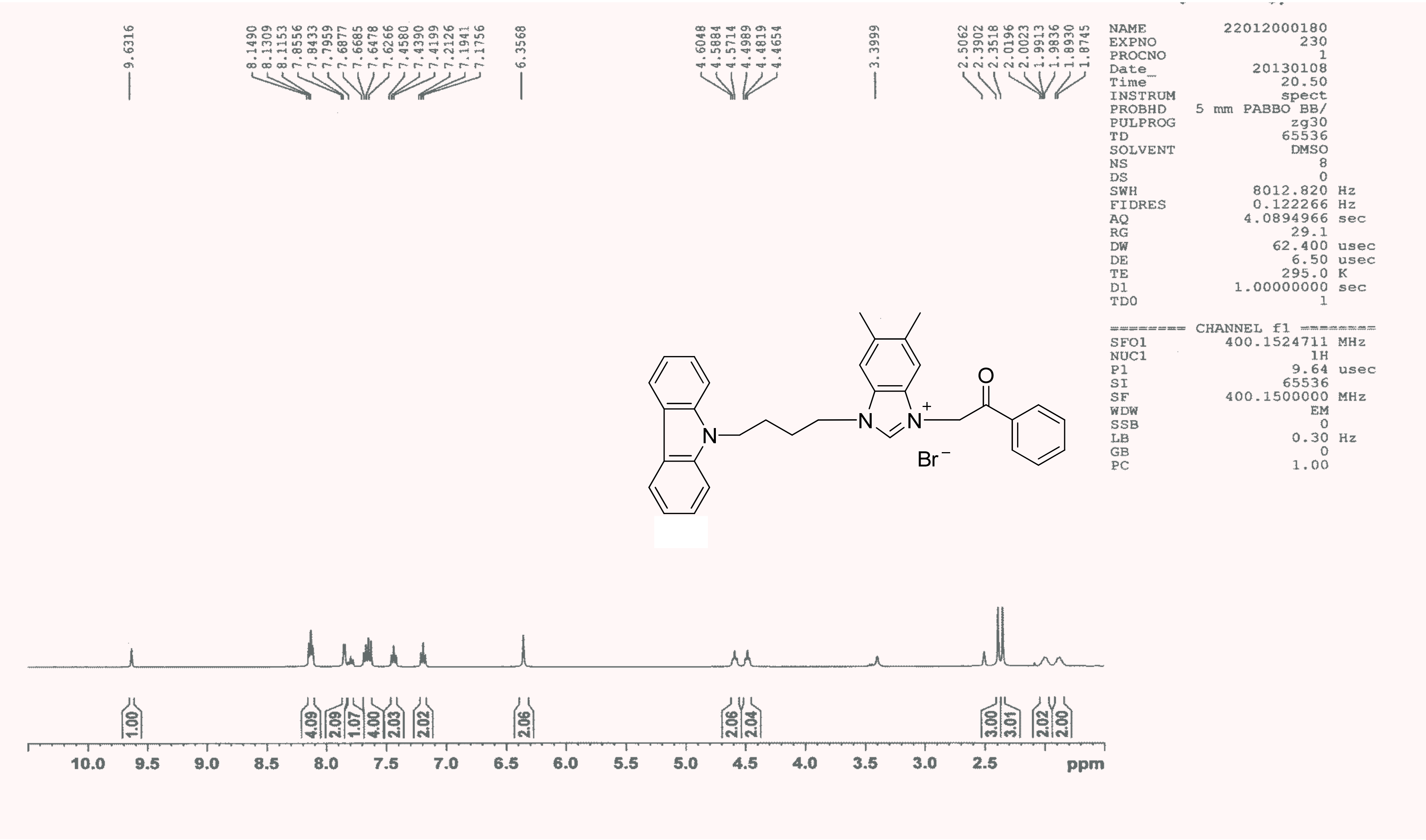
9.6316
8.1490 8.1309 8.1153 7.8556 7.8433 7.7959 7.6877 7.6685 7.6478 7.6266 7.4580 7.4390 7.4199 7.2126 7.1941 7.1756
6.3568
4.6048 4.5884 4.5714 4.4989 4.4819 4.4654
3.3999
2.5062 2.3902 2.3518 2.0196 2.0023 1.9913 1.9836 1.8930 1.8745
NAME 22012000180
EXPNO 230
PROCNO 1
Date_ 20130108
Time 20.50
INSTRUM spect
PROBHD 5 mm PABBO BB/
PULPROG zg30
TD 65536
SOLVENT DMSO
NS 8
DS 0
SWH 8012.820 Hz
FIDRES 0.122266 Hz
AQ 4.0894966 sec
RG 29.1
DW 62.400 usec
DE 6.50 usec
TE 295.0 K
D1 1.00000000 sec
TD0 1
======== CHANNEL f1 ========
SFO1 400.1524711 MHz
NUC1 1H
P1 9.64 usec
SI 65536
SF 400.1500000 MHz
WDW EM
SSB 0
LB 0.30 Hz
GB 0
PC 1.00
N
N
N+
O
Br−
1.00
4.09 2.09 1.07 4.00 2.03 2.02
2.06
2.06 2.04
3.00 3.01 2.02 2.00
10.0 9.5 9.0 8.5 8.0 7.5 7.0 6.5 6.0 5.5 5.0 4.5 4.0 3.5 3.0 2.5 ppm

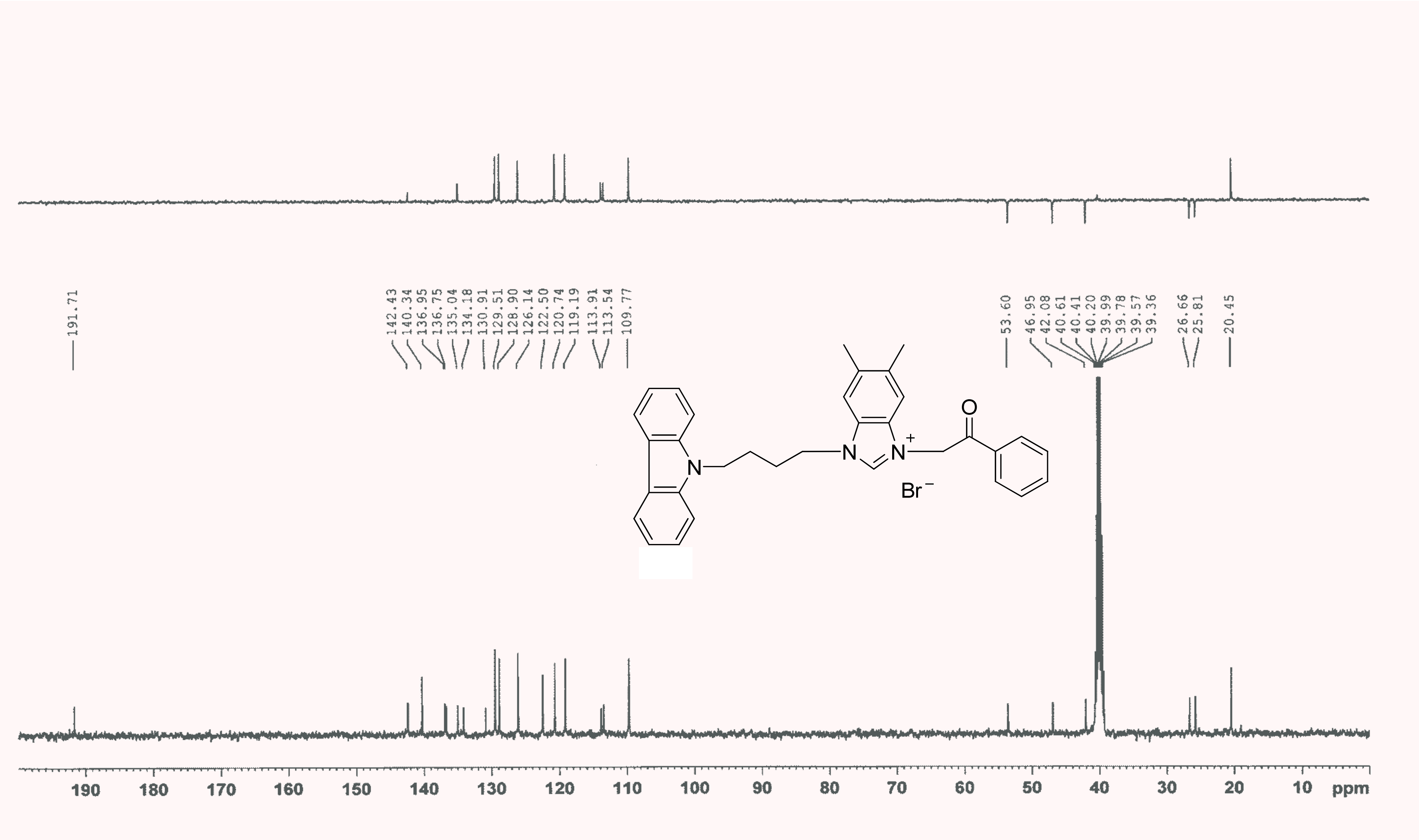
191.71
142.43
140.34
136.95
136.75
135.04
134.18
130.91
129.51
128.90
126.14
122.50
120.74
119.19
113.91
113.54
109.77
53.60
46.95
42.08
40.61
40.41
40.20
39.99
39.78
39.57
39.36
26.66
25.81
20.45
N
N
N+
O
Br−
190 180 170 160 150 140 130 120 110 100 90 80 70 60 50 40 30 20 10 ppm

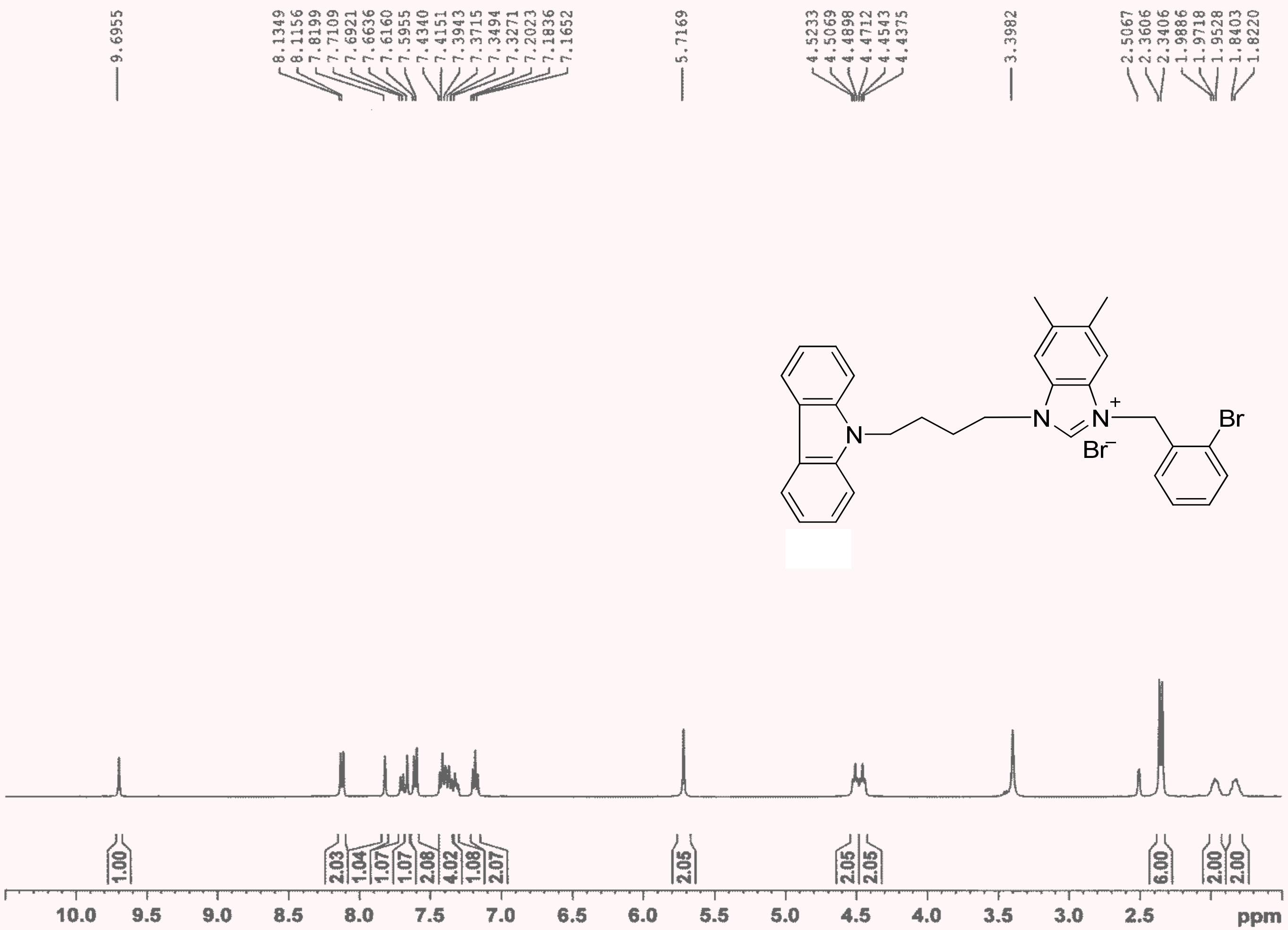

NAME	22012000180
EXPNO	227
PROCNO	1
Date_	20130108
Time	20.42
INSTRUM	spect
PROBHD	5 mm PABBO BB/
PULPROG	zg30
TD	65536
SOLVENT	DMSO
NS	8
DS	0
SWH	8012.820 Hz
FIDRES	0.122266 Hz
AQ	4.0894966 sec
RG	24.73
DW	62.400 usec
DE	6.50 usec
TE	295.0 K
D1	1.00000000 sec
TD0	1

======== CHANNEL f1 ========

SFO1	400.1524711 MHz
NUC1	1H
P1	9.64 usec
SI	65536
SF	400.1500000 MHz
WDW	EM
SSB	0
LB	0.30 Hz
GB	0
PC	1.00

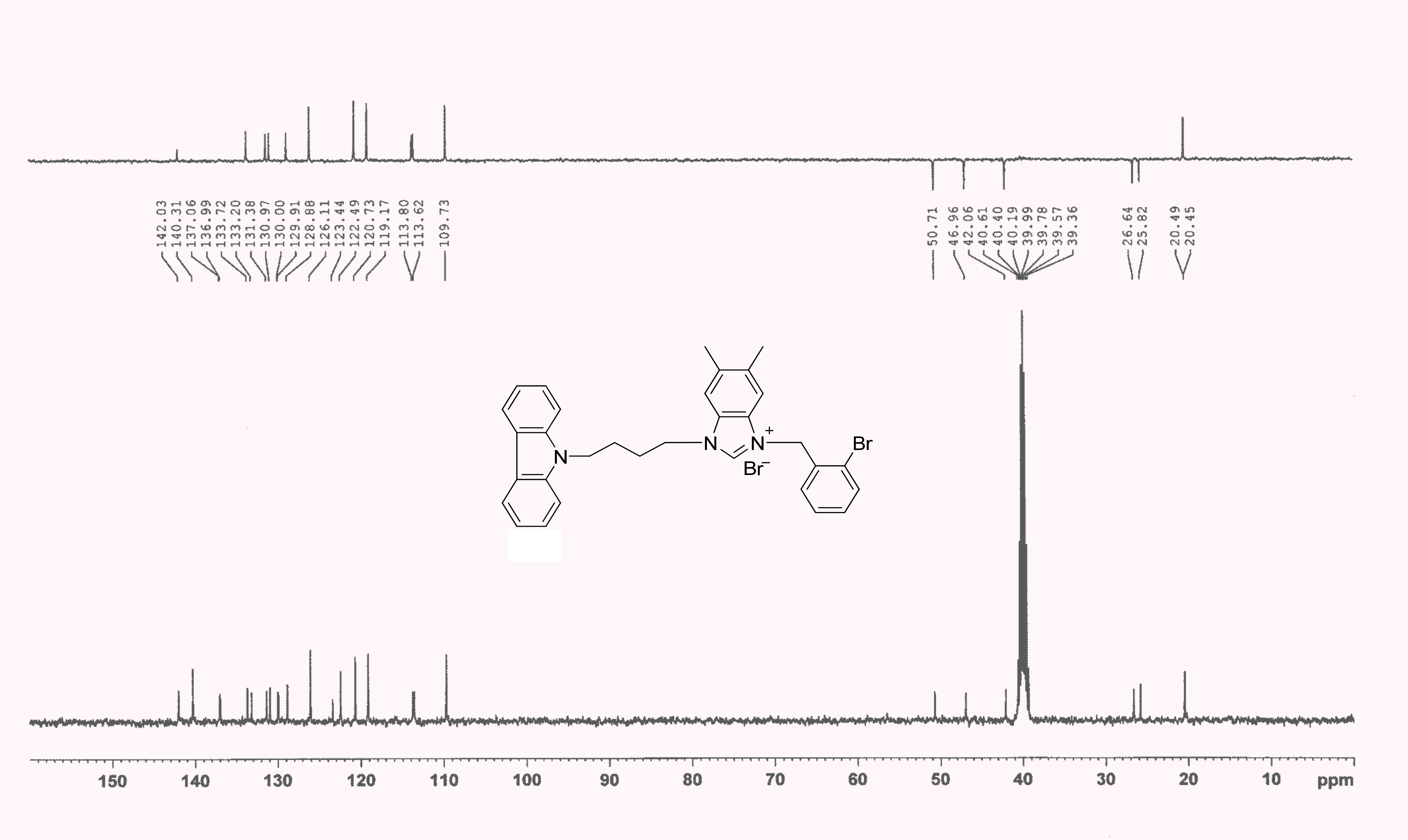
142.03
140.31
137.06
136.99
133.72
133.20
131.38
130.97
130.00
129.91
128.88
126.11
123.44
122.49
120.73
119.17
113.80
113.62
109.73
50.71
46.96
42.06
40.61
40.40
40.19
39.99
39.78
39.57
39.36
26.64
25.82
20.49
20.45
N
N
N+
Br
Br−
150
140
130
120
110
100
90
80
70
60
50
40
30
20
10
ppm

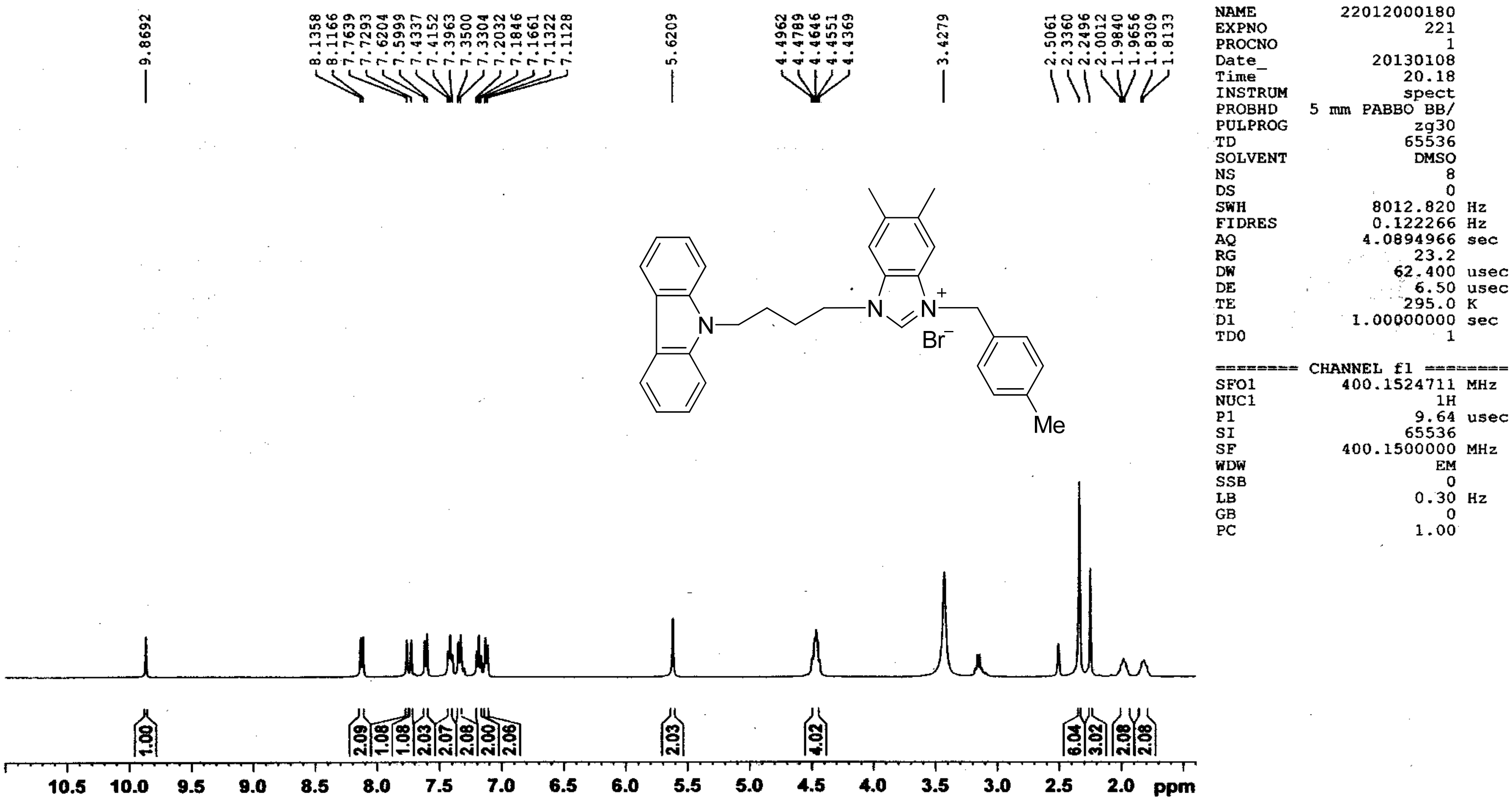
9.8692
8.1358
8.1166
7.7639
7.7293
7.6204
7.5999
7.4337
7.4152
7.3963
7.3500
7.3304
7.2032
7.1846
7.1661
7.1322
7.1128
5.6209
4.4962
4.4789
4.4646
4.4551
4.4369
3.4279
2.5061
2.3360
2.2496
2.0012
1.9840
1.9656
1.8309
1.8133
N
N
N
+
Br⁻
Me
NAME 22012000180
EXPNO 221
PROCNO 1
Date_ 20130108
Time 20.18
INSTRUM spect
PROBHD 5 mm PABBO BB/
PULPROG zg30
TD 65536
SOLVENT DMSO
NS 8
DS 0
SWH 8012.820 Hz
FIDRES 0.122266 Hz
AQ 4.0894966 sec
RG 23.2
DW 62.400 usec
DE 6.50 usec
TE 295.0 K
D1 1.00000000 sec
TD0 1
======== CHANNEL f1 ========
SFO1 400.1524711 MHz
NUC1 1H
P1 9.64 usec
SI 65536
SF 400.1500000 MHz
WDW EM
SSB 0
LB 0.30 Hz
GB 0
PC 1.00
1.00
2.09
1.08
1.08
2.03
2.07
2.08
2.00
2.06
2.03
4.02
6.04
3.02
2.08
2.08
10.5
10.0
9.5
9.0
8.5
8.0
7.5
7.0
6.5
6.0
5.5
5.0
4.5
4.0
3.5
3.0
2.5
2.0
ppm

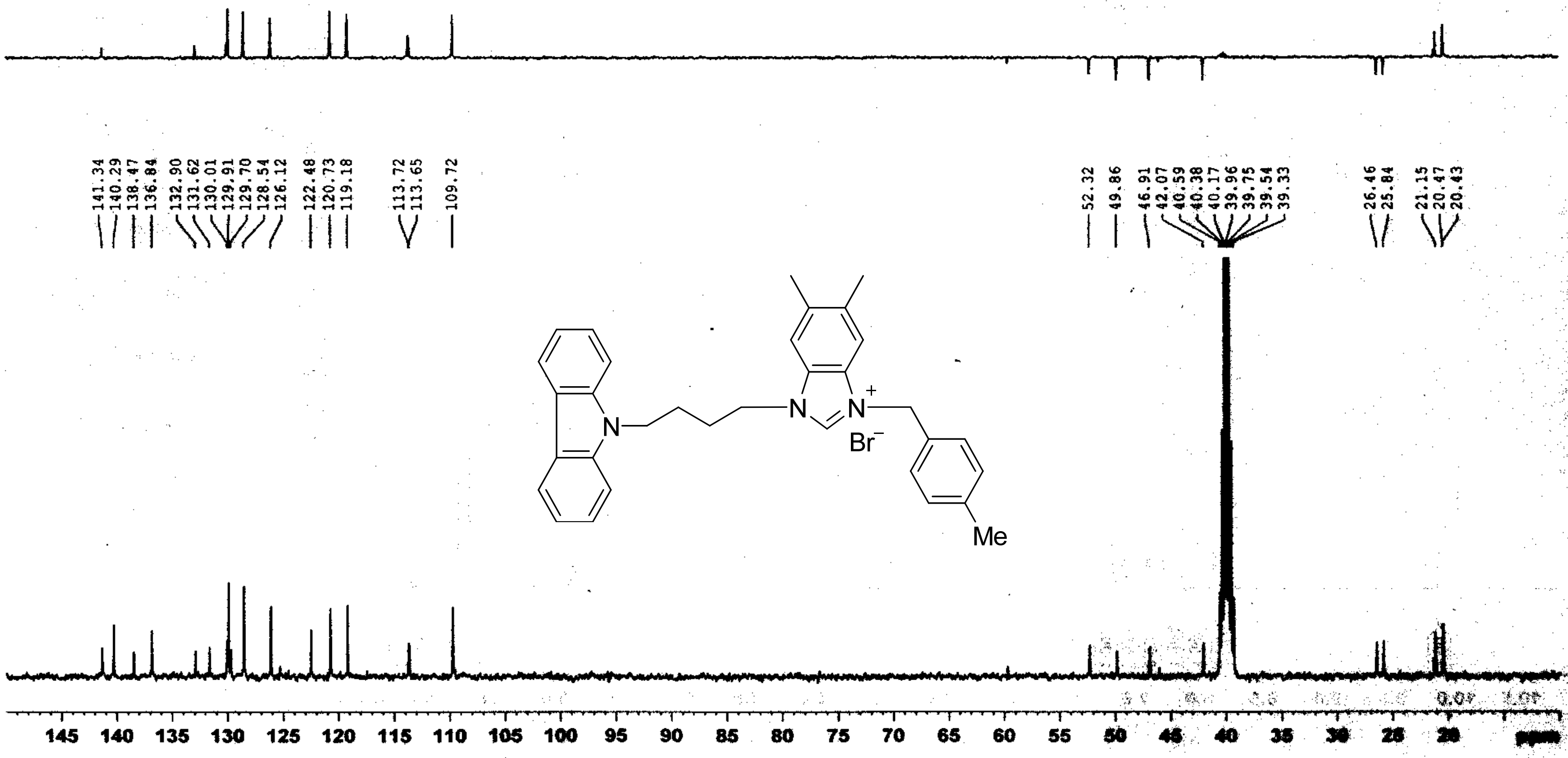
141.34
140.29
138.47
136.84
132.90
131.62
130.01
129.91
129.70
128.54
126.12
122.48
120.73
119.18
113.72
113.65
109.72
52.32
49.86
46.91
42.07
40.59
40.38
40.17
39.96
39.75
39.54
39.33
26.46
25.84
21.15
20.47
20.43
N
N
N+
Br−
Me
145
140
135
130
125
120
115
110
105
100
95
90
85
80
75
70
65
60
55
50
45
40
35
30
25
20
ppm

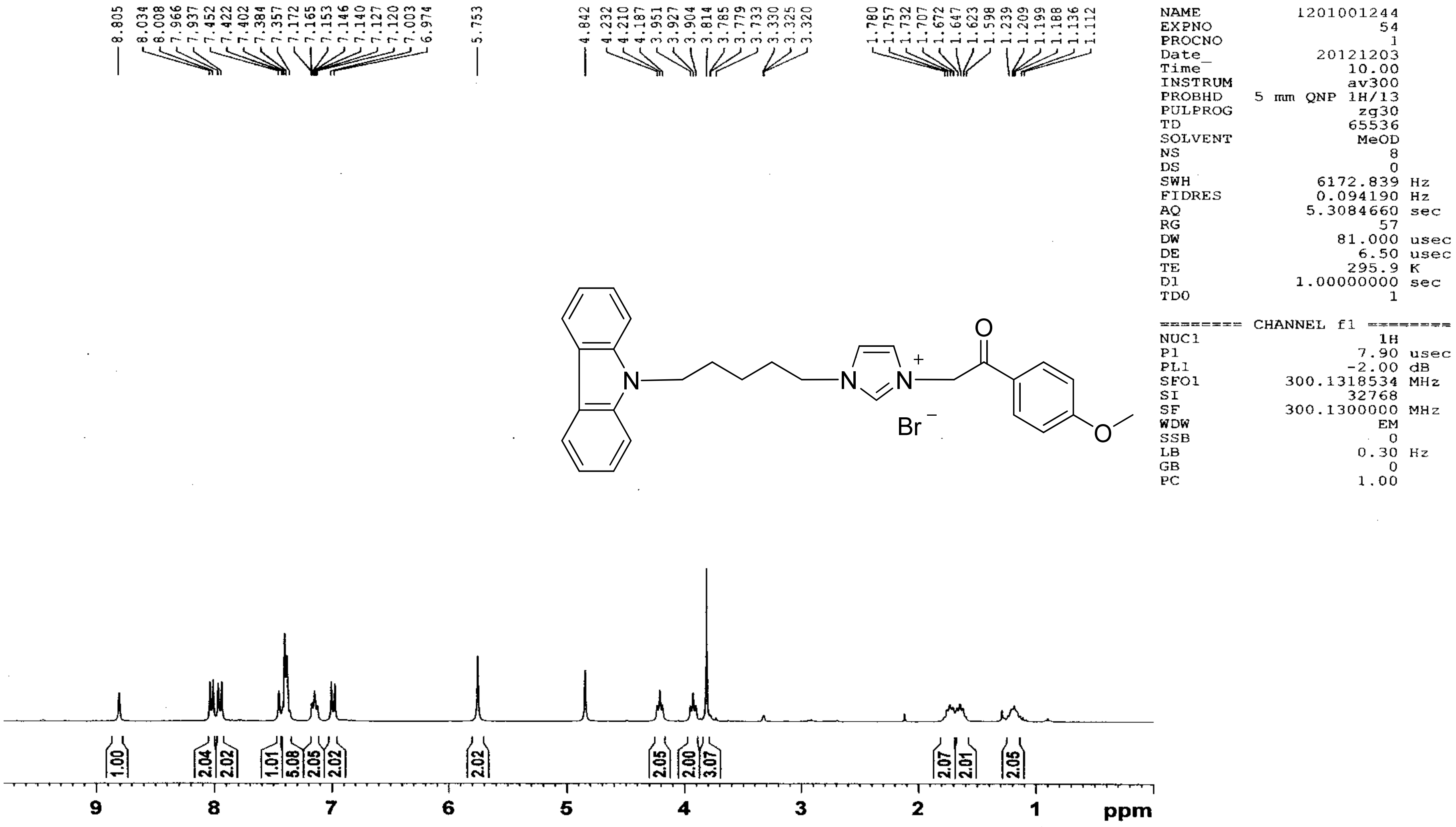
8.805
8.034
8.008
7.966
7.937
7.452
7.422
7.402
7.384
7.357
7.172
7.165
7.153
7.146
7.140
7.127
7.120
7.003
6.974
5.753
4.842
4.232
4.210
4.187
3.951
3.927
3.904
3.814
3.785
3.779
3.733
3.330
3.325
3.320
1.780
1.757
1.732
1.707
1.672
1.647
1.623
1.598
1.239
1.209
1.199
1.188
1.136
1.112
NAME 1201001244
EXPNO 54
PROCNO 1
Date_ 20121203
Time 10.00
INSTRUM av300
PROBHD 5 mm QNP 1H/13
PULPROG zg30
TD 65536
SOLVENT MeOD
NS 8
DS 0
SWH 6172.839 Hz
FIDRES 0.094190 Hz
AQ 5.3084660 sec
RG 57
DW 81.000 usec
DE 6.50 usec
TE 295.9 K
D1 1.00000000 sec
TD0 1
======== CHANNEL f1 ========
NUC1 1H
P1 7.90 usec
PL1 -2.00 dB
SFO1 300.1318534 MHz
SI 32768
SF 300.1300000 MHz
WDW EM
SSB 0
LB 0.30 Hz
GB 0
PC 1.00
N
N
N
+
O
O
Br −
1.00
2.04
2.02
1.01
5.08
2.05
2.02
2.02
2.05
2.00
3.07
2.07
2.01
2.05
9
8
7
6
5
4
3
2
1
ppm

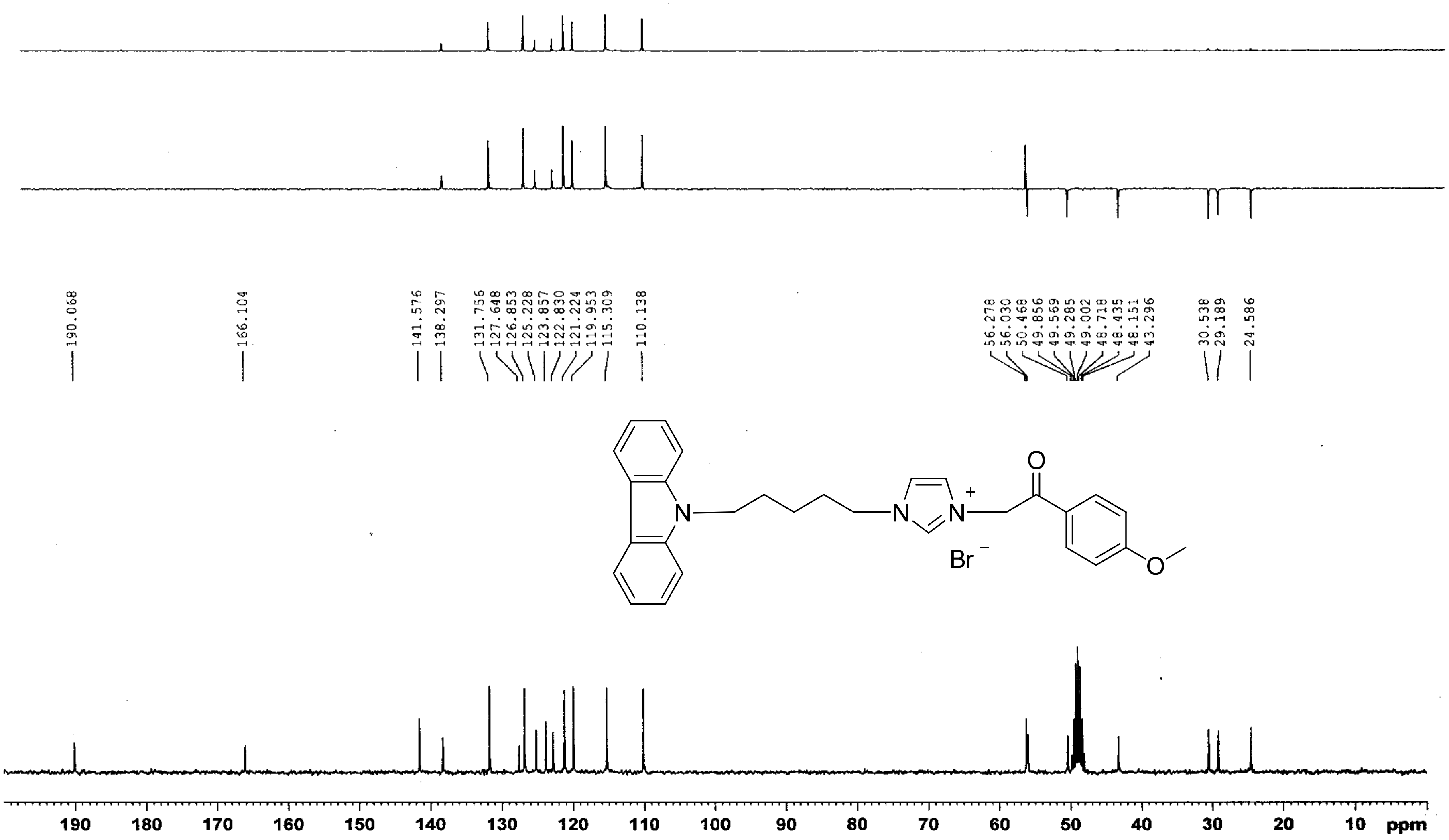
190.068
166.104
141.576
138.297
131.756
127.648
126.853
125.228
123.857
122.830
121.224
119.953
115.309
110.138
56.278
56.030
50.468
49.856
49.569
49.285
49.002
48.718
48.435
48.151
43.296
30.538
29.189
24.586
N
N
N
+
O
O
Br −
190
180
170
160
150
140
130
120
110
100
90
80
70
60
50
40
30
20
10
ppm

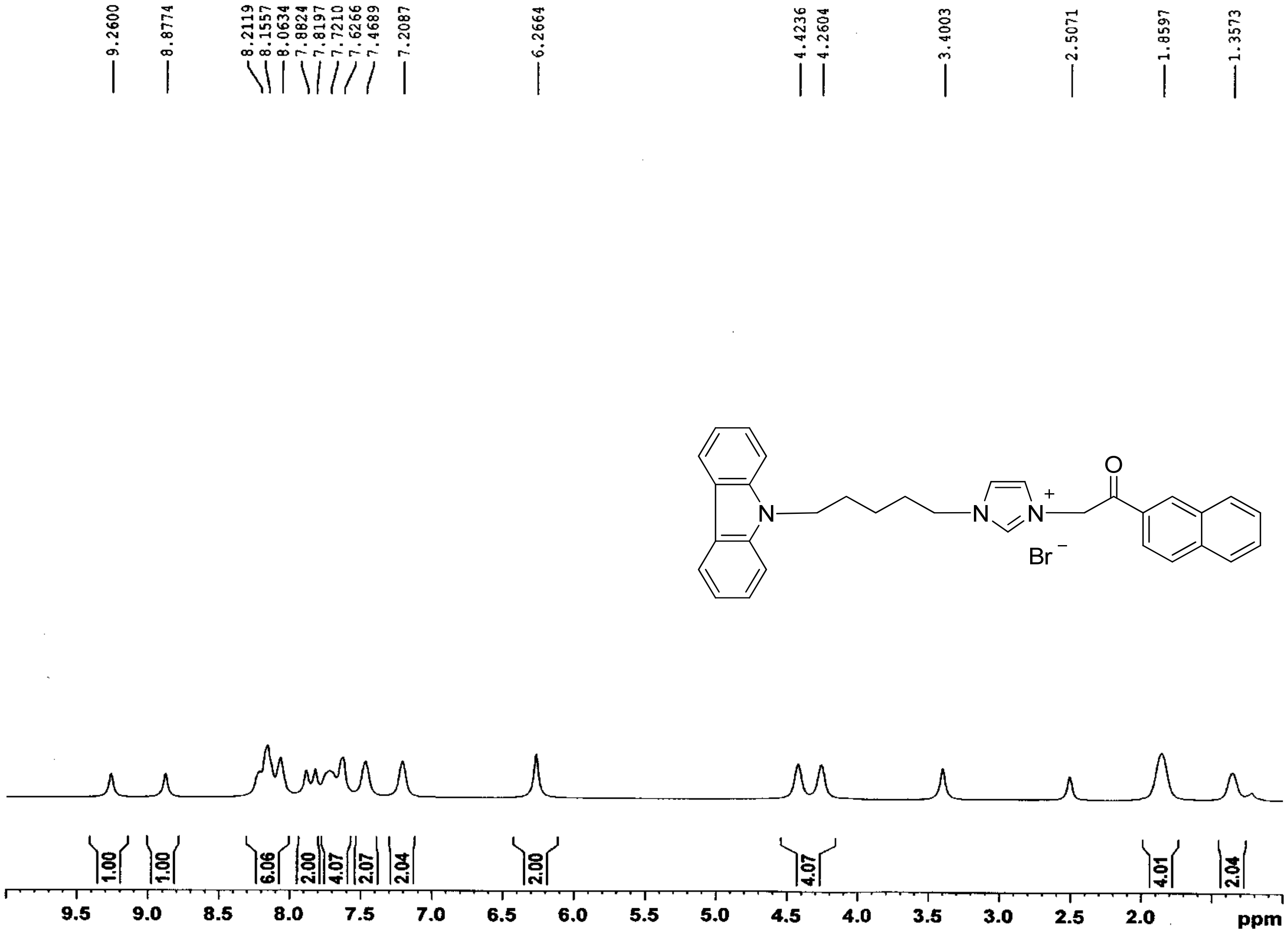

NAME	22012000180
EXPNO	155
PROCNO	1
Date_	20121230
Time	21.20
INSTRUM	spect
PROBHD	5 mm PABBO BB/
PULPROG	zg30
TD	65536
SOLVENT	DMSO
NS	8
DS	0
SWH	8012.820 Hz
FIDRES	0.122266 Hz
AQ	4.0894966 sec
RG	29.1
DW	62.400 usec
DE	6.50 usec
TE	297.0 K
D1	1.00000000 sec
TD0	1

======== CHANNEL f1 ========

SFO1	400.1524711 MHz
NUC1	1H
P1	9.64 usec
SI	65536
SF	400.1500000 MHz
WDW	EM
SSB	0
LB	0.30 Hz
GB	0
PC	1.00

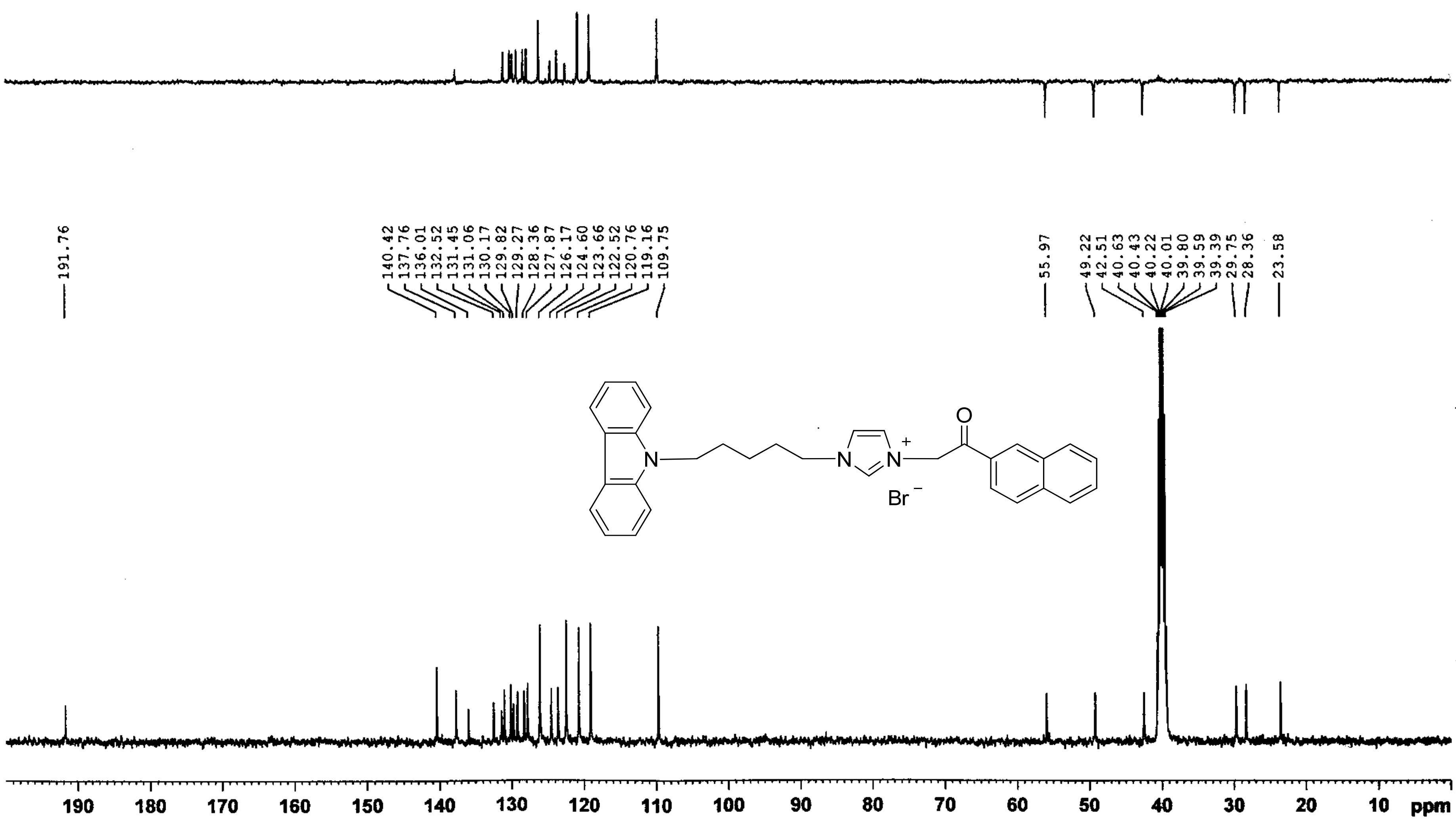
191.76
140.42
137.76
136.01
132.52
131.45
131.06
130.17
129.82
129.27
128.36
127.87
126.17
124.60
123.66
122.52
120.76
119.16
109.75
55.97
49.22
42.51
40.63
40.43
40.22
40.01
39.80
39.59
39.39
29.75
28.36
23.58
Br⁻
190 180 170 160 150 140 130 120 110 100 90 80 70 60 50 40 30 20 10 ppm

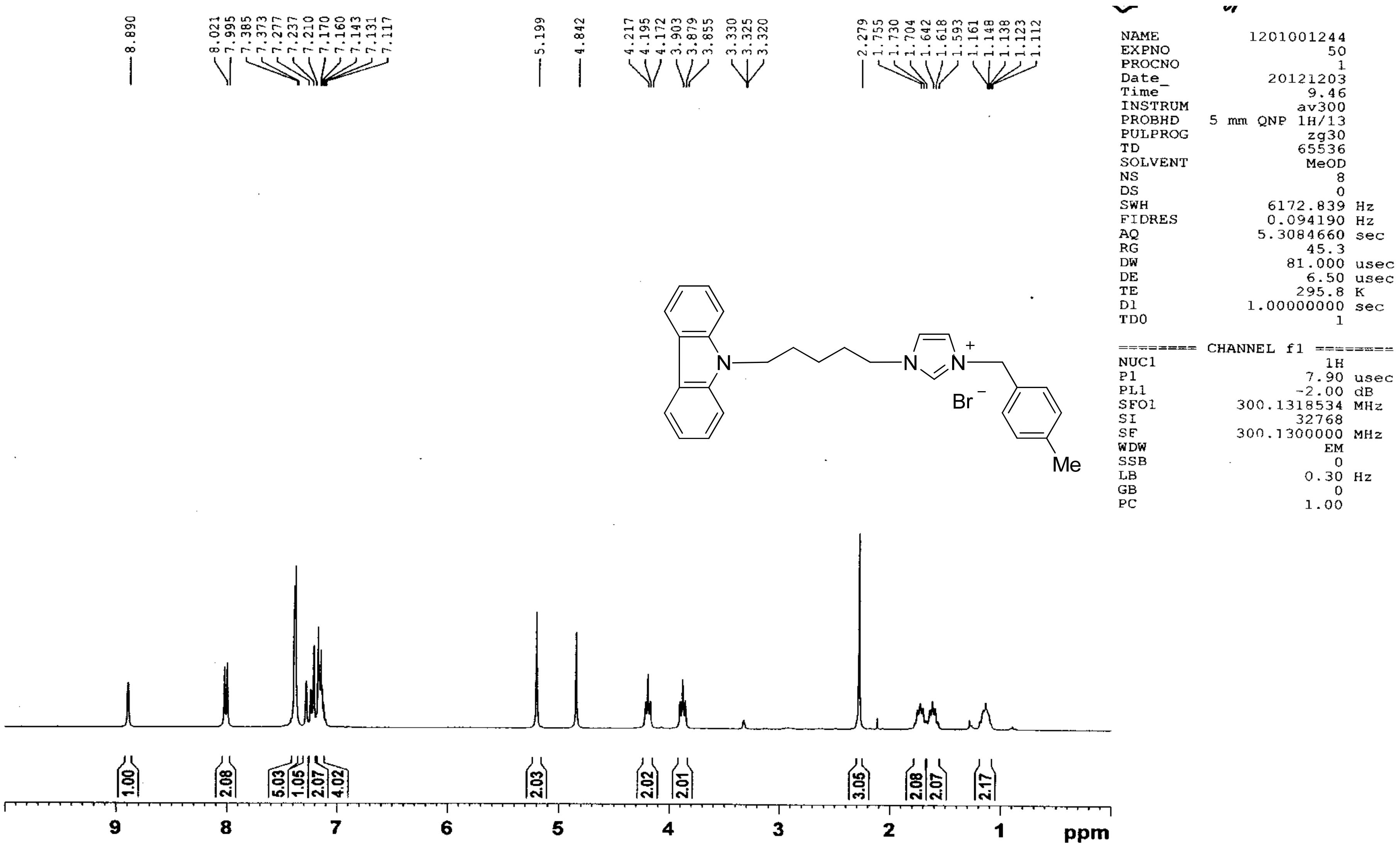
8.890
8.021 7.995 7.385 7.373 7.277 7.237 7.210 7.170 7.160 7.143 7.131 7.117
5.199
4.842
4.217 4.195 4.172 3.903 3.879 3.855 3.330 3.325 3.320
2.279 1.755 1.730 1.704 1.642 1.618 1.593 1.161 1.148 1.138 1.123 1.112
N
N
N
+
Br −
Me
NAME 1201001244
EXPNO 50
PROCNO 1
Date_ 20121203
Time 9.46
INSTRUM av300
PROBHD 5 mm QNP 1H/13
PULPROG zg30
TD 65536
SOLVENT MeOD
NS 8
DS 0
SWH 6172.839 Hz
FIDRES 0.094190 Hz
AQ 5.3084660 sec
RG 45.3
DW 81.000 usec
DE 6.50 usec
TE 295.8 K
D1 1.00000000 sec
TD0 1
======== CHANNEL f1 ========
NUC1 1H
P1 7.90 usec
PL1 -2.00 dB
SFO1 300.1318534 MHz
SI 32768
SF 300.1300000 MHz
WDW EM
SSB 0
LB 0.30 Hz
GB 0
PC 1.00
1.00 2.08 5.03 1.05 2.07 4.02 2.03 2.02 2.01 3.05 2.08 2.07 2.17
9 8 7 6 5 4 3 2 1 ppm

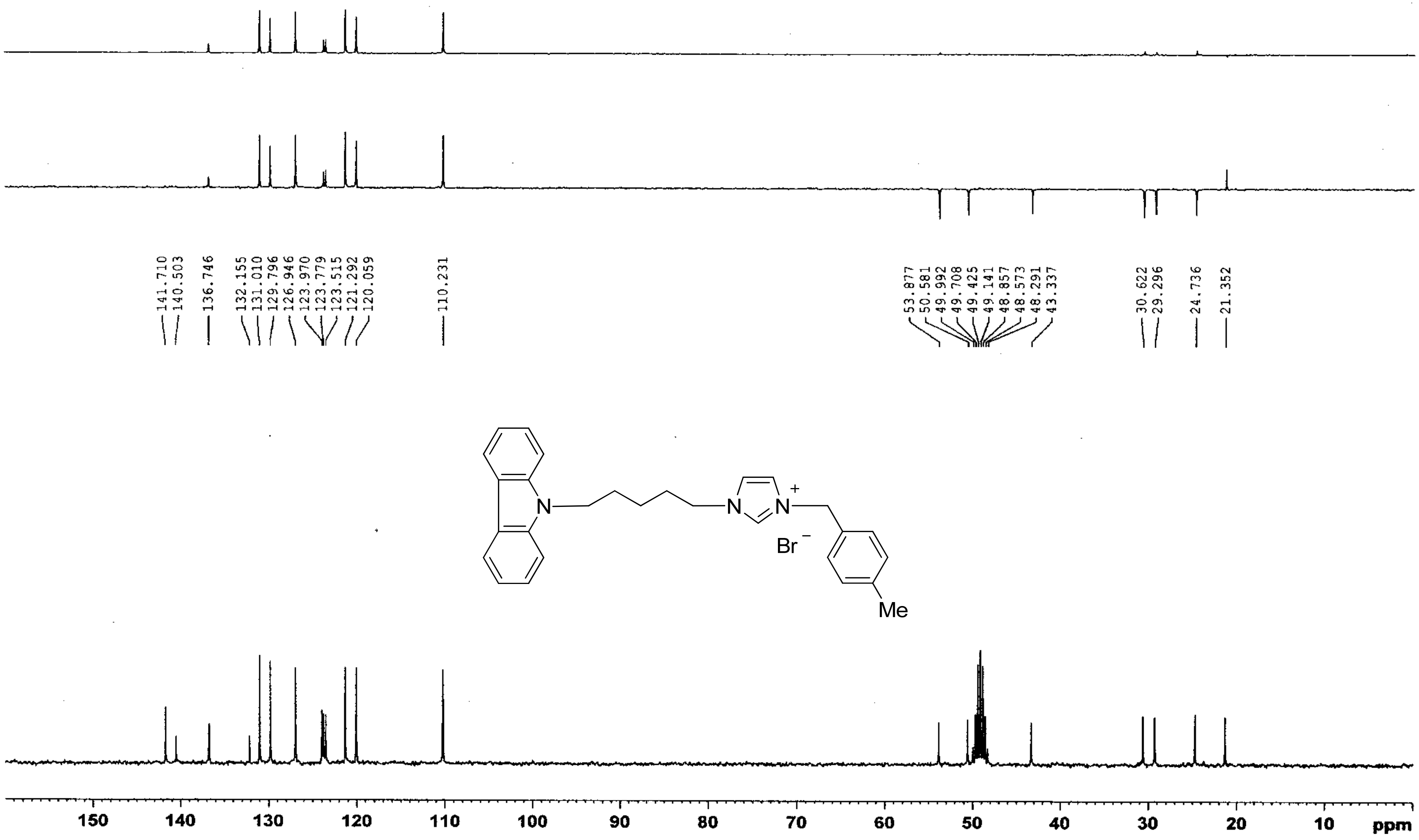
141.710
140.503
136.746
132.155
131.010
129.796
126.946
123.970
123.779
123.515
121.292
120.059
110.231
53.877
50.581
49.992
49.708
49.425
49.141
48.857
48.573
48.291
43.337
30.622
29.296
24.736
21.352
N
N
N
+
Br −
Me
150
140
130
120
110
100
90
80
70
60
50
40
30
20
10
ppm

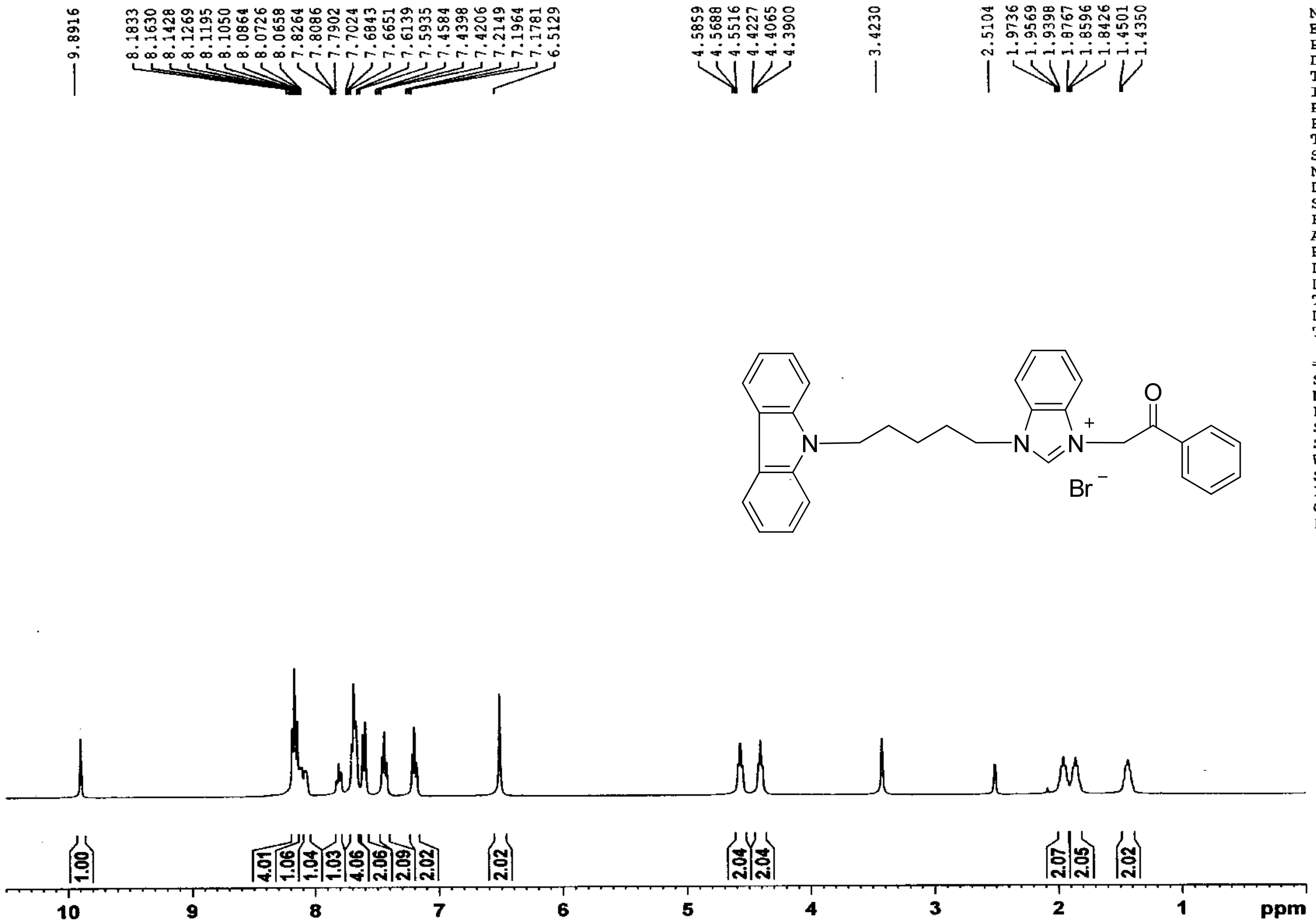

NAME	22012000180	
EXPNO	158	
PROCNO	1	
Date_	20121230	
Time	21.59	
INSTRUM	spect	
PROBHD	5 mm PABBO BB/	
PULPROG	zg30	
TD	65536	
SOLVENT	DMSO	
NS	8	
DS	0	
SWH	8012.820	Hz
FIDRES	0.122266	Hz
AQ	4.0894966	sec
RG	23.2	
DW	62.400	usec
DE	6.50	usec
TE	297.0	K
D1	1.00000000	sec
TD0	1	
======== CHANNEL f1 ========		
SFO1	400.1524711	MHz
NUC1	1H	
P1	9.64	usec
SI	65536	
SF	400.1499984	MHz
WDW	EM	
SSB	0	
LB	0.30	Hz
GB	0	
PC	1.00	

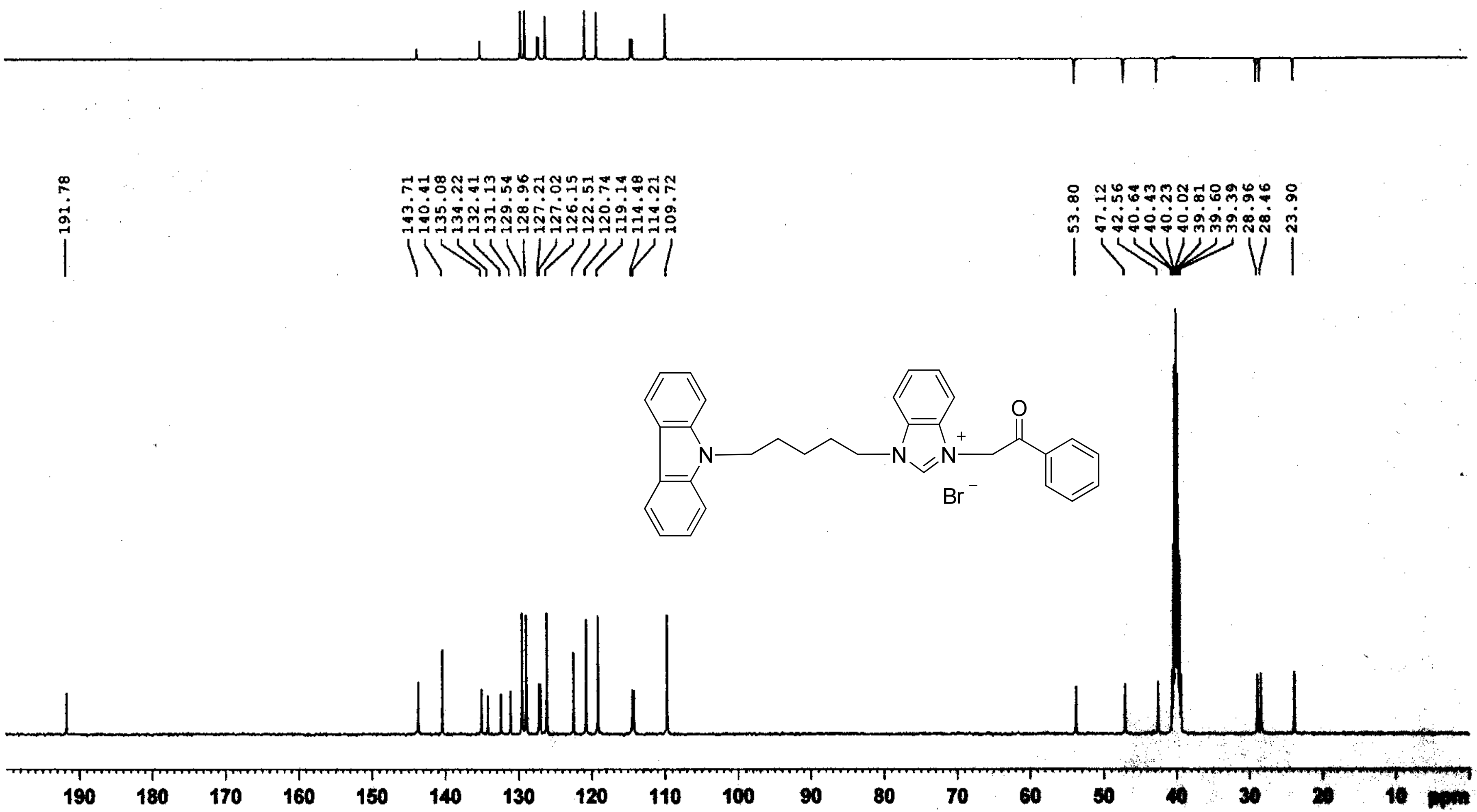

191.78
143.71
140.41
135.08
134.22
132.41
131.13
129.54
128.96
127.21
127.02
126.15
122.51
120.74
119.14
114.48
114.21
109.72
53.80
47.12
42.56
40.64
40.43
40.23
40.02
39.81
39.60
39.39
28.96
28.46
23.90
N
N
N
+
O
Br
−
190
180
170
160
150
140
130
120
110
100
90
80
70
60
50
40
30
20
10
ppm

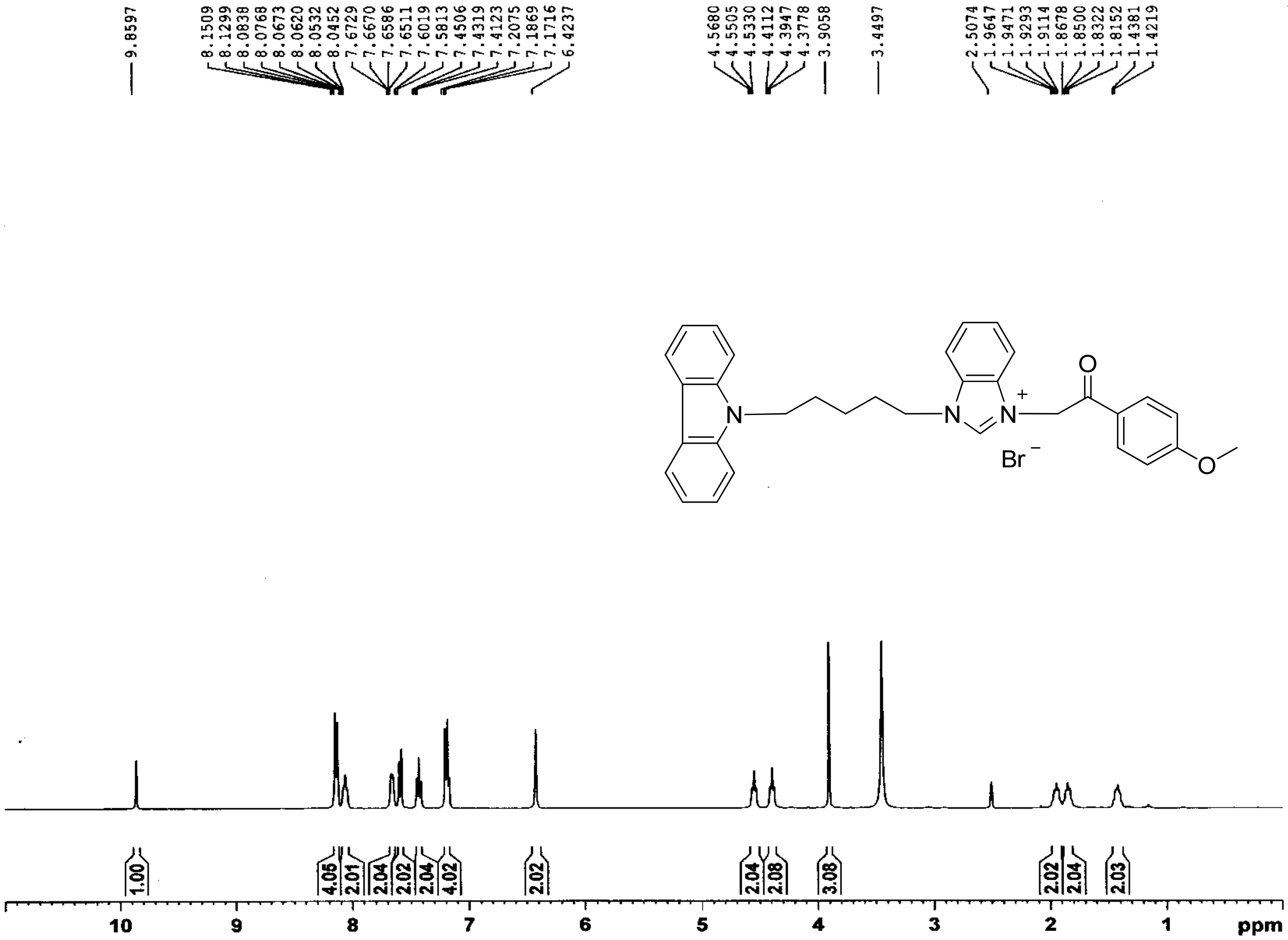

NAME	22012000180
EXPNO	161
PROCNO	1
Date_	20121230
Time	22.47
INSTRUM	spect
PROBHD	5 mm PABBO BB/
PULPROG	zg30
TD	65536
SOLVENT	DMSO
NS	8
DS	0
SWH	8012.820 Hz
FIDRES	0.122266 Hz
AQ	4.0894966 sec
RG	23.2
DW	62.400 usec
DE	6.50 usec
TE	297.0 K
D1	1.00000000 sec
TD0	1

======== CHANNEL f1 ========

SFO1	400.1524711 MHz
NUC1	1H
P1	9.64 usec
SI	65536
SF	400.1500000 MHz
WDW	EM
SSB	0
LB	0.30 Hz
GB	0
PC	1.00

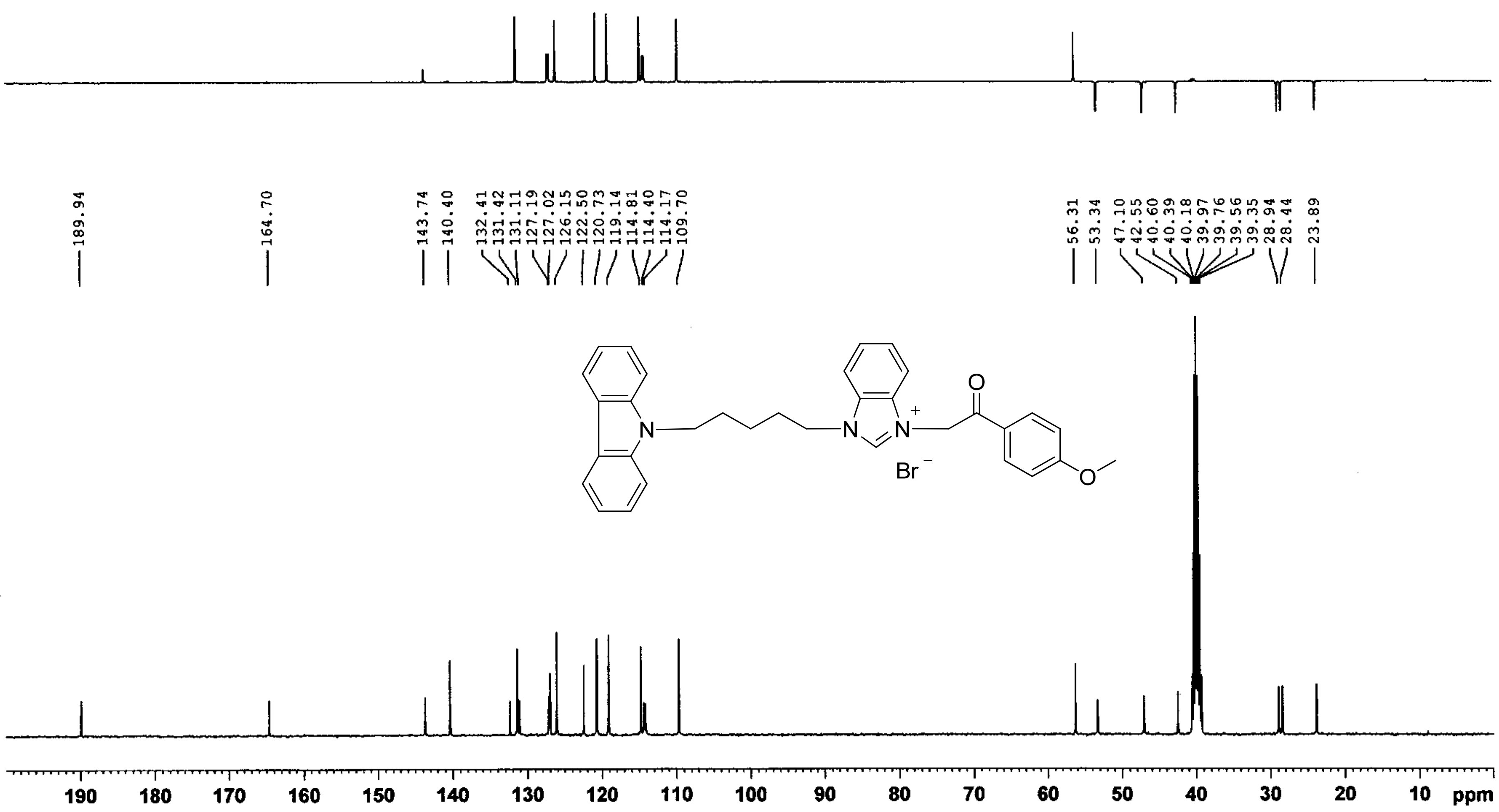
189.94
164.70
143.74
140.40
132.41
131.42
131.11
127.19
127.02
126.15
122.50
120.73
119.14
114.81
114.40
114.17
109.70
56.31
53.34
47.10
42.55
40.60
40.39
40.18
39.97
39.76
39.56
39.35
28.94
28.44
23.89
190
180
170
160
150
140
130
120
110
100
90
80
70
60
50
40
30
20
10
ppm

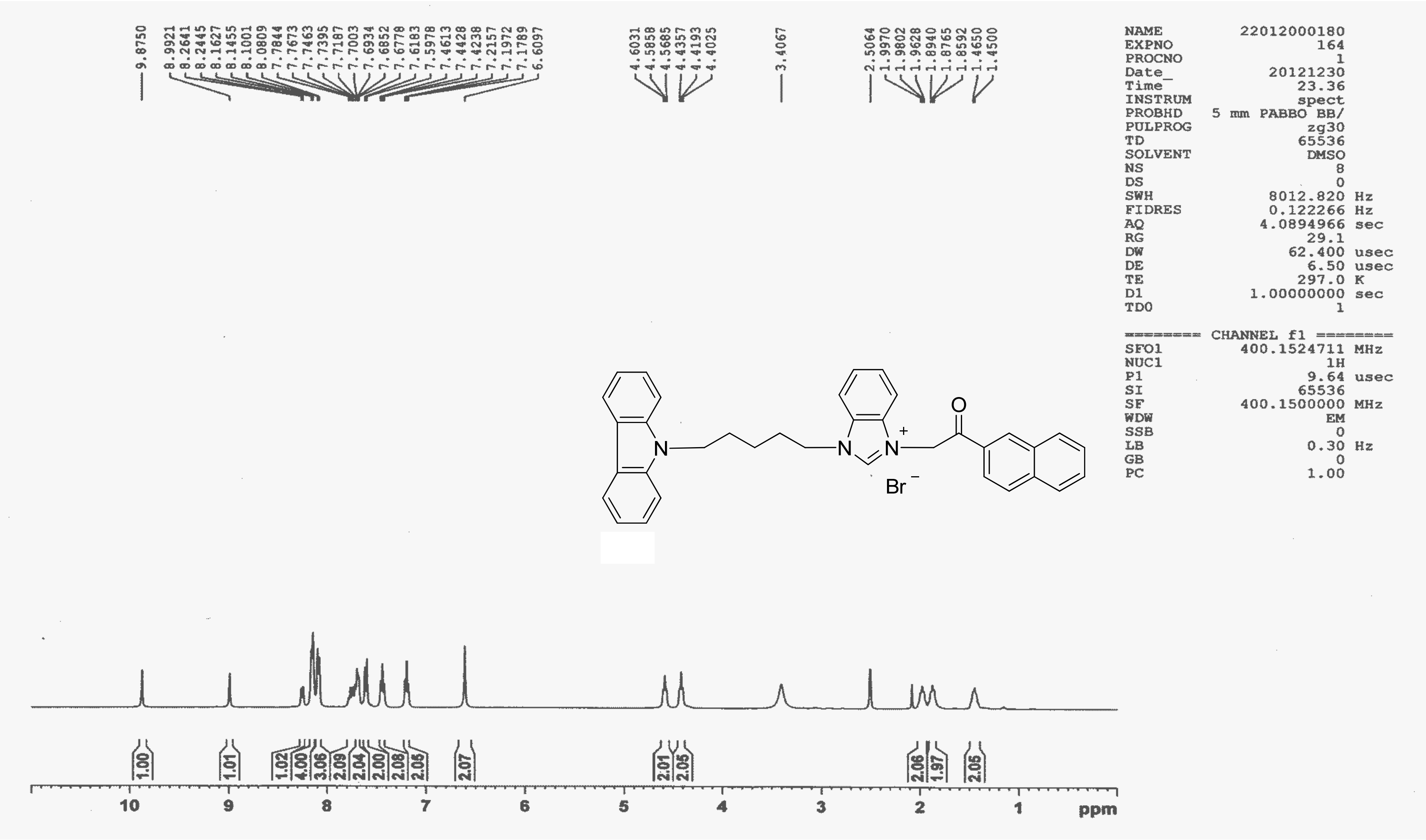

NAME 22012000180
EXPNO 164
PROCNO 1
Date_ 20121230
Time 23.36
INSTRUM spect
PROBHD 5 mm PABBO BB/
PULPROG zg30
TD 65536
SOLVENT DMSO
NS 8
DS 0
SWH 8012.820 Hz
FIDRES 0.122266 Hz
AQ 4.0894966 sec
RG 29.1
DW 62.400 usec
DE 6.50 usec
TE 297.0 K
D1 1.00000000 sec
TD0 1
======== CHANNEL f1 ========
SFO1 400.1524711 MHz
NUC1 1H
P1 9.64 usec
SI 65536
SF 400.1500000 MHz
WDW EM
SSB 0
LB 0.30 Hz
GB 0
PC 1.00
ppm

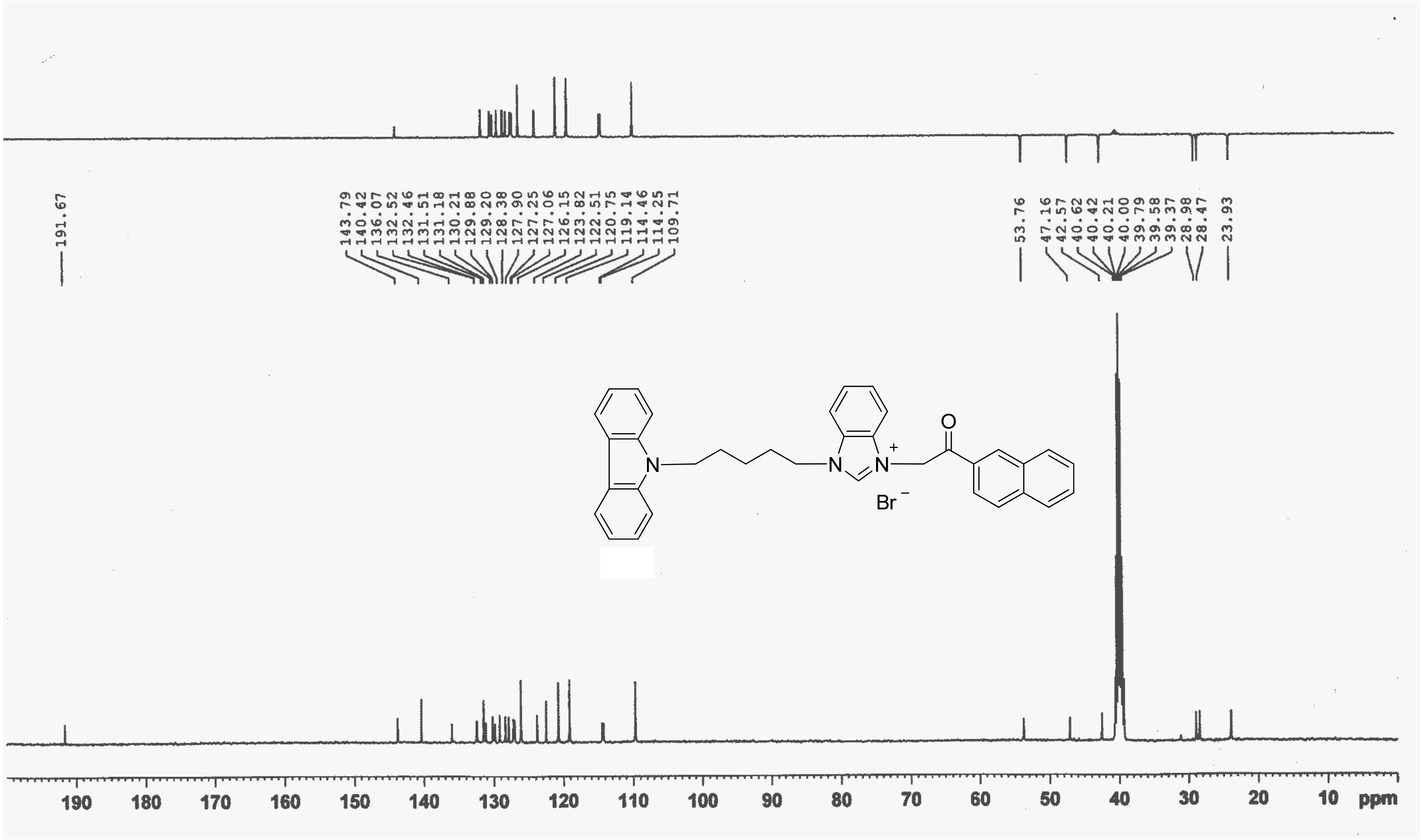

191.67
143.79
140.42
136.07
132.52
132.46
131.51
131.18
130.21
129.88
129.20
128.38
127.90
127.25
127.06
126.15
123.82
122.51
120.75
119.14
114.46
114.25
109.71
53.76
47.16
42.57
40.62
40.42
40.21
40.00
39.79
39.58
39.37
28.98
28.47
23.93
N
N
N
+
O
Br −
190 180 170 160 150 140 130 120 110 100 90 80 70 60 50 40 30 20 10 ppm

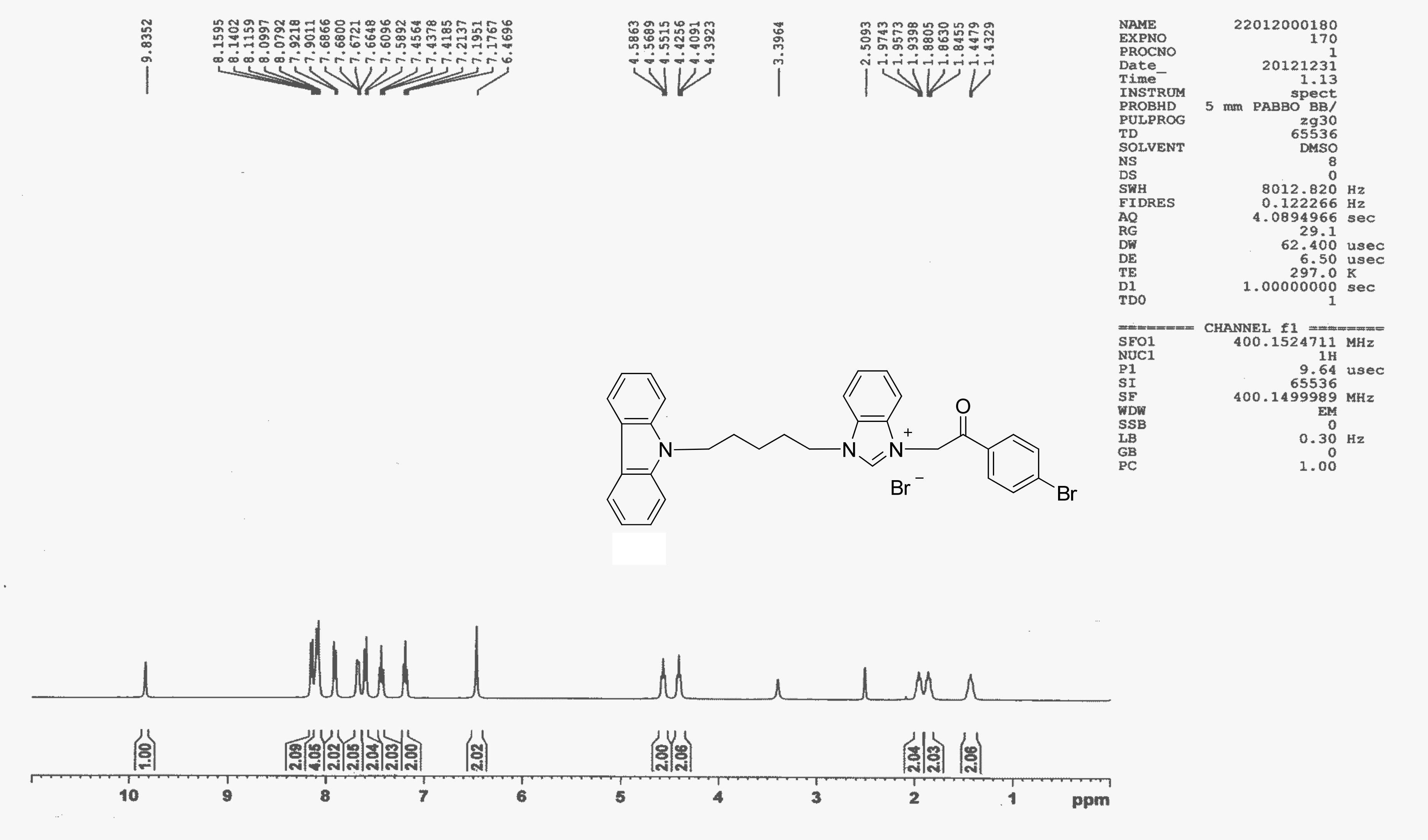
9.8352
8.1595 8.1402 8.1159 8.0997 8.0792 7.9218 7.9011 7.6866 7.6800 7.6721 7.6648 7.6096 7.5892 7.4564 7.4378 7.4185 7.2137 7.1951 7.1767 6.4696
4.5863 4.5689 4.5515 4.4256 4.4091 4.3923
3.3964
2.5093 1.9743 1.9573 1.9398 1.8805 1.8630 1.8455 1.4479 1.4329
NAME 22012000180
EXPNO 170
PROCNO 1
Date_ 20121231
Time 1.13
INSTRUM spect
PROBHD 5 mm PABBO BB/
PULPROG zg30
TD 65536
SOLVENT DMSO
NS 8
DS 0
SWH 8012.820 Hz
FIDRES 0.122266 Hz
AQ 4.0894966 sec
RG 29.1
DW 62.400 usec
DE 6.50 usec
TE 297.0 K
D1 1.00000000 sec
TD0 1
======== CHANNEL f1 ========
SFO1 400.1524711 MHz
NUC1 1H
P1 9.64 usec
SI 65536
SF 400.1499989 MHz
WDW EM
SSB 0
LB 0.30 Hz
GB 0
PC 1.00
N
N
N
O
Br
Br
1.00 2.09 4.05 2.02 2.05 2.04 2.03 2.00 2.02 2.00 2.06 2.04 2.03 2.06
10 9 8 7 6 5 4 3 2 1 ppm

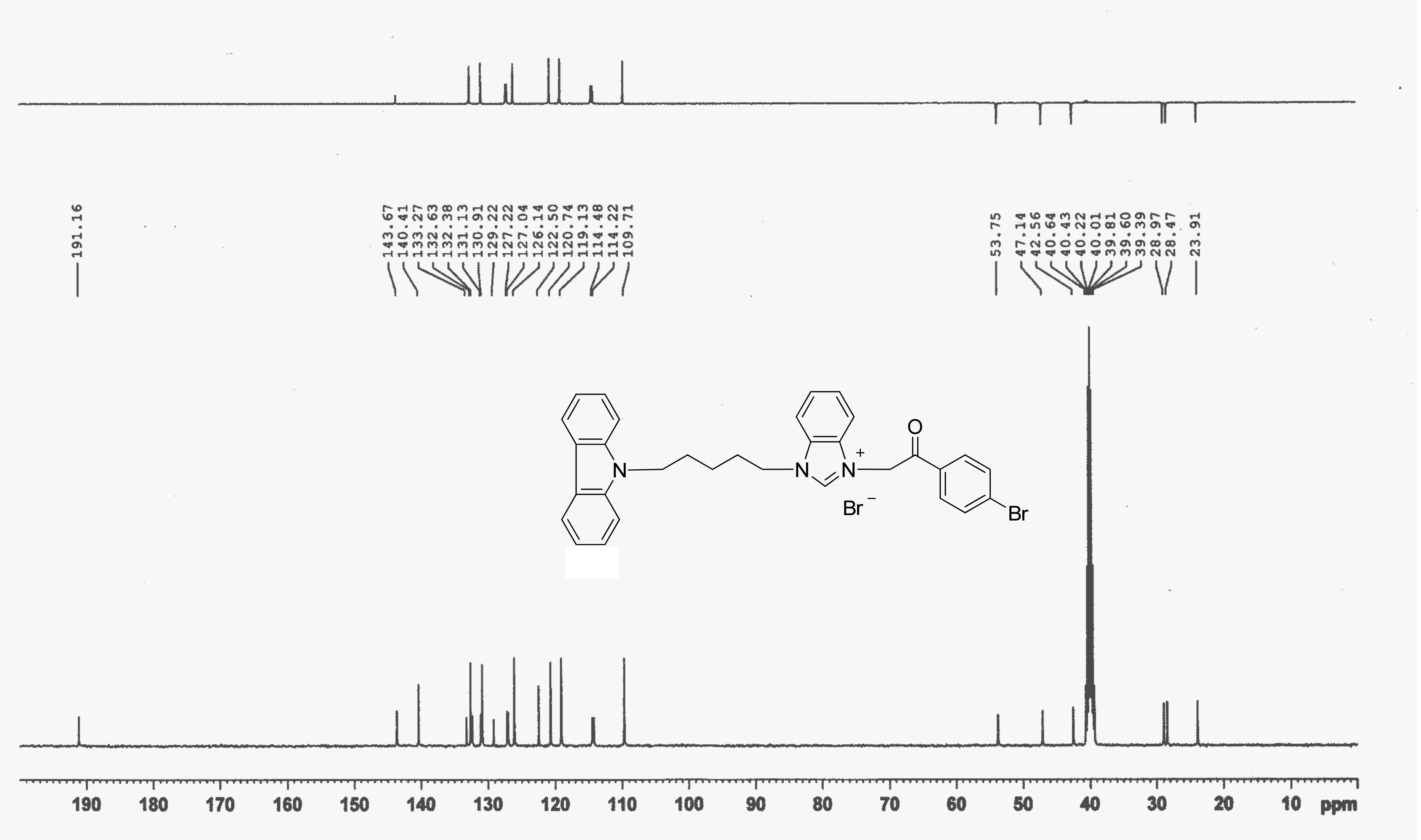
191.16
143.67
140.41
133.27
132.63
132.38
131.13
130.91
129.22
127.22
127.04
126.14
122.50
120.74
119.13
114.48
114.22
109.71
53.75
47.14
42.56
40.64
40.43
40.22
40.01
39.81
39.60
39.39
28.97
28.47
23.91
N
N
N
+
O
Br
Br −
190
180
170
160
150
140
130
120
110
100
90
80
70
60
50
40
30
20
10
ppm

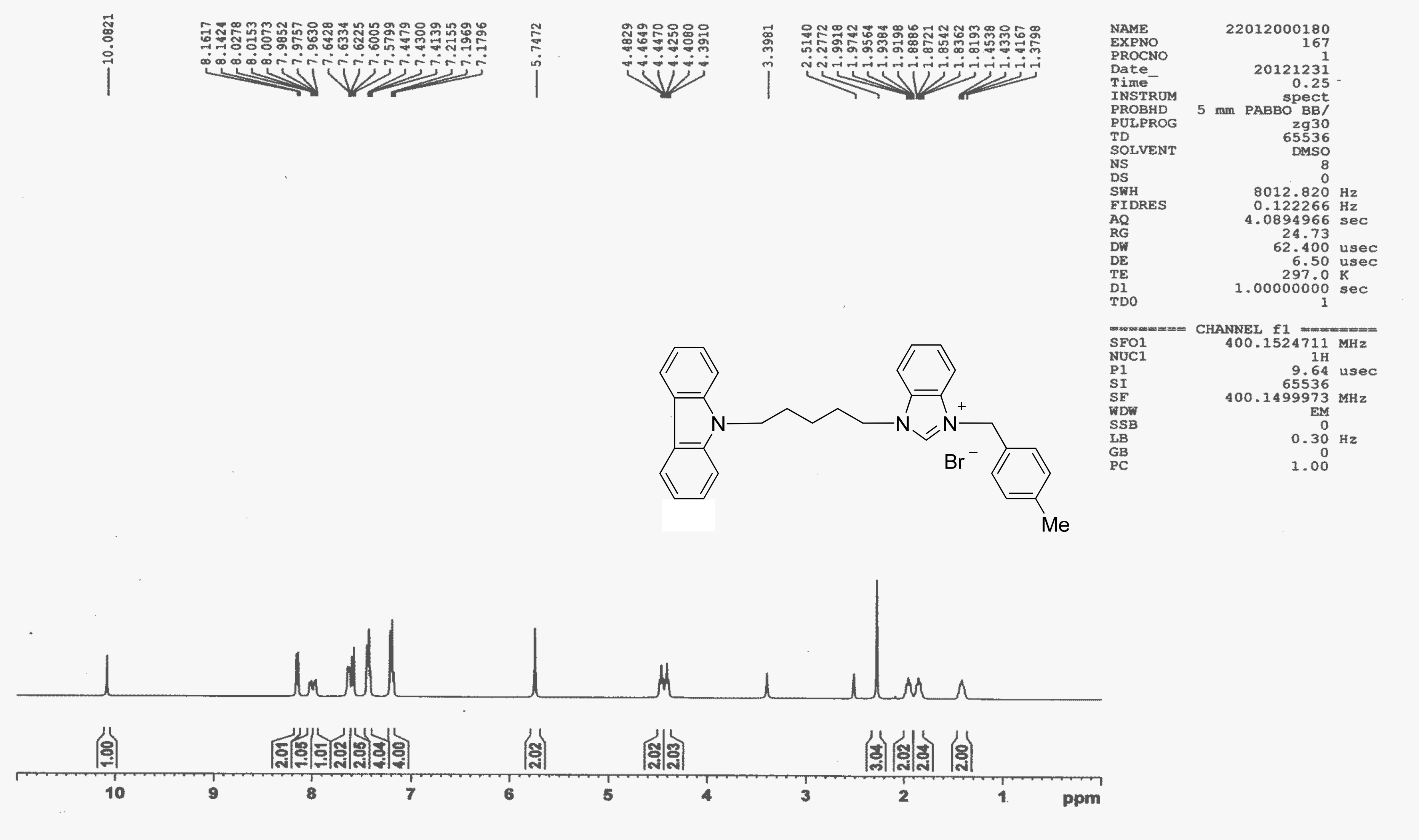

10.0821
8.1617 8.1424 8.0278 8.0153 8.0073 7.9852 7.9757 7.9630 7.6428 7.6334 7.6225 7.6005 7.5799 7.4479 7.4300 7.4139 7.2155 7.1969 7.1796
5.7472
4.4829 4.4649 4.4470 4.4250 4.4080 4.3910
3.3981
2.5140 2.2772 1.9918 1.9742 1.9564 1.9384 1.9198 1.8886 1.8721 1.8542 1.8362 1.8193 1.4538 1.4330 1.4167 1.3798
NAME 22012000180
EXPNO 167
PROCNO 1
Date_ 20121231
Time 0.25
INSTRUM spect
PROBHD 5 mm PABBO BB/
PULPROG zg30
TD 65536
SOLVENT DMSO
NS 8
DS 0
SWH 8012.820 Hz
FIDRES 0.122266 Hz
AQ 4.0894966 sec
RG 24.73
DW 62.400 usec
DE 6.50 usec
TE 297.0 K
D1 1.00000000 sec
TD0 1
======== CHANNEL f1 ========
SFO1 400.1524711 MHz
NUC1 1H
P1 9.64 usec
SI 65536
SF 400.1499973 MHz
WDW EM
SSB 0
LB 0.30 Hz
GB 0
PC 1.00
N
N
N
+
Br −
Me
1.00 2.01 1.05 1.01 2.02 2.05 4.04 4.00 2.02 2.02 2.03 3.04 2.02 2.04 2.00
10 9 8 7 6 5 4 3 2 1 ppm

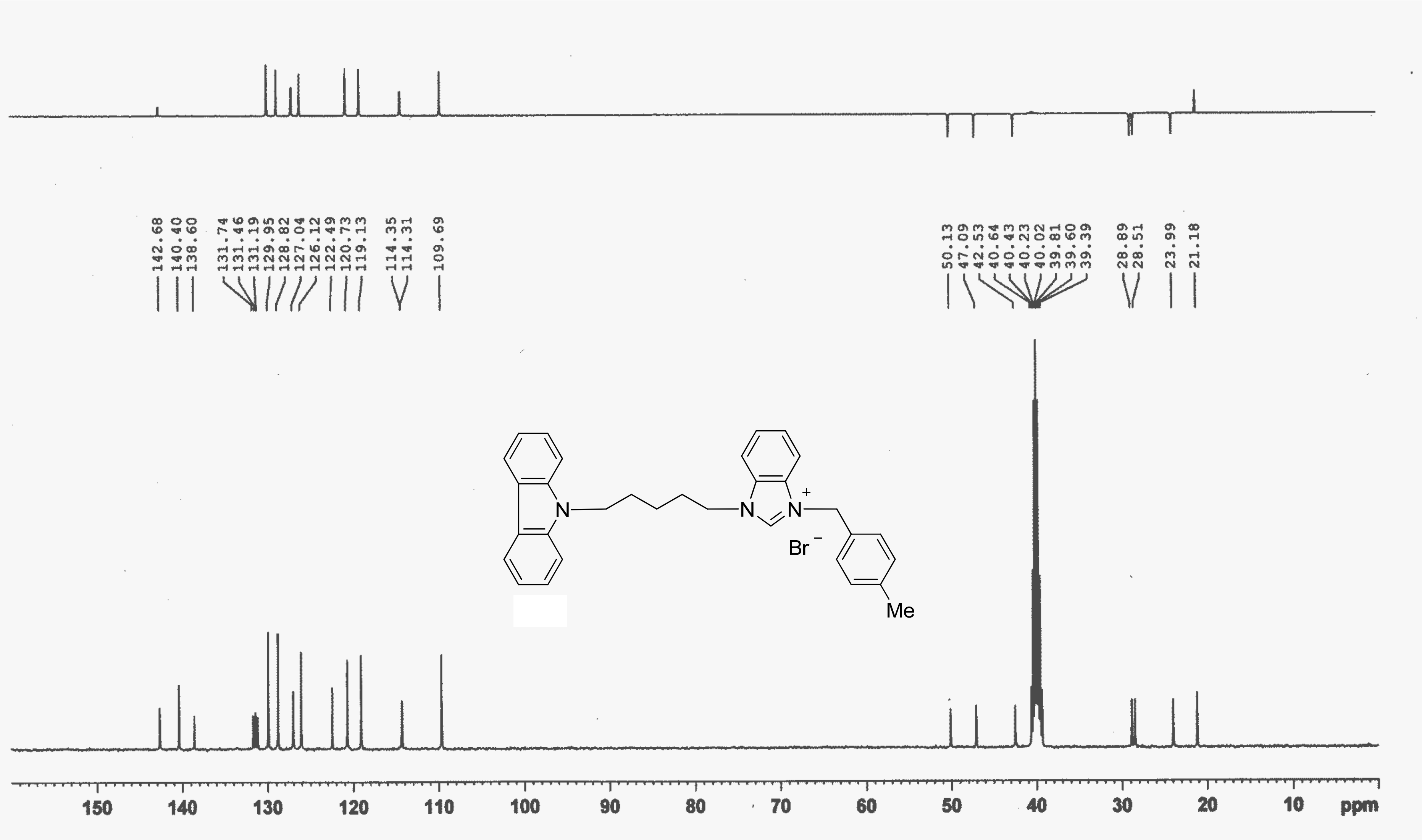
142.68
140.40
138.60
131.74
131.46
131.19
129.95
128.82
127.04
126.12
122.49
120.73
119.13
114.35
114.31
109.69
50.13
47.09
42.53
40.64
40.43
40.23
40.02
39.81
39.60
39.39
28.89
28.51
23.99
21.18
N
N
N
+
Br −
Me
150
140
130
120
110
100
90
80
70
60
50
40
30
20
10
ppm

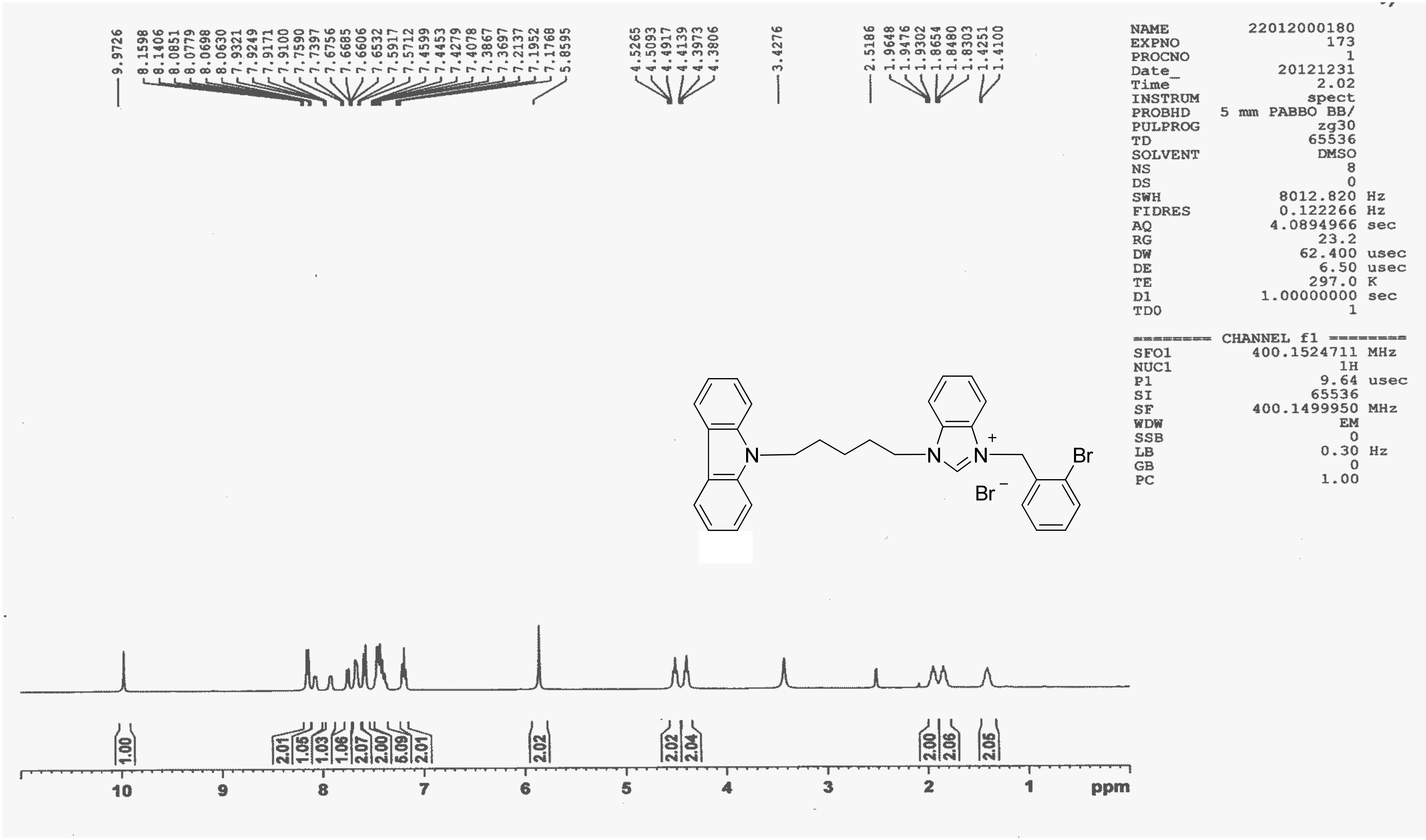
9.9726
8.1598
8.1406
8.0851
8.0779
8.0698
8.0630
7.9321
7.9249
7.9171
7.9100
7.7590
7.7397
7.6756
7.6685
7.6606
7.6532
7.5917
7.5712
7.4599
7.4453
7.4279
7.4078
7.3867
7.3697
7.2137
7.1952
7.1768
5.8595
4.5265
4.5093
4.4917
4.4139
4.3973
4.3806
3.4276
2.5186
1.9648
1.9476
1.9302
1.8654
1.8480
1.8303
1.4251
1.4100
NAME 22012000180
EXPNO 173
PROCNO 1
Date_ 20121231
Time 2.02
INSTRUM spect
PROBHD 5 mm PABBO BB/
PULPROG zg30
TD 65536
SOLVENT DMSO
NS 8
DS 0
SWH 8012.820 Hz
FIDRES 0.122266 Hz
AQ 4.0894966 sec
RG 23.2
DW 62.400 usec
DE 6.50 usec
TE 297.0 K
D1 1.00000000 sec
TD0 1
======== CHANNEL f1 ========
SFO1 400.1524711 MHz
NUC1 1H
P1 9.64 usec
SI 65536
SF 400.1499950 MHz
WDW EM
SSB 0
LB 0.30 Hz
GB 0
PC 1.00
N
N
N
+
Br
Br −
1.00
2.01
1.05
1.03
1.06
2.07
2.00
6.09
2.01
2.02
2.02
2.04
2.00
2.06
2.05
10
9
8
7
6
5
4
3
2
1
ppm

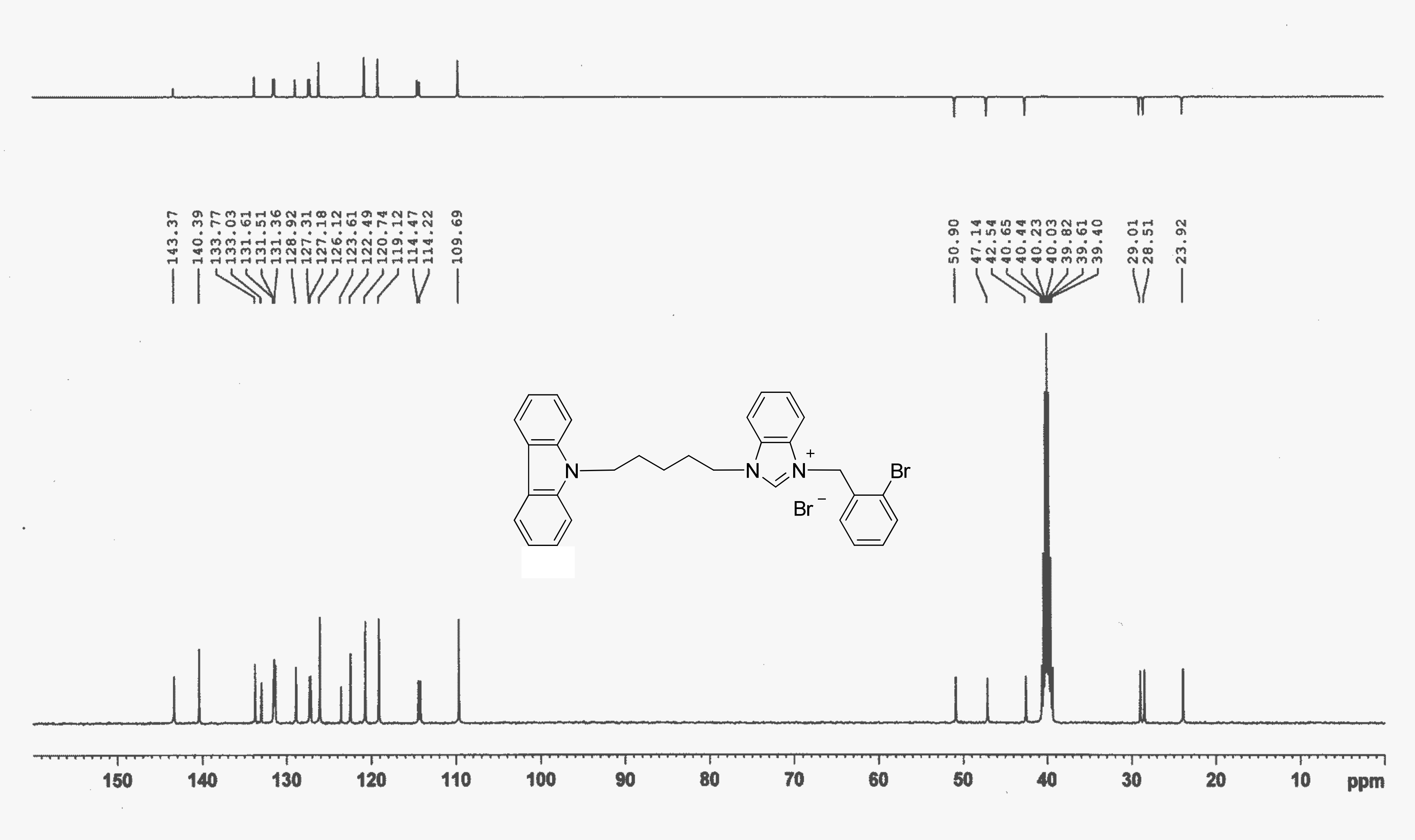
143.37
140.39
133.77
133.03
131.61
131.51
131.36
128.92
127.31
127.18
126.12
123.61
122.49
120.74
119.12
114.47
114.22
109.69
50.90
47.14
42.54
40.65
40.44
40.23
40.03
39.82
39.61
39.40
29.01
28.51
23.92
N
N
N
+
Br
Br
−
150
140
130
120
110
100
90
80
70
60
50
40
30
20
10
ppm

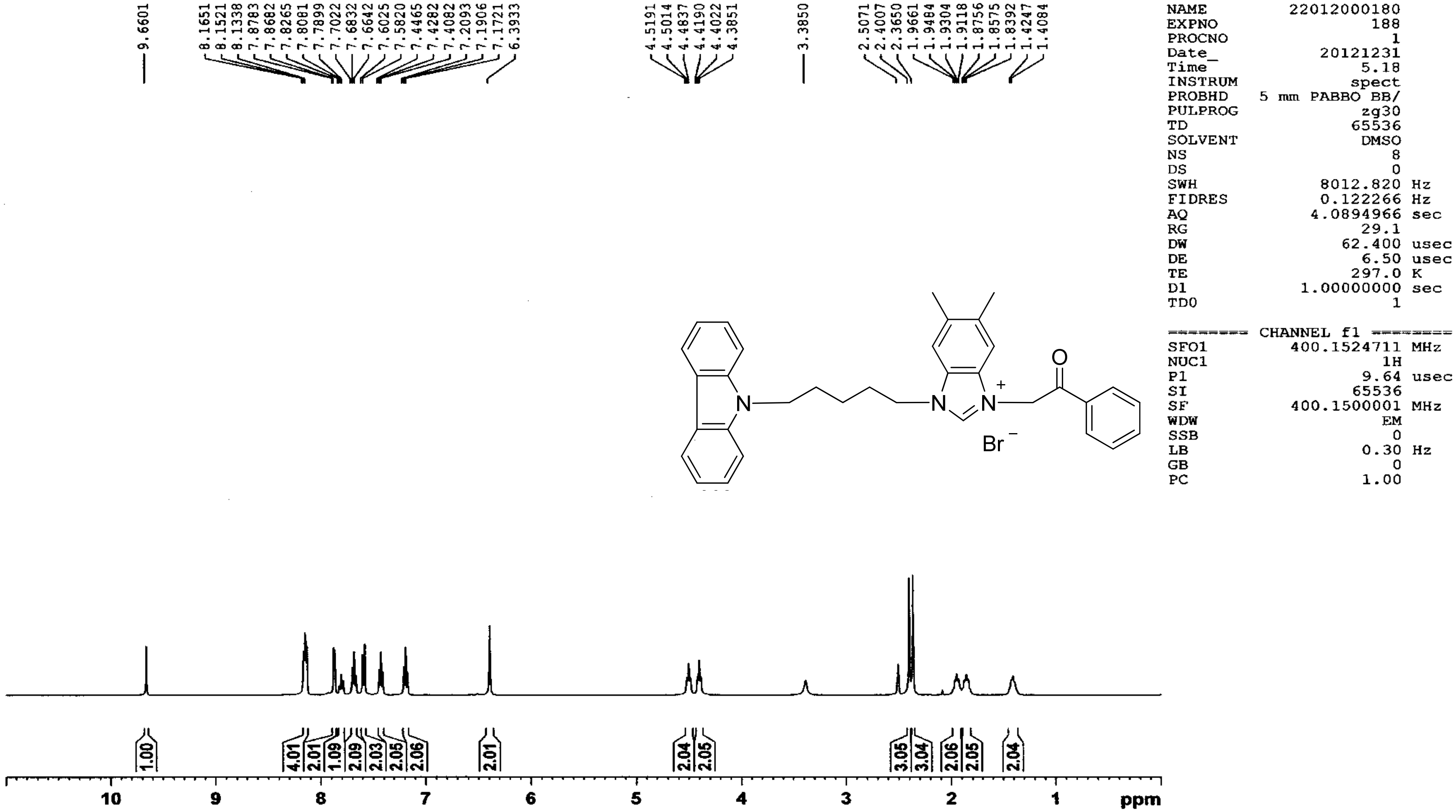
9.6601
8.1651 8.1521 8.1338 7.8783 7.8682 7.8265 7.8081 7.7899 7.7022 7.6832 7.6642 7.6025 7.5820 7.4465 7.4282 7.4082 7.2093 7.1906 7.1721 6.3933
4.5191 4.5014 4.4837 4.4190 4.4022 4.3851
3.3850
2.5071 2.4007 2.3650 1.9661 1.9484 1.9304 1.9118 1.8756 1.8575 1.8392 1.4247 1.4084
NAME 22012000180
EXPNO 188
PROCNO 1
Date_ 20121231
Time 5.18
INSTRUM spect
PROBHD 5 mm PABBO BB/
PULPROG zg30
TD 65536
SOLVENT DMSO
NS 8
DS 0
SWH 8012.820 Hz
FIDRES 0.122266 Hz
AQ 4.0894966 sec
RG 29.1
DW 62.400 usec
DE 6.50 usec
TE 297.0 K
D1 1.00000000 sec
TD0 1
======== CHANNEL f1 ========
SFO1 400.1524711 MHz
NUC1 1H
P1 9.64 usec
SI 65536
SF 400.1500001 MHz
WDW EM
SSB 0
LB 0.30 Hz
GB 0
PC 1.00
N
N
N
O
Br⁻
1.00 4.01 2.01 1.09 2.09 2.03 2.05 2.06 2.01 2.04 2.05 3.05 3.04 2.06 2.05 2.04
10 9 8 7 6 5 4 3 2 1 ppm

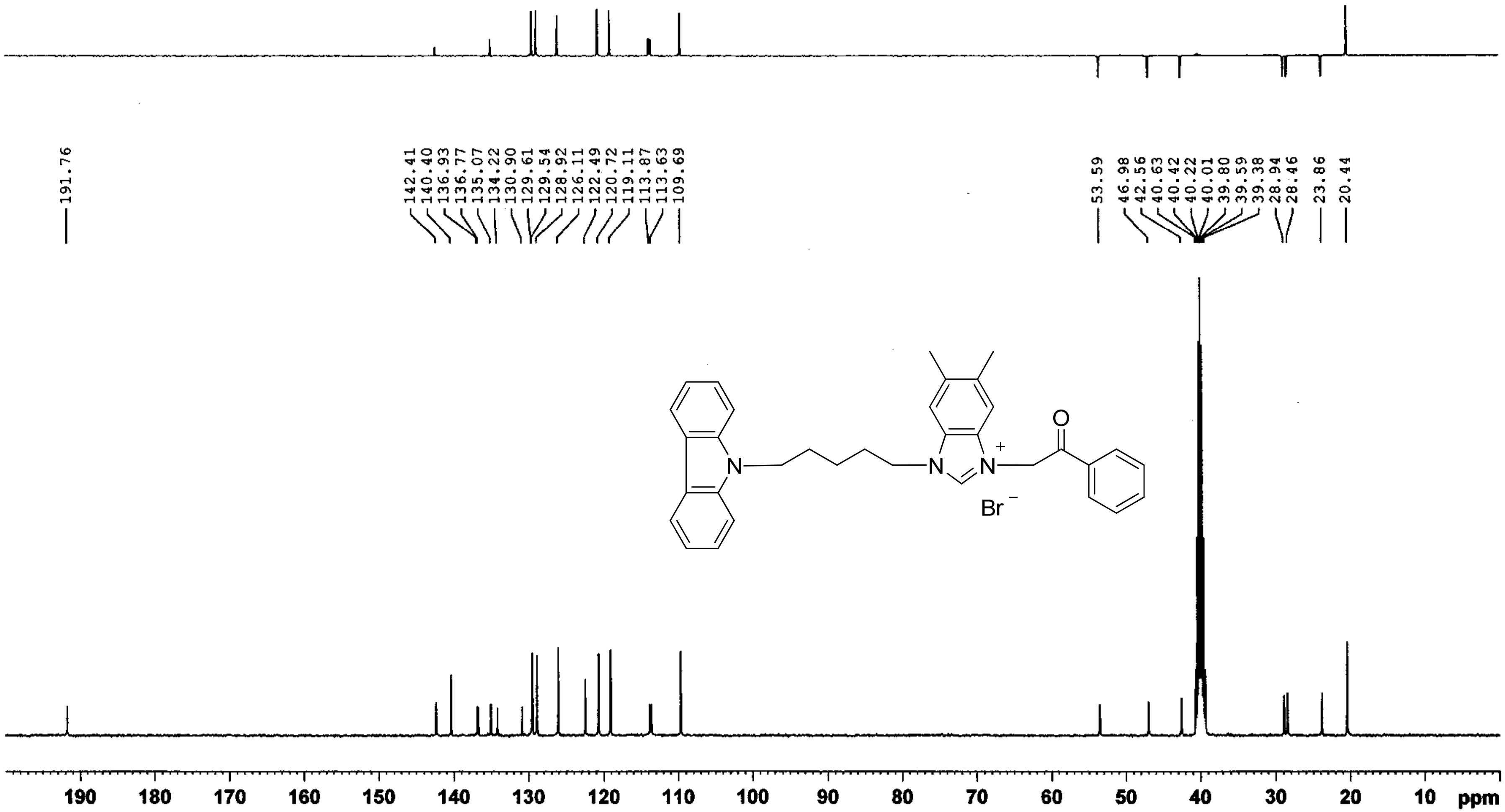
191.76
142.41
140.40
136.93
136.77
135.07
134.22
130.90
129.61
129.54
128.92
126.11
122.49
120.72
119.11
113.87
113.63
109.69
53.59
46.98
42.56
40.63
40.42
40.22
40.01
39.80
39.59
39.38
28.94
28.46
23.86
20.44
N
N
N
+
O
Br −
190
180
170
160
150
140
130
120
110
100
90
80
70
60
50
40
30
20
10
ppm

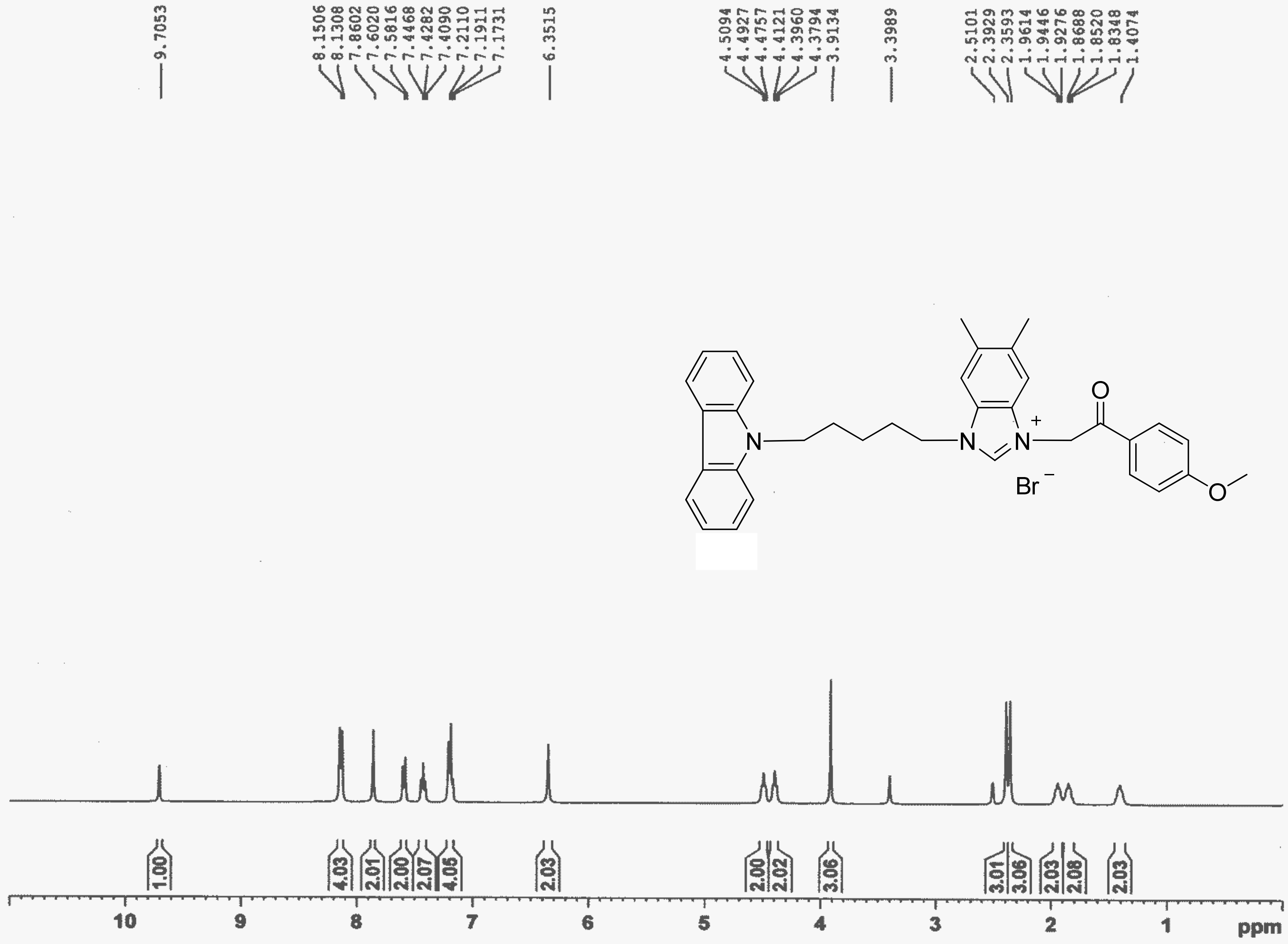

NAME	22012000180	
EXPNO	176	
PROCNO	1	
Date_	20121230	
Time	21.35	
INSTRUM	spect	
PROBHD	5 mm PABBO BB/	
PULPROG	zg30	
TD	65536	
SOLVENT	DMSO	
NS	8	
DS	0	
SWH	8012.820	Hz
FIDRES	0.122266	Hz
AQ	4.0894966	sec
RG	23.2	
DW	62.400	usec
DE	6.50	usec
TE	297.0	K
D1	1.00000000	sec
TD0	1	

======== CHANNEL f1 ========

SFO1	400.1524711	MHz
NUC1	1H	
P1	9.64	usec
SI	65536	
SF	400.1499987	MHz
WDW	EM	
SSB	0	
LB	0.30	Hz
GB	0	
PC	1.00	

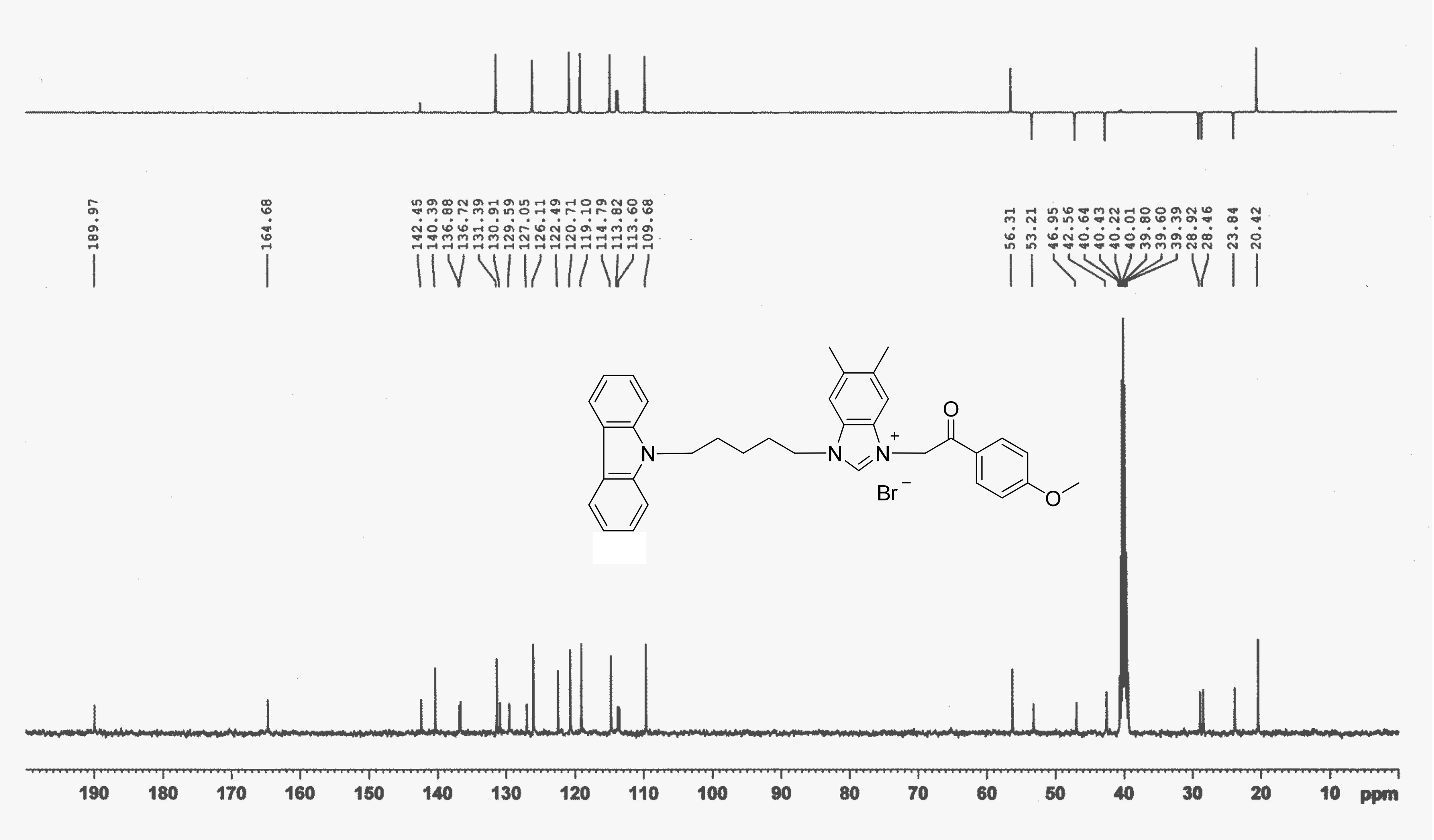
189.97
164.68
142.45
140.39
136.88
136.72
131.39
130.91
129.59
127.05
126.11
122.49
120.71
119.10
114.79
113.82
113.60
109.68
56.31
53.21
46.95
42.56
40.64
40.43
40.22
40.01
39.80
39.60
39.39
28.92
28.46
23.84
20.42
Br
190
180
170
160
150
140
130
120
110
100
90
80
70
60
50
40
30
20
10
ppm

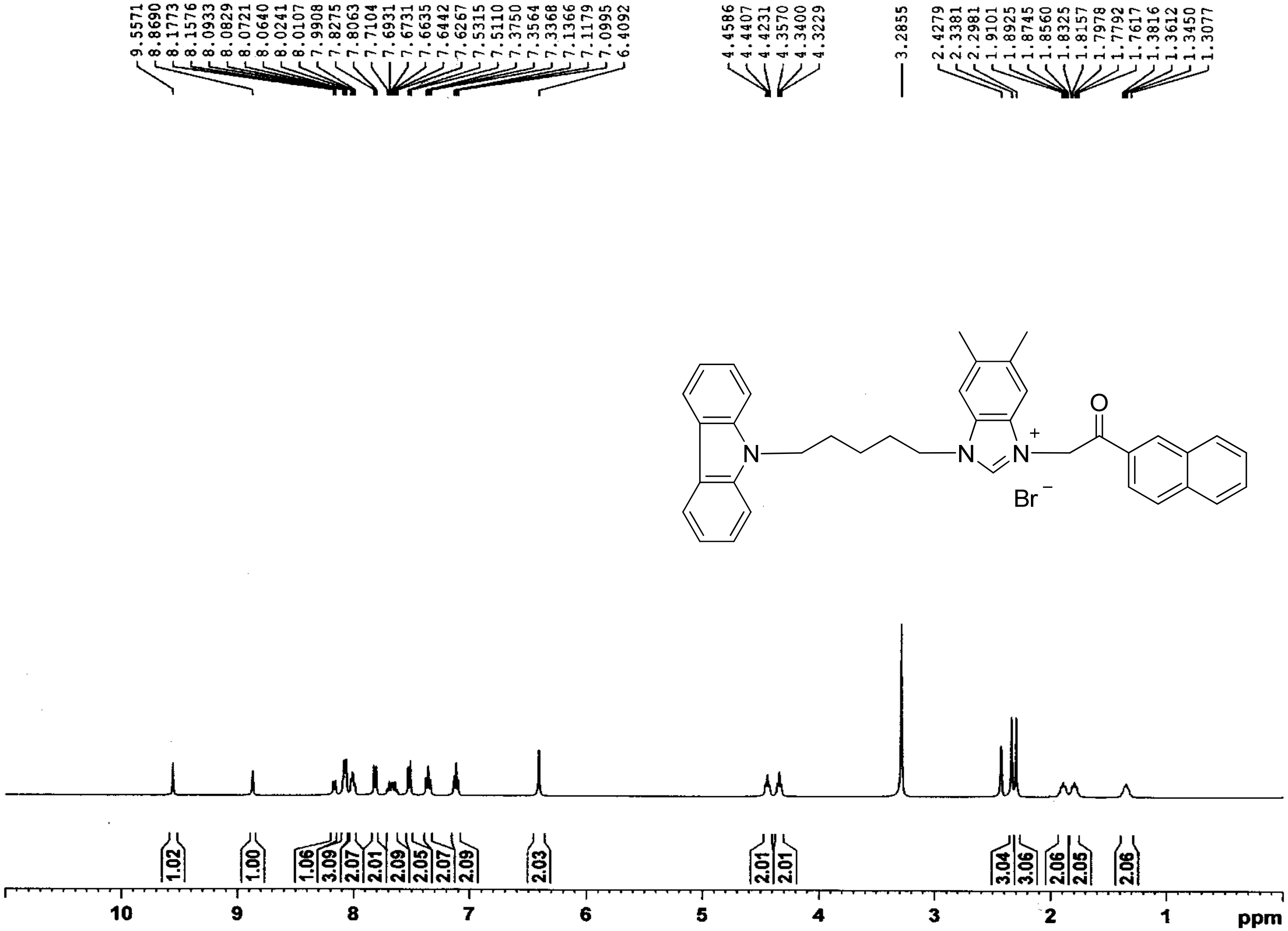

NAME	22012000180	
EXPNO	182	
PROCNO	1	
Date_	20121231	
Time	3.40	
INSTRUM	spect	
PROBHD	5 mm PABBO BB/	
PULPROG	zg30	
TD	65536	
SOLVENT	DMSO	
NS	8	
DS	0	
SWH	8012.820	Hz
FIDRES	0.122266	Hz
AQ	4.0894966	sec
RG	36.33	
DW	62.400	usec
DE	6.50	usec
TE	297.0	K
D1	1.00000000	sec
TD0	1	

======== CHANNEL f1 ========

SFO1	400.1524711	MHz
NUC1	1H	
P1	9.64	usec
SI	65536	
SF	400.1500316	MHz
WDW	EM	
SSB	0	
LB	0.30	Hz
GB	0	
PC	1.00	

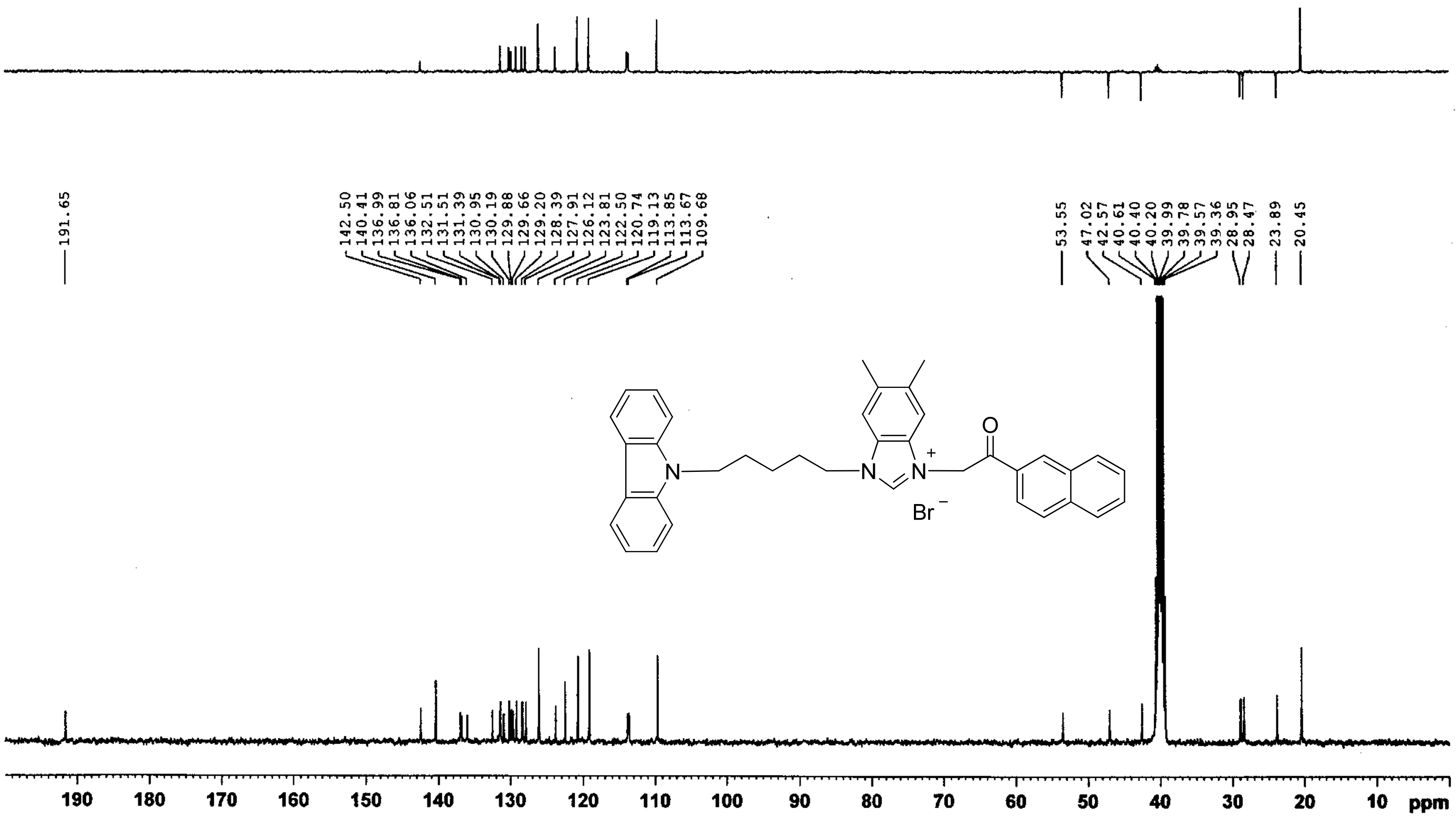
191.65
142.50
140.41
136.99
136.81
136.06
132.51
131.51
131.39
130.95
130.19
129.88
129.66
129.20
128.39
127.91
126.12
123.81
122.50
120.74
119.13
113.85
113.67
109.68
53.55
47.02
42.57
40.61
40.40
40.20
39.99
39.78
39.57
39.36
28.95
28.47
23.89
20.45
N
N
N
O
Br
190 180 170 160 150 140 130 120 110 100 90 80 70 60 50 40 30 20 10 ppm

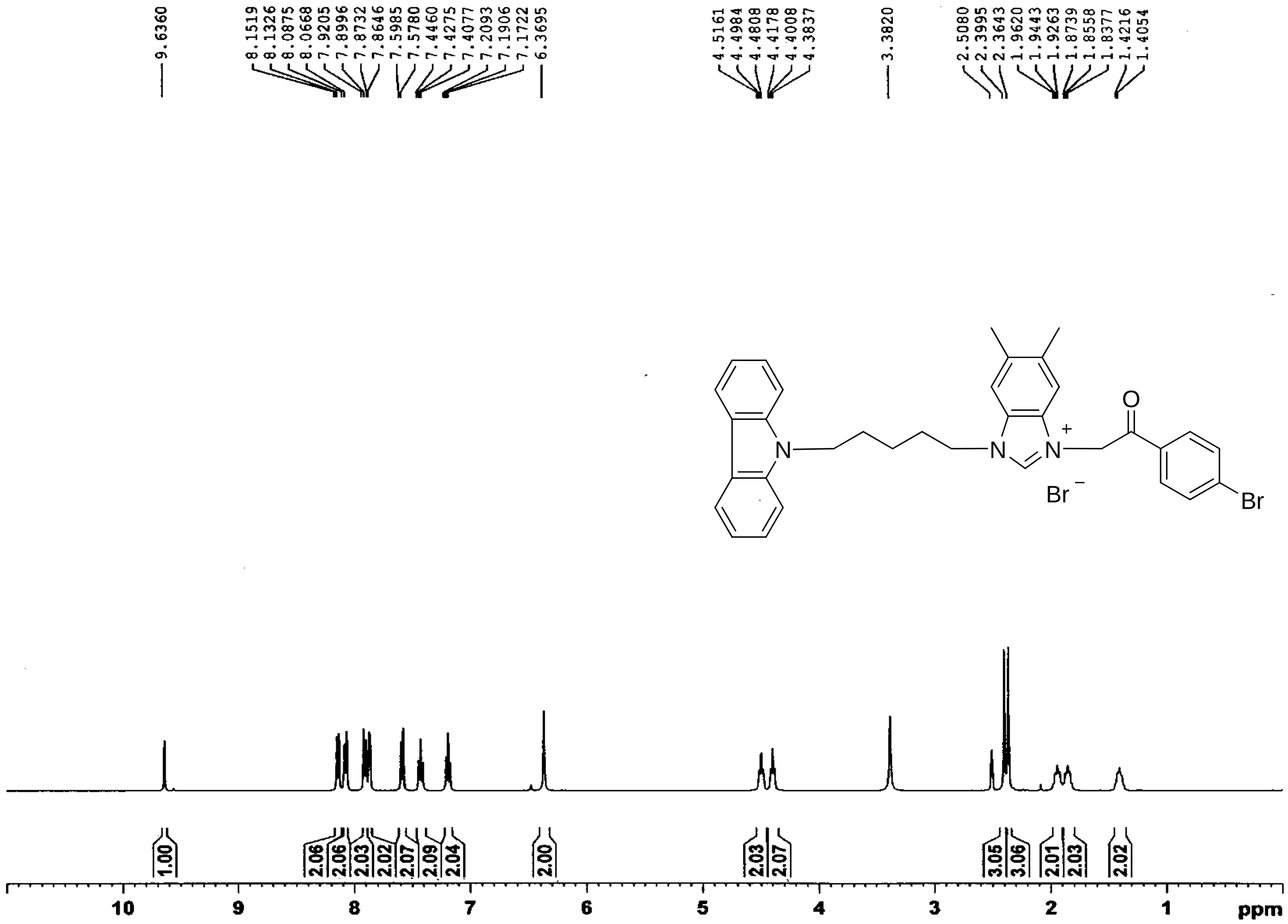

NAME	22012000180
EXPNO	185
PROCNO	1
Date_	20121231
Time	4.29
INSTRUM	spect
PROBHD	5 mm PABBO BB/
PULPROG	zg30
TD	65536
SOLVENT	DMSO
NS	8
DS	0
SWH	8012.820 Hz
FIDRES	0.122266 Hz
AQ	4.0894966 sec
RG	31.56
DW	62.400 usec
DE	6.50 usec
TE	297.0 K
D1	1.00000000 sec
TD0	1

======== CHANNEL f1 ========

SFO1	400.1524711 MHz
NUC1	1H
P1	9.64 usec
SI	65536
SF	400.1499997 MHz
WDW	EM
SSB	0
LB	0.30 Hz
GB	0
PC	1.00

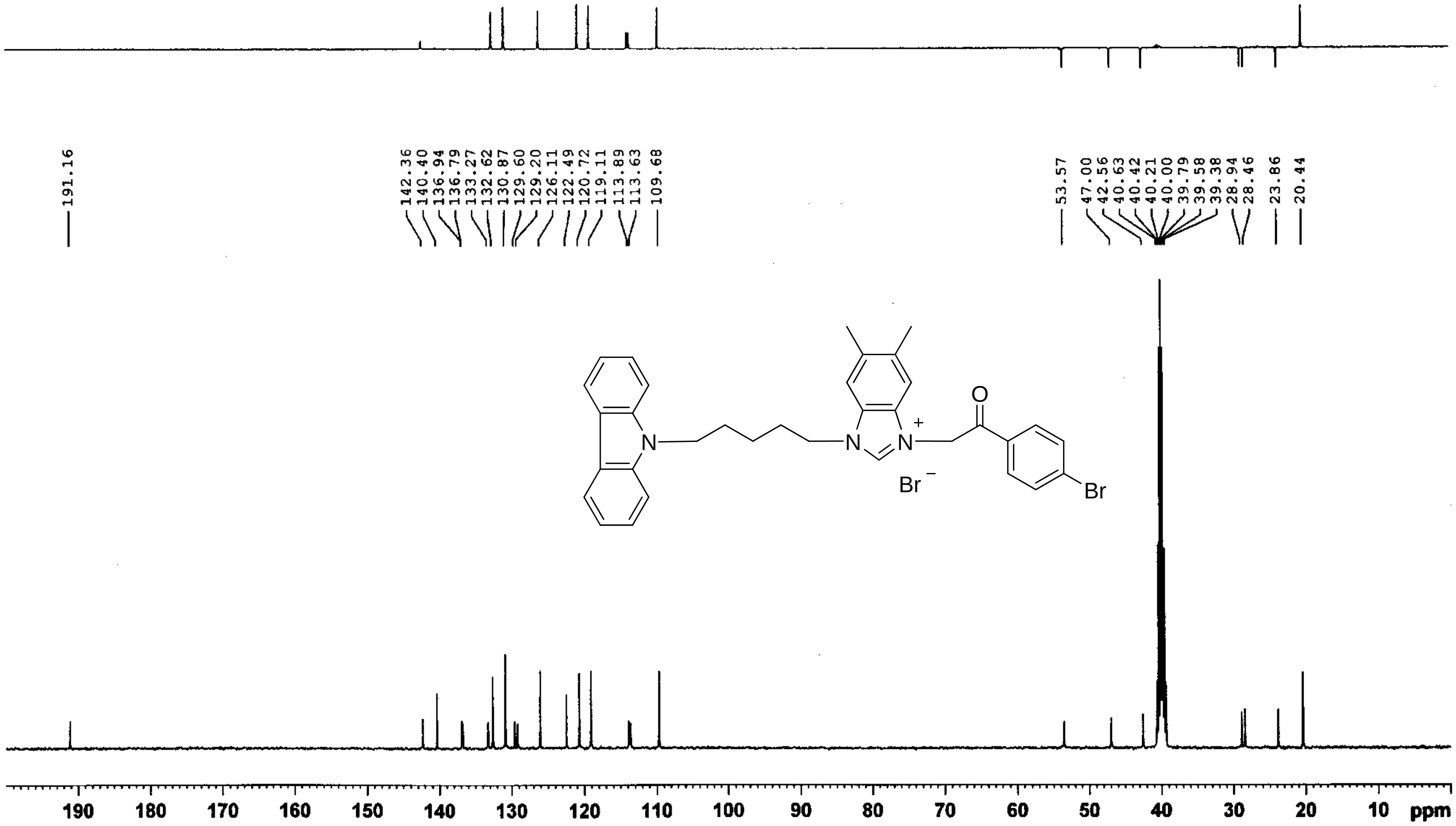
191.16
142.36
140.40
136.94
136.79
133.27
132.62
130.87
129.60
129.20
126.11
122.49
120.72
119.11
113.89
113.63
109.68
53.57
47.00
42.56
40.63
40.42
40.21
40.00
39.79
39.58
39.38
28.94
28.46
23.86
20.44
N
N
N
O
Br
Br
190 180 170 160 150 140 130 120 110 100 90 80 70 60 50 40 30 20 10 ppm

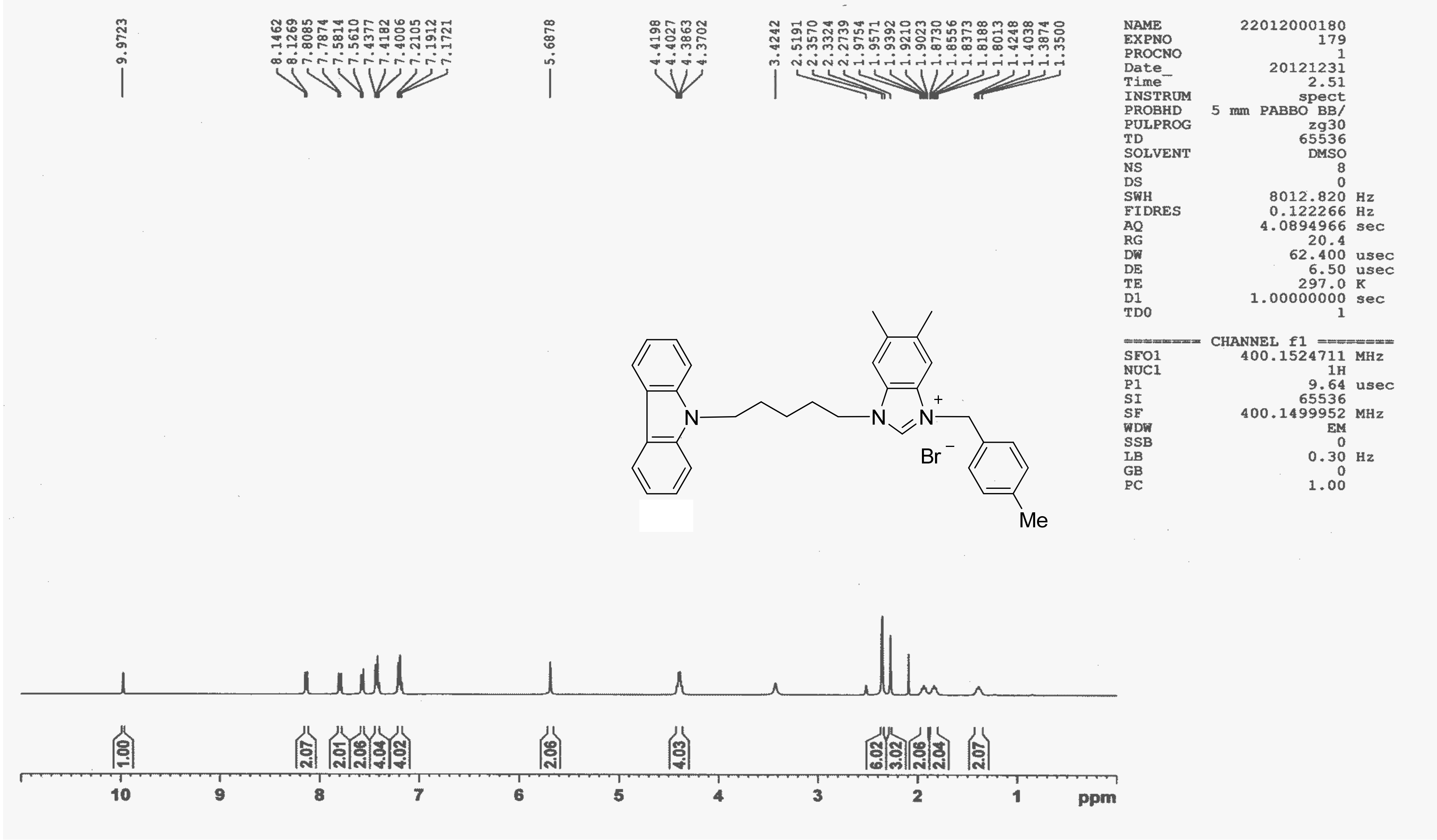

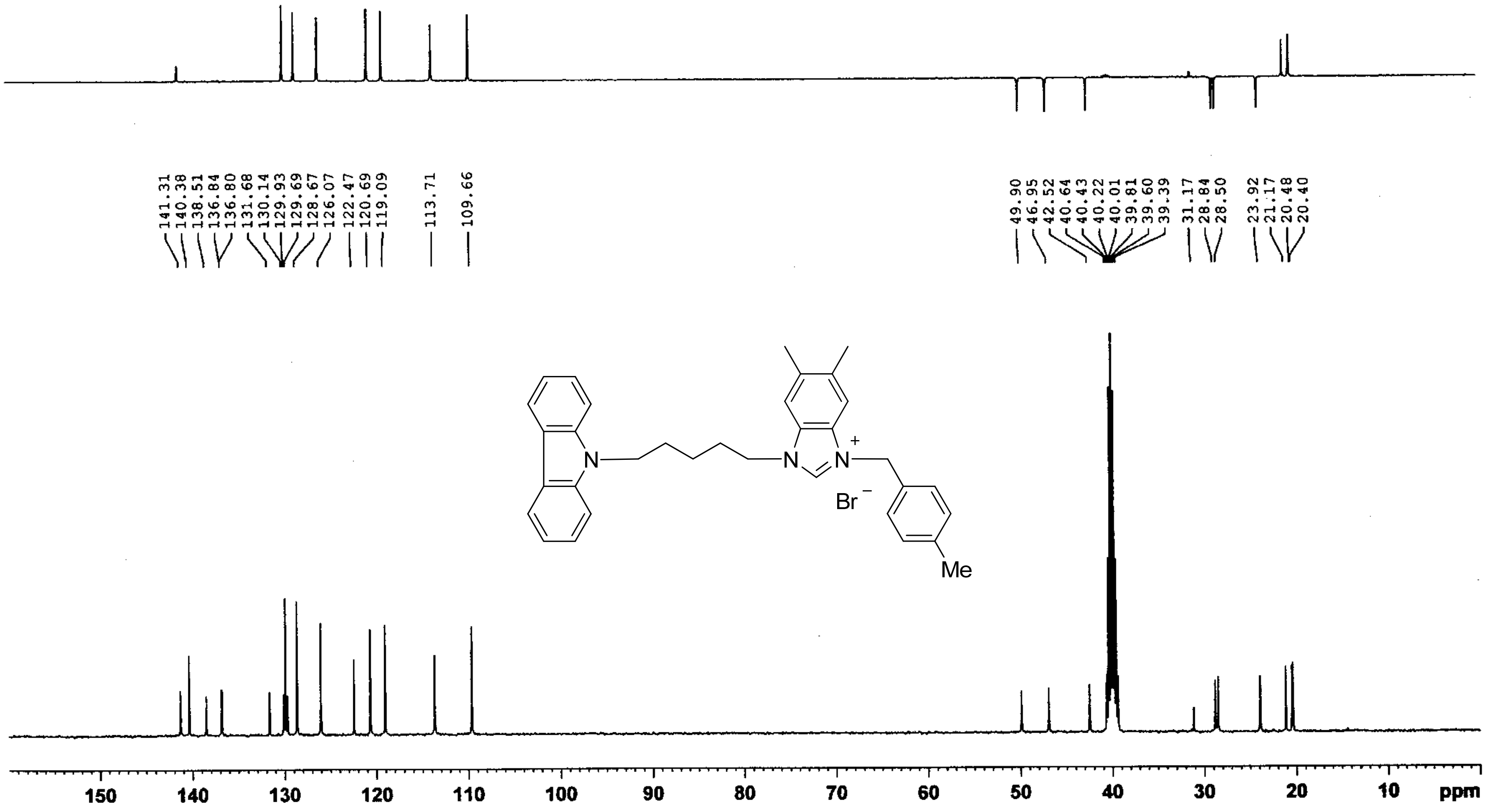
141.31
140.38
138.51
136.84
136.80
131.68
130.14
129.93
129.69
128.67
126.07
122.47
120.69
119.09
113.71
109.66
49.90
46.95
42.52
40.64
40.43
40.22
40.01
39.81
39.60
39.39
31.17
28.84
28.50
23.92
21.17
20.48
20.40
N
N
N
+
Br −
Me
150
140
130
120
110
100
90
80
70
60
50
40
30
20
10
ppm

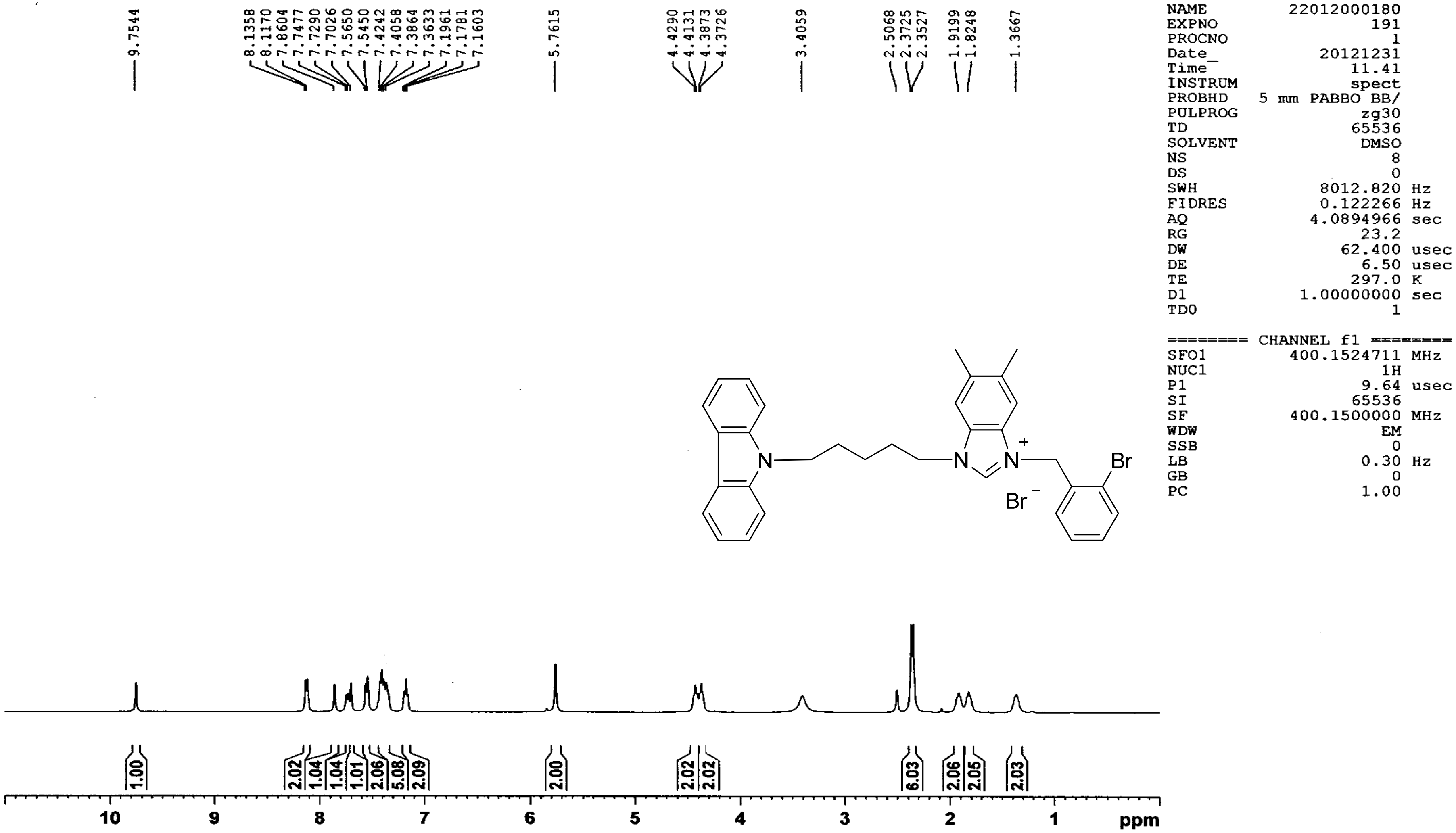
9.7544
8.1358
8.1170
7.8604
7.7477
7.7290
7.7026
7.5650
7.5450
7.4242
7.4058
7.3864
7.3633
7.1961
7.1781
7.1603
5.7615
4.4290
4.4131
4.3873
4.3726
3.4059
2.5068
2.3725
2.3527
1.9199
1.8248
1.3667
NAME 22012000180
EXPNO 191
PROCNO 1
Date_ 20121231
Time 11.41
INSTRUM spect
PROBHD 5 mm PABBO BB/
PULPROG zg30
TD 65536
SOLVENT DMSO
NS 8
DS 0
SWH 8012.820 Hz
FIDRES 0.122266 Hz
AQ 4.0894966 sec
RG 23.2
DW 62.400 usec
DE 6.50 usec
TE 297.0 K
D1 1.00000000 sec
TD0 1
======== CHANNEL f1 ========
SFO1 400.1524711 MHz
NUC1 1H
P1 9.64 usec
SI 65536
SF 400.1500000 MHz
WDW EM
SSB 0
LB 0.30 Hz
GB 0
PC 1.00
N
N
N+
Br
Br−
1.00
2.02
1.04
1.04
1.01
2.06
5.08
2.09
2.00
2.02
2.02
6.03
2.06
2.05
2.03
10
9
8
7
6
5
4
3
2
1
ppm

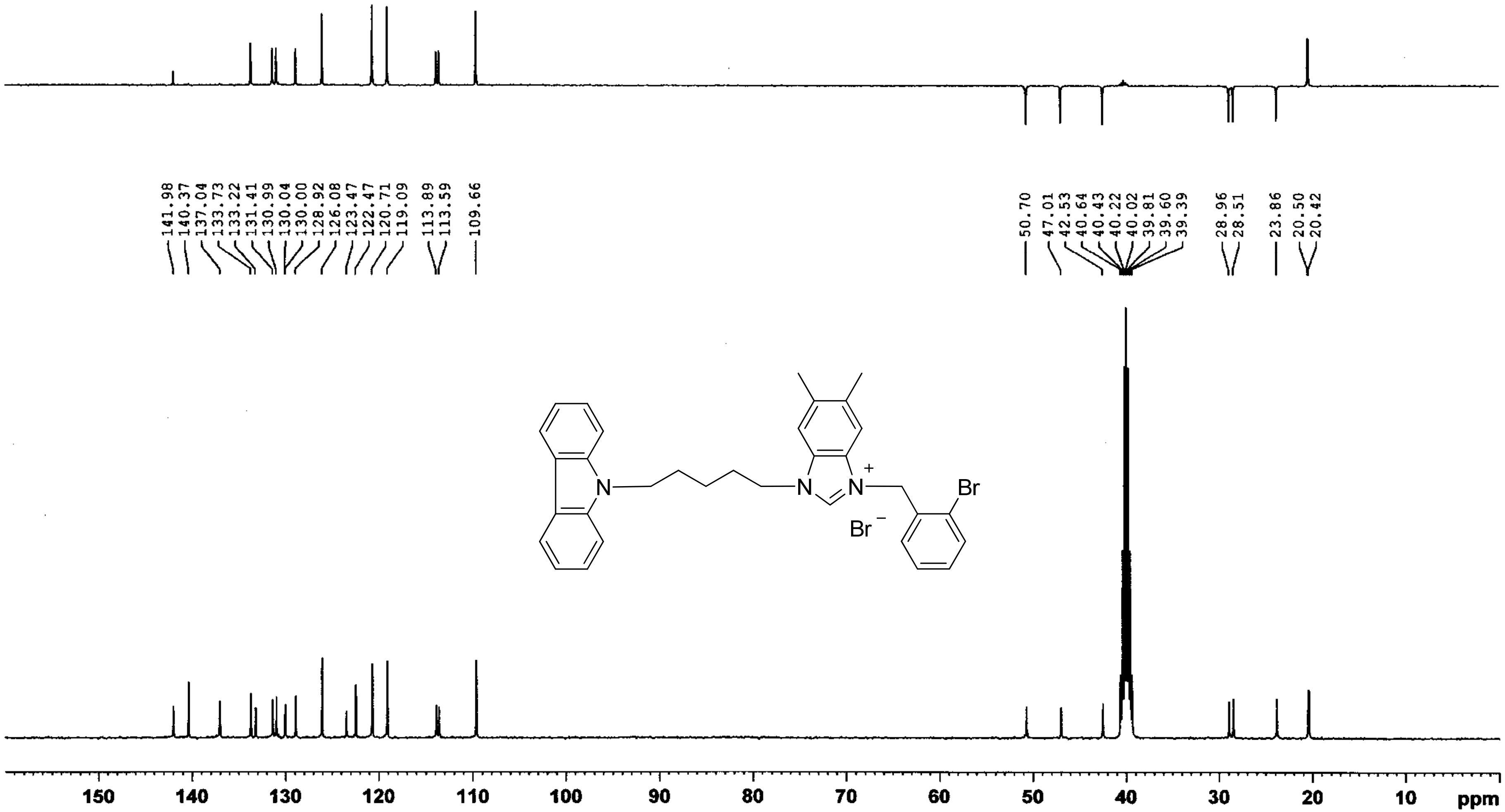
141.98
140.37
137.04
133.73
133.22
131.41
130.99
130.04
130.00
128.92
126.08
123.47
122.47
120.71
119.09
113.89
113.59
109.66
50.70
47.01
42.53
40.64
40.43
40.22
40.02
39.81
39.60
39.39
28.96
28.51
23.86
20.50
20.42
N
N
N
+
Br
Br
−
150
140
130
120
110
100
90
80
70
60
50
40
30
20
10
ppm
